# Supplementary figures and images for: Modeling bee movement shows how a perceptual masking effect can influence flower discovery
Source: PLoS Comput Biol. 2023 Mar 24;19(3):e1010558. doi: 10.1371/journal.pcbi.1010558 (PMC10075415; doi:10.1371/journal.pcbi.1010558)

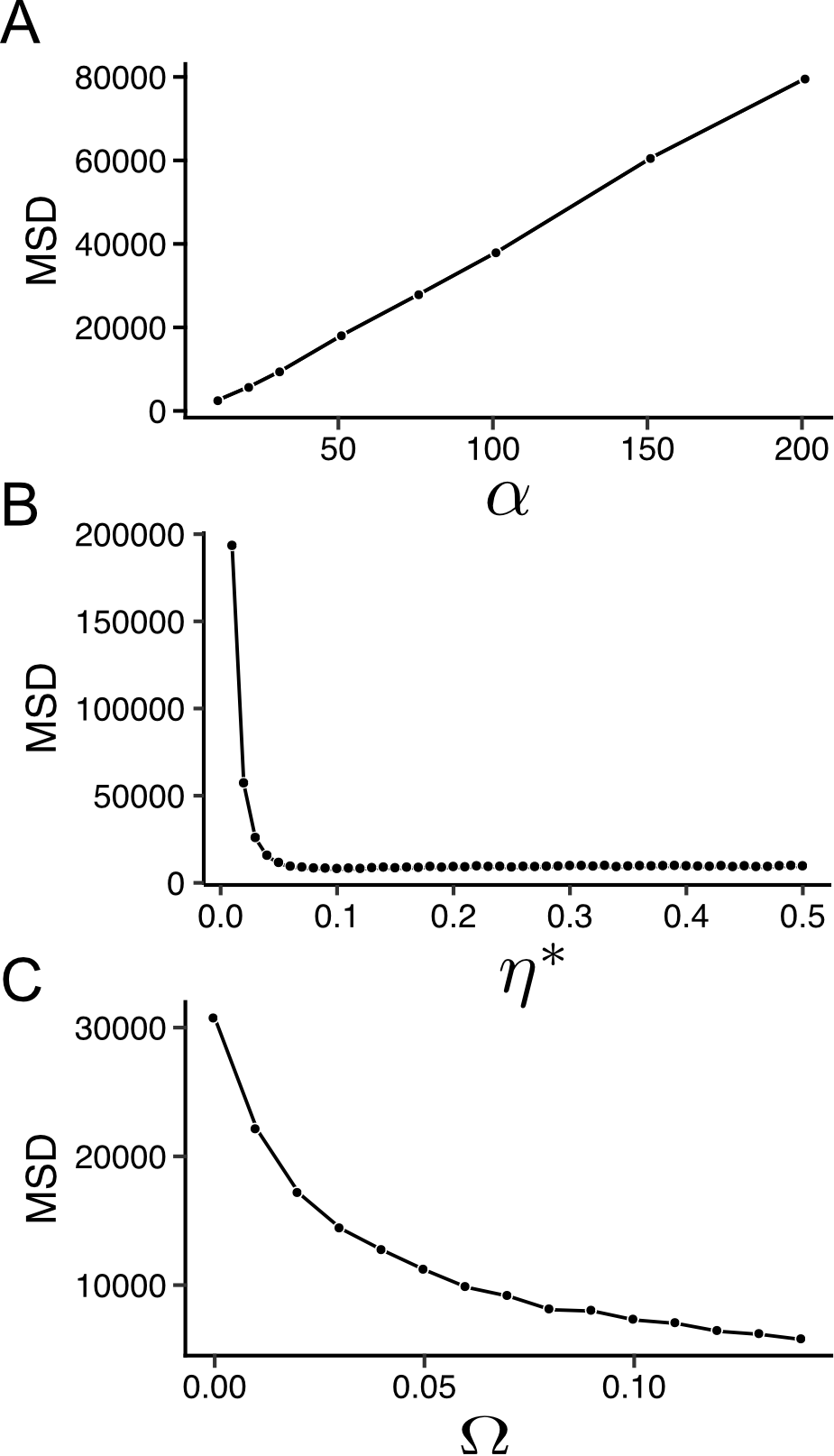

Supplement: S1 Fig — We numerically computed the MSD (m2), varying each parameter in turn, leaving the others unchanged (keeping their values fitted from the dataset). The MSD was estimated using 10^5 simulated loops for each point, using the default parameters γ = 1.0 s−1, σ = 0.37 rad/ s1/2, preturn = 1/30 s−1 and η* = 0.2 s-1 and varying (A) α (s), (B) η* (s−1) or (C) Ω=σ22γ (rad2/s-2) while leaving the other parameters unchanged. Note that for smaller values of η*, the distribution tends to (non stationary) diffusion, so, some loops are censored at one hour. The effect of α=1pturn, the mean duration in exploration mode, appears quite linear, which is not surprising but would call for an analytical demonstration. During the return phase, η* represents the intensity of the steering (the potential stiffness), since for smaller values of η*, the relaxation to the preferred turning speed would be less effective. As η* gets smaller and smaller values, the steering vanishes, so that the bee would adopts a diffusive behavior, with no Non Equilibrium Stationary State (NESS). On the other hand, we observe a clear effect of saturation for large enough values of η*, meaning that the effectiveness of the steering is limited by the relaxation time gamma. Finally, Ω controls the level of noise the turning speed can undergo. For Ω = 0, the turning speed has no noise at all, and the process becomes deterministic: bee would fly from the nest ballistically in the exploration phase and go back ballistically to the nest after turning maneuver induced by the steering process. In this case, given the initial condition of null turning speed, the trajectories would push bees the farthest from the nest during exploration (hence, maximal MSD), while larger values of Ω would drive bees to meander around the nest, leading to trajectories that remain closer to the nest. (TIFF) [file pcbi.1010558.s001.tiff]

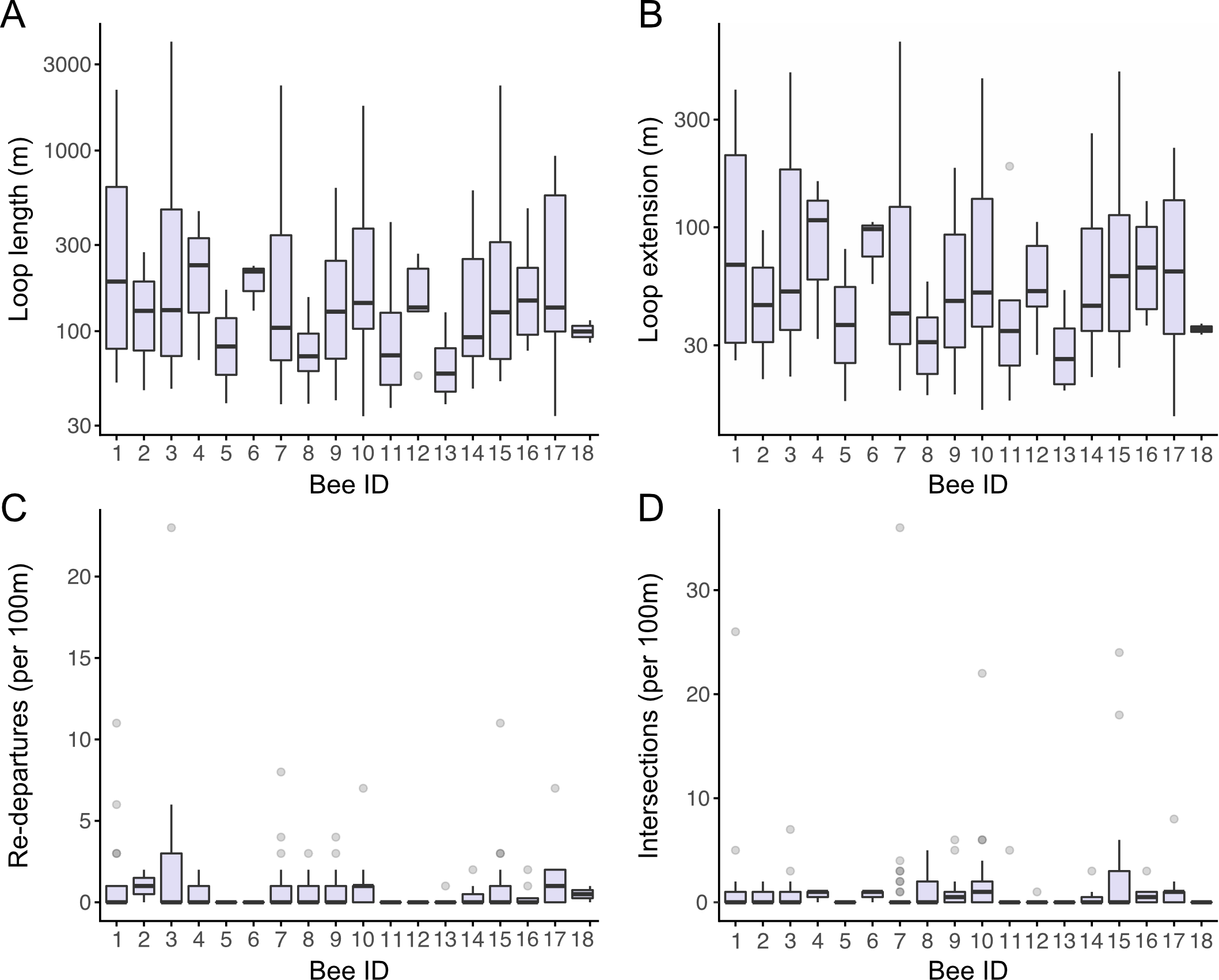

Supplement: S2 Fig — (A) Loop lengths (m) for each bee, as defined in Fig 3 in the main text. Boxplots, show the median (middle line), 25 and 75% quantiles (box), range of data within 1.5 interquartile deviations (whiskers), and outliers (dots). (B) Same as A but for the loop extension (maximum distance between the nest and the individual). (C) Same as A, but for the number of re-departures per 100m traveled. A re-departure is defined as three consecutive positions such that the second position is closer to the nest than the first one, but the third is again further away than the second. (D) Same as A but for the intersections (number of times the loop intersects with itself). (TIFF) [file pcbi.1010558.s002.tiff]

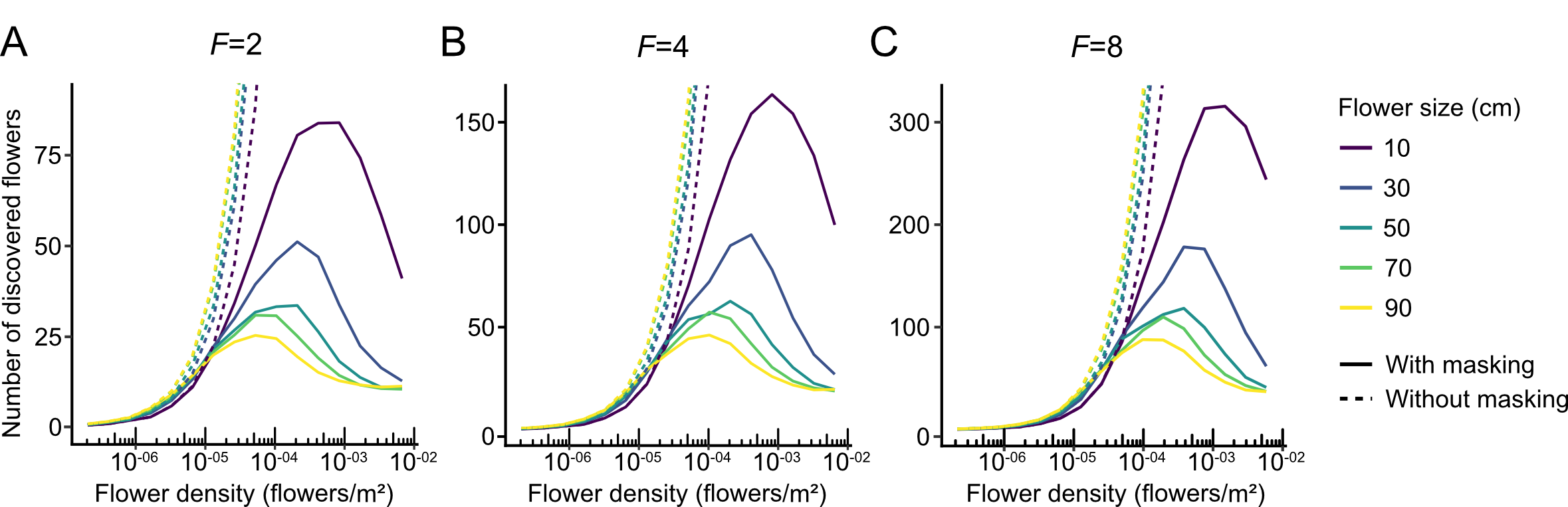

Supplement: S3 Fig — (A) Number of different flowers discovered in 100 exploration trips of 900 s, in an environment with randomly distributed flowers. Results are averaged over 80 simulations, keeping the environment fixed for every simulation. Solid lines: Number calculated taking into account the masking effect (i.e., only counting the first flowerF = 2 flowers that wereas discovered on each trip). Dotted lines: Probability calculated without taking into account the masking effect. (B) Same as (A), but for F = 4. (C) Same as (A), but for F = 8. Note the difference of scales for the ordinates. (TIFF) [file pcbi.1010558.s003.tiff]

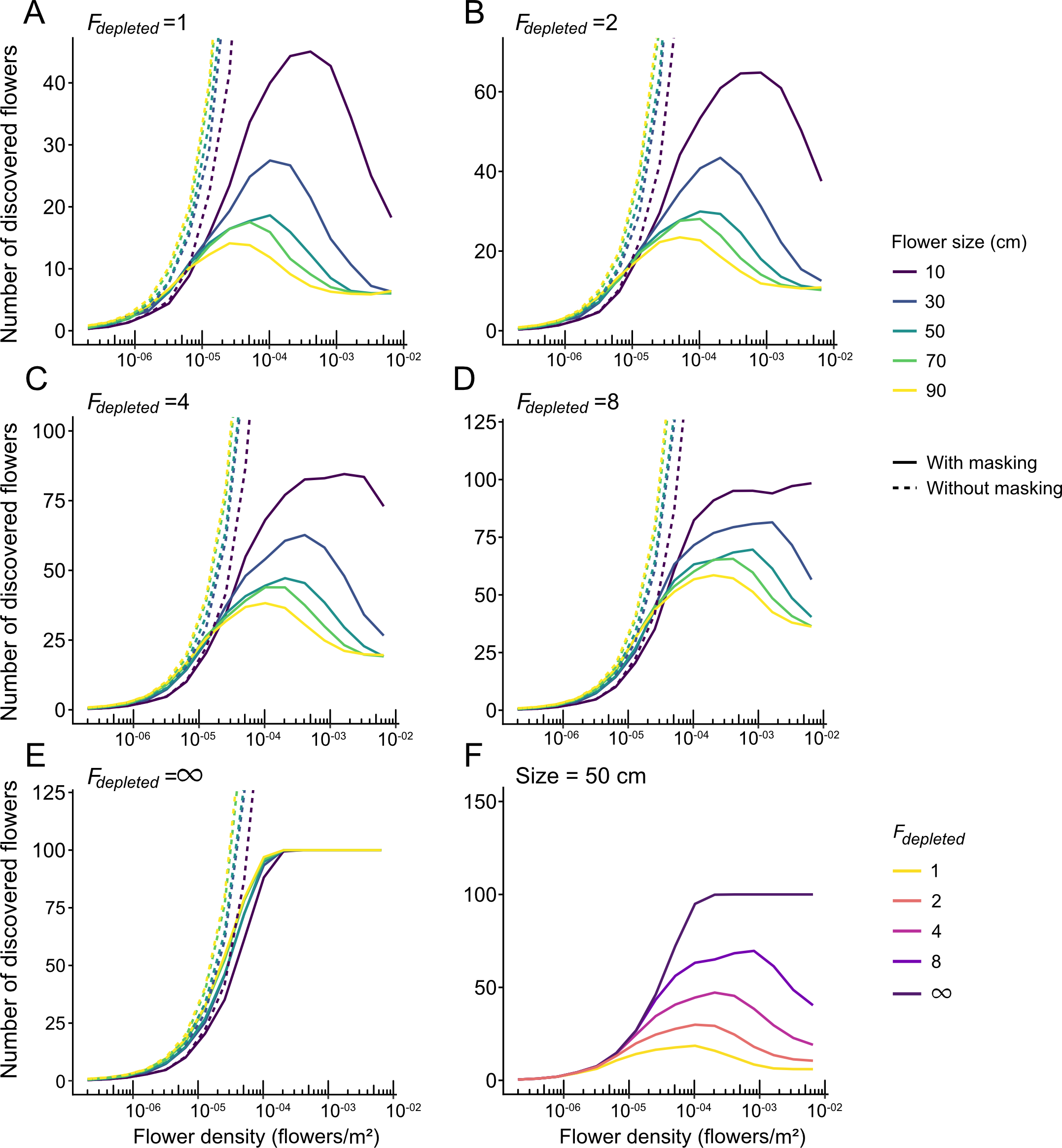

Supplement: S4 Fig — Here we assumed that an individual will continue exploring after visiting an already-explored flower, and will return to the nest only when encountering a fresh flower or after a fixed number of visits to already-visited flowers (Fdepleted). (A) Number of different flowers discovered in 100 exploration trips of 900 s, in an environment with randomly distributed flowers, and with Fdepleted. = 1 (note that this value of Fdepleted makes the simulation identical to that in the main text). Results are averaged over 80 simulations, keeping the environment fixed for every simulation. Solid lines: With masking effect. Dotted lines: Without masking effect (the bee does not react to previous flower encounters, and all discovered flowers were counted). Colors correspond to different flower sizes. (B) Same as A but with Fdepleted = 2. (C) Same as A but with Fdepleted = 4. (D) Same as A but with Fdepleted = 8. (E) Same as A but with Fdepleted = ∞. (F) Same as A but for a given flower size (50 cm), with colors representing the value of Fdepleted. (TIFF) [file pcbi.1010558.s004.tiff]
