# Supplementary material for: Modeling bee movement shows how a perceptual masking effect can influence flower discovery
Source: PLoS Comput Biol. 2023 Mar 24;19(3):e1010558. doi: 10.1371/journal.pcbi.1010558 (PMC10075415; doi:10.1371/journal.pcbi.1010558)
Supplement: S1 Text — (PDF) [file pcbi.1010558.s005.pdf]

Supplemental Information For The Article
Modeling bee search shows how a perceptual masking effect can influence foraging efficiency and pollination

Moran et al.

September, 2022

Contents

1 Statistics of tracks **2**

2 Parameters fitting **5**
- 2.1 Best Fit : 5
- 2.2 Fits with qT<0.07 5
- 2.3 Figures for each rating 6
- 2.4 Predictions for $\alpha \in (0, 10, 20, 30, 50, 100)$ 11
  - 2.4.1 Predictions for $\alpha = 10$ 11
  - 2.4.2 Predictions for $\alpha = 20$ 12
  - 2.4.3 Predictions for $\alpha = 30$ (in the main text) 13
  - 2.4.4 Predictions for $\alpha = 50$ 14
  - 2.4.5 Predictions for $\alpha = 75$ 15
  - 2.4.6 Predictions for $\alpha = 100$ 16
  - 2.4.7 Predictions for $\alpha = 150$ 17
  - 2.4.8 Predictions for $\alpha = 200$ 18
  - 2.4.9 Sensitivity of MSD to parameters (see Main Text) 19

3 First Hitting locations for bumblebee **20**

4 Hitting statistics for flowers **22**

5 Individual tracks **24**

This document results from the execution of the R-script 00-DoAll.R

It contains raw results and figures for the full analysis and simulations.

1 Statistics of tracks

We use 01-Data/01-AllLoopsSevilla.csv data file.

For each observable, min, first quartile, median, third quartile, max values are:

```
Loop Length : [1]    7.82   43.96   88.97  245.04 3981.56


Loop extension : [1]  14.5  30.8  45.1 105.5 664.8


Speed : [1] 0.3 3.1 4.2 4.8 6.2


Intersects : [1]  0  0  0  1 36


Redeparts : [1]  0  0  0  1 23
```

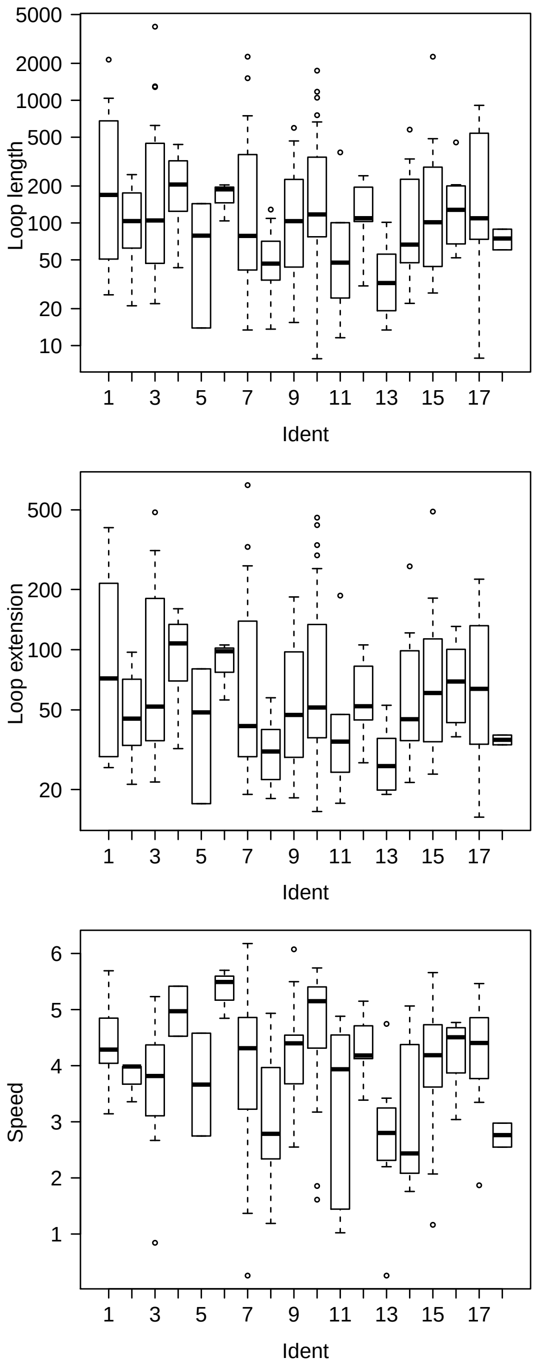

Fig SI-I-1 : Individual statistics for loop length, extension and speed.

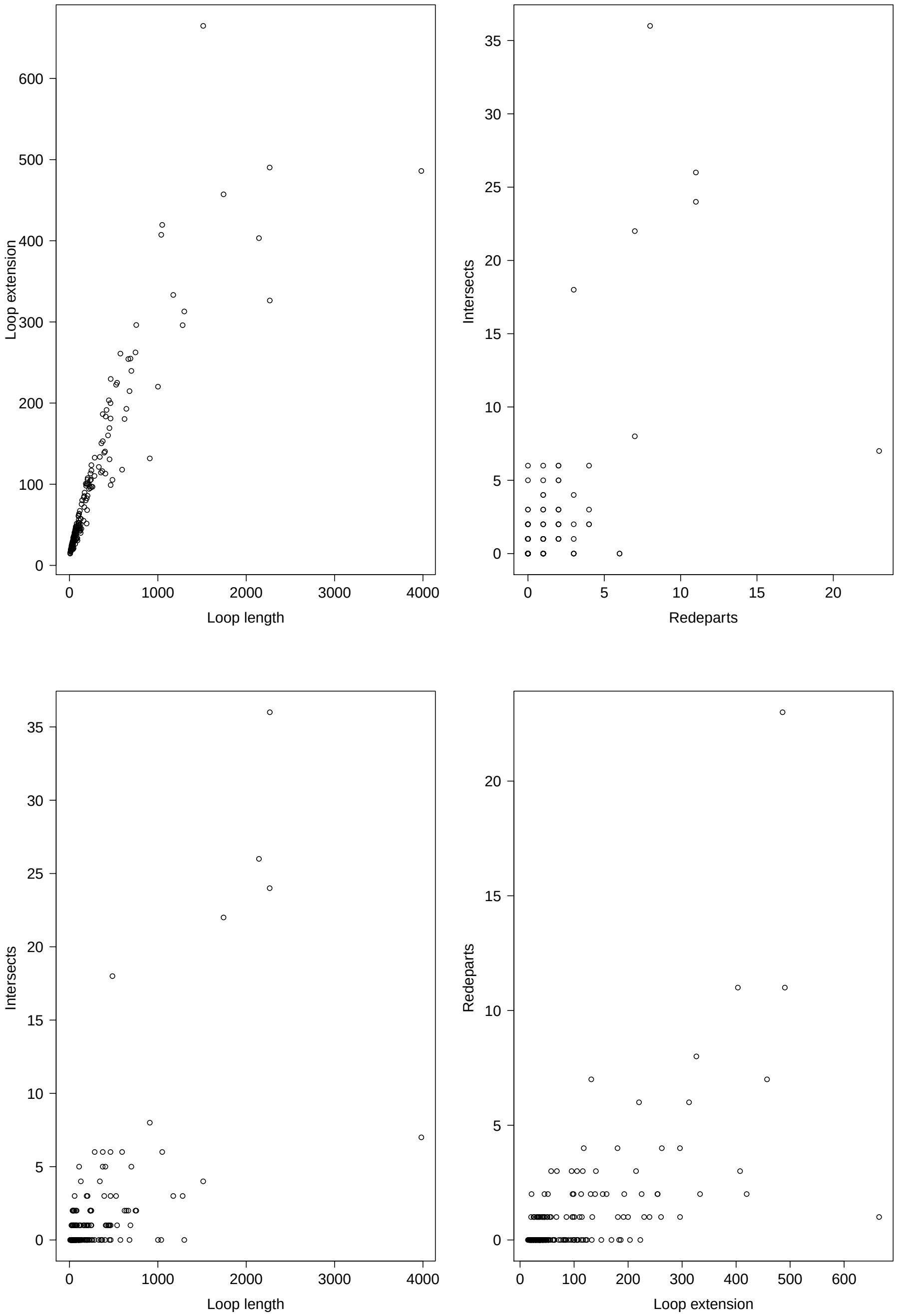

Fig SI-I-2 : Correlations between loop length, extension, intersects and redepartures.

2 Parameters fitting

We use ../01/10-AllLoopData.csv data file.

Fixed Parameters :

Nest size : 0.680834

Perception Distance : 13

Flying speed : 3.938

2.1 Best Fit :

	Gamma	Omega	Alpha	Eta	Length	MaxAway	Intersects	Redeparts	qL
3053	1	0.07	30	0.2	109.0678	23.15209	0.189227	0.13827	0.01769481

	qM	qI	qR	qT
3053	0.0150974	0.06331169	0.04188312	0.03449675

The variables q[L,M,I,R] give the respective quantiles for Length, MaxAway, Intersects and Redeparts.

The variables qT gives the average quantile.

2.2 Fits with qT<0.07

In order to check that the best fit is not a peculiar point, we show all combinations for which $qT < 0.07$

	Gamma	Omega	Alpha	Eta	Length	MaxAway	Intersects	Redeparts	qL
1934	0.8	0.07	30	0.25	120.3157	24.64490	0.1763490	0.1697972	0.06672078
2493	0.9	0.07	30	0.20	106.7304	23.18055	0.1649982	0.2014622	0.01217532
3045	1.0	0.07	25	0.20	112.2967	23.47995	0.2069530	0.1445516	0.02905844
3053	1.0	0.07	30	0.20	109.0678	23.15209	0.1892270	0.1382700	0.01769481
4181	1.2	0.07	35	0.20	110.4792	27.77261	0.1737675	0.1407206	0.02142857
4277	1.2	0.09	25	0.20	110.6016	21.47902	0.2095936	0.1728932	0.02224026
4797	1.3	0.08	35	0.20	108.0654	24.43073	0.1933003	0.1608860	0.01444805
4853	1.3	0.09	35	0.20	107.9349	26.29172	0.1964997	0.1276662	0.01412338
5405	1.4	0.09	30	0.20	110.8849	24.56493	0.1994103	0.1425561	0.02337662

	qM	qI	qR	qT
1934	0.03522727	0.02694805	0.09788961	0.05669643
2493	0.01558442	0.01185065	0.16038961	0.05000000
3045	0.01801948	0.13603896	0.05194805	0.05876623
3053	0.01509740	0.06331169	0.04188312	0.03449675
4181	0.12500000	0.02224026	0.04529221	0.05349026
4277	0.00275974	0.14805195	0.10357143	0.06915584
4797	0.03051948	0.07857143	0.08198052	0.05137987
4853	0.08035714	0.09009740	0.02678571	0.05284091
5405	0.03360390	0.10275974	0.04902597	0.05219156

2.3 Figures for each rating

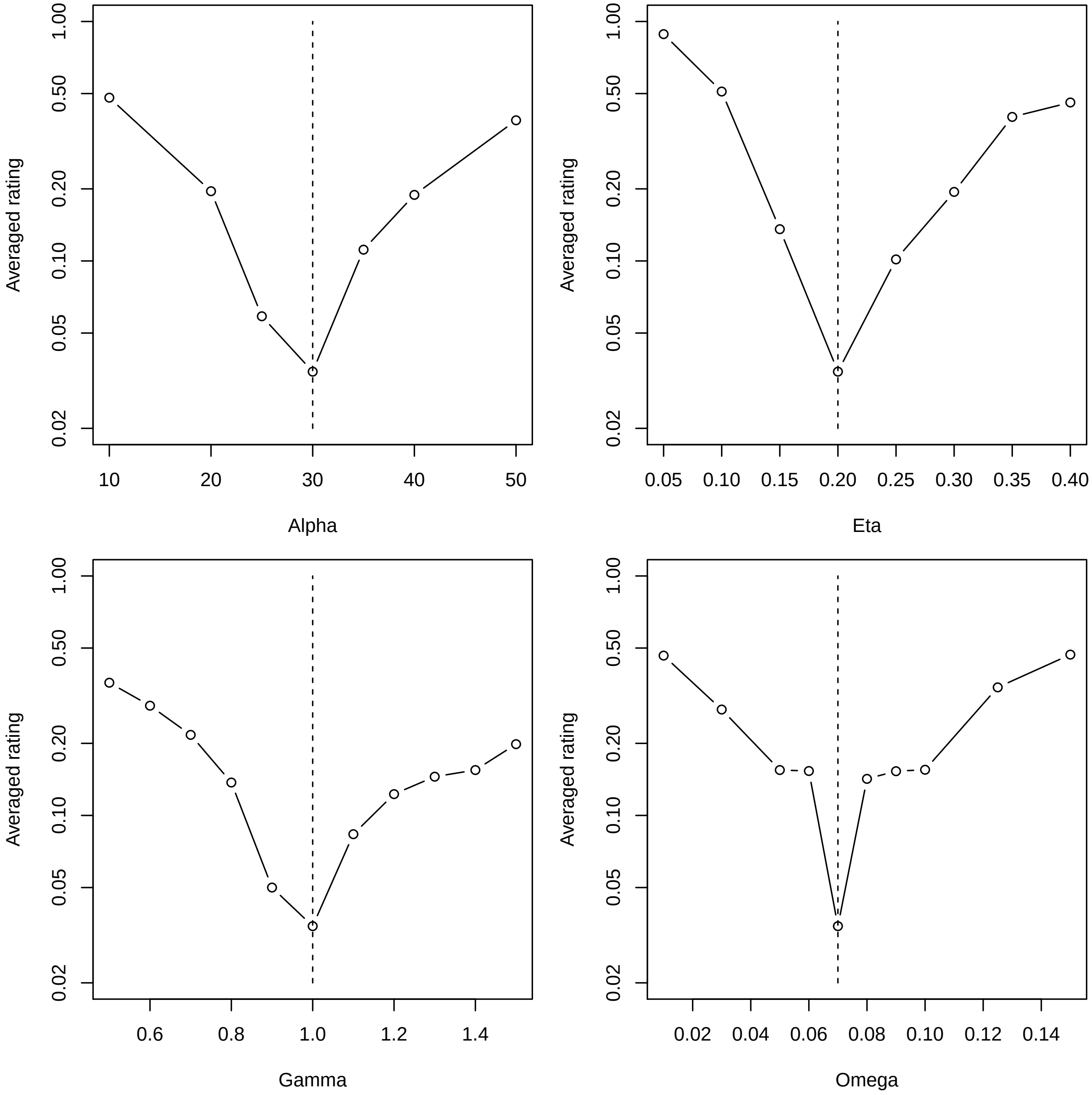

Fig SI-III-1 : Average ratings for each parameter, when others are kept constant.

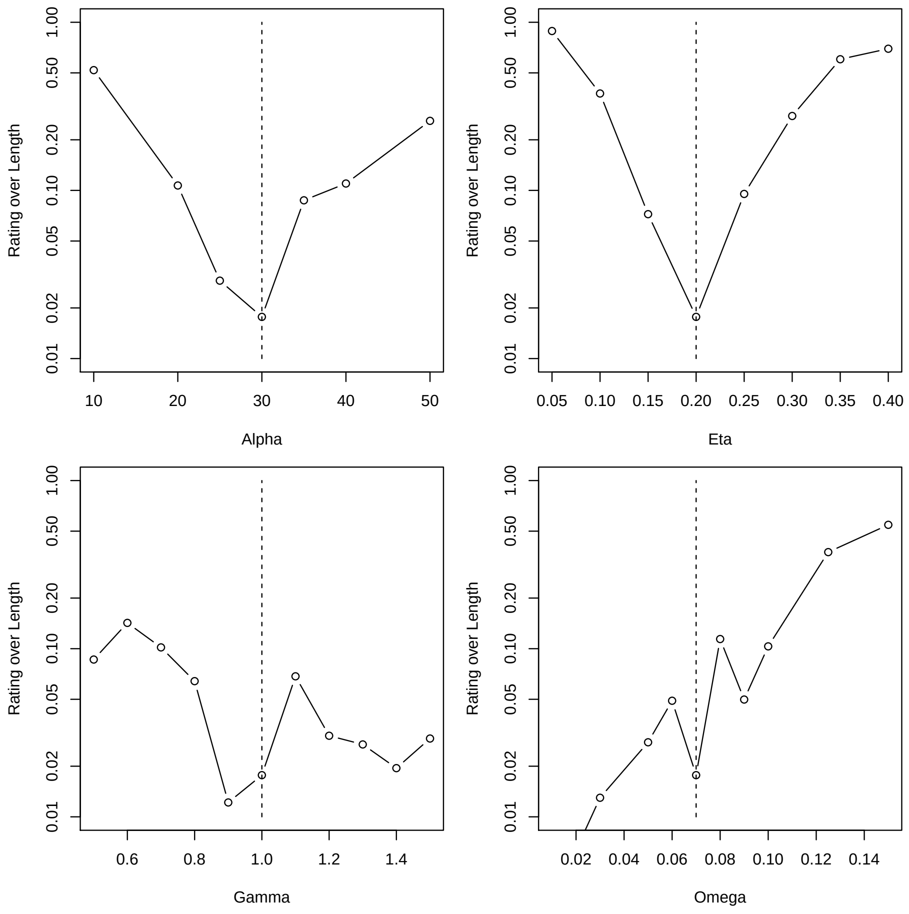

Fig SI-III-2 : Ratings for loop length for each parameter, when others are kept constant.

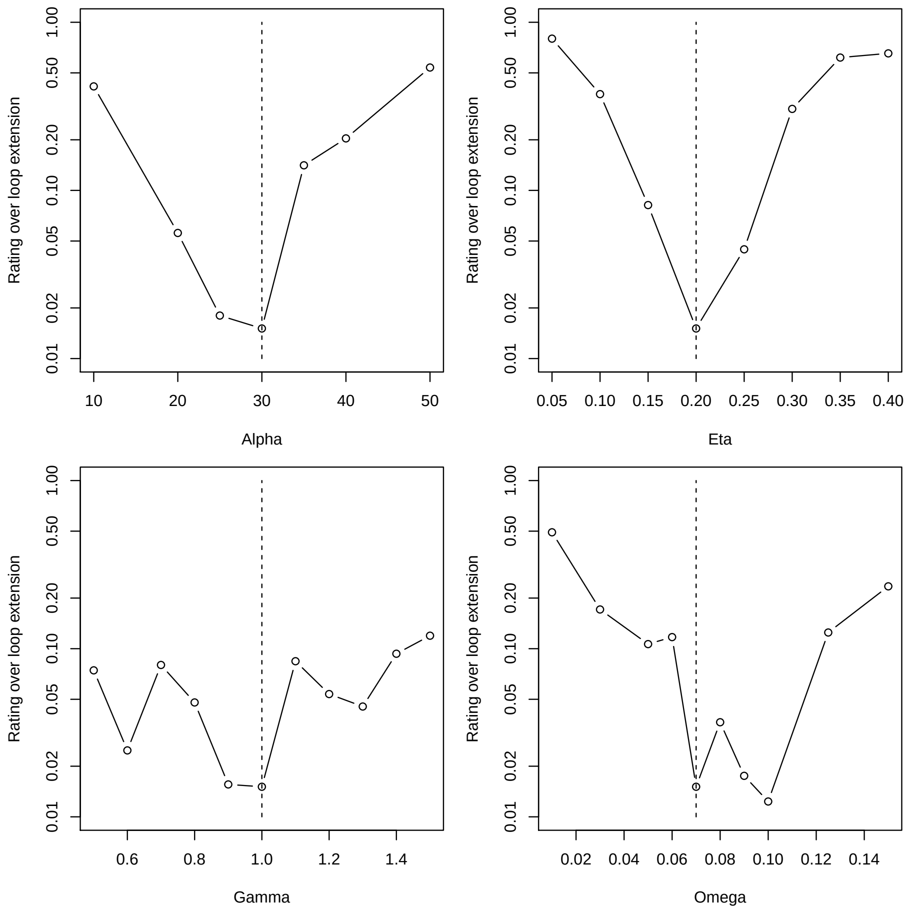

Fig SI-III-3 : Ratings for loop extension for each parameter, when others are kept constant.

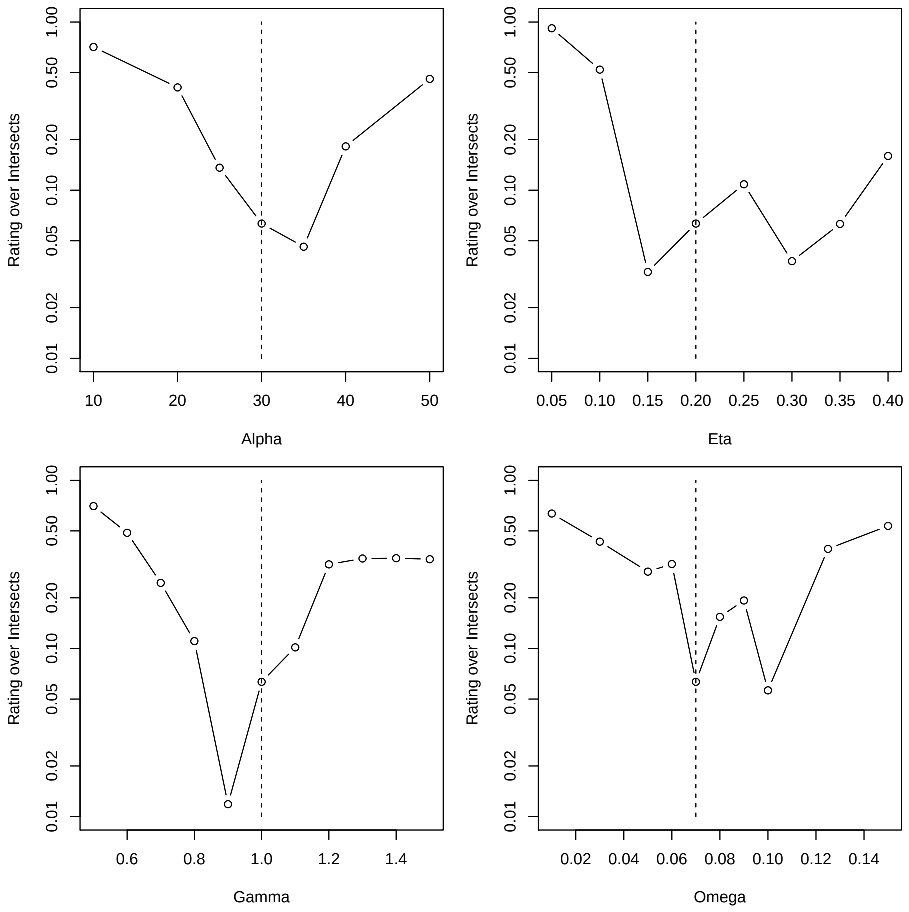

Fig SI-III-4 : Ratings for intersects for each parameter, when others are kept constant.

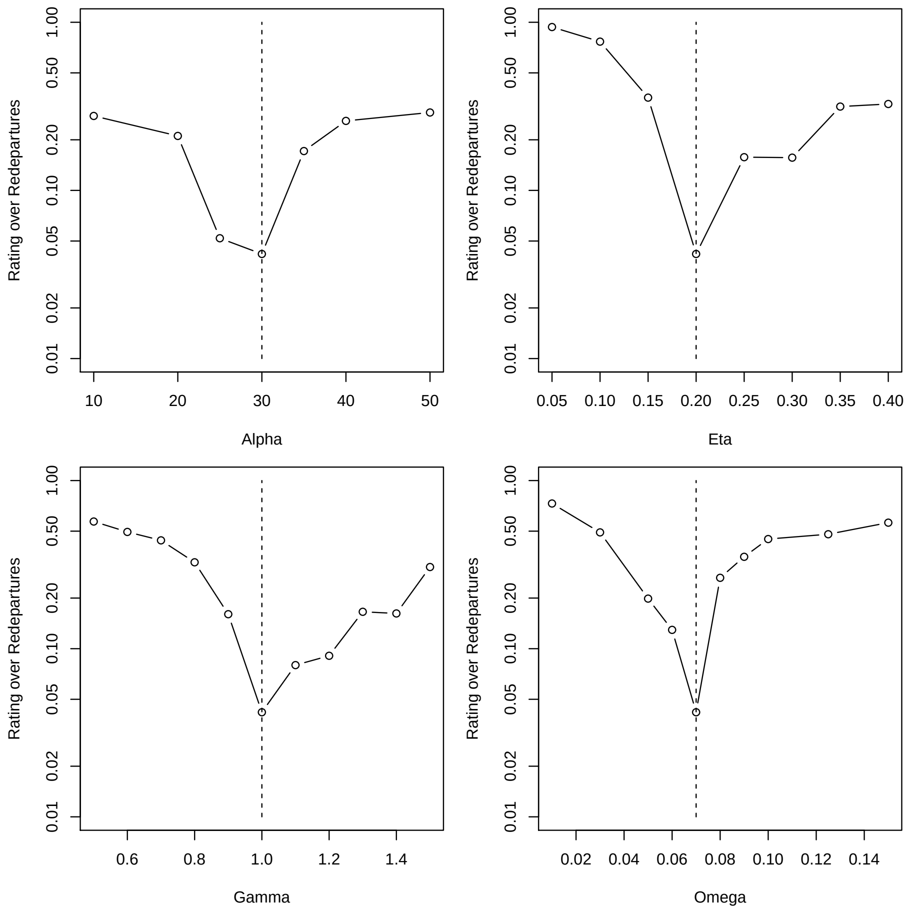

Fig SI-III-5 : Ratings for redepartures for each parameter, when others are kept constant.

2.4 Predictions for $\alpha \in (0, 10, 20, 30, 50, 100)$

2.4.1 Predictions for $\alpha = 10$

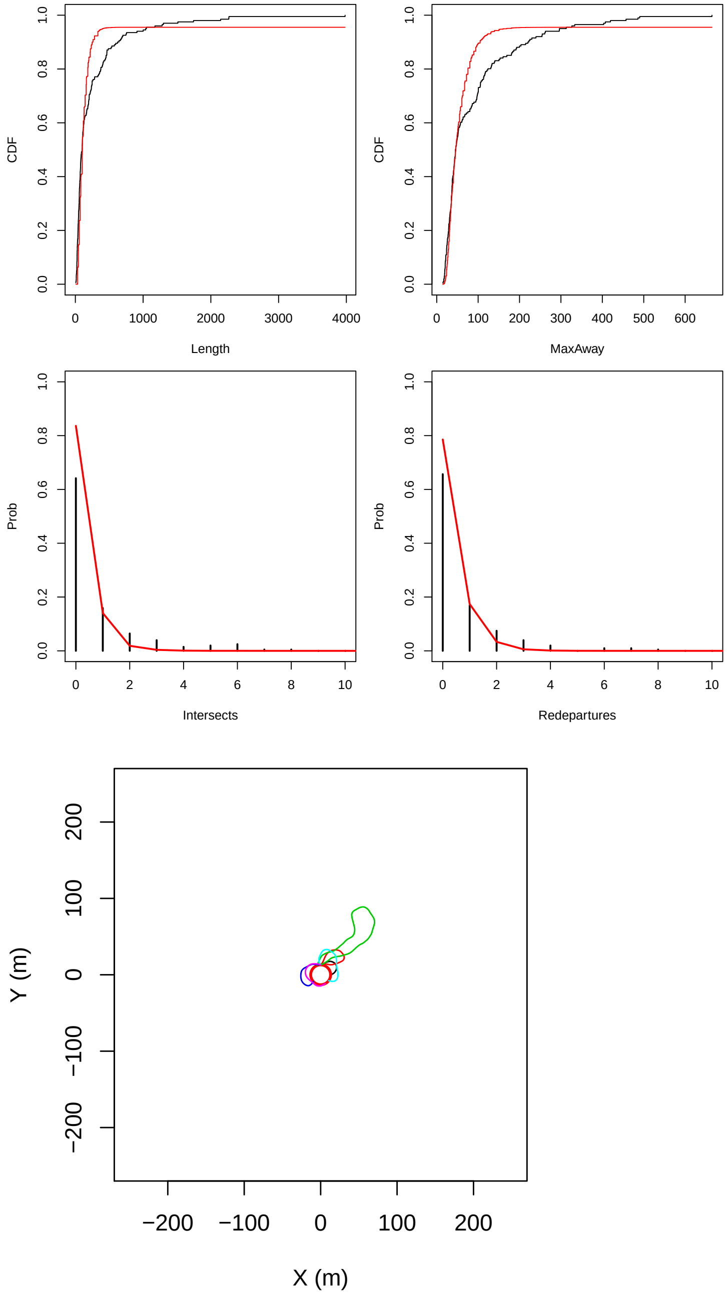

Fig SI-III-6 : Predictions for $\alpha = 10$.

2.4.2 Predictions for $\alpha = 20$

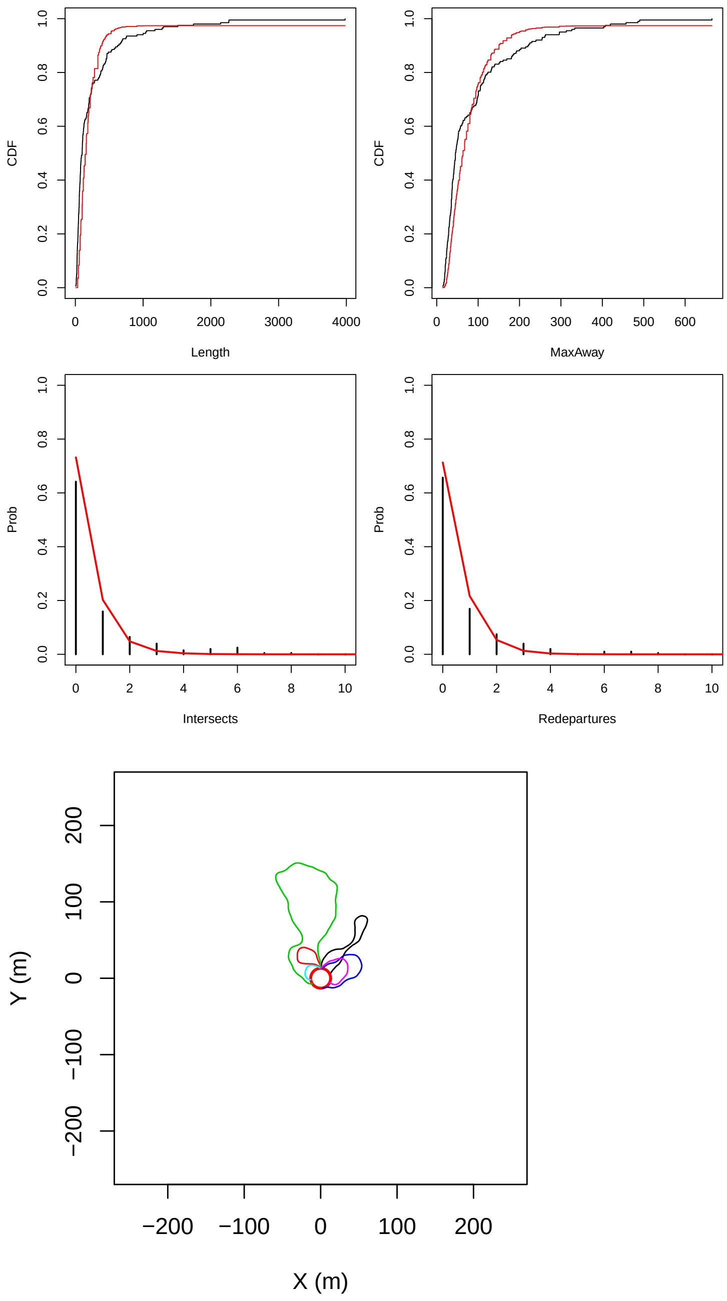

Fig SI-III-7 : Predictions for $\alpha = 20$.

2.4.3 Predictions for $\alpha = 30$ (in the main text)

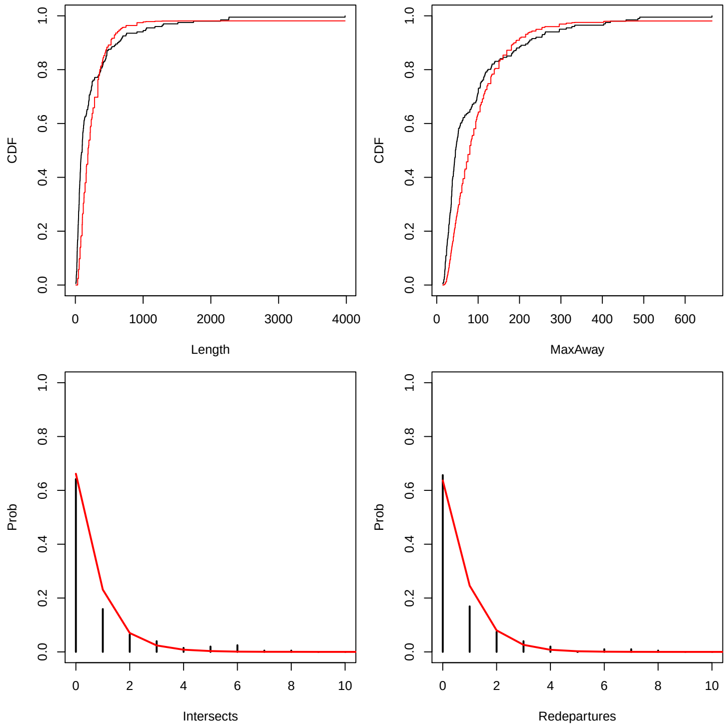

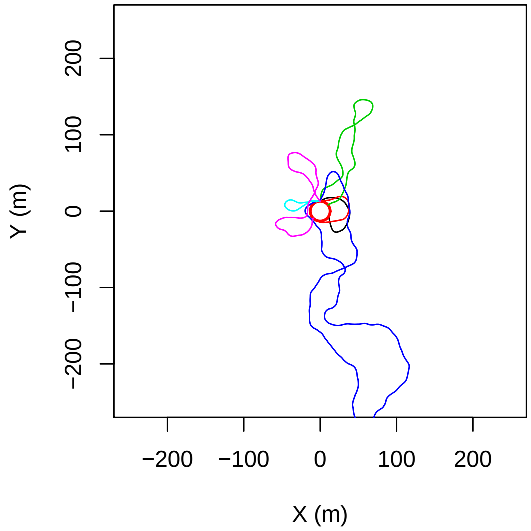

Fig SI-III-8 : Predictions for $\alpha = 30$.

2.4.4 Predictions for $\alpha = 50$

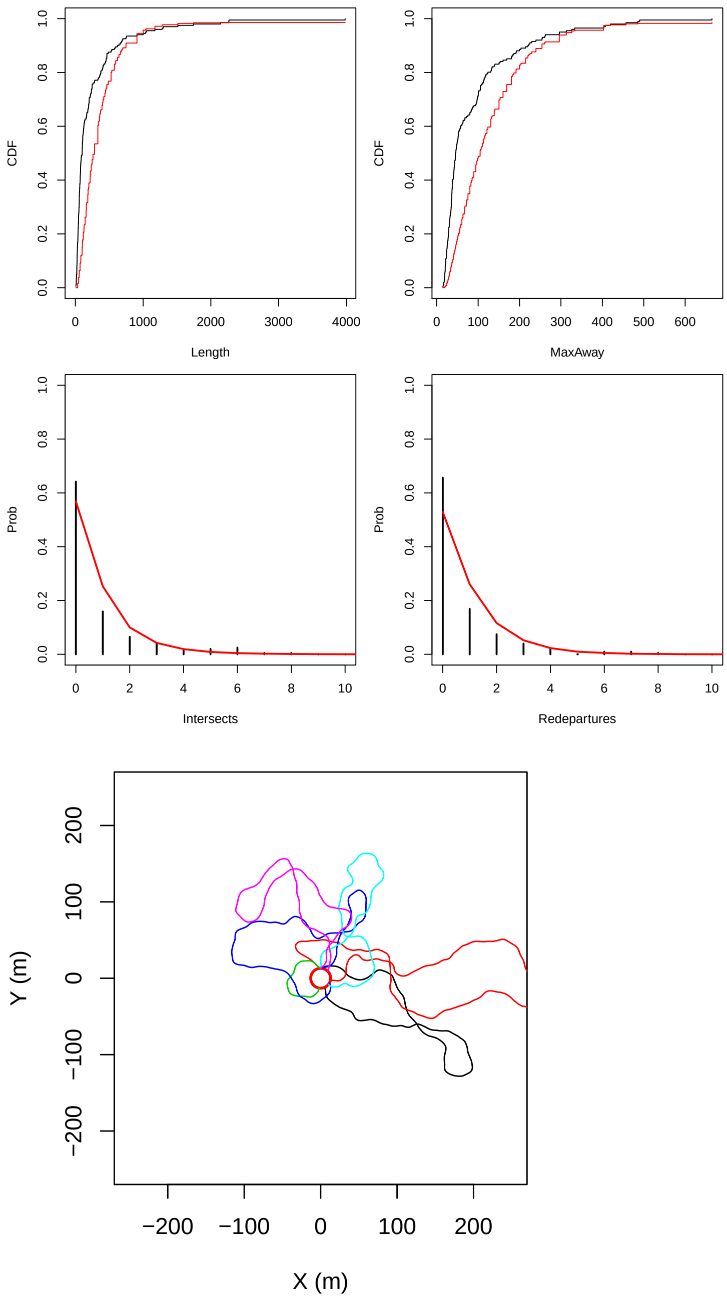

Fig SI-III-9 : Predictions for $\alpha = 50$.

2.4.5 Predictions for $\alpha = 75$

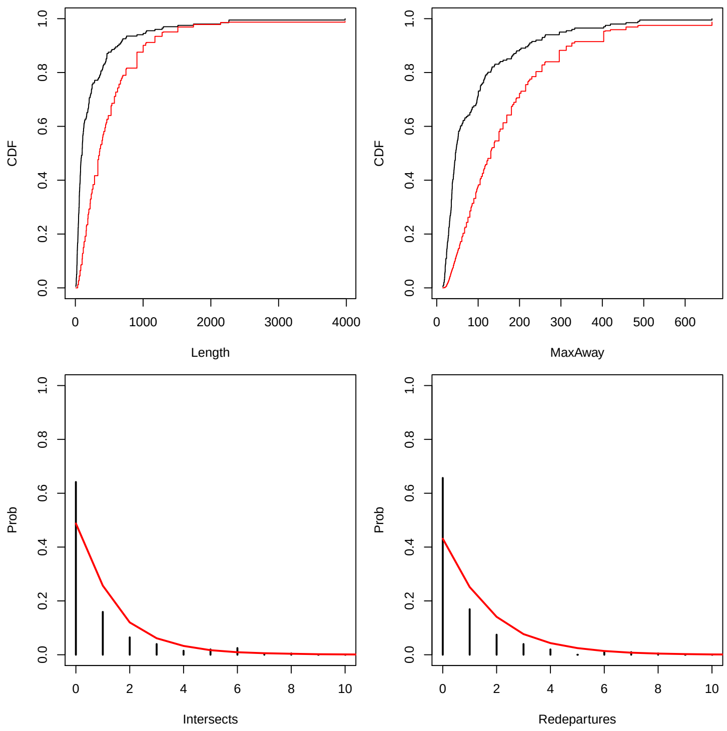

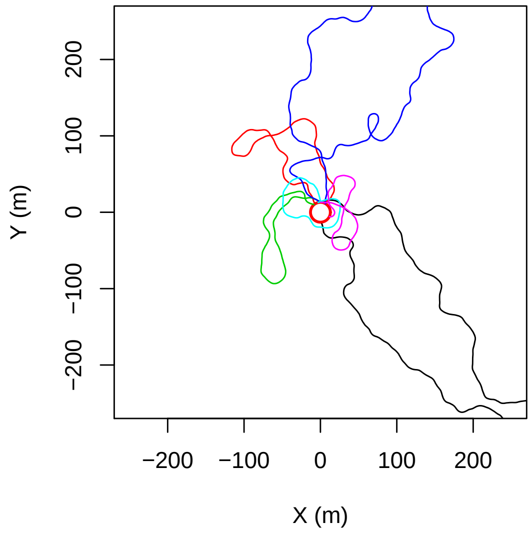

Fig SI-III-10 : Predictions for $\alpha = 75$.

2.4.6 Predictions for $\alpha = 100$

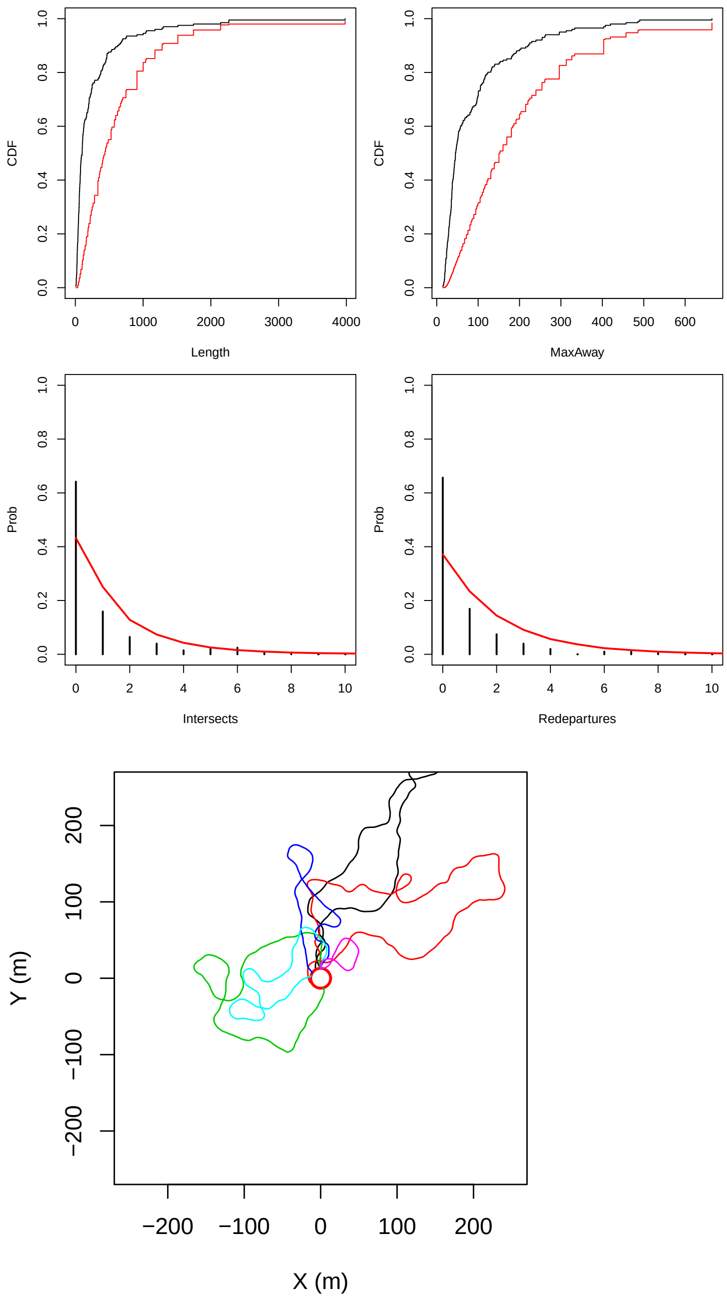

Fig SI-III-11 : Predictions for $\alpha = 100$.

2.4.7 Predictions for $\alpha = 150$

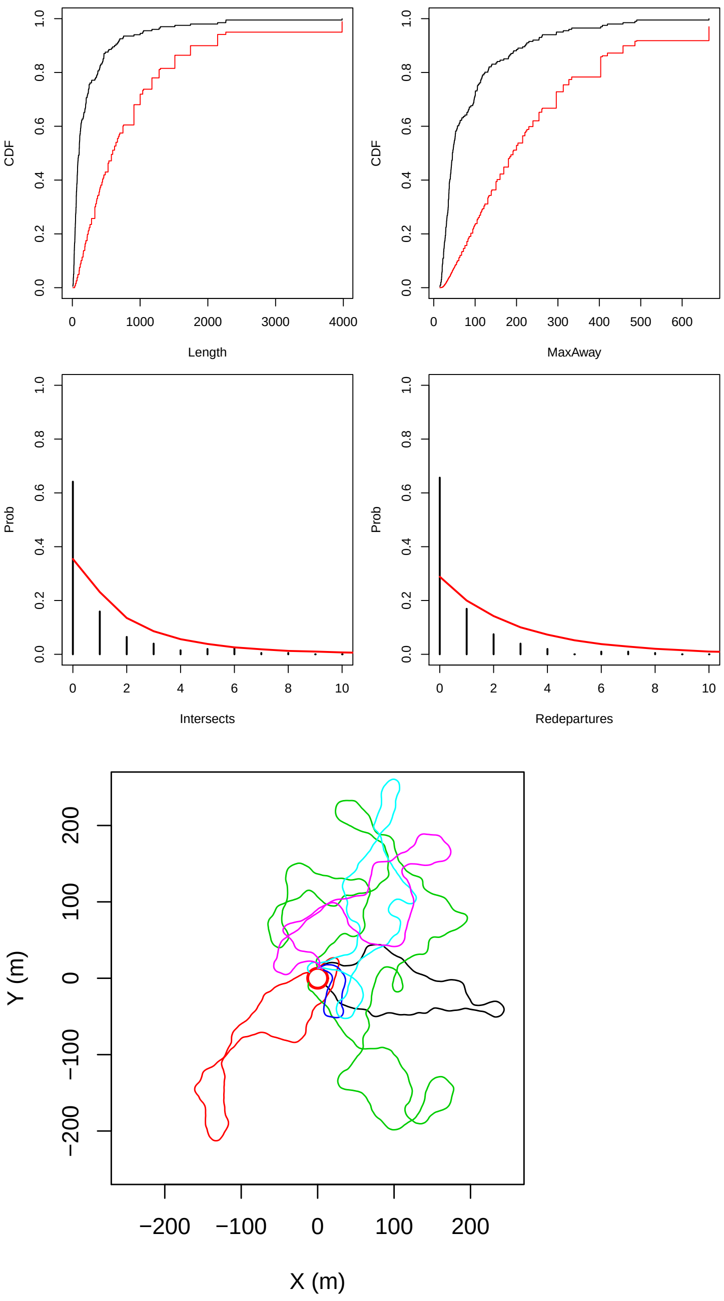

Fig SI-III-12 : Predictions for $\alpha = 150$.

2.4.8 Predictions for $\alpha = 200$

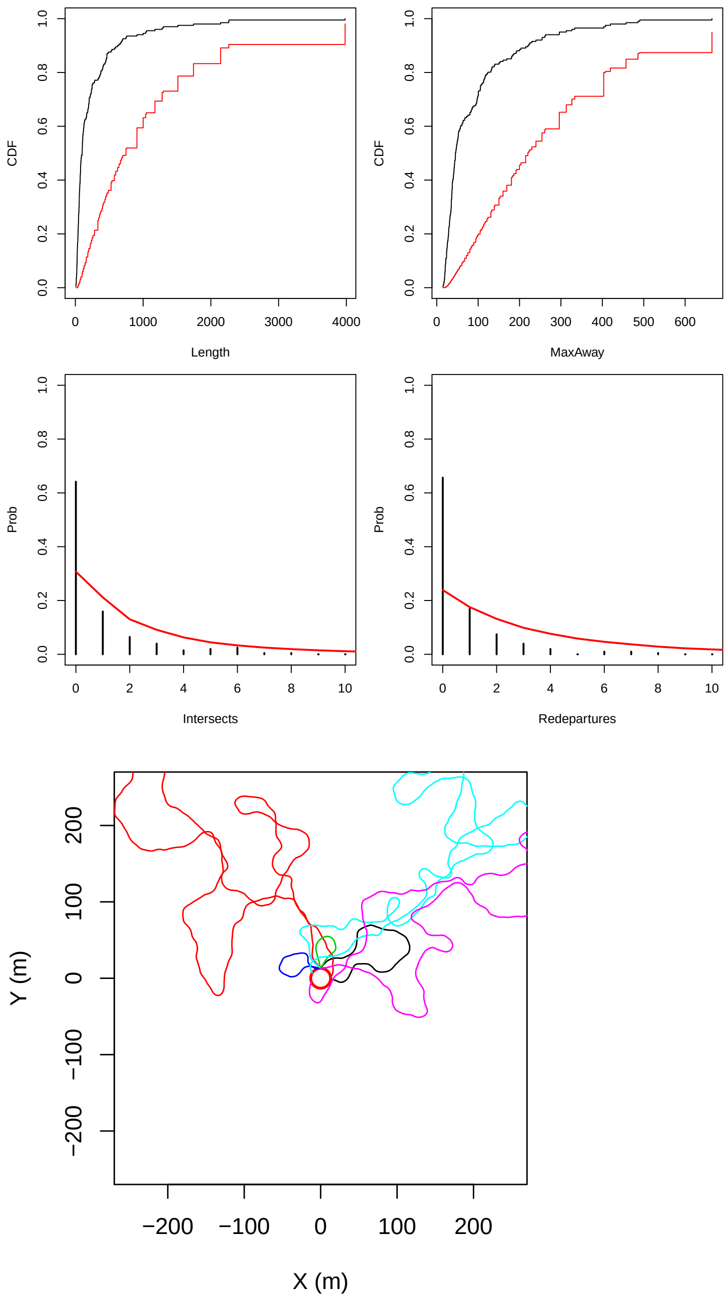

Fig SI-III-13 : Predictions for $\alpha = 200$.

2.4.9 Sensitivity of MSD to parameters (see Main Text)

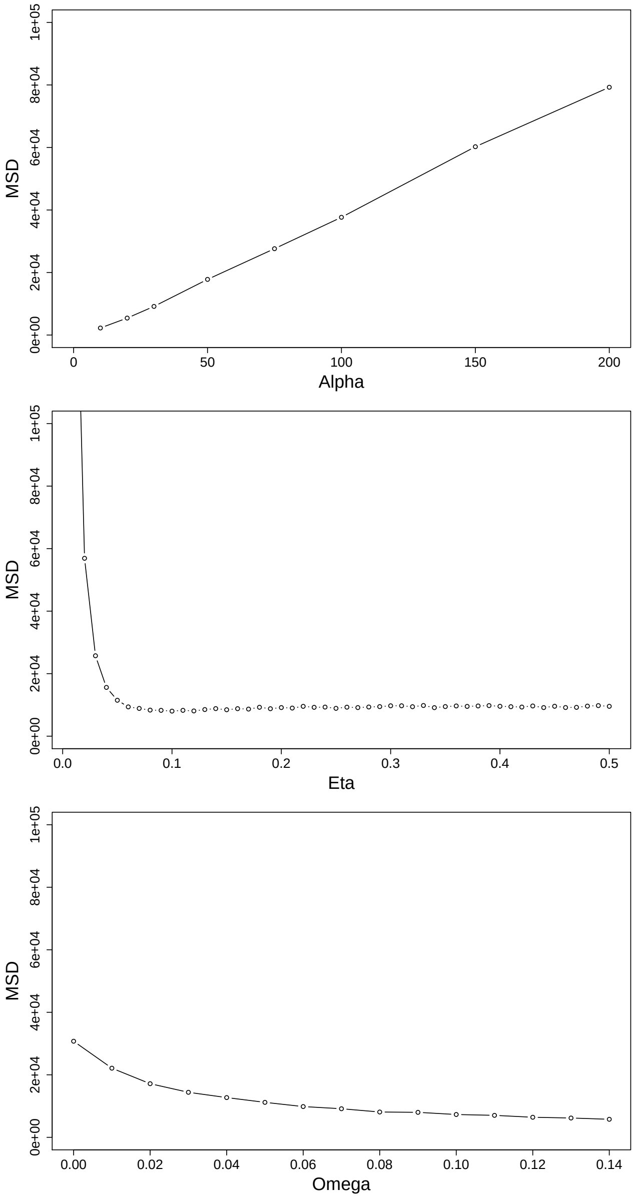

Fig SI-III-14 : Sensitivity of MSD (m^2) to α, η and Ω.

3 First Hitting locations for bumblebee

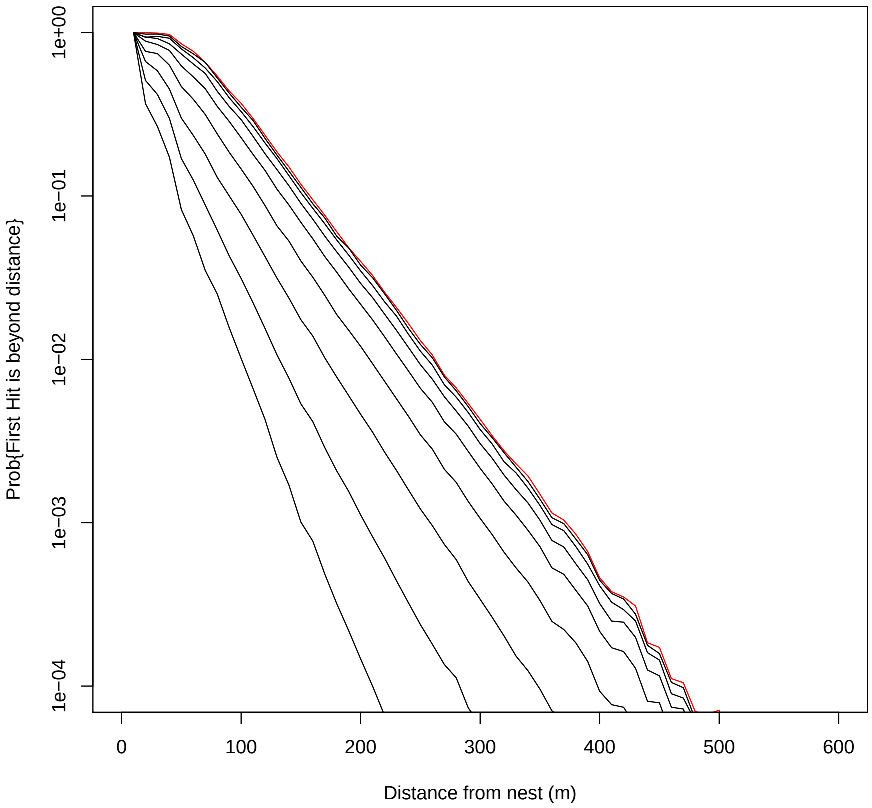

Fig SI-IV-1 : Locations (distance from nest) of first hit flower for CPF-PTW, depending on flower density (survival function, see main text).

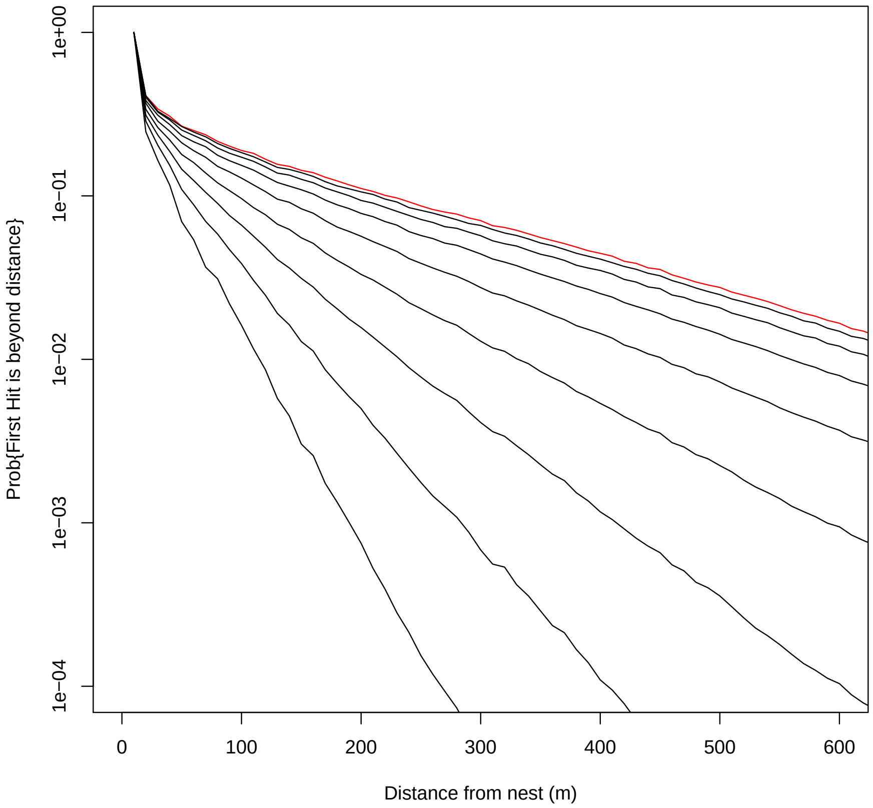

Fig SI-IV-2 : Location (distance from nest) of first hit flower for PTW.

4 Hitting statistics for flowers

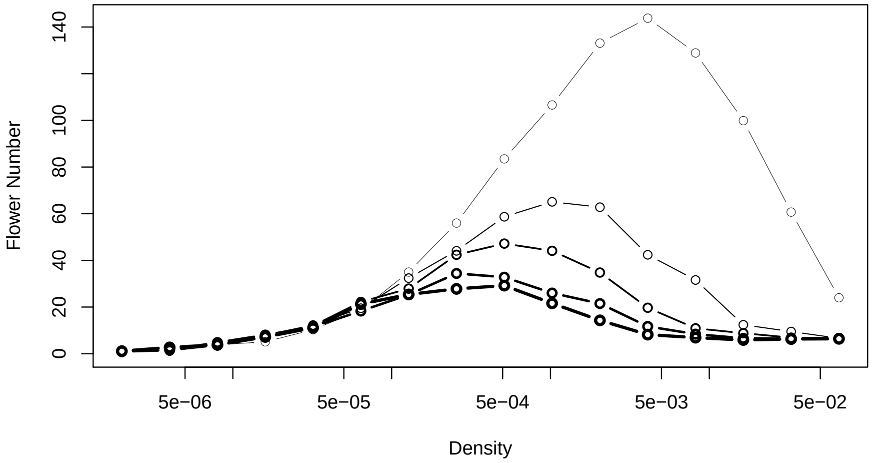

Fig SI-V-1 : Number of disctinct flower visited, depending on density, for CPF-PTW (see main text).

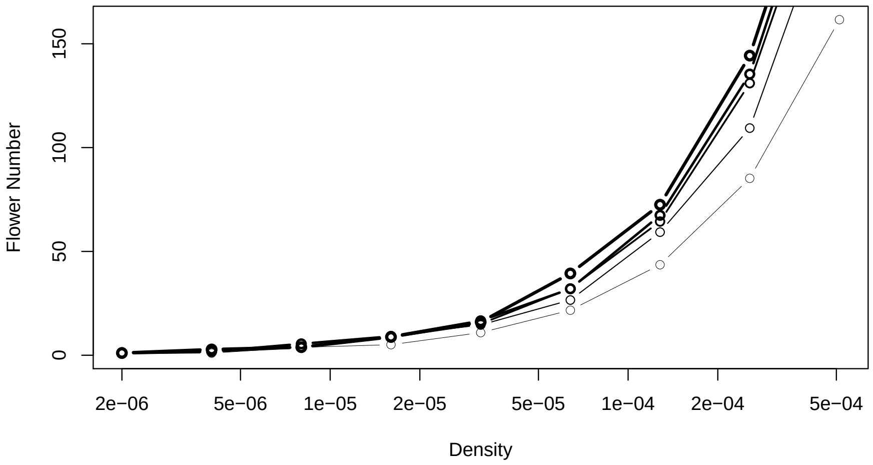

Fig SI-V-2 : Number of total flower found (see main text).

5 Individual tracks

Here, we report the tracks used for the calibration.

For each recording session, we first report the whole track, separating loops by color.

Then, we report each loop separately.

For some loops, black line reports the original data, and the red line reports the loop we actually used, after summarizing all the points detected in an area within 6 m of an artificial flower as a single point at the location of the flower.

List of Track Ids:

```
 [1] "JR-1" "JR-2" "JR-3" "JS-1" "JS-2" "JS-3" "GH-1" "GE-1" "GE-2" "JS-4"
[11] "JP-2" "GH-3" "JT-1" "JT-2" "JT-3" "KC-2" "JP-3" "JP-4" "JP-5" "KH-1"
[21] "GF-1" "GE-3" "GS-2" "KG-1" "JN-4" "JU-1" "LL-1" "LE-1" "GX-1" "KO-1"
[31] "KH-2" "JY-1"
```

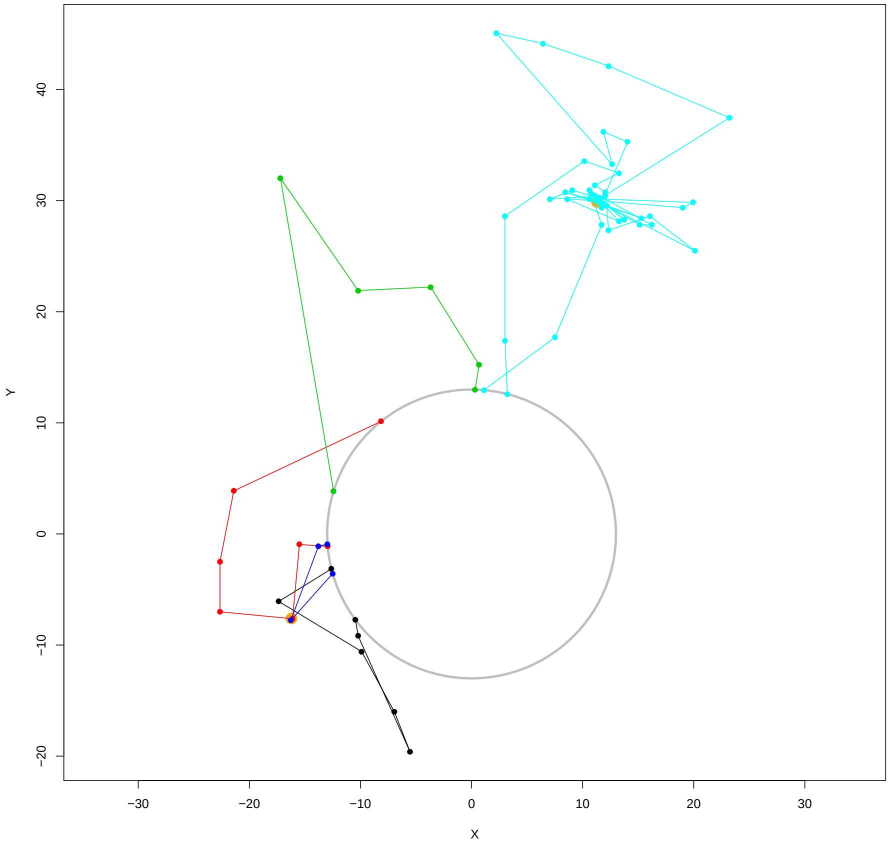

Fig SI-V-1 : Tracks of bee JR-1

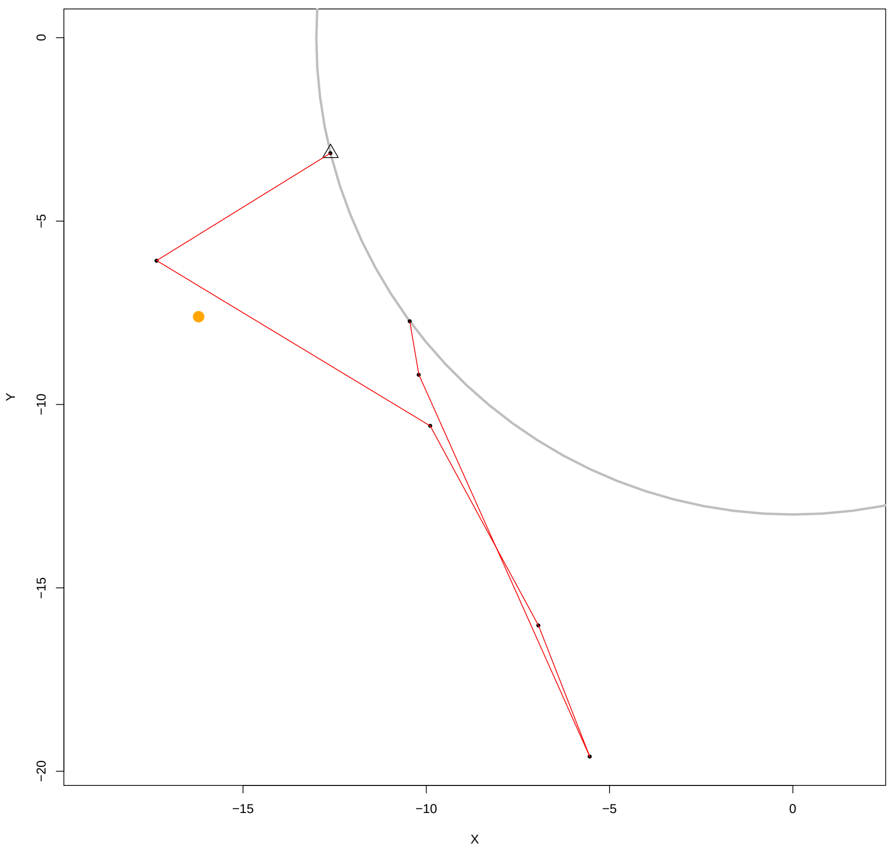

Fig SI-V-1.1 : Loop 1 — loop 1 of bee JR-1

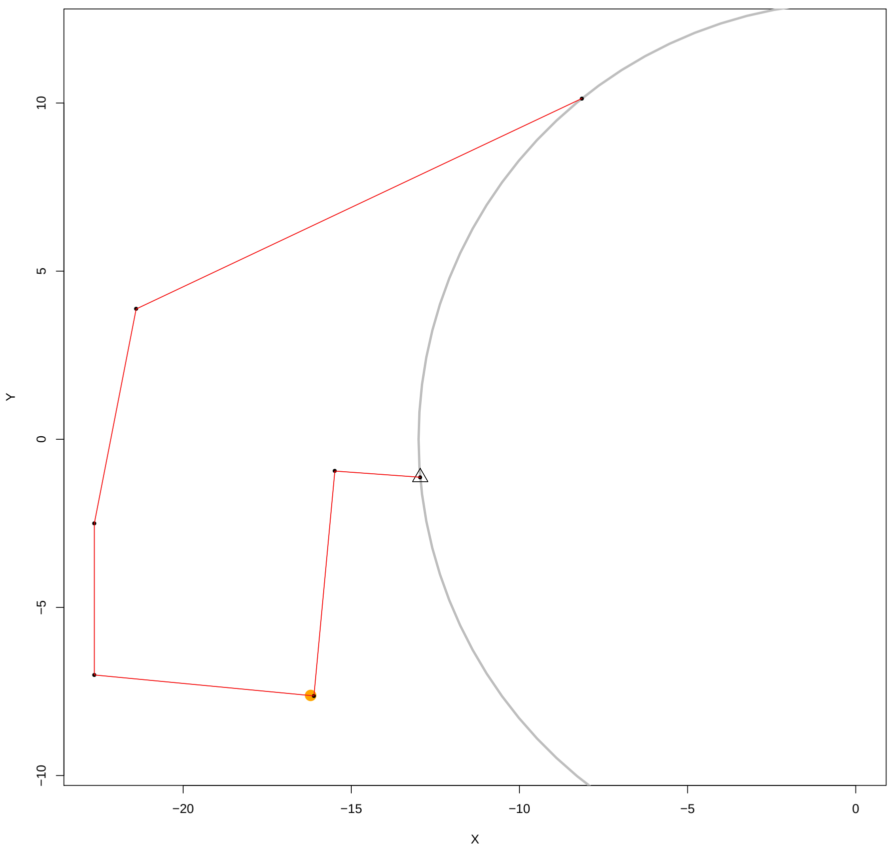

Fig SI-V-1.2 : Loop 2 — loop 2 of bee JR-1

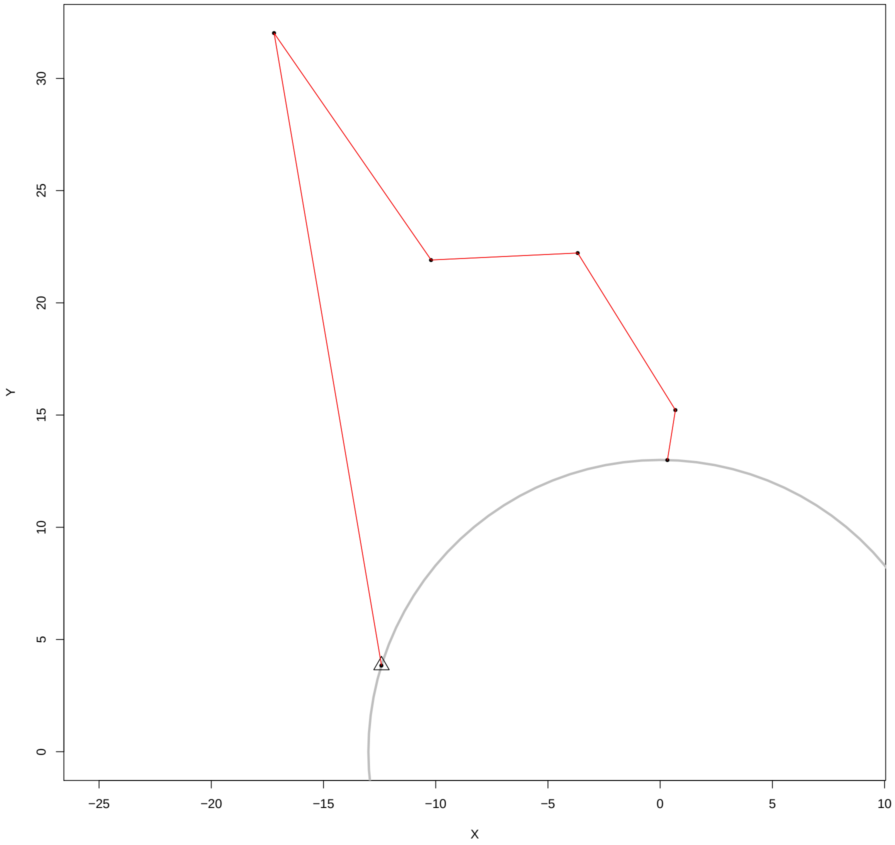

Fig SI-V-1.3 : Loop 3 — loop 3 of bee JR-1

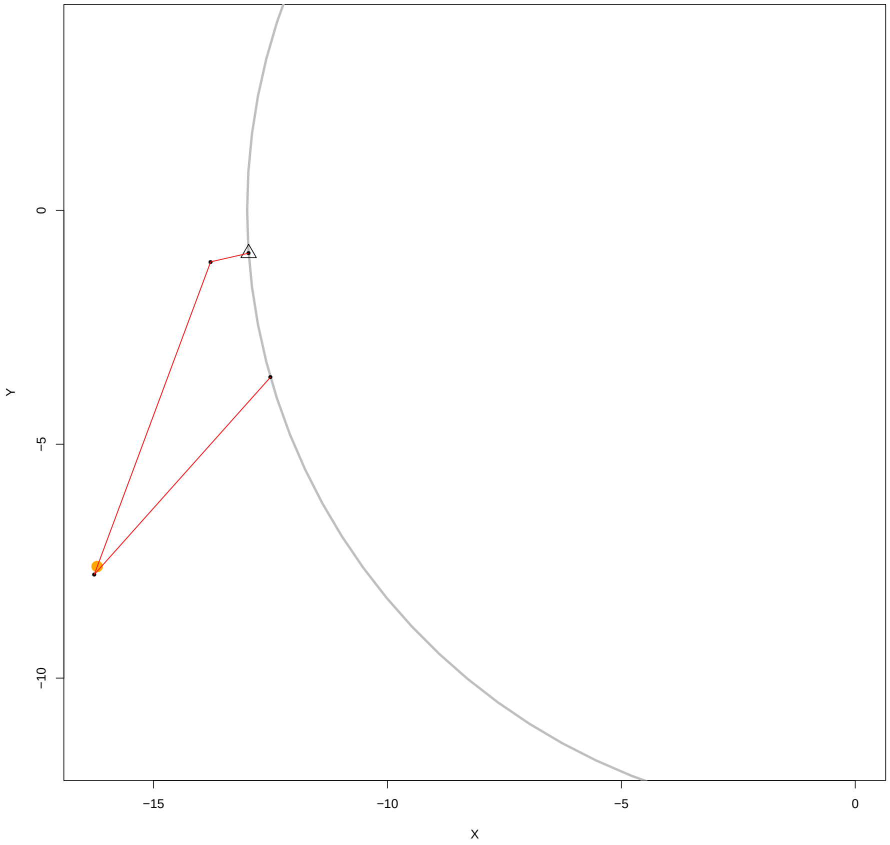

Fig SI-V-1.4 : Loop 4 — loop 4 of bee JR-1

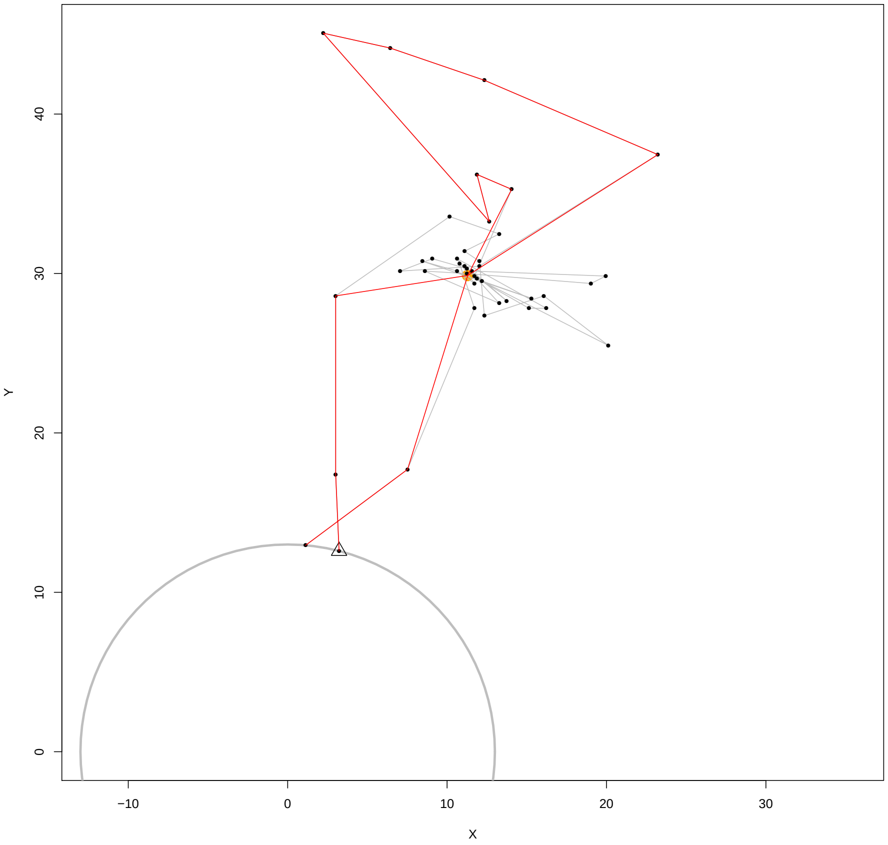

Fig SI-V-1.5 : Loop 5 — loop 5 of bee JR-1

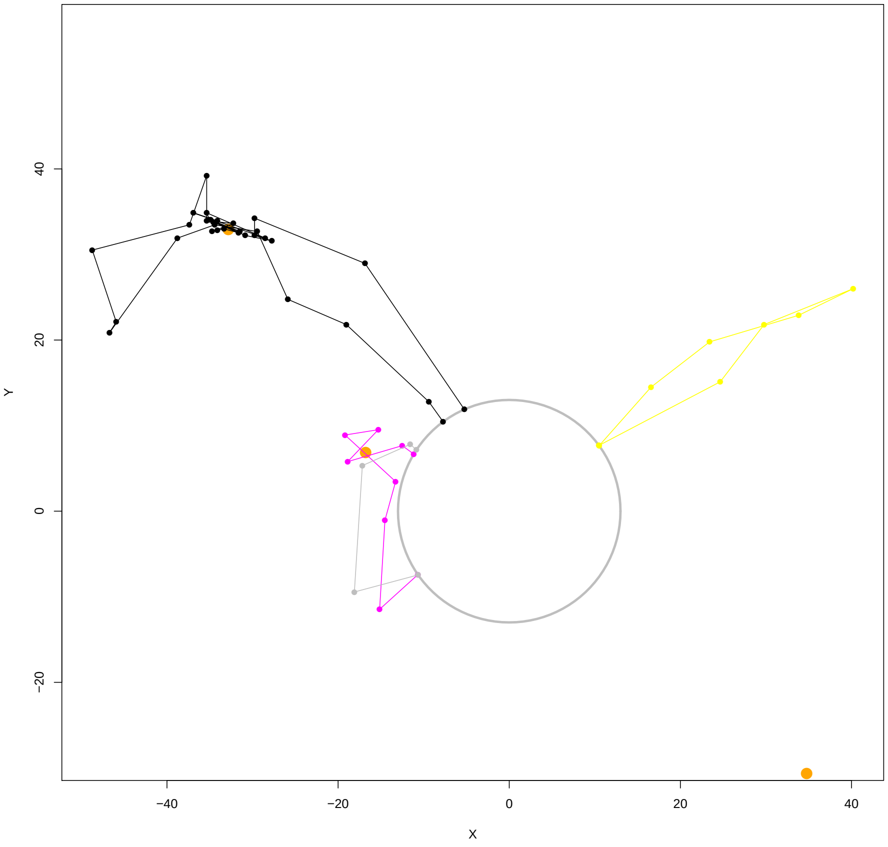

Fig SI-V-2 : Tracks of bee JR-2

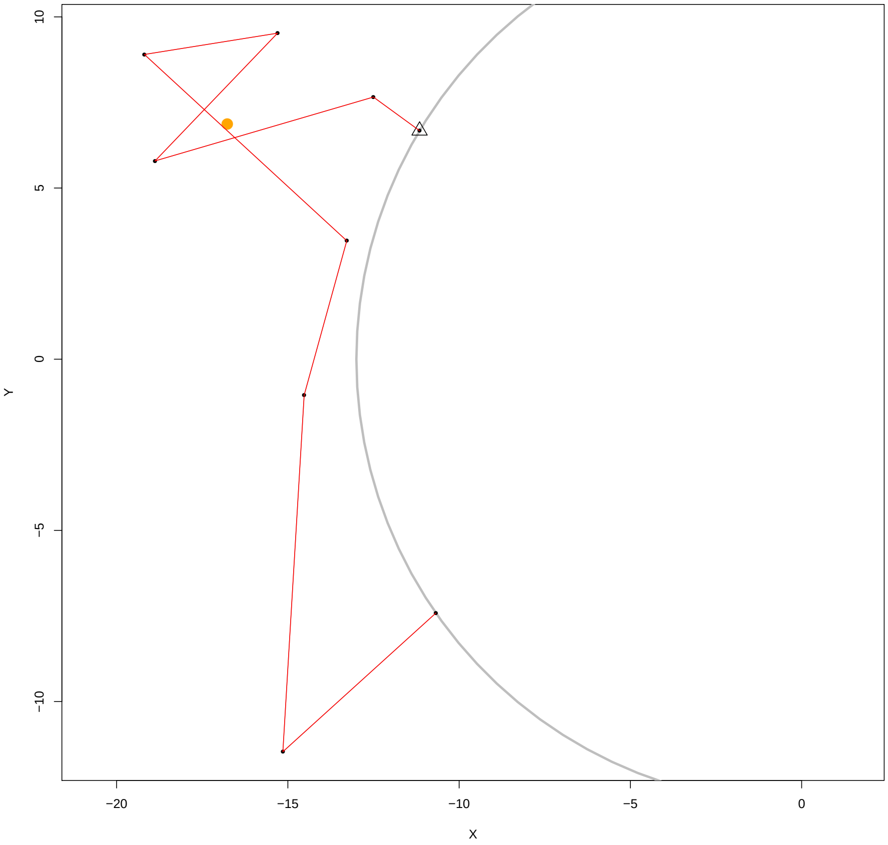

Fig SI-V-2.1 : Loop 6 — loop 1 of bee JR-2

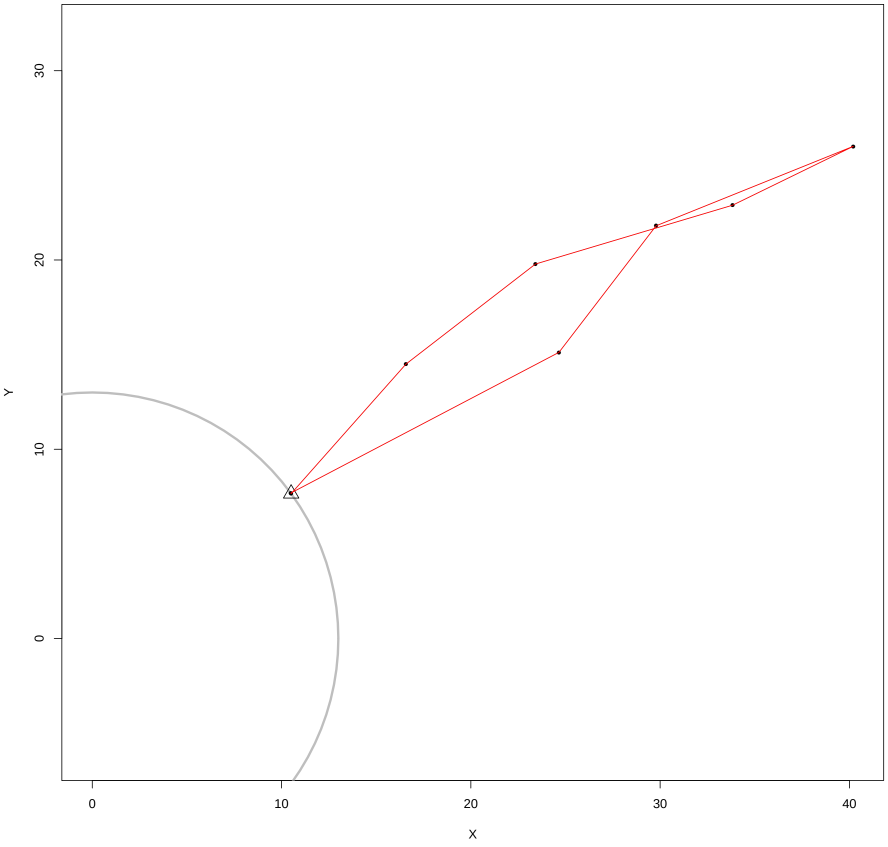

Fig SI-V-2.2 : Loop 7 — loop 2 of bee JR-2

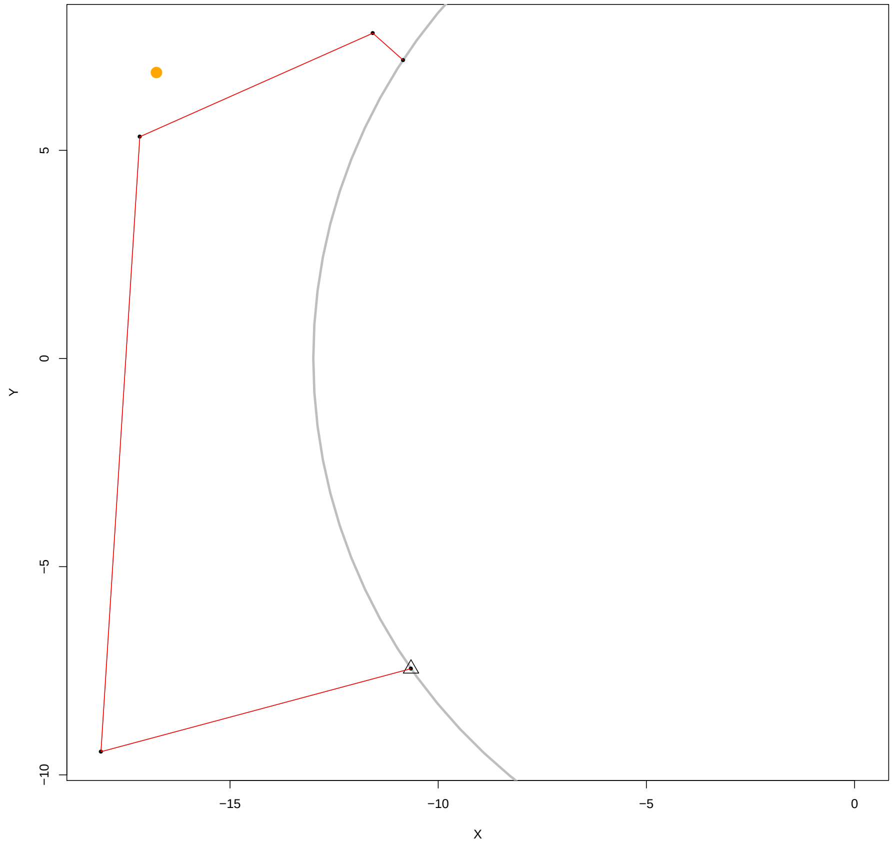

Fig SI-V-2.3 : Loop 8 — loop 3 of bee JR-2

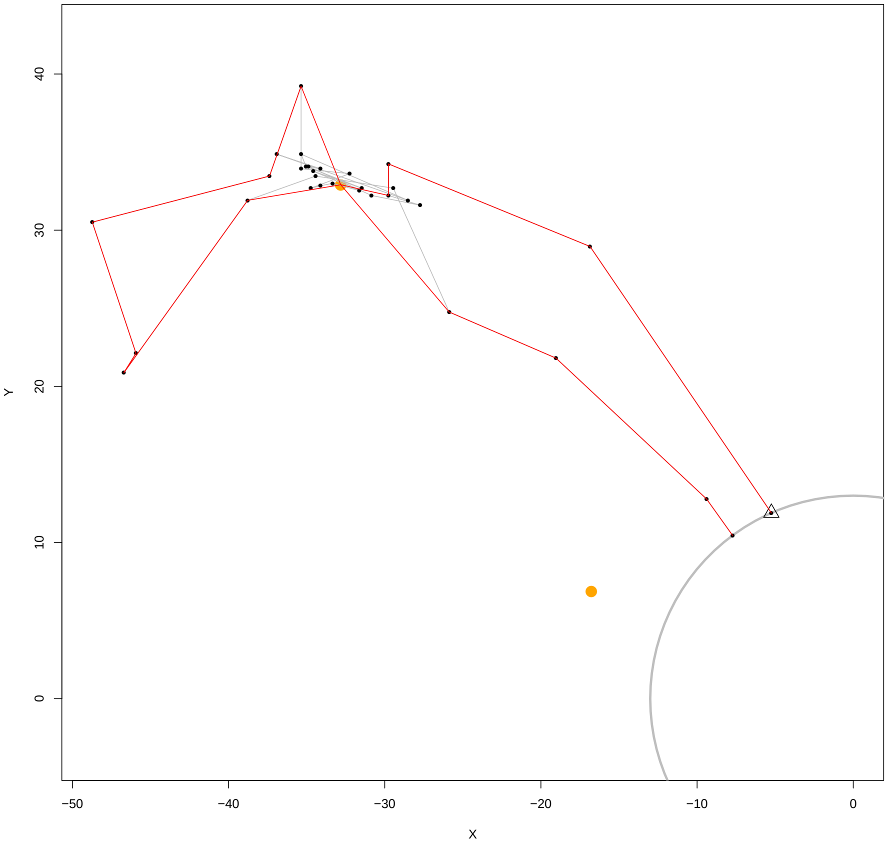

Fig SI-V-2.4 : Loop 9 — loop 4 of bee JR-2

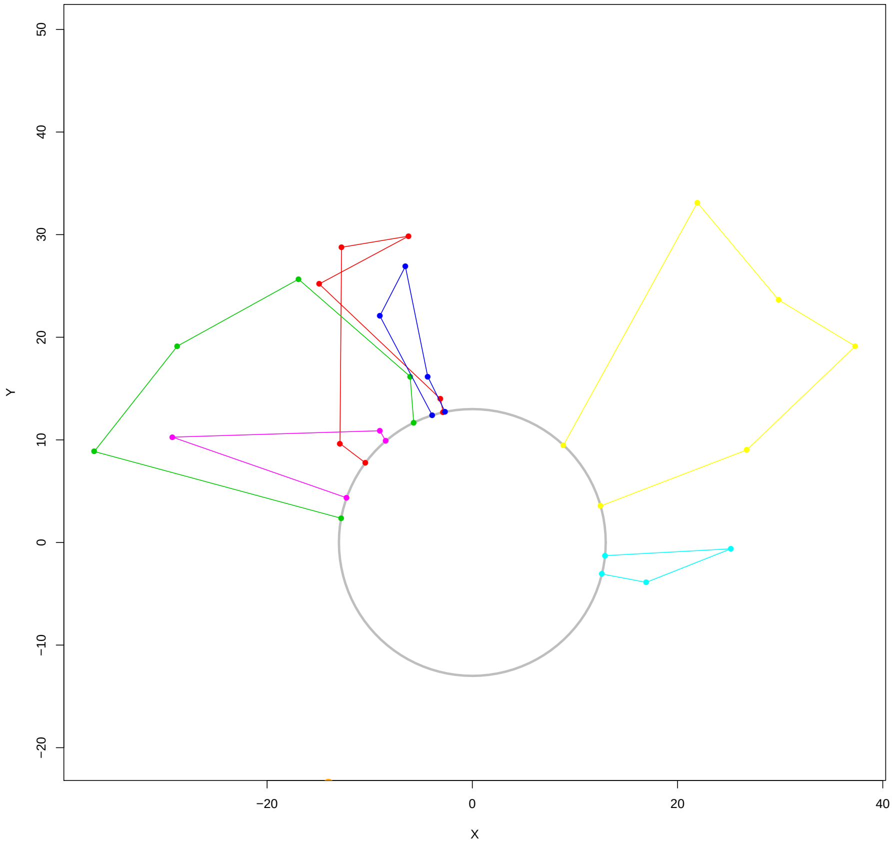

Fig SI-V-3 : Tracks of bee JR-3

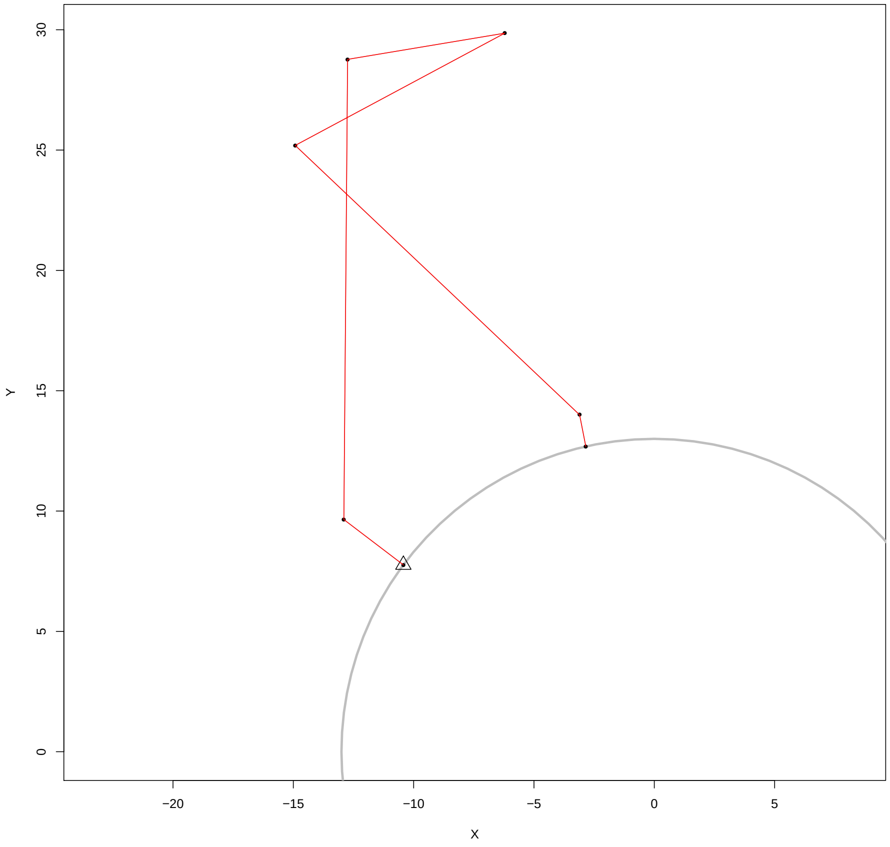

Fig SI-V-3.1 : Loop 10 — loop 1 of bee JR-3

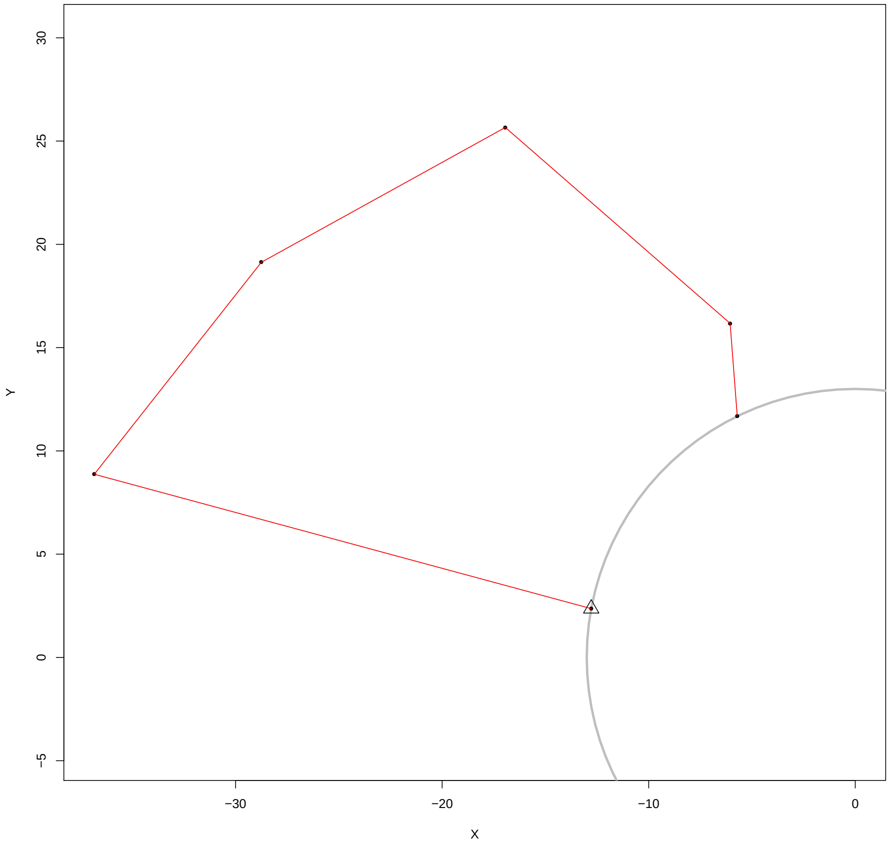

Fig SI-V-3.2 : Loop 11 — loop 2 of bee JR-3

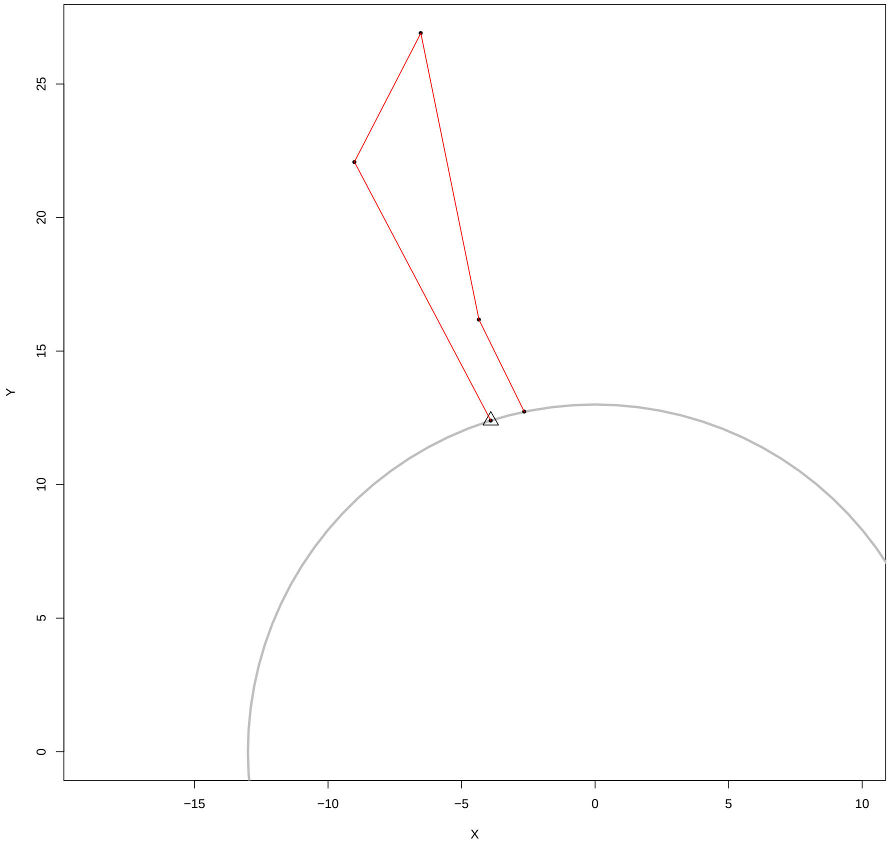

Fig SI-V-3.3 : Loop 12 — loop 3 of bee JR-3

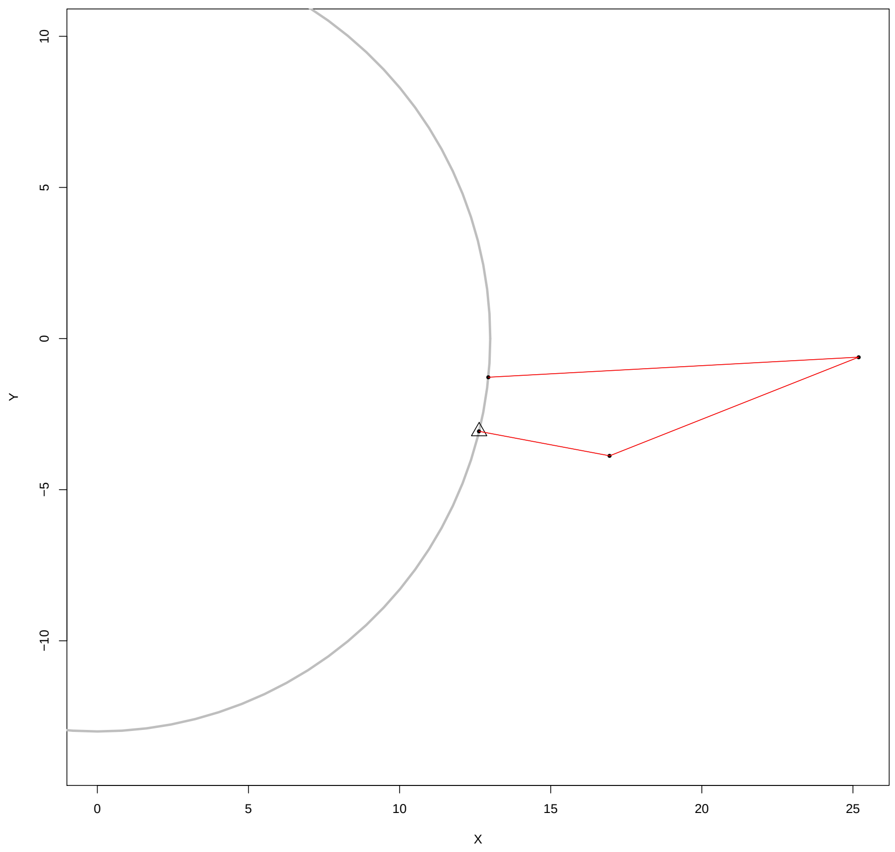

Fig SI-V-3.4 : Loop 13 — loop 4 of bee JR-3

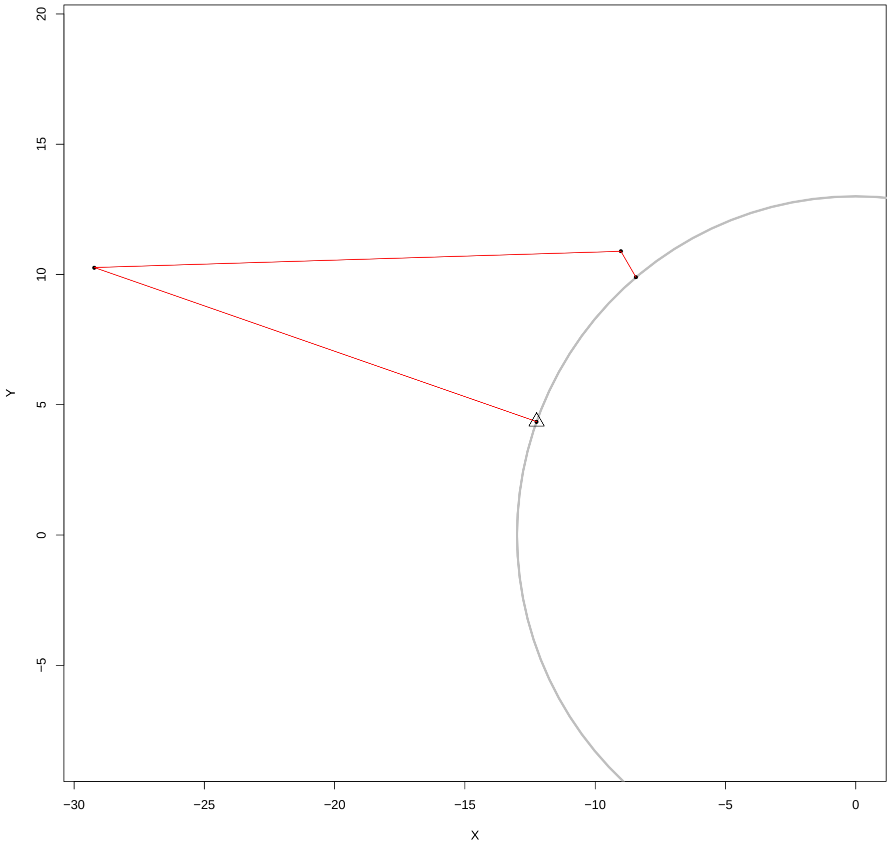

Fig SI-V-3.5 : Loop 14 — loop 5 of bee JR-3

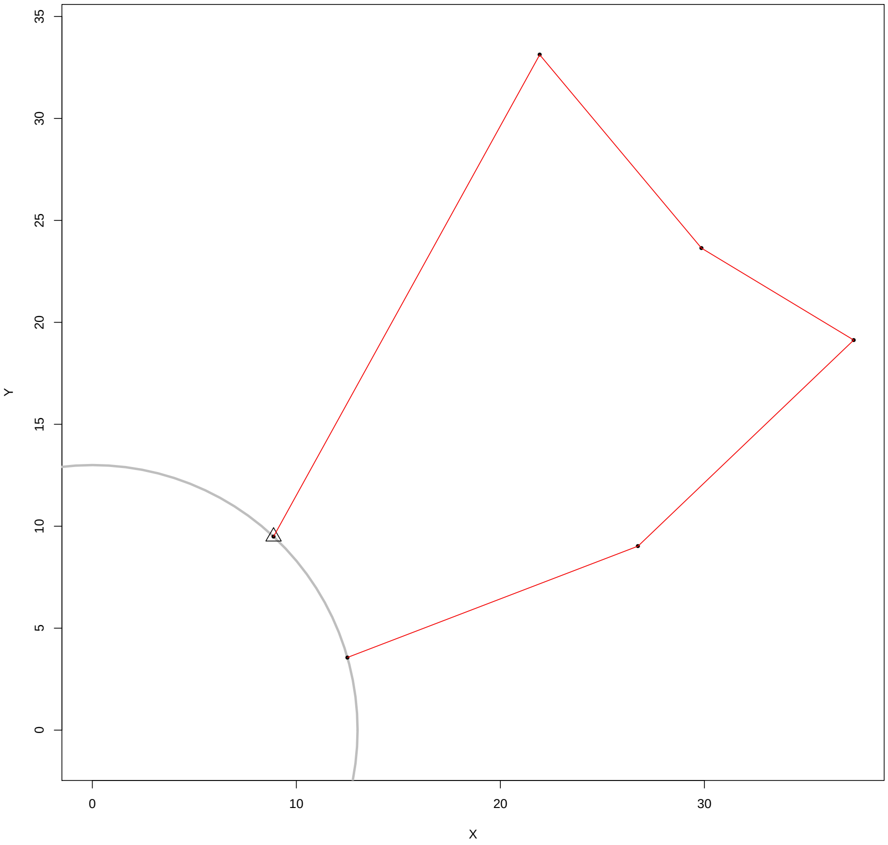

Fig SI-V-3.6 : Loop 15 — loop 6 of bee JR-3

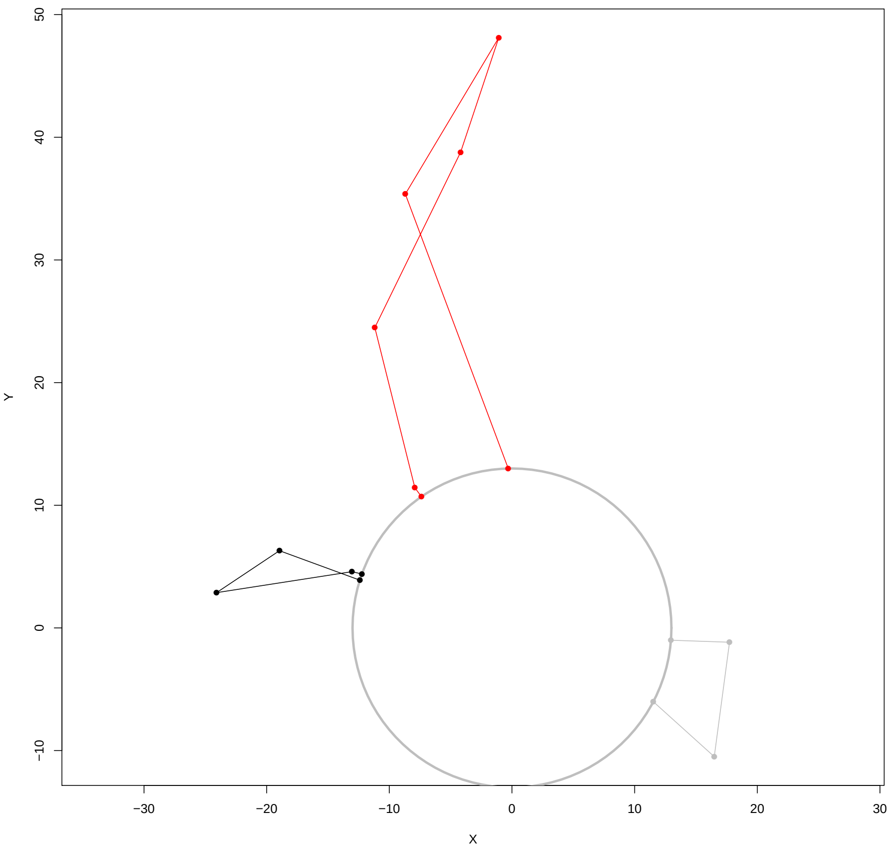

Fig SI-V-4 : Tracks of bee JS-1

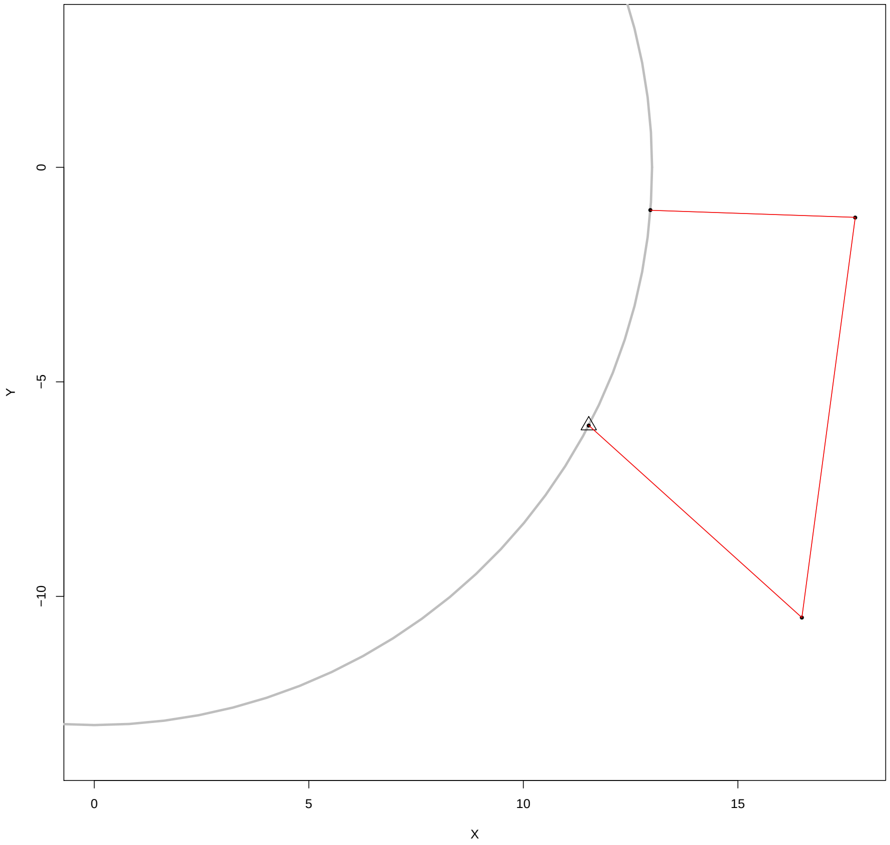

Fig SI-V-4.1 : Loop 16 — loop 1 of bee JS-1

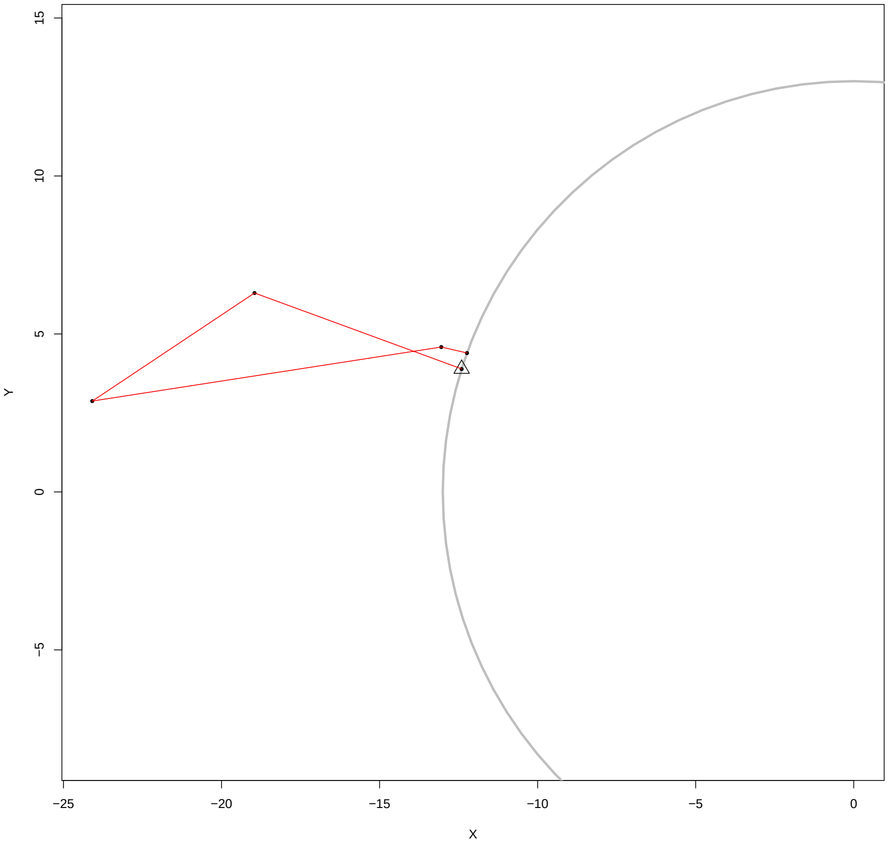

Fig SI-V-4.2 : Loop 17 — loop 2 of bee JS-1

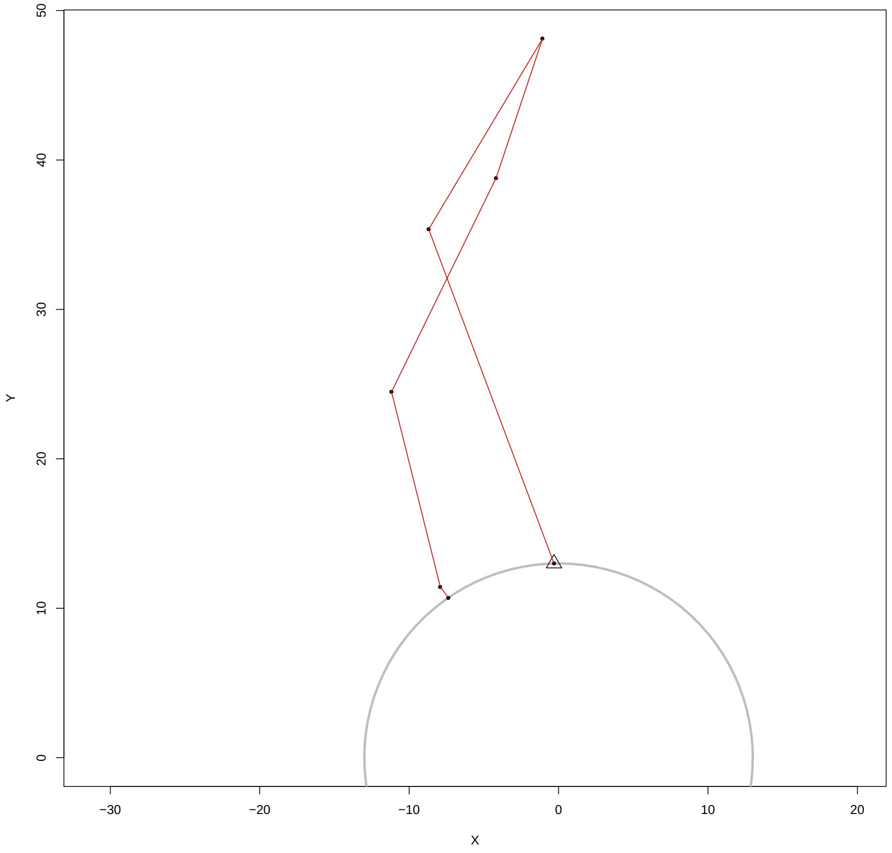

Fig SI-V-4.3 : Loop 18 — loop 3 of bee JS-1

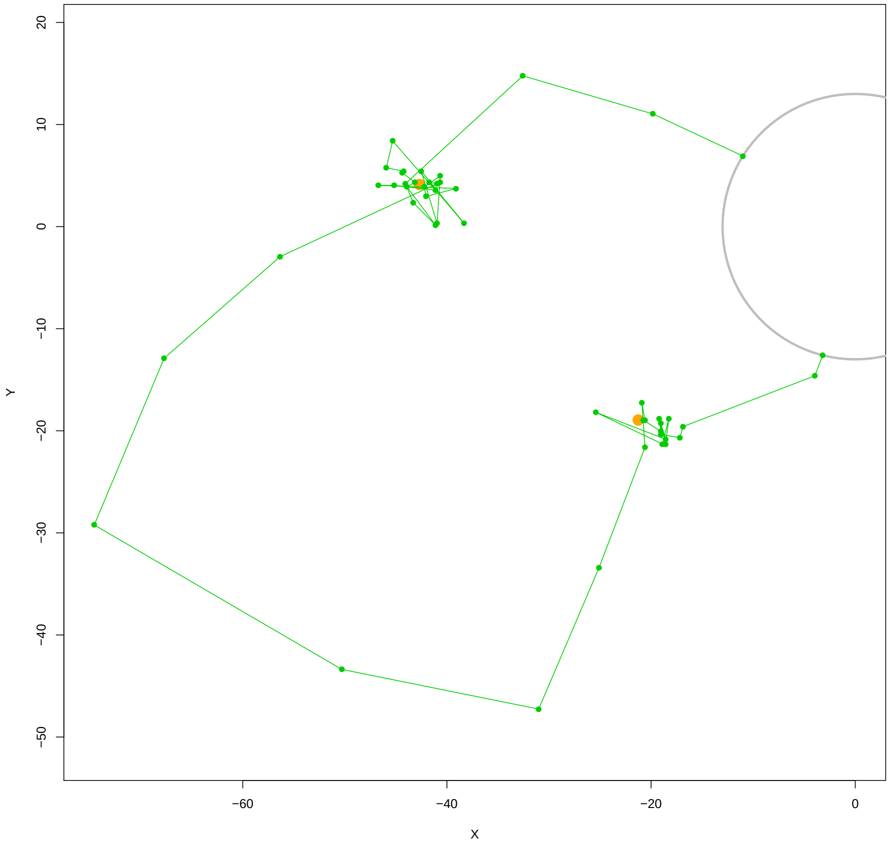

Fig SI-V-5 : Tracks of bee JS-2

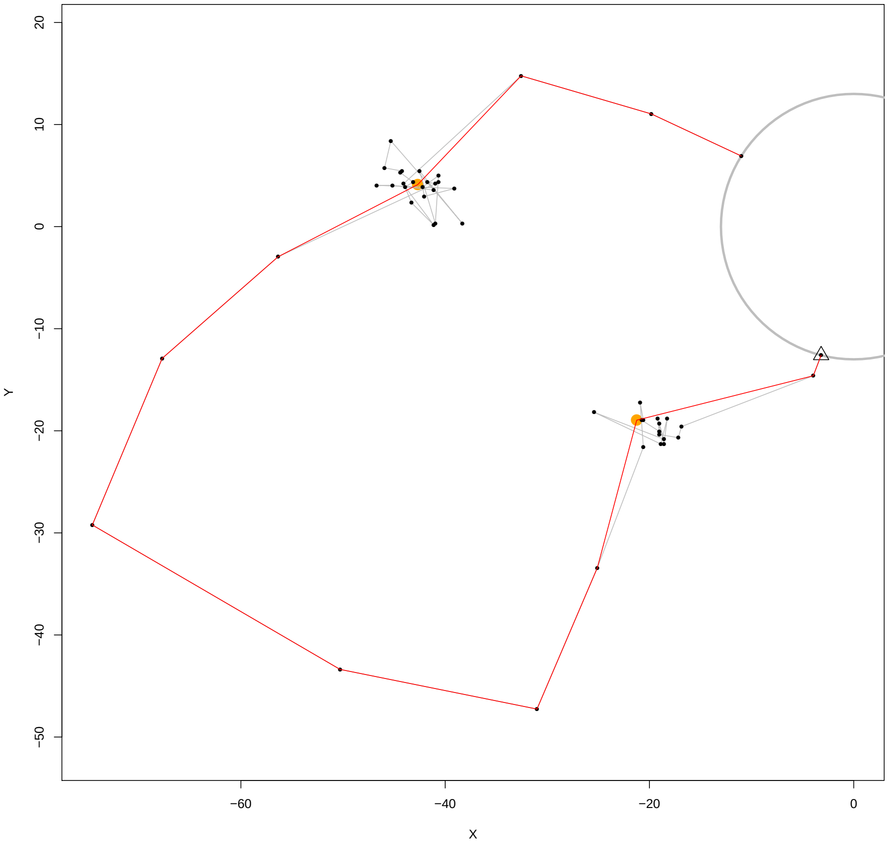

Fig SI-V-5.1 : Loop 19 — loop 1 of bee JS-2

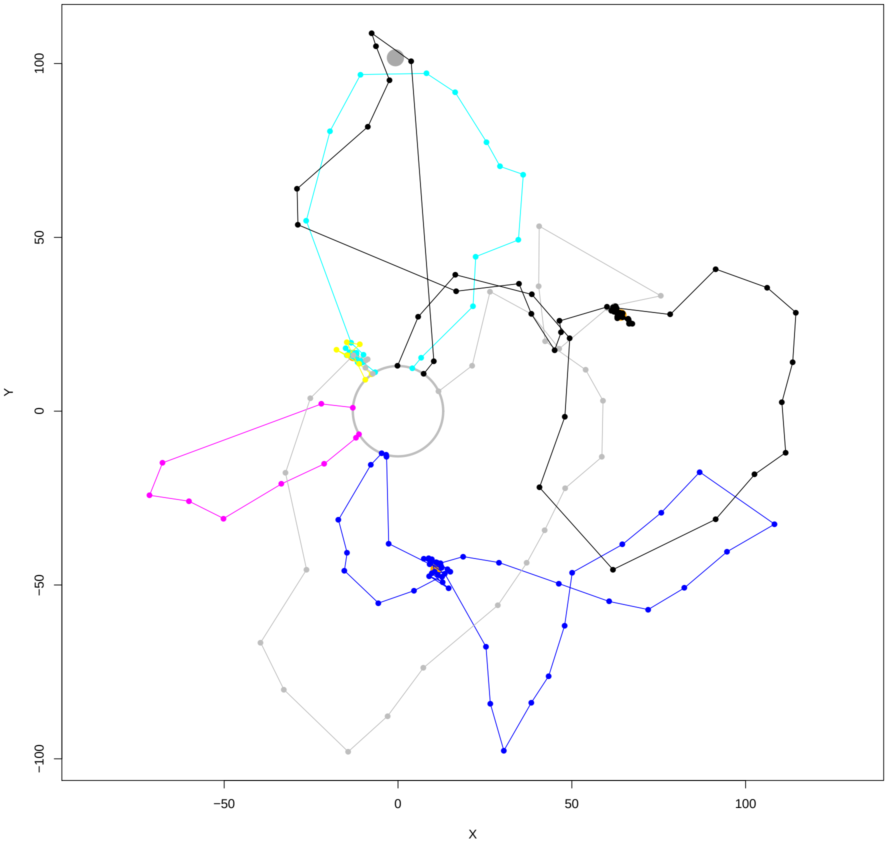

Fig SI-V-6 : Tracks of bee JS-3

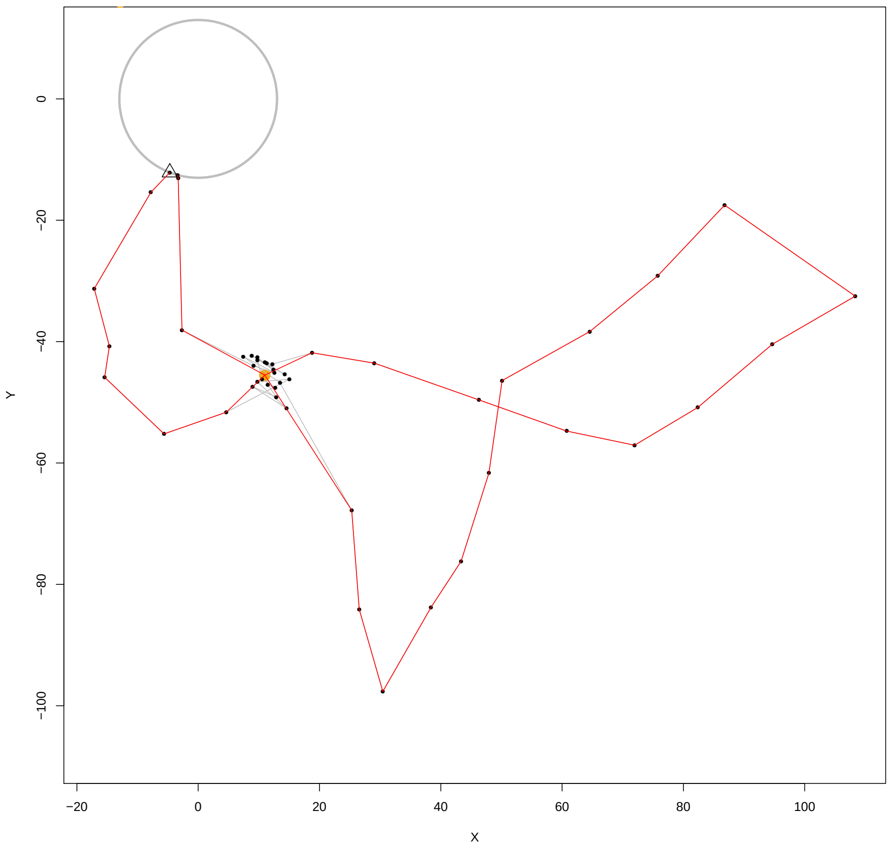

Fig SI-V-6.1 : Loop 20 — loop 1 of bee JS-3

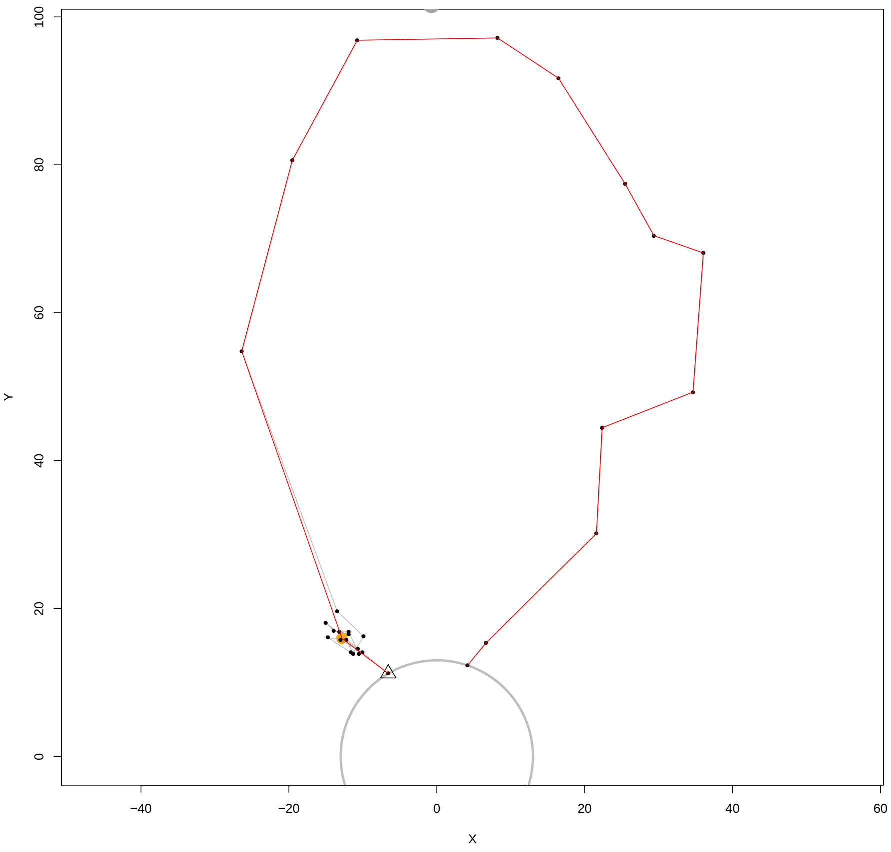

Fig SI-V-6.2 : Loop 21 — loop 2 of bee JS-3

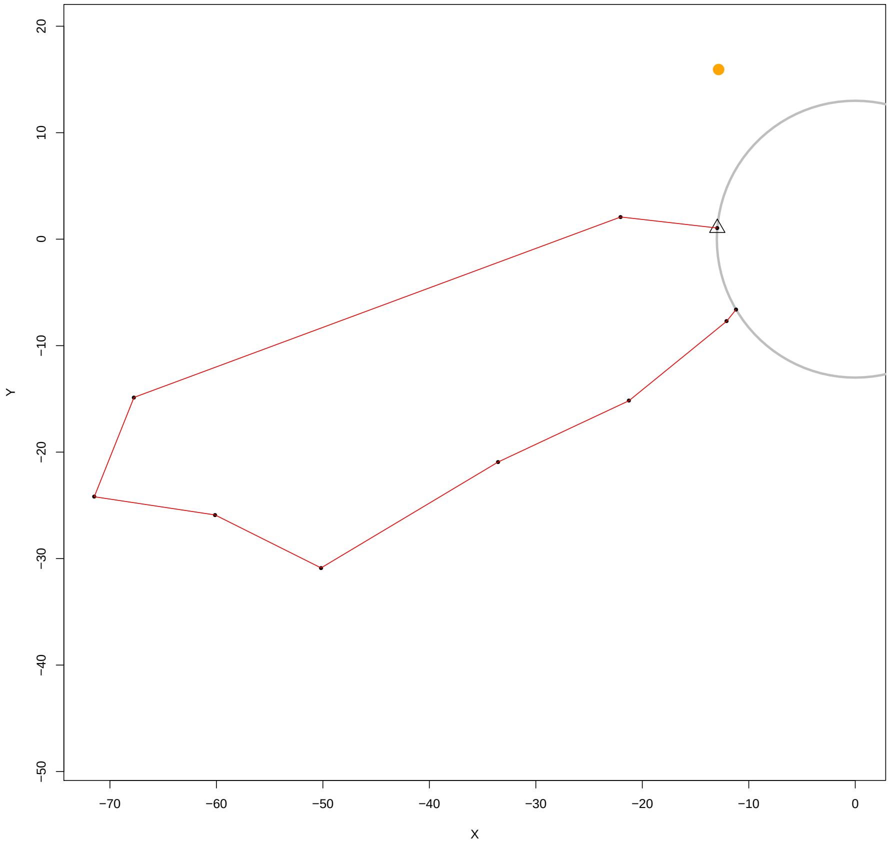

Fig SI-V-6.3 : Loop 22 — loop 3 of bee JS-3

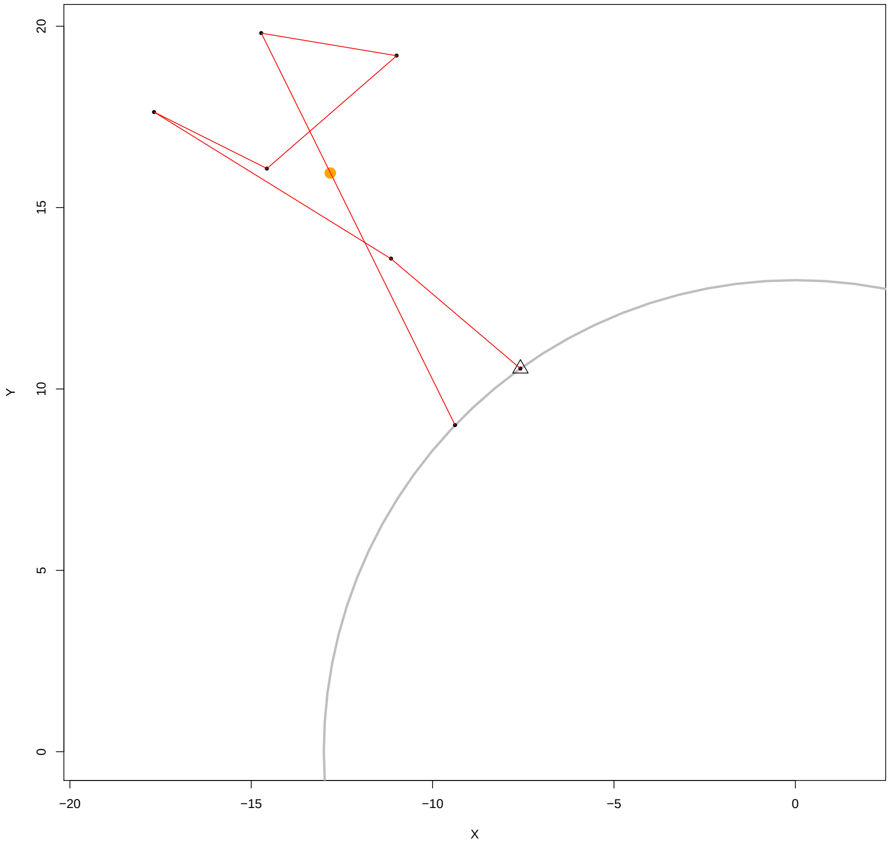

Fig SI-V-6.4 : Loop 23 — loop 4 of bee JS-3

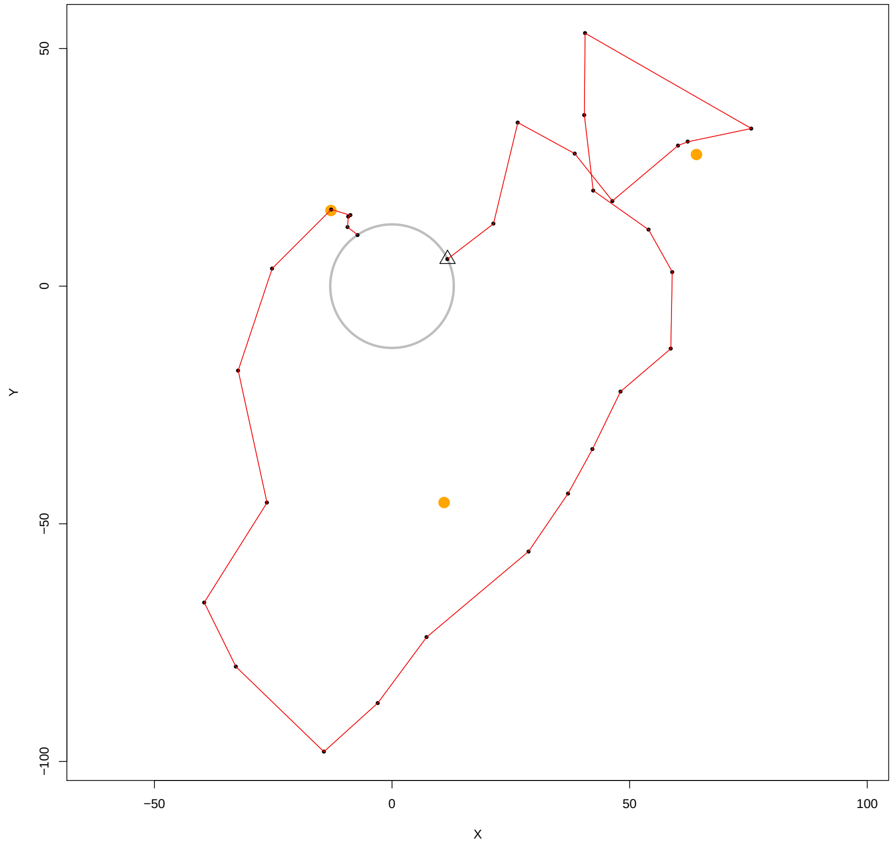

Fig SI-V-6.5 : Loop 24 — loop 5 of bee JS-3

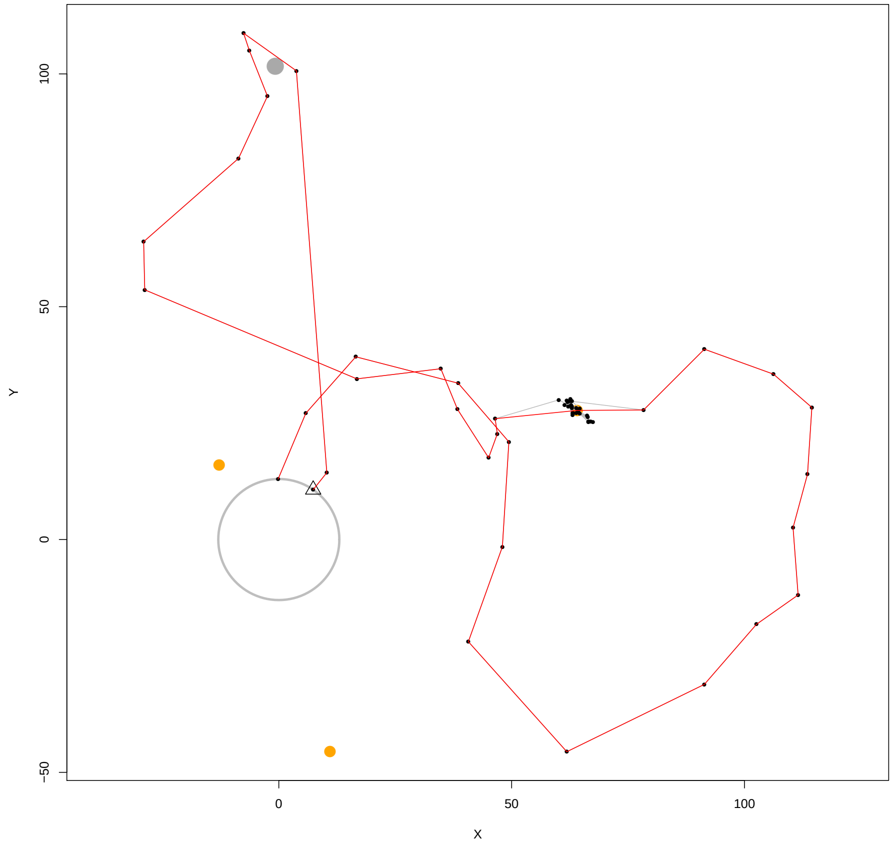

Fig SI-V-6.6 : Loop 25 — loop 6 of bee JS-3

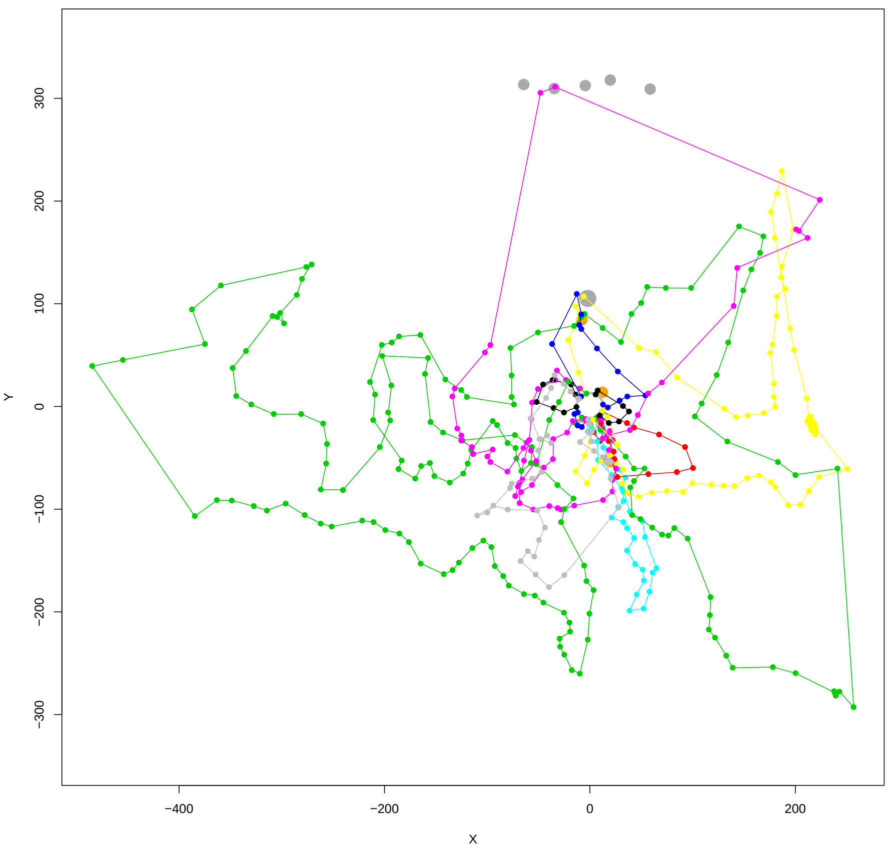

Fig SI-V-7 : Tracks of bee GH-1

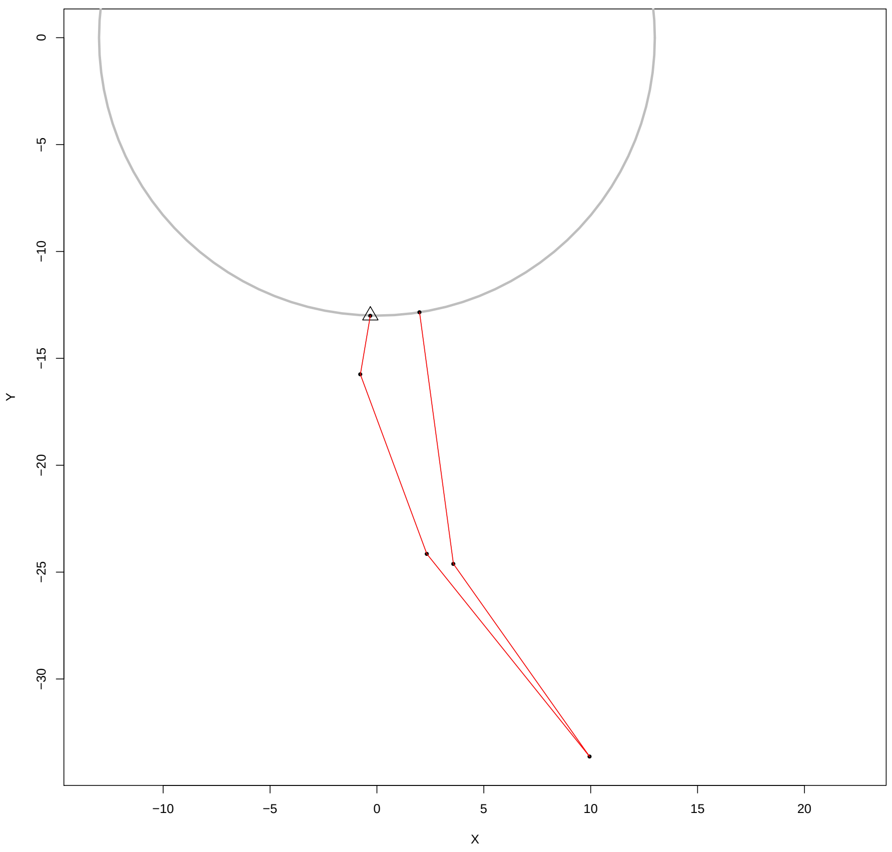

Fig SI-V-7.1 : Loop 26 — loop 1 of bee GH-1

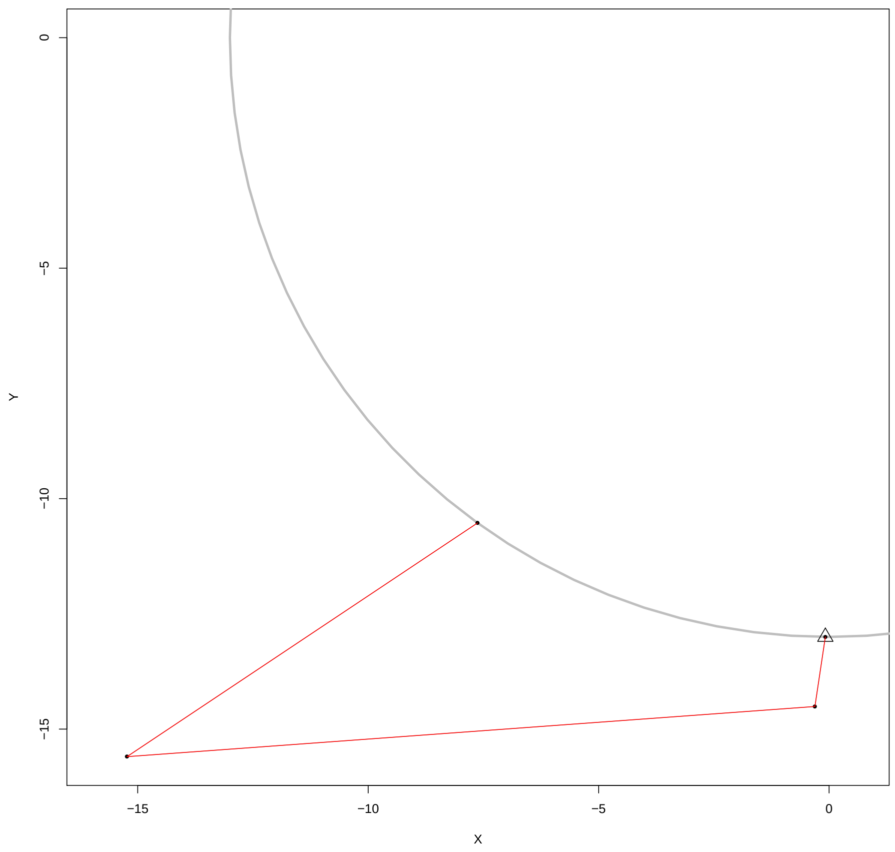

Fig SI-V-7.2 : Loop 27 — loop 2 of bee GH-1

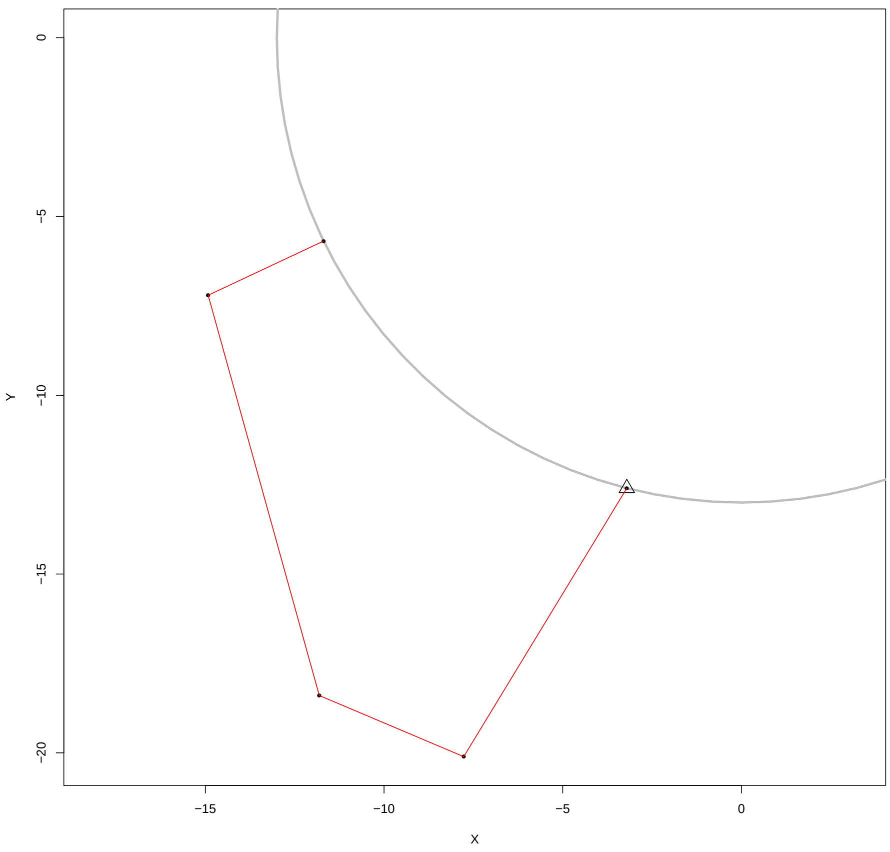

Fig SI-V-7.3 : Loop 28 — loop 3 of bee GH-1

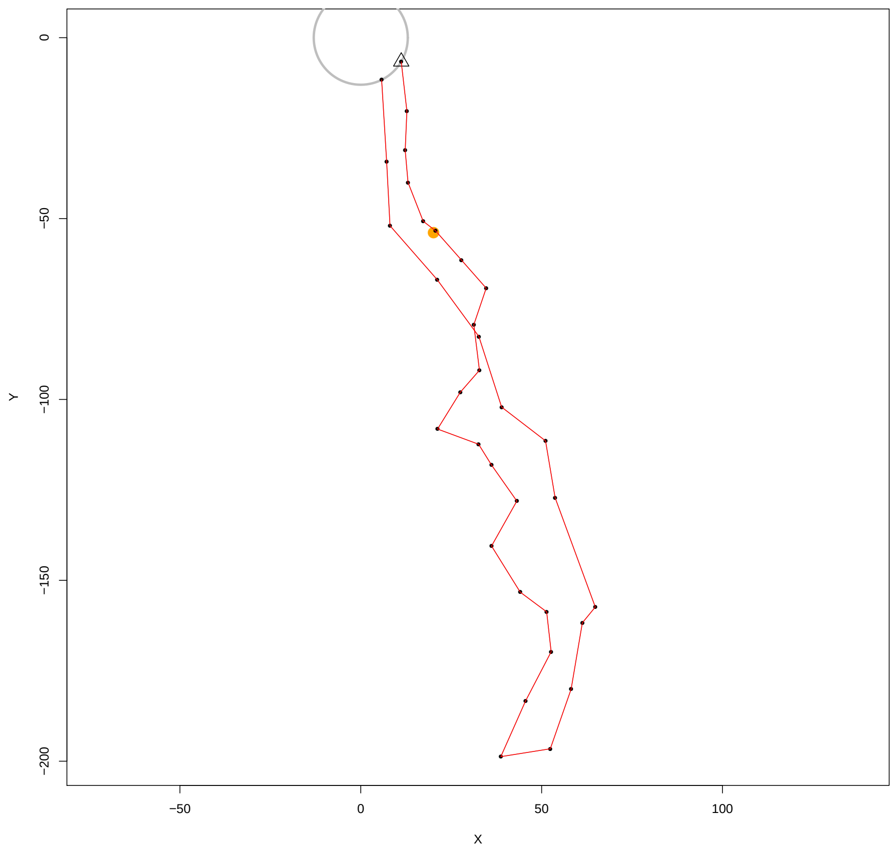

Fig SI-V-7.4 : Loop 29 — loop 4 of bee GH-1

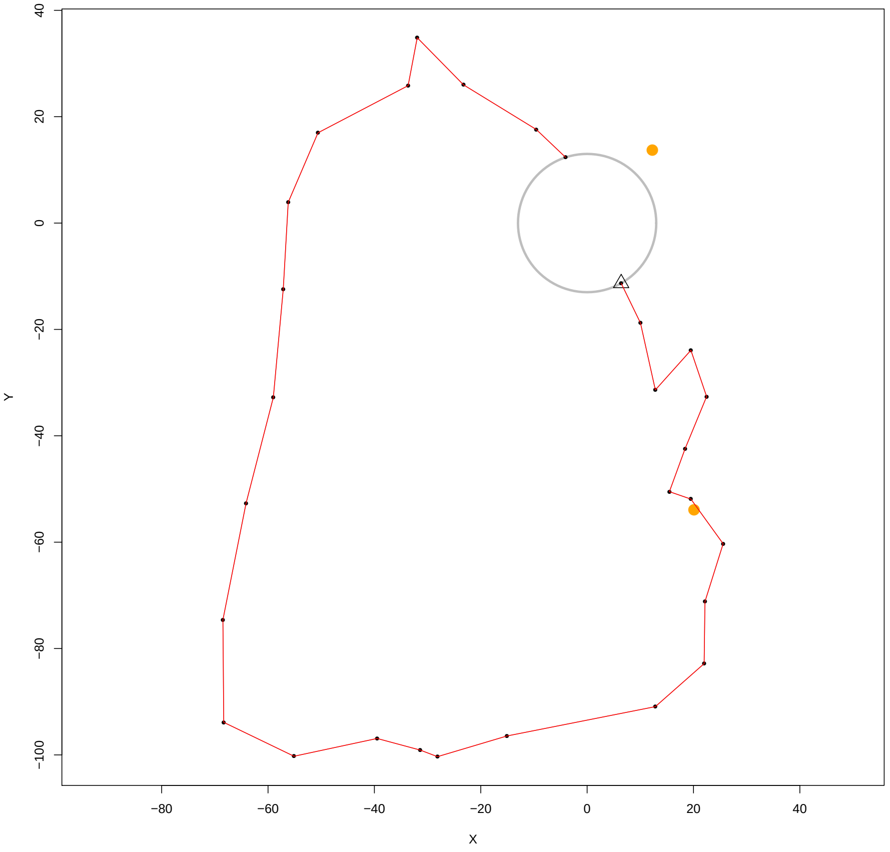

Fig SI-V-7.5 : Loop 30 — loop 5 of bee GH-1

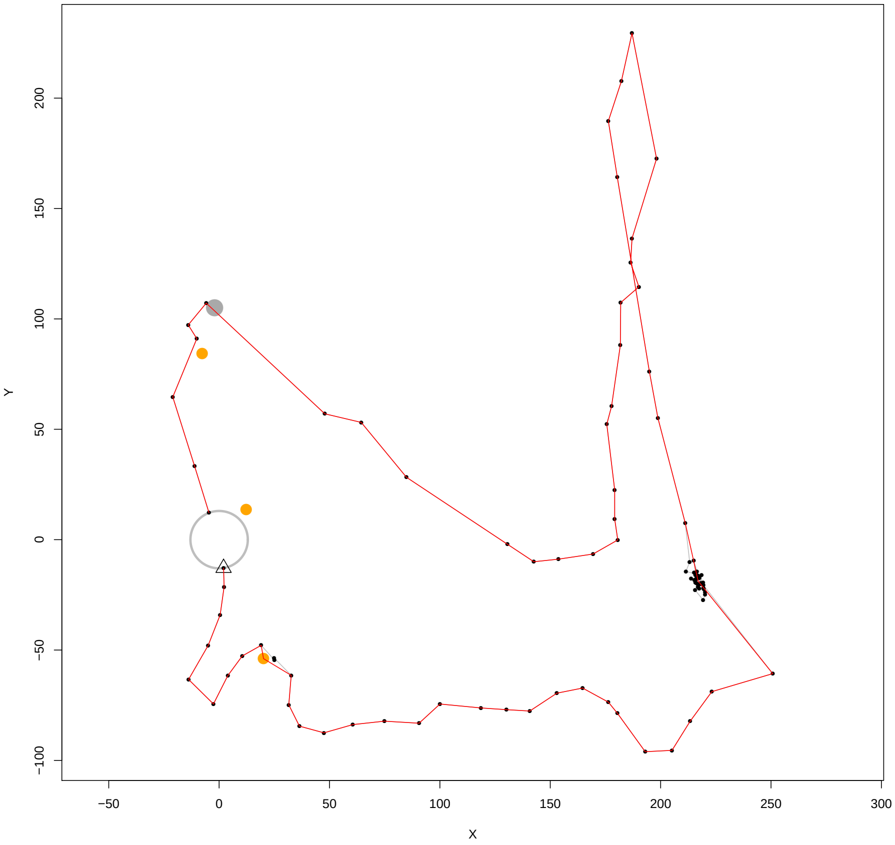

Fig SI-V-7.6 : Loop 31 — loop 6 of bee GH-1

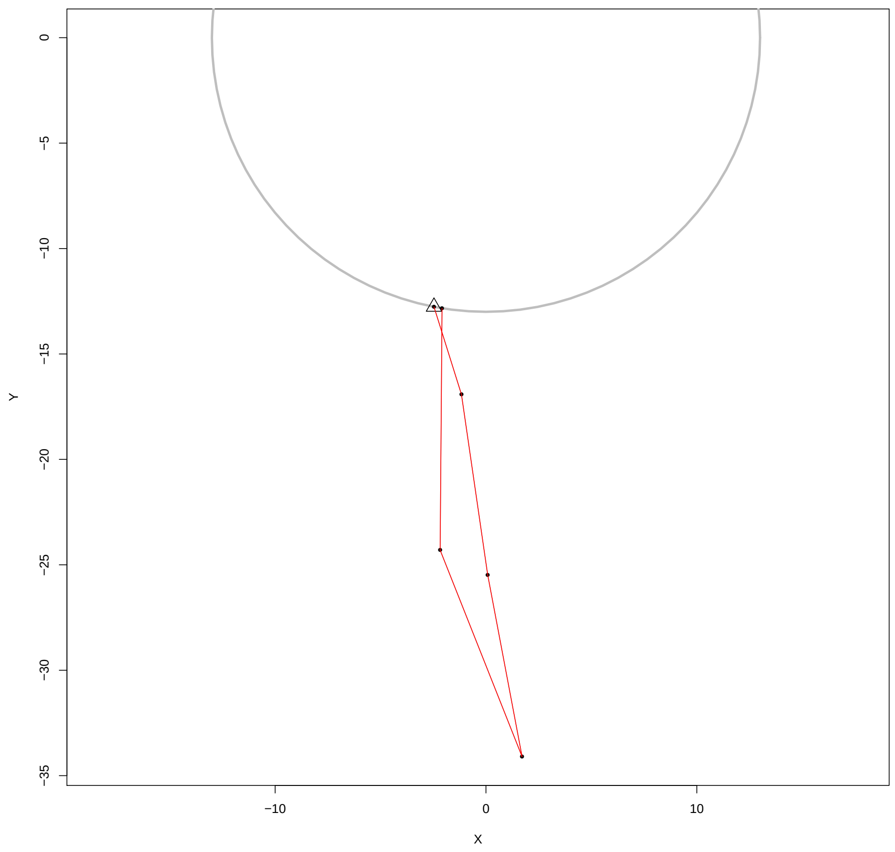

Fig SI-V-7.7 : Loop 32 — loop 7 of bee GH-1

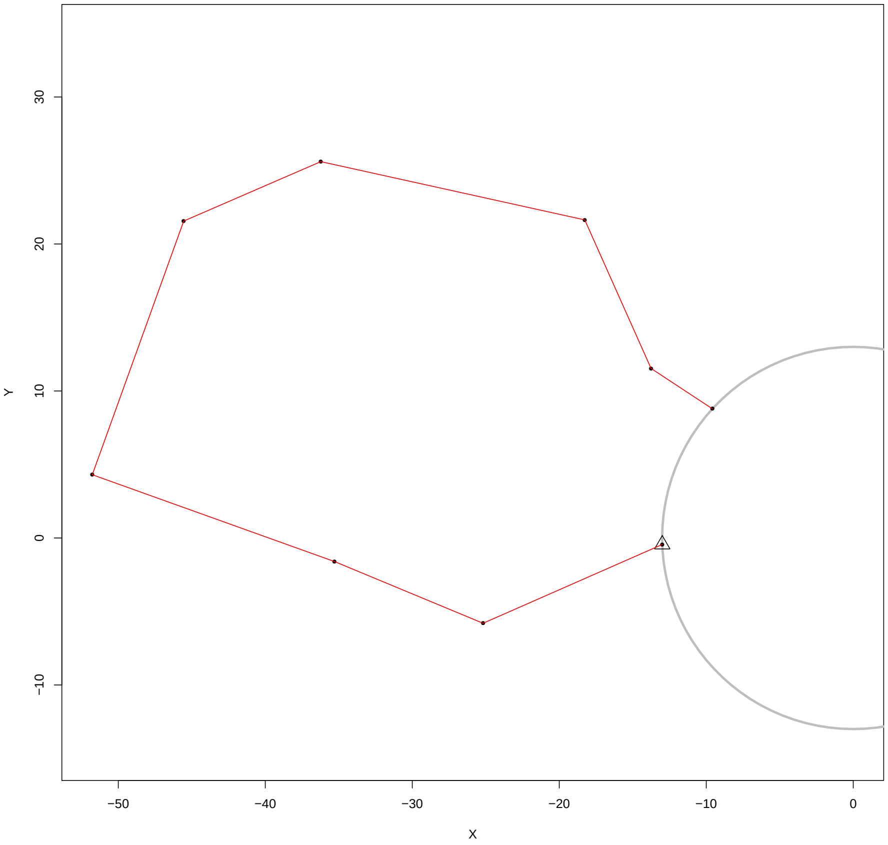

Fig SI-V-7.8 : Loop 33 — loop 8 of bee GH-1

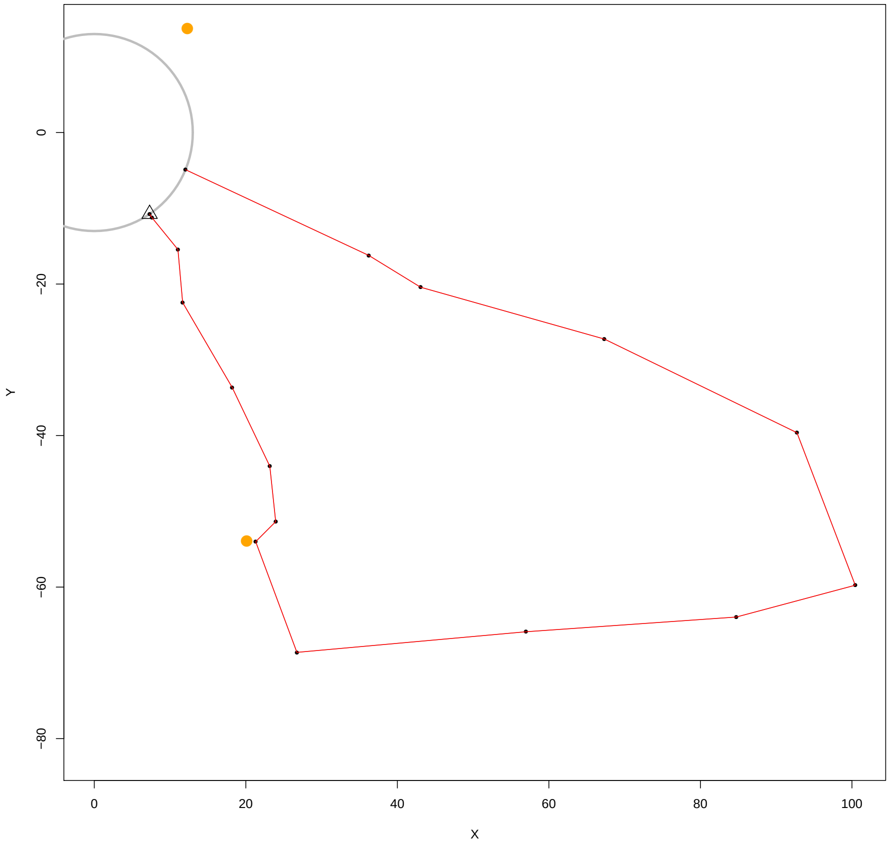

Fig SI-V-7.9 : Loop 34 — loop 9 of bee GH-1

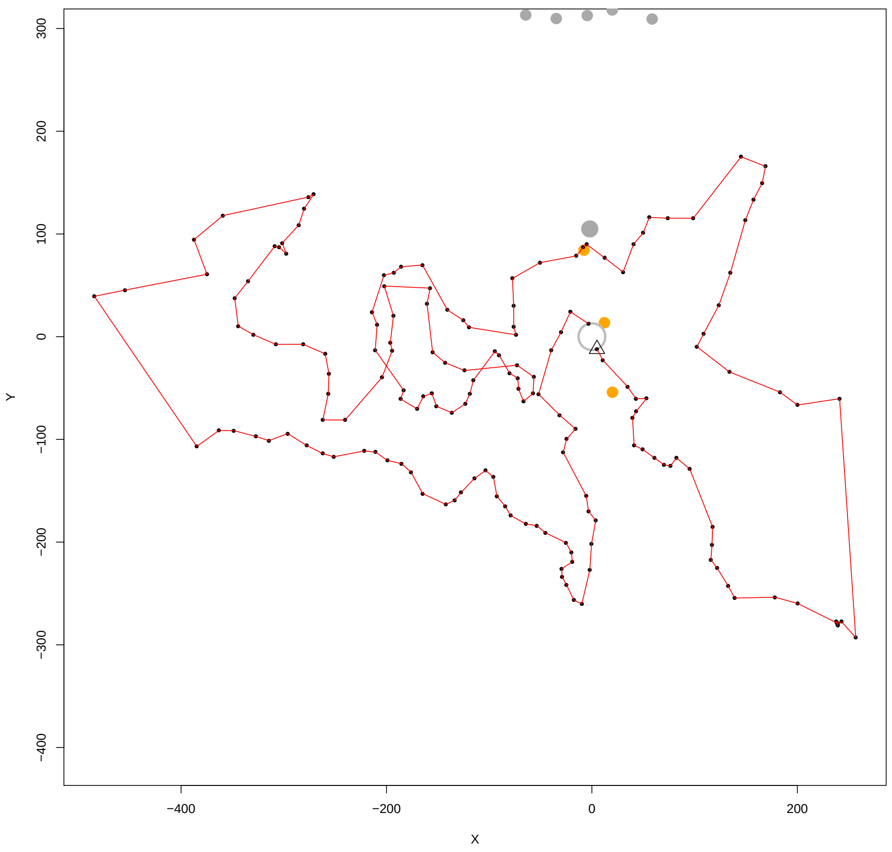

Fig SI-V-7.10 : Loop 35 — loop 10 of bee GH-1

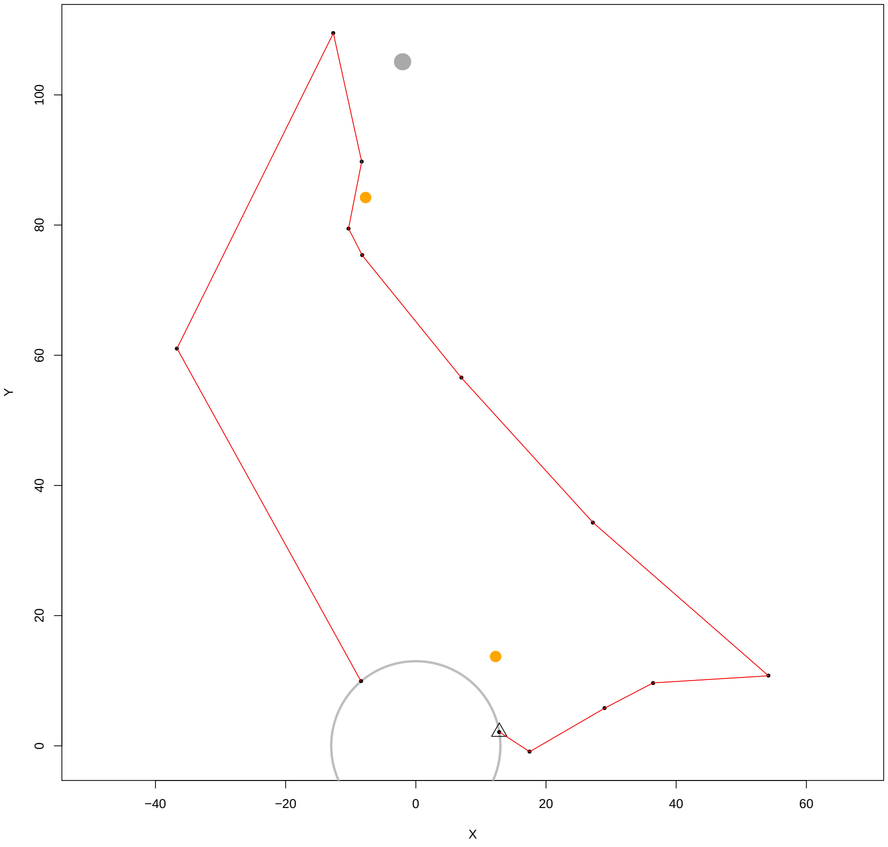

Fig SI-V-7.11 : Loop 36 — loop 11 of bee GH-1

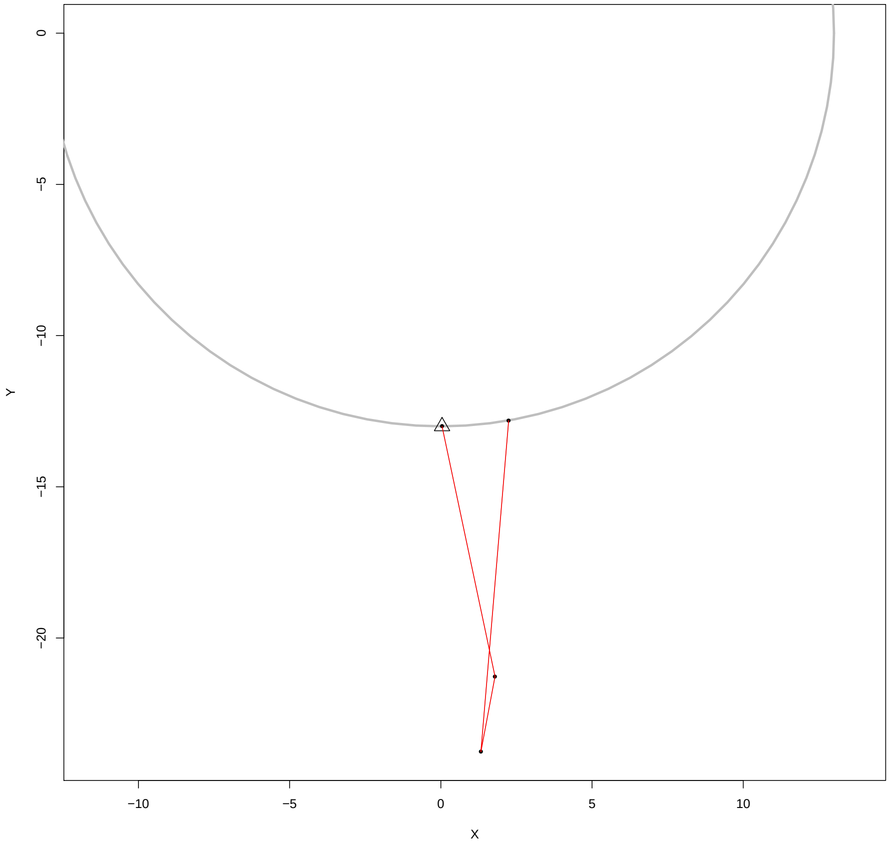

Fig SI-V-7.12 : Loop 37 — loop 12 of bee GH-1

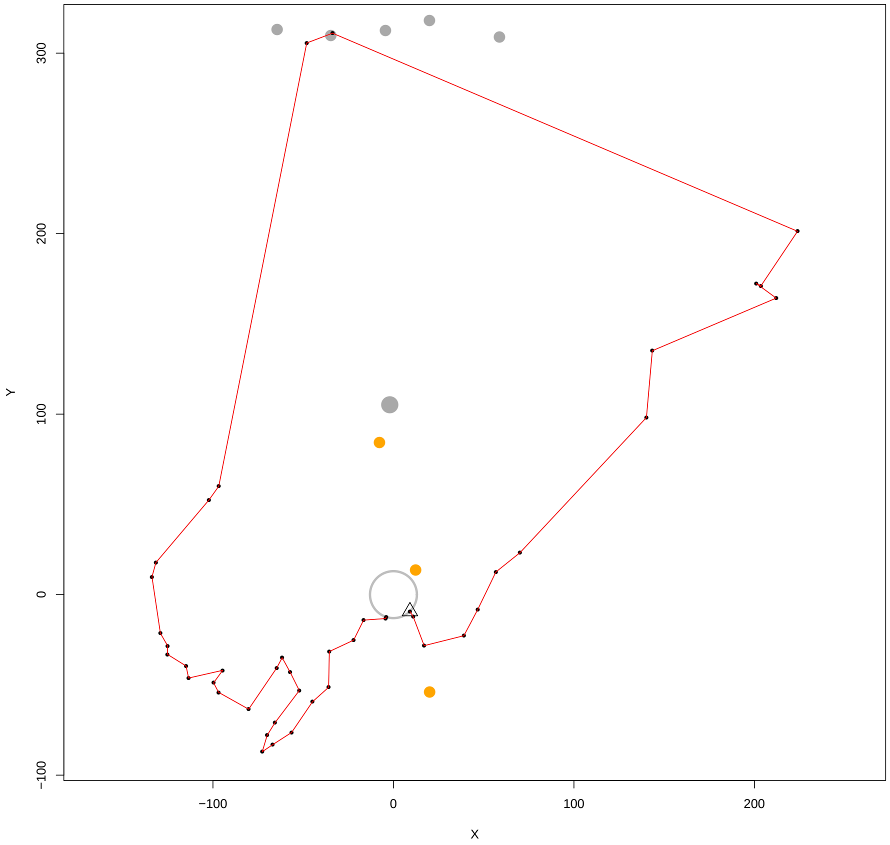

Fig SI-V-7.13 : Loop 38 — loop 13 of bee GH-1

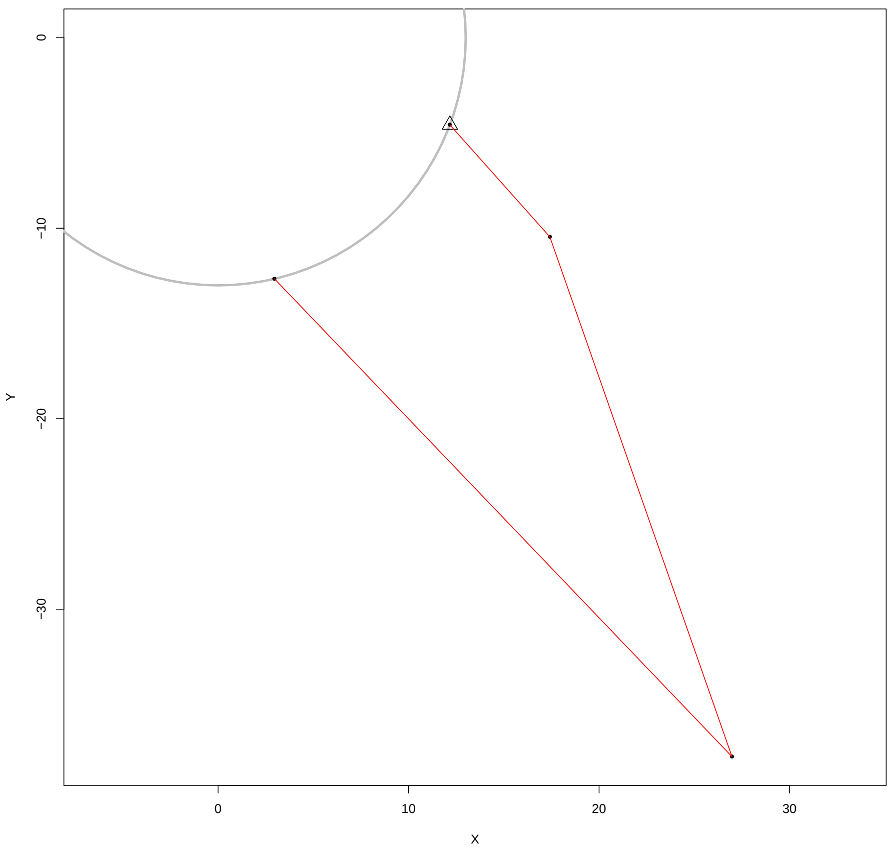

Fig SI-V-7.14 : Loop 39 — loop 14 of bee GH-1

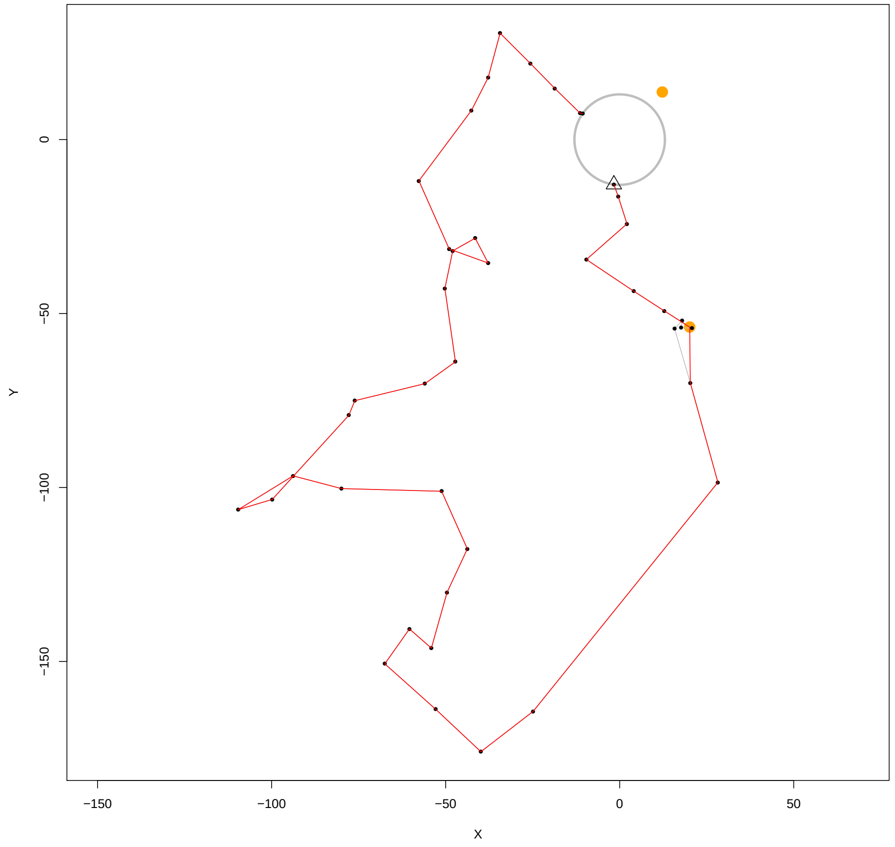

Fig SI-V-7.15 : Loop 40 — loop 15 of bee GH-1

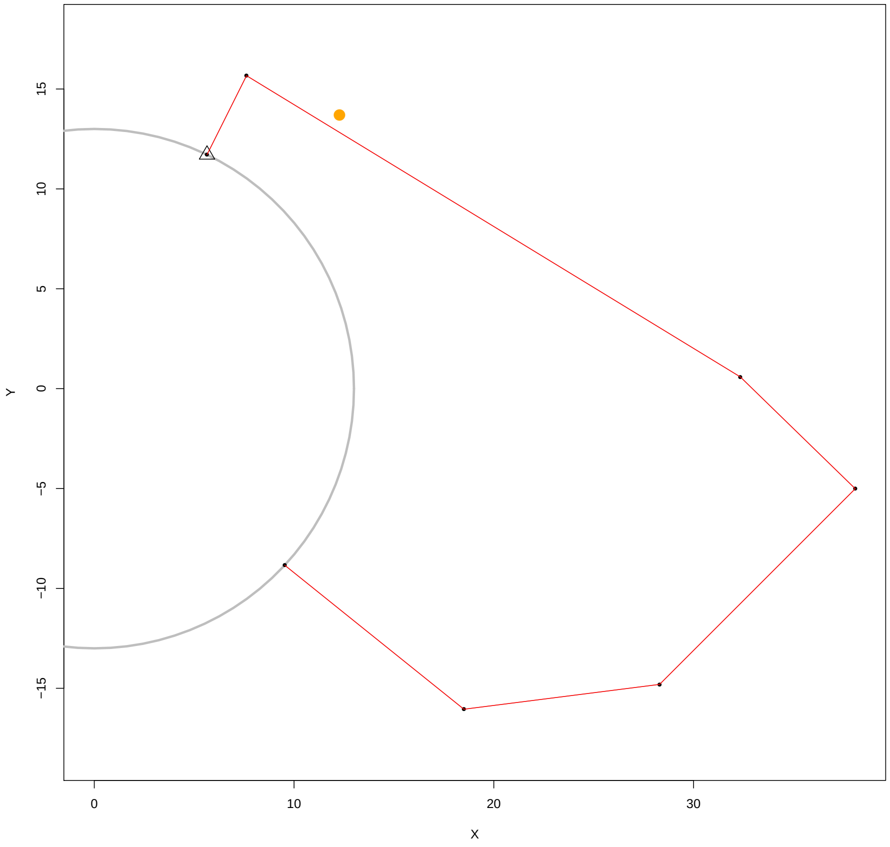

Fig SI-V-7.16 : Loop 41 — loop 16 of bee GH-1

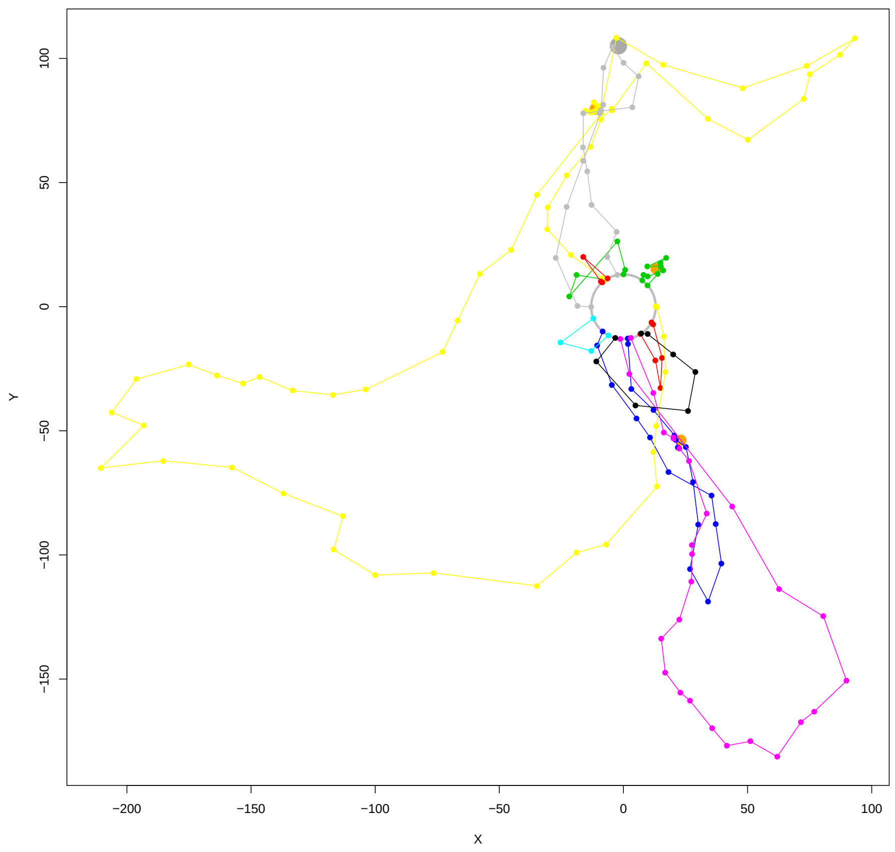

Fig SI-V-8 : Tracks of bee GE-1

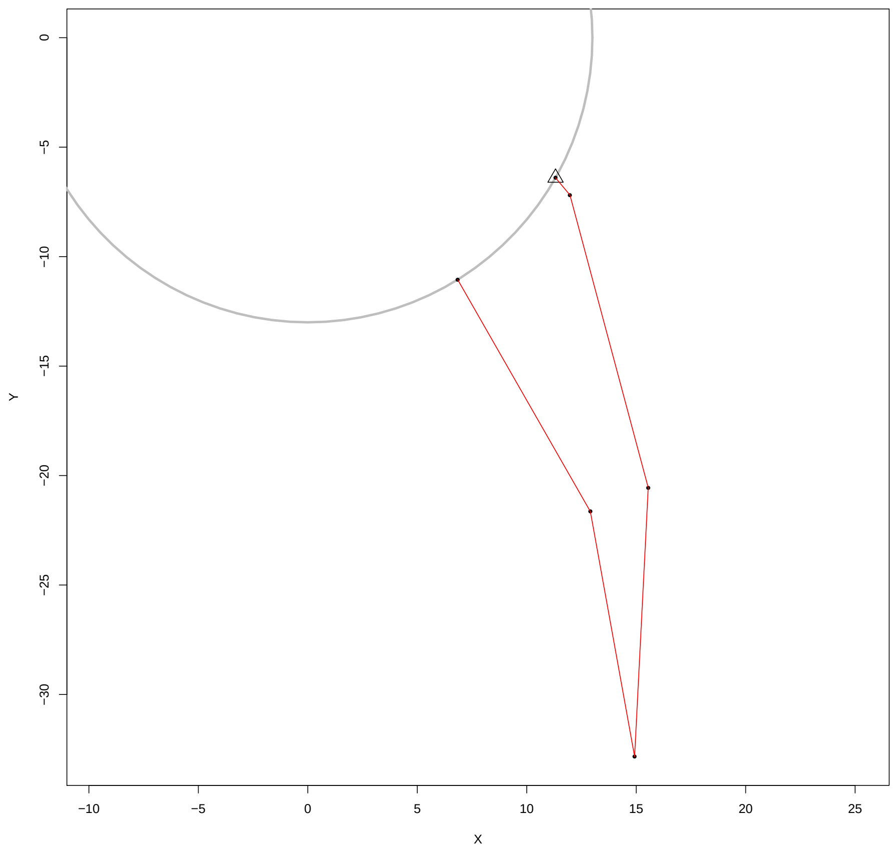

Fig SI-V-8.1 : Loop 42 — loop 1 of bee GE-1

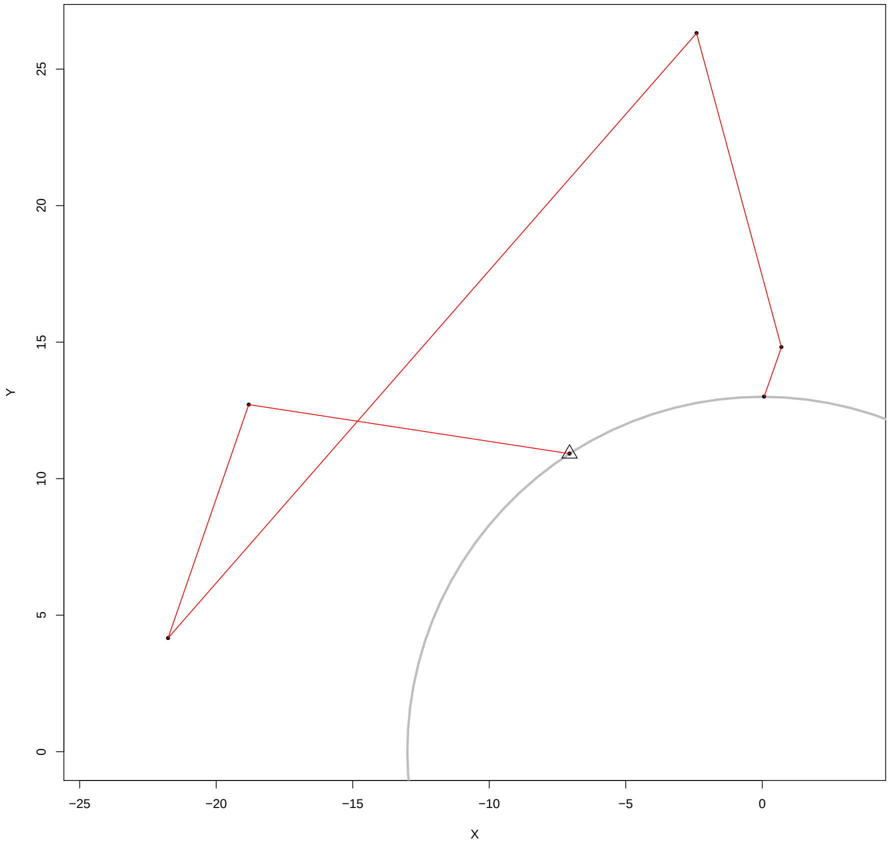

Fig SI-V-8.2 : Loop 43 — loop 2 of bee GE-1

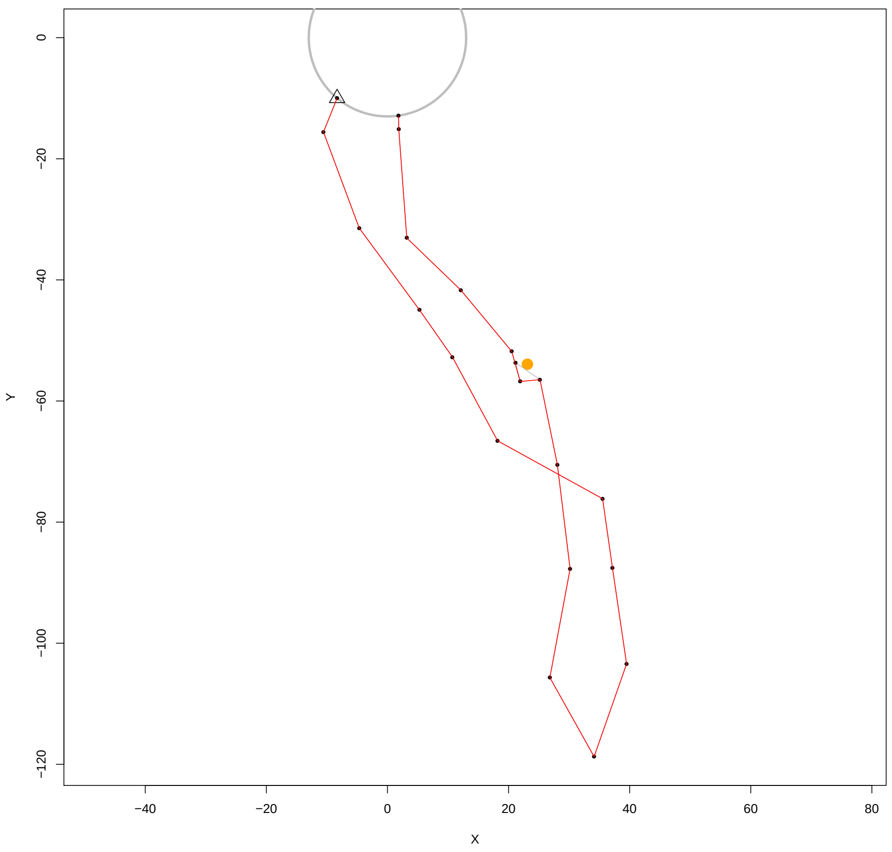

Fig SI-V-8.3 : Loop 44 — loop 3 of bee GE-1

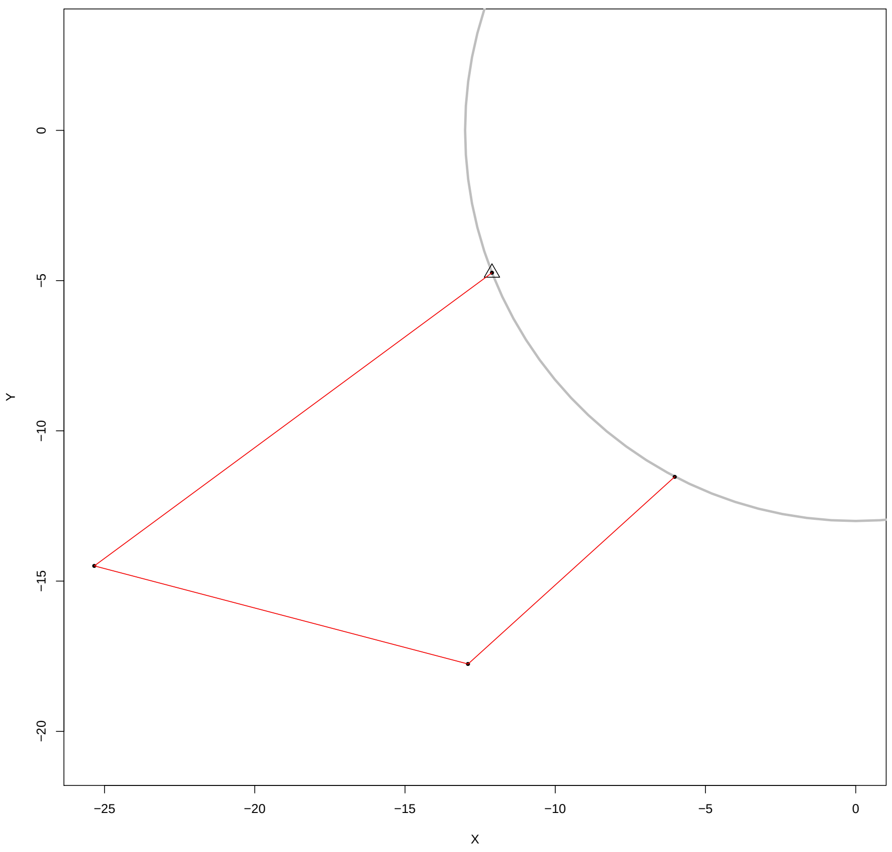

Fig SI-V-8.4 : Loop 45 — loop 4 of bee GE-1

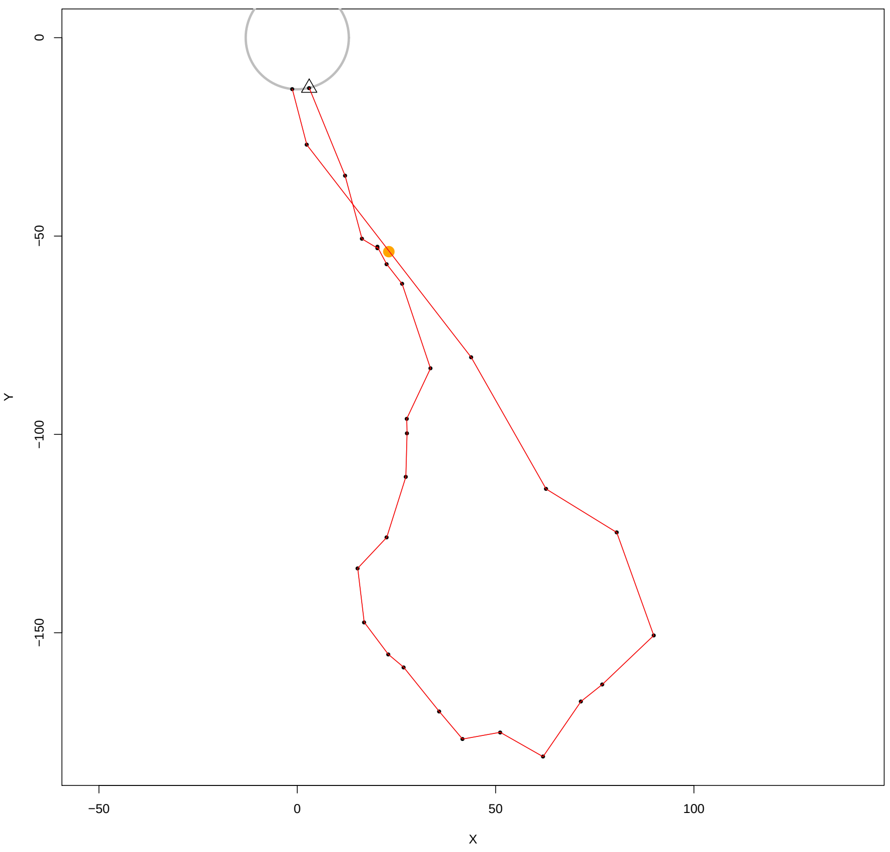

Fig SI-V-8.5 : Loop 46 — loop 5 of bee GE-1

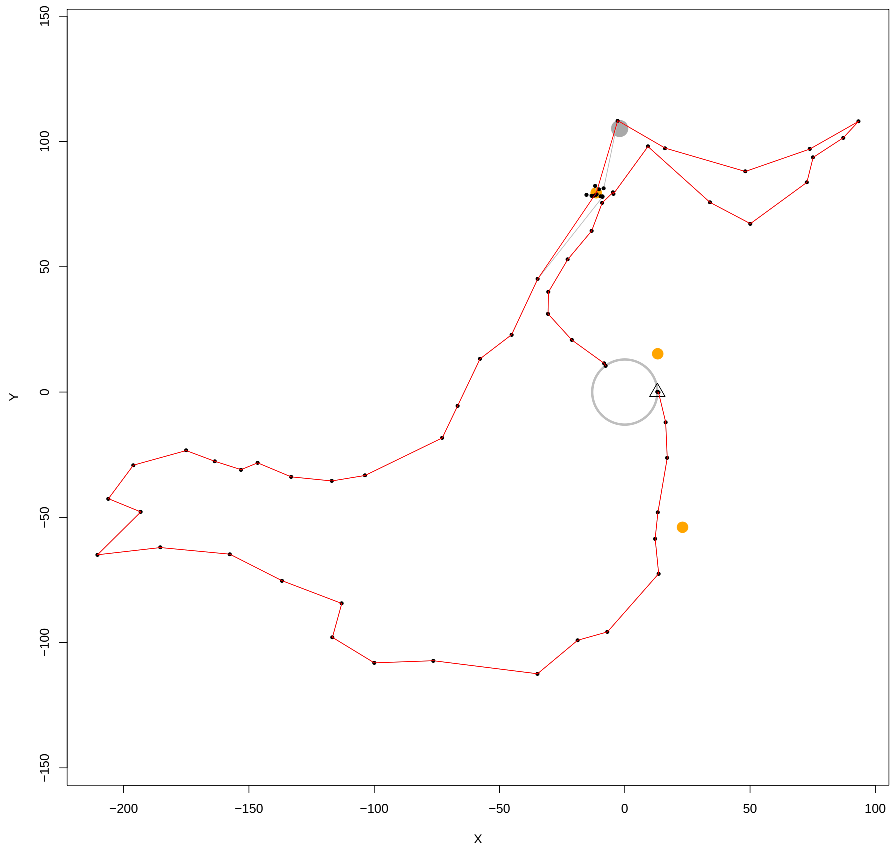

Fig SI-V-8.6 : Loop 47 — loop 6 of bee GE-1

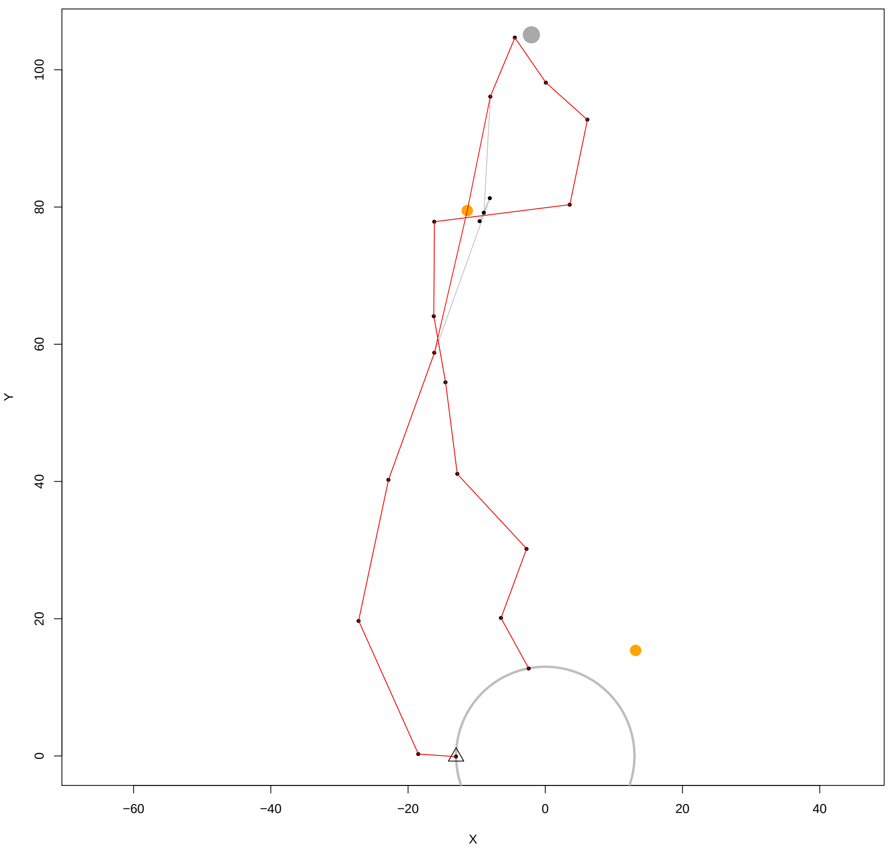

Fig SI-V-8.7 : Loop 48 — loop 7 of bee GE-1

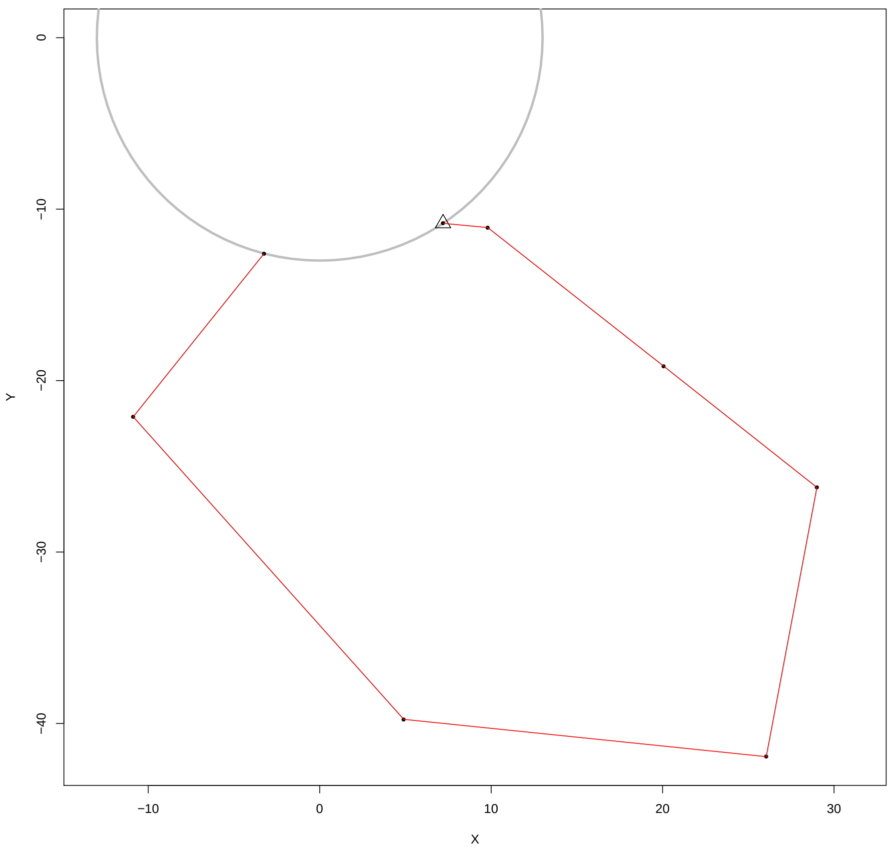

Fig SI-V-8.8 : Loop 49 — loop 8 of bee GE-1

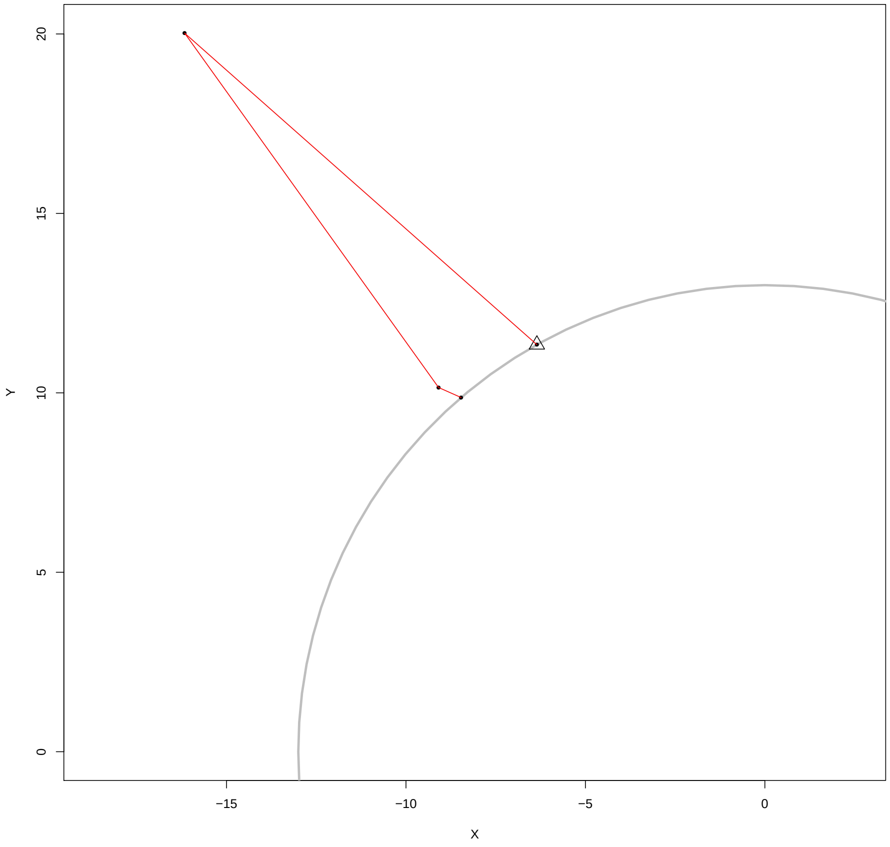

Fig SI-V-8.9 : Loop 50 — loop 9 of bee GE-1

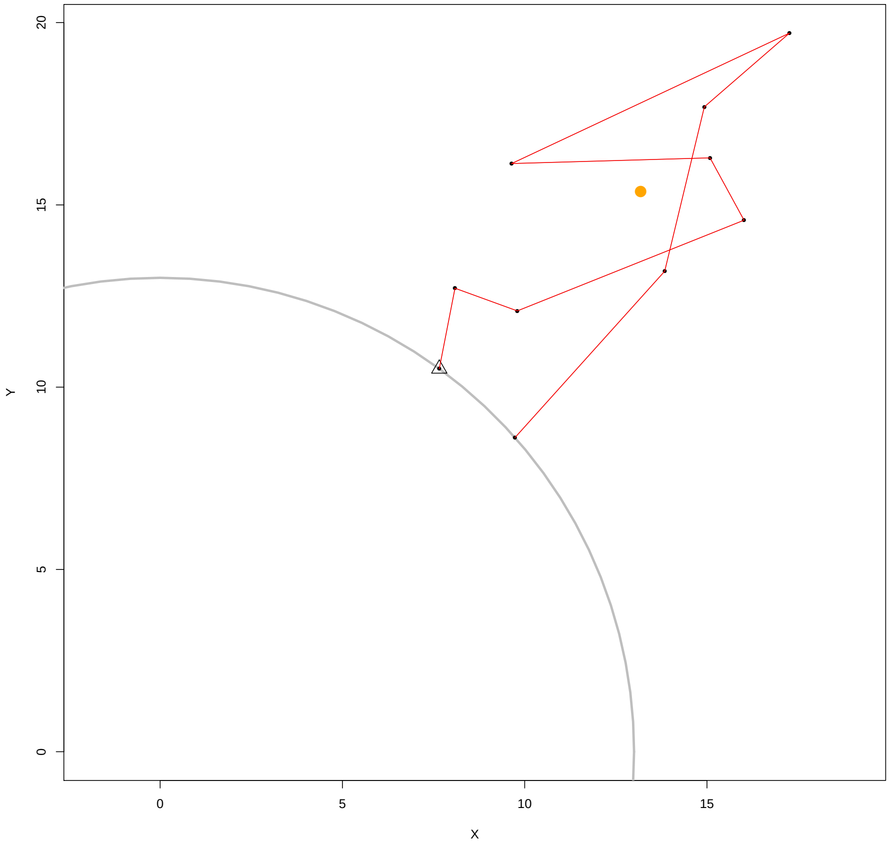

Fig SI-V-8.10 : Loop 51 — loop 10 of bee GE-1

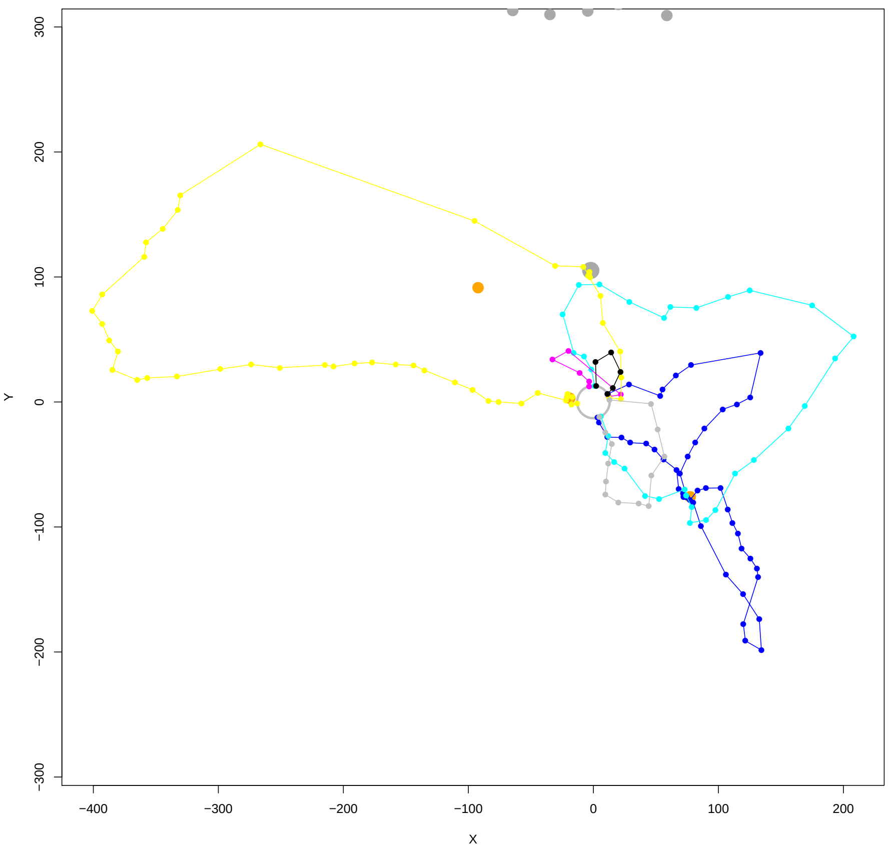

Fig SI-V-9 : Tracks of bee GE-2

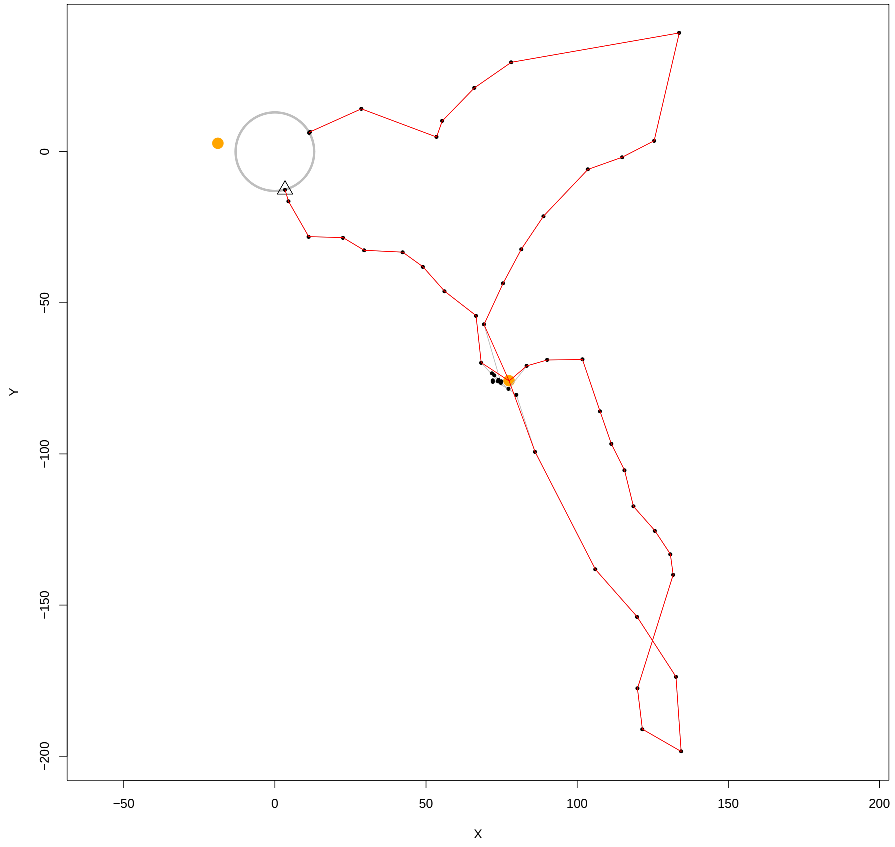

Fig SI-V-9.1 : Loop 52 — loop 1 of bee GE-2

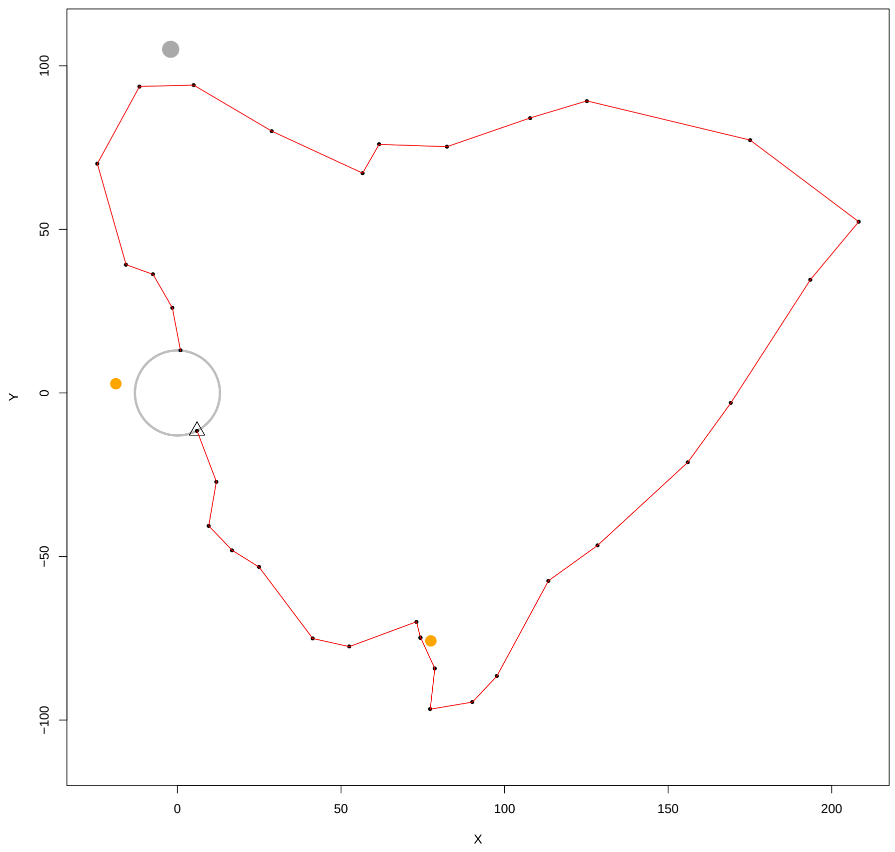

Fig SI-V-9.2 : Loop 53 — loop 2 of bee GE-2

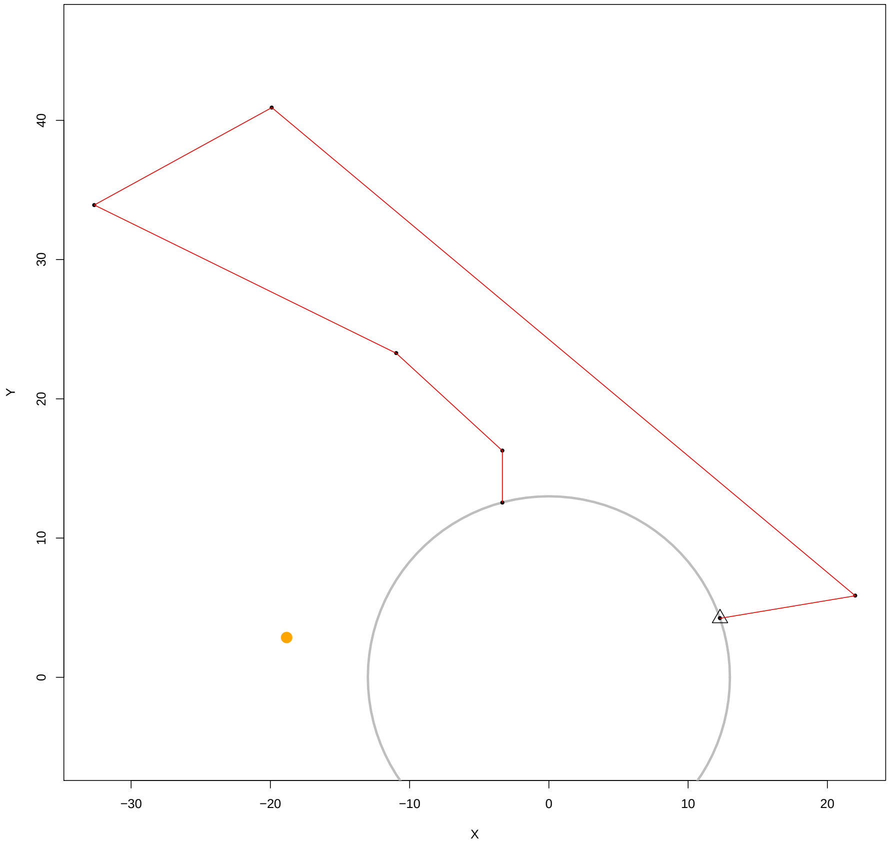

Fig SI-V-9.3 : Loop 54 — loop 3 of bee GE-2

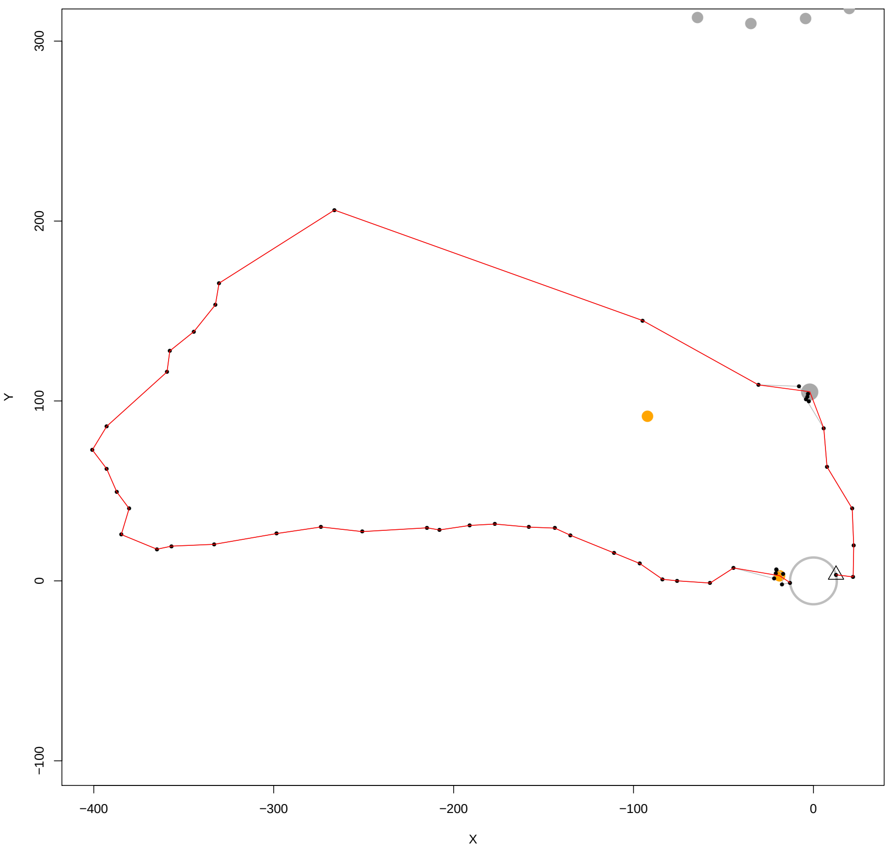

Fig SI-V-9.4 : Loop 55 — loop 4 of bee GE-2

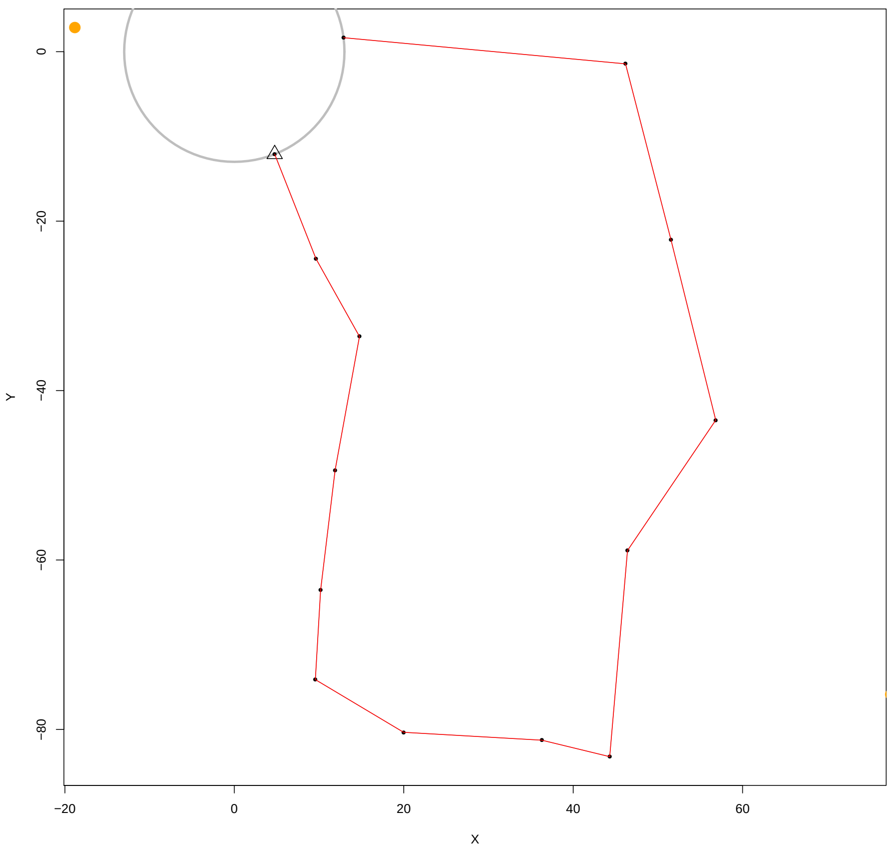

Fig SI-V-9.5 : Loop 56 — loop 5 of bee GE-2

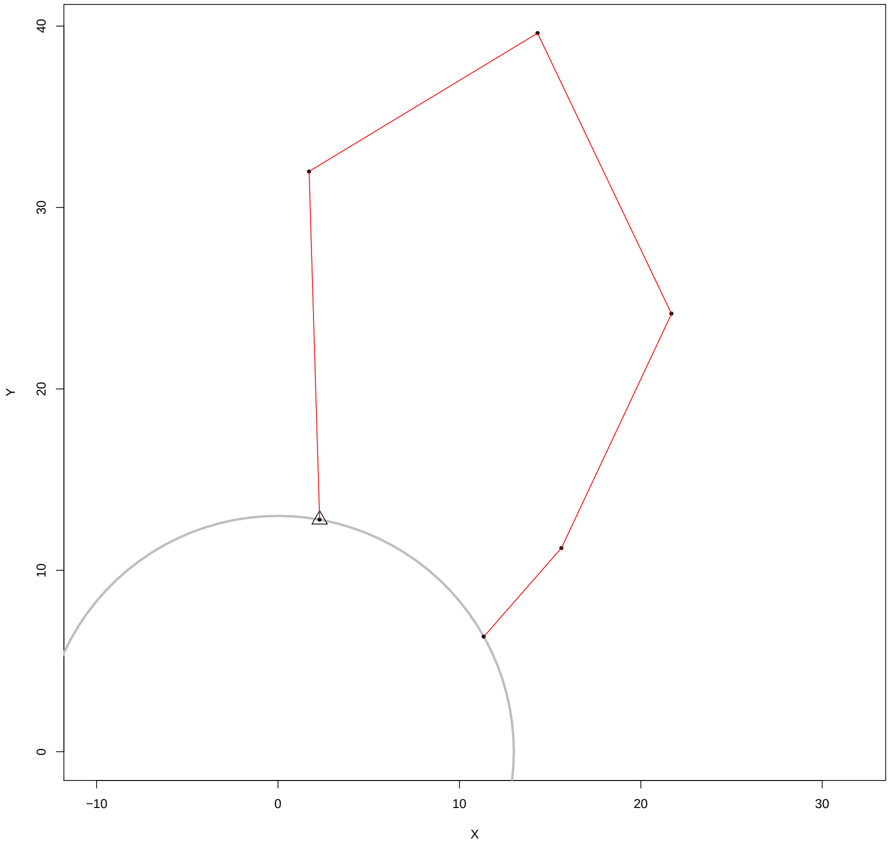

Fig SI-V-9.6 : Loop 57 — loop 6 of bee GE-2

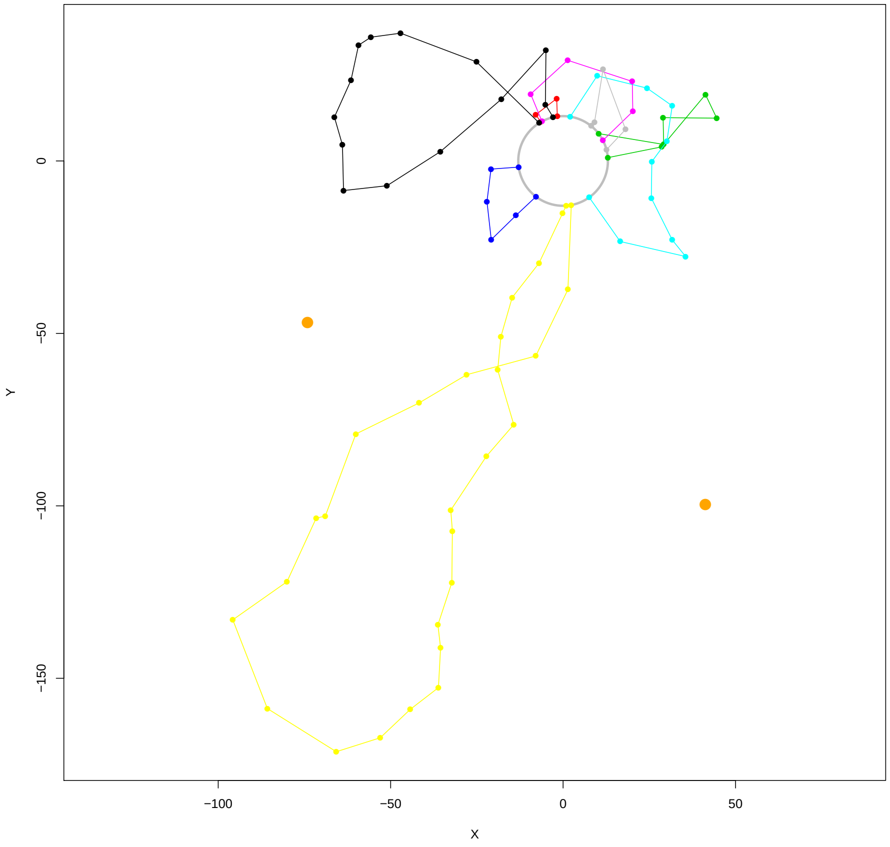

Fig SI-V-10 : Tracks of bee JS-4

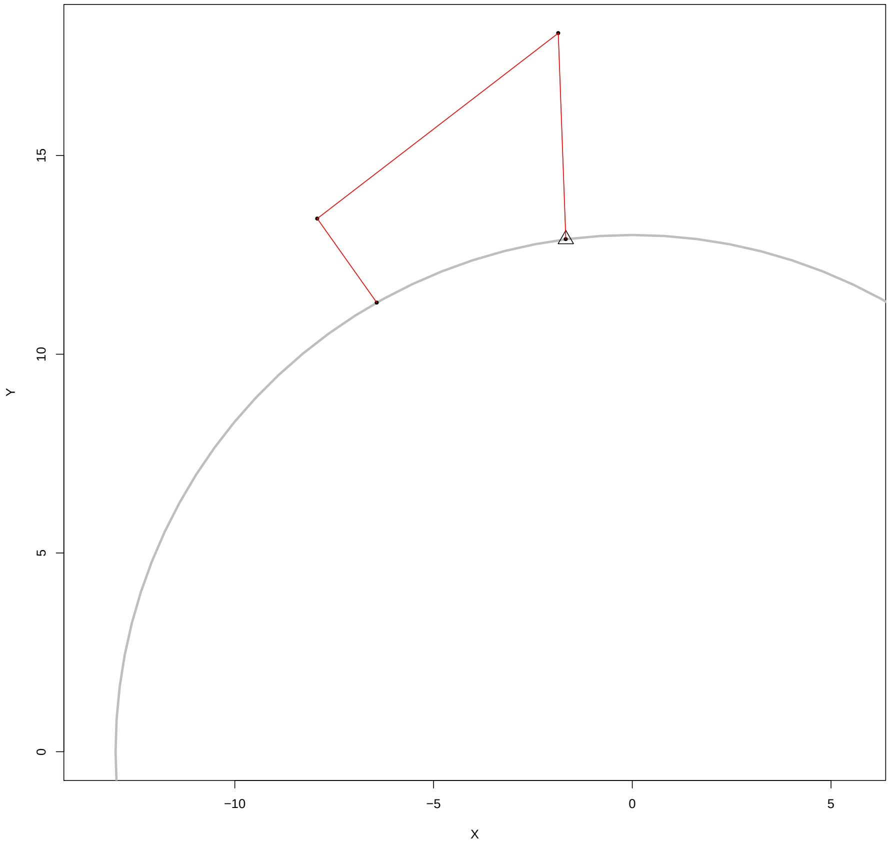

Fig SI-V-10.1 : Loop 58 — loop 1 of bee JS-4

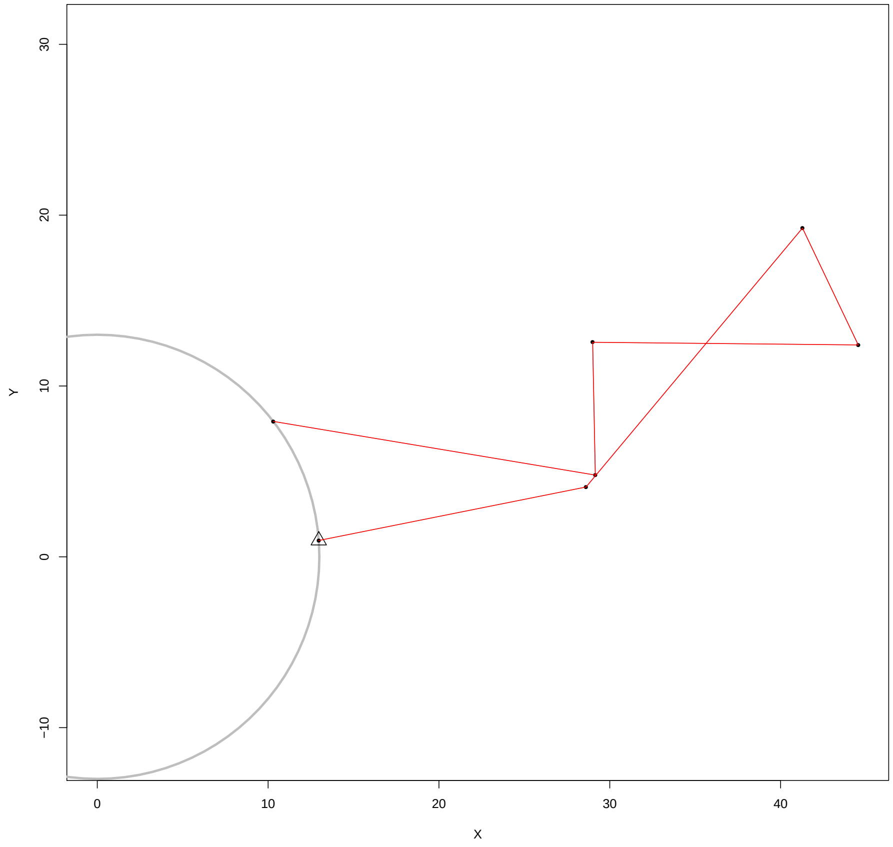

Fig SI-V-10.2 : Loop 59 — loop 2 of bee JS-4

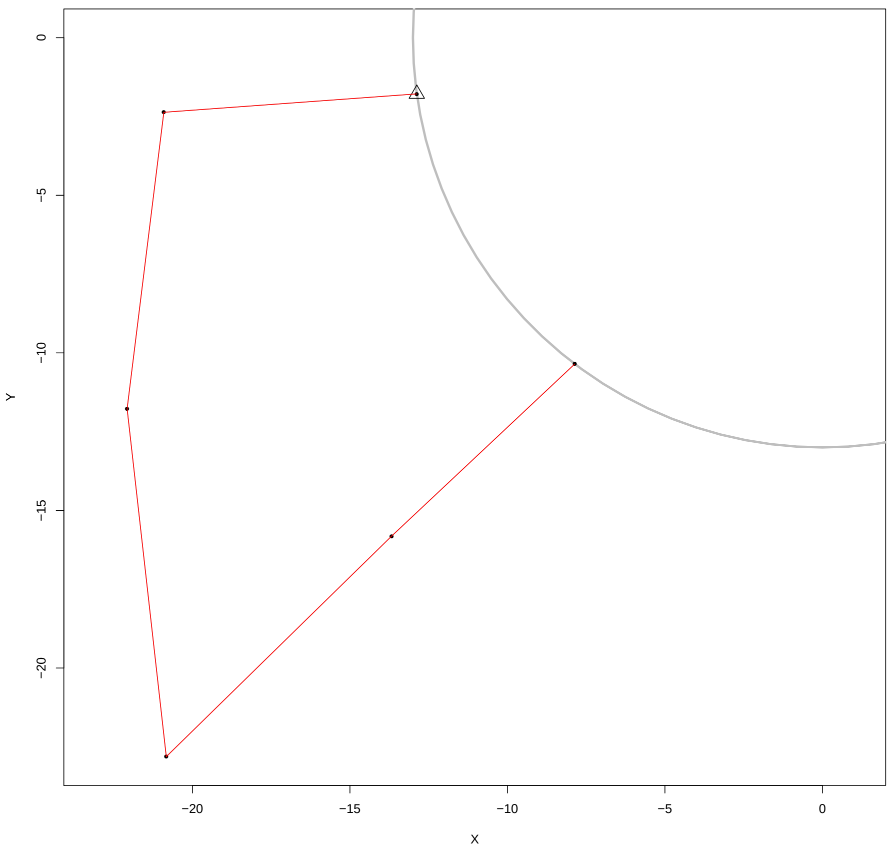

Fig SI-V-10.3 : Loop 60 — loop 3 of bee JS-4

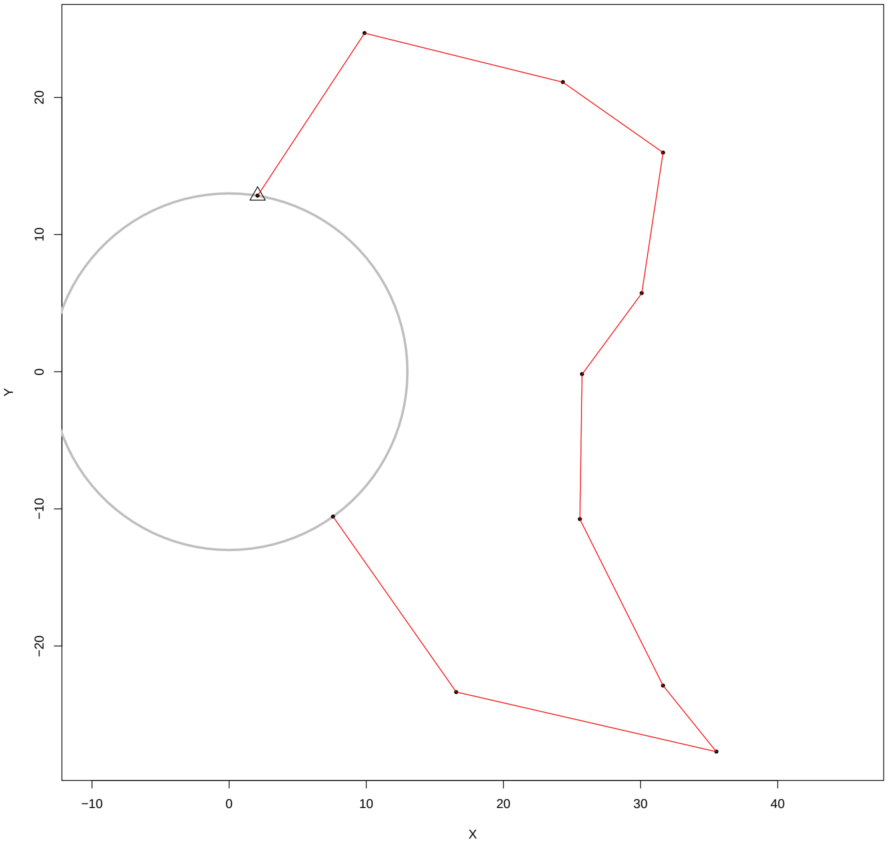

Fig SI-V-10.4 : Loop 61 — loop 4 of bee JS-4

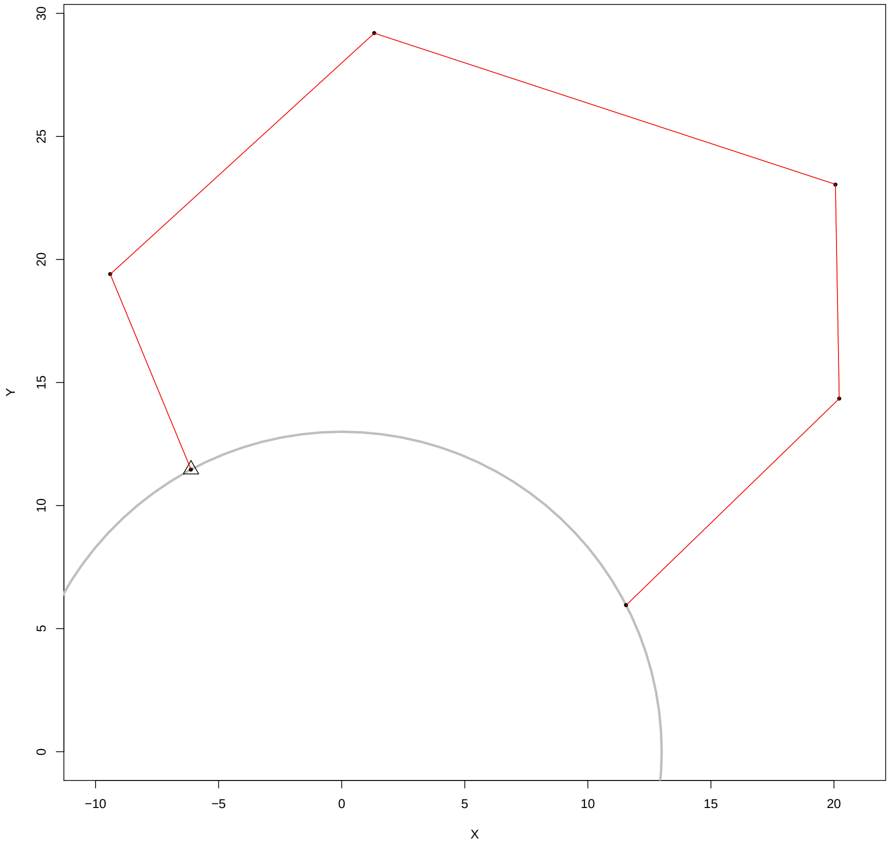

Fig SI-V-10.5 : Loop 62 — loop 5 of bee JS-4

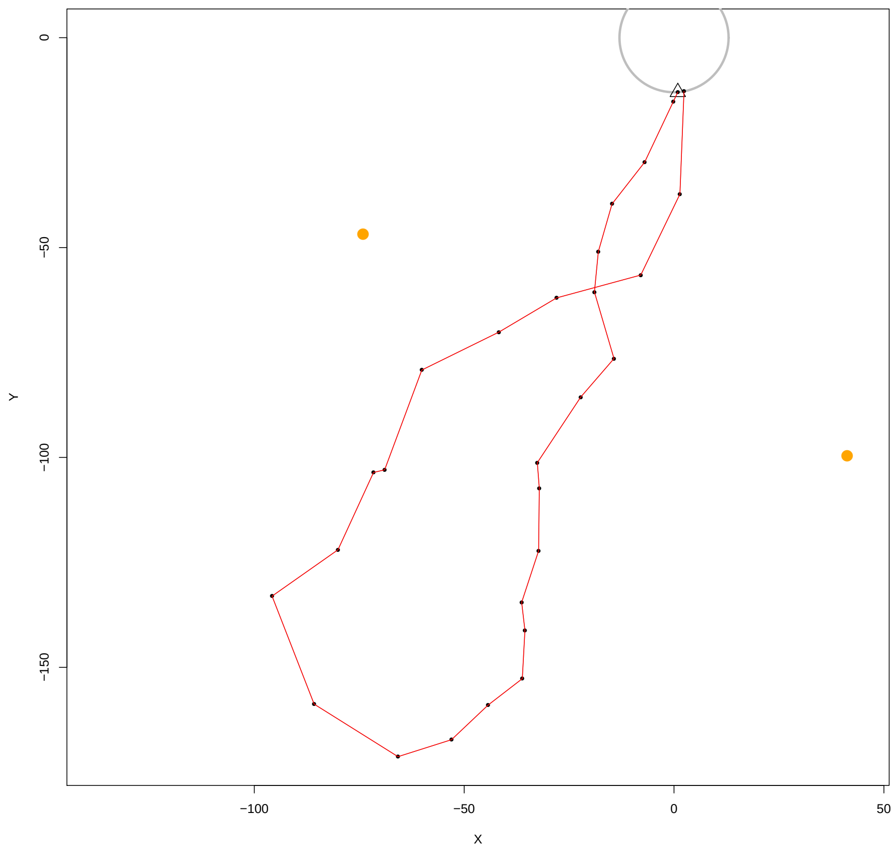

Fig SI-V-10.6 : Loop 63 — loop 6 of bee JS-4

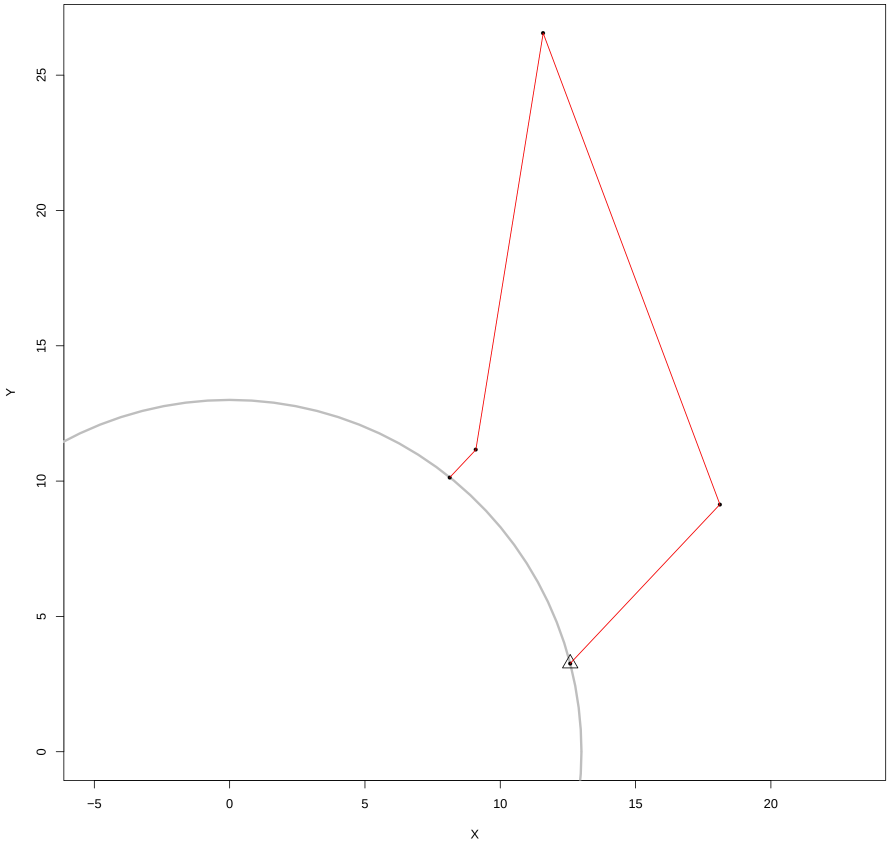

Fig SI-V-10.7 : Loop 64 — loop 7 of bee JS-4

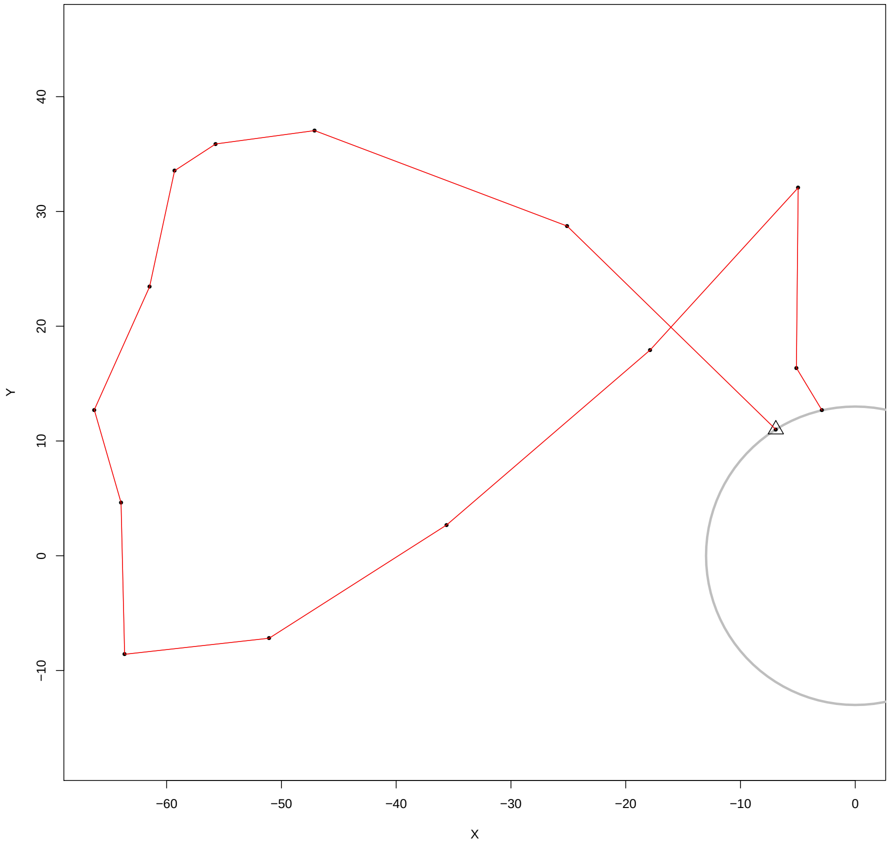

Fig SI-V-10.8 : Loop 65 — loop 8 of bee JS-4

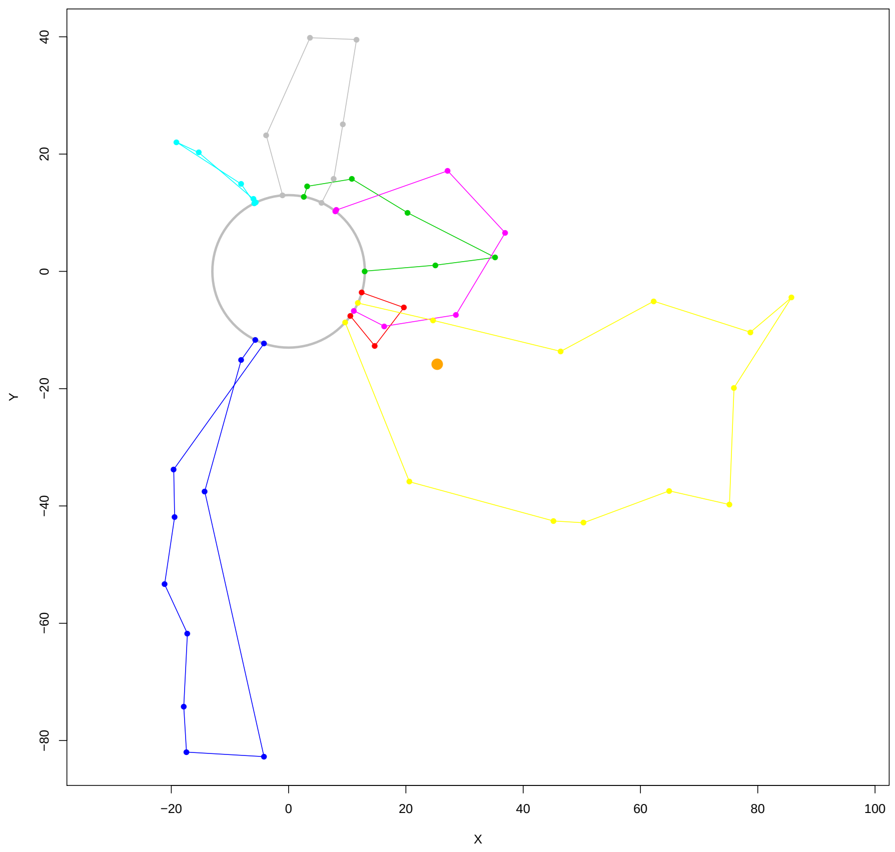

Fig SI-V-11 : Tracks of bee JP-2

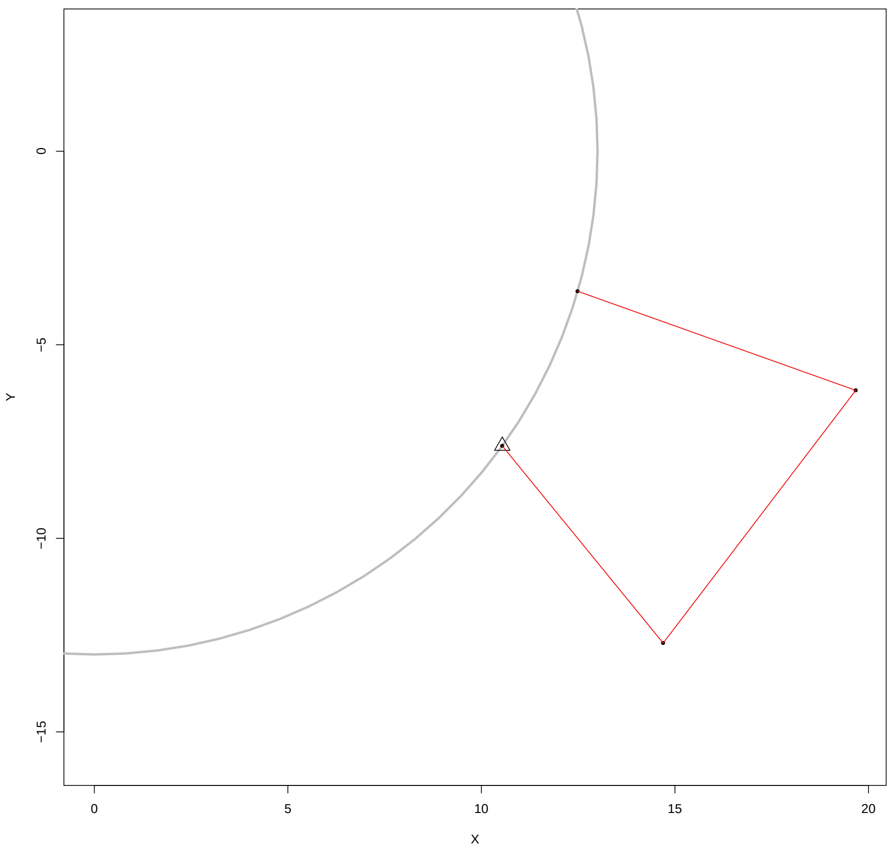

Fig SI-V-11.1 : Loop 66 — loop 1 of bee JP-2

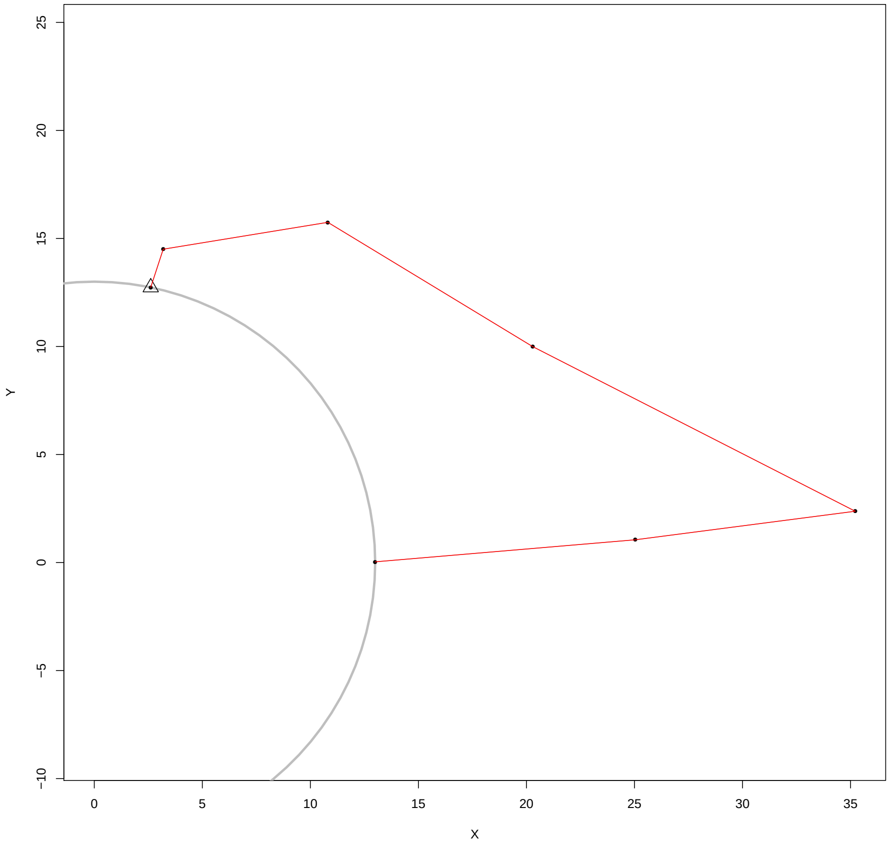

Fig SI-V-11.2 : Loop 67 — loop 2 of bee JP-2

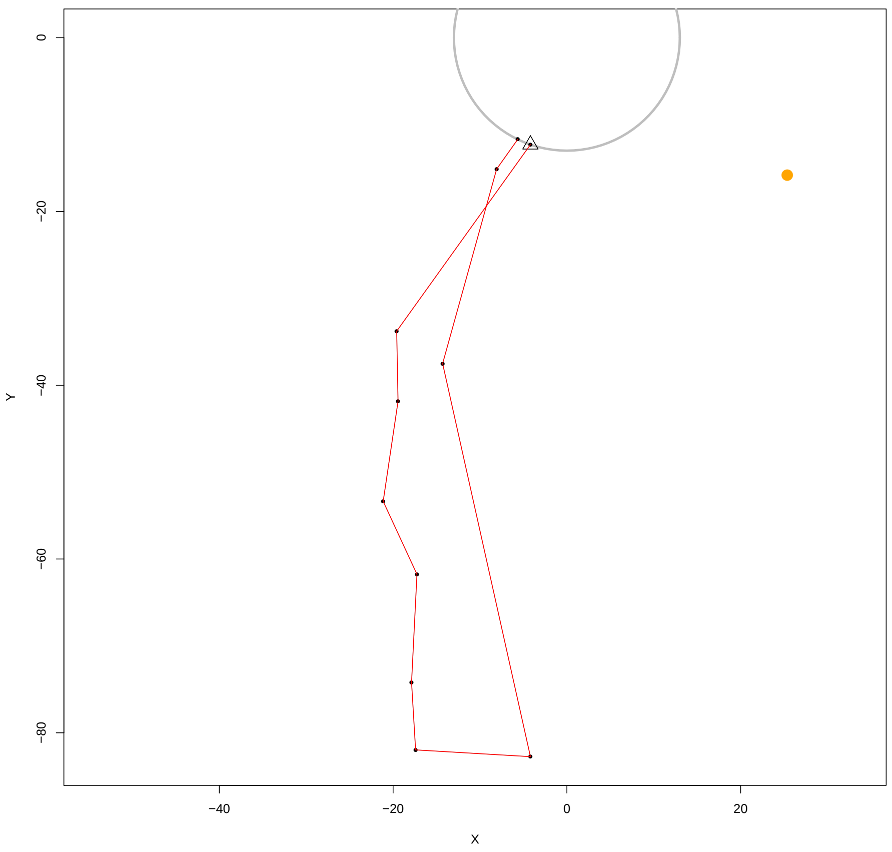

Fig SI-V-11.3 : Loop 68 — loop 3 of bee JP-2

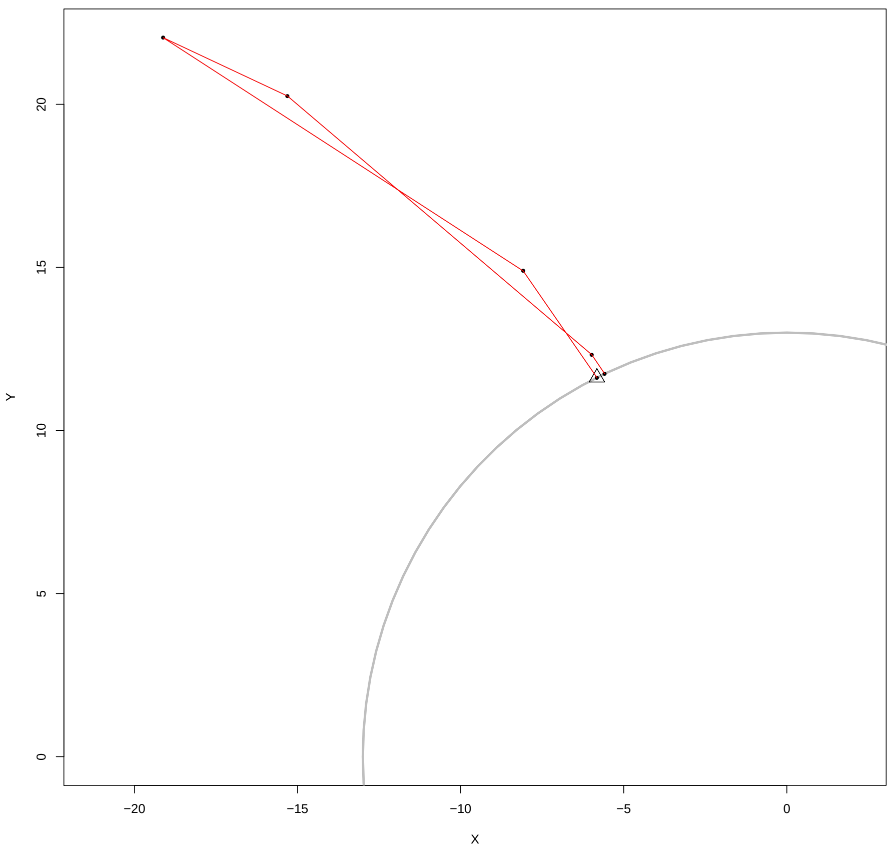

Fig SI-V-11.4 : Loop 69 — loop 4 of bee JP-2

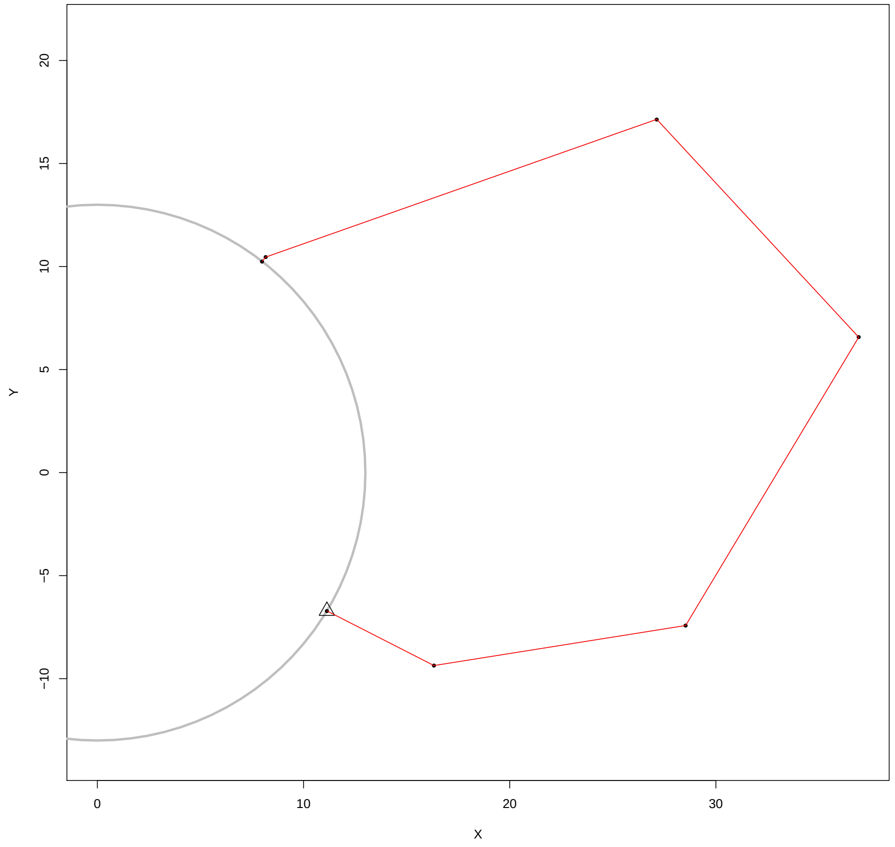

Fig SI-V-11.5 : Loop 70 — loop 5 of bee JP-2

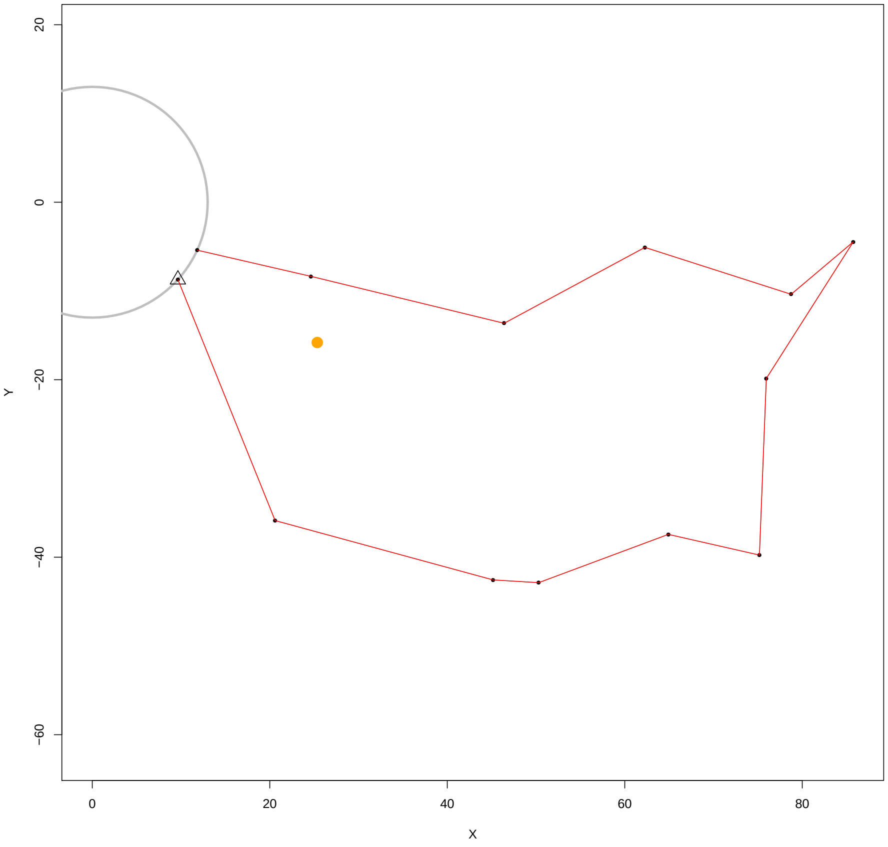

Fig SI-V-11.6 : Loop 71 — loop 6 of bee JP-2

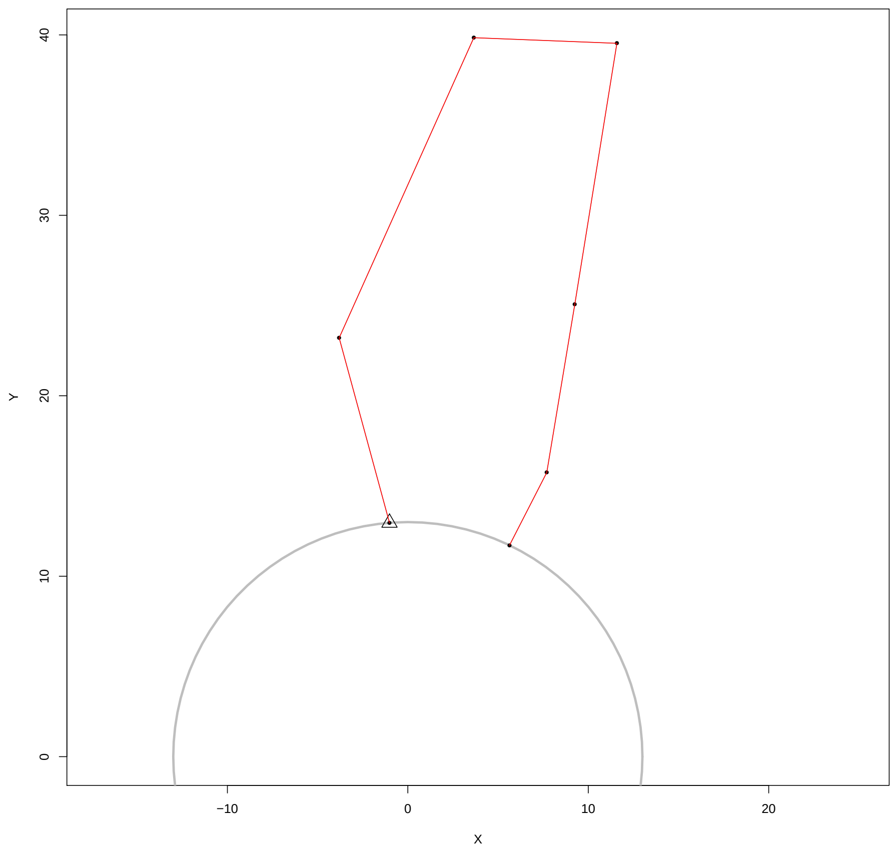

Fig SI-V-11.7 : Loop 72 — loop 7 of bee JP-2

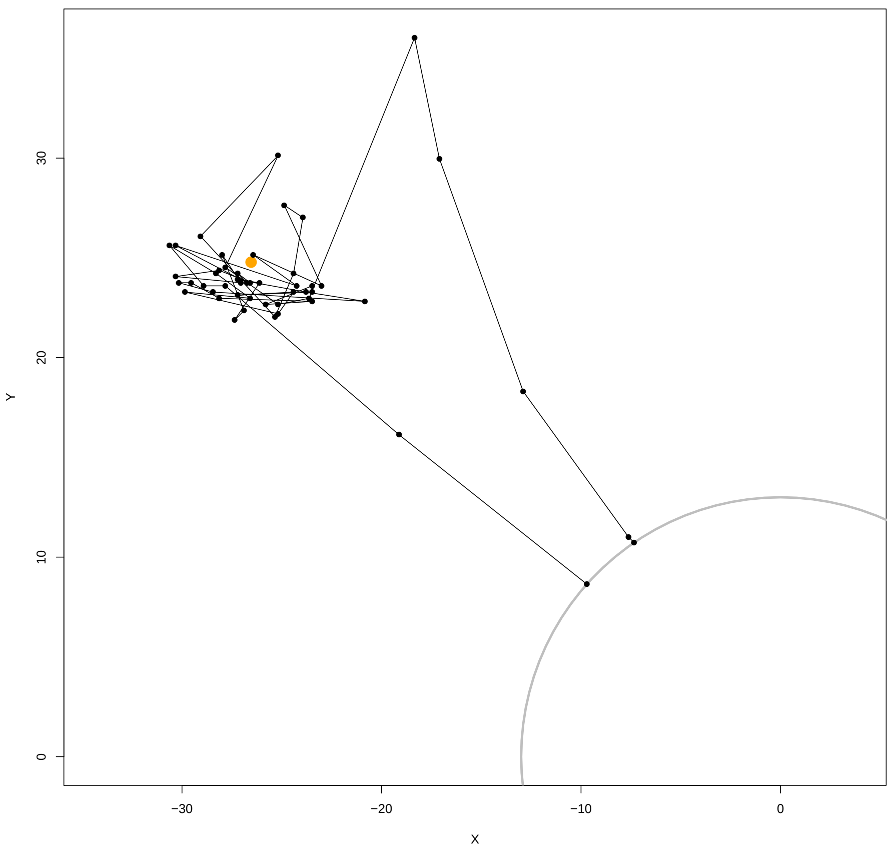

Fig SI-V-12 : Tracks of bee GH-3

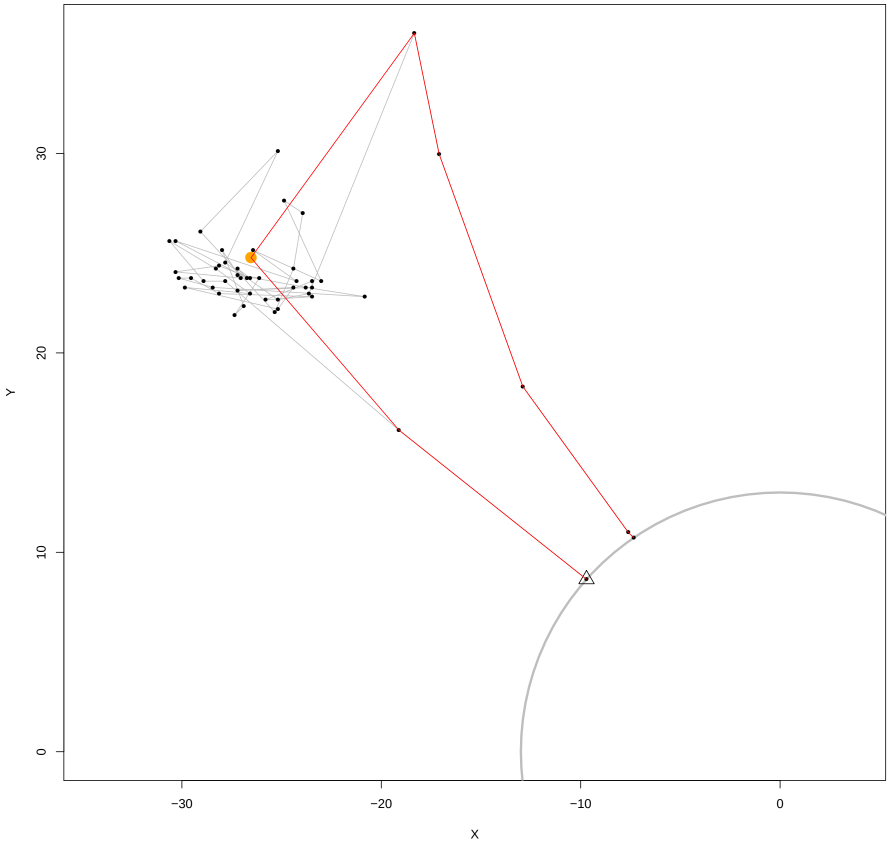

Fig SI-V-12.1 : Loop 73 — loop 1 of bee GH-3

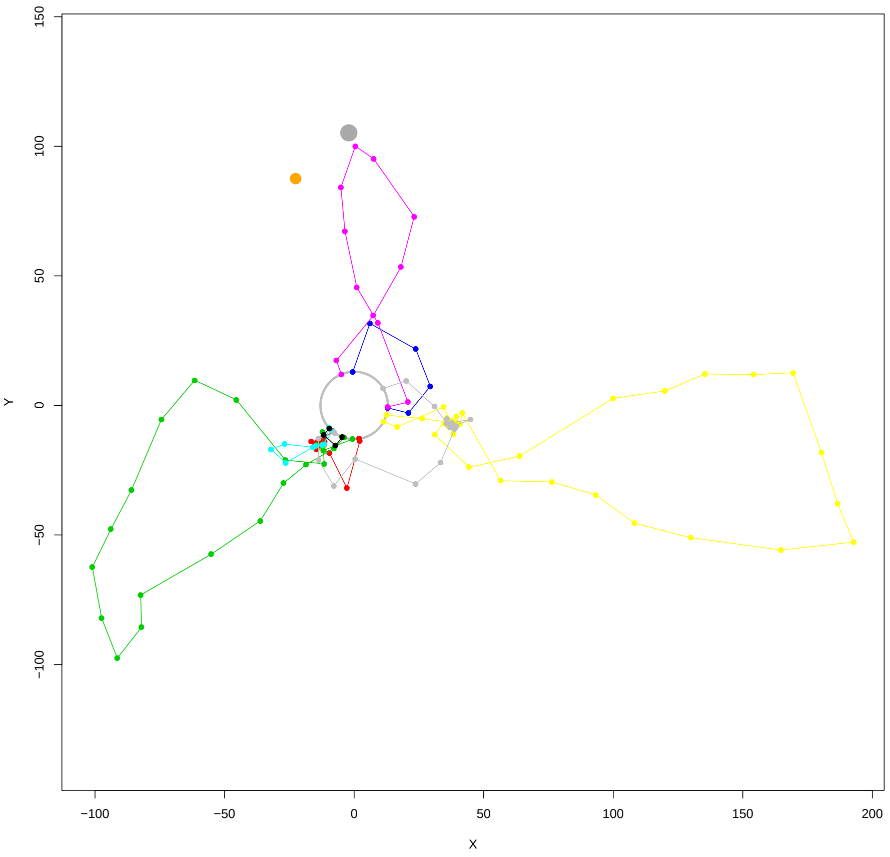

Fig SI-V-13 : Tracks of bee JT-1

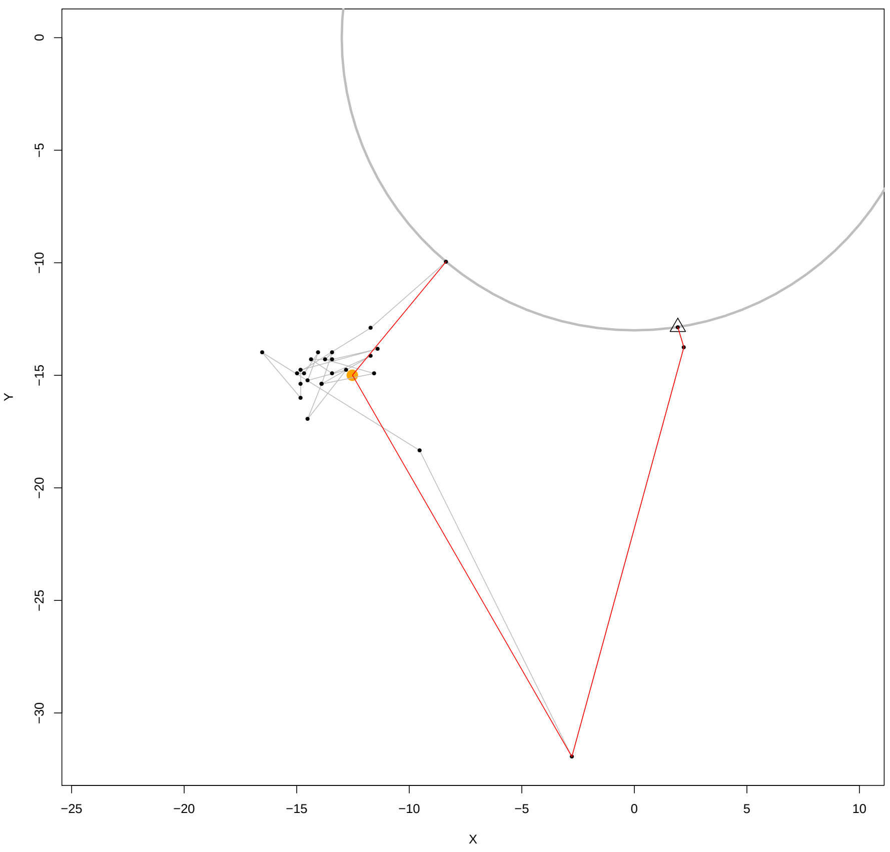

Fig SI-V-13.1 : Loop 74 — loop 1 of bee JT-1

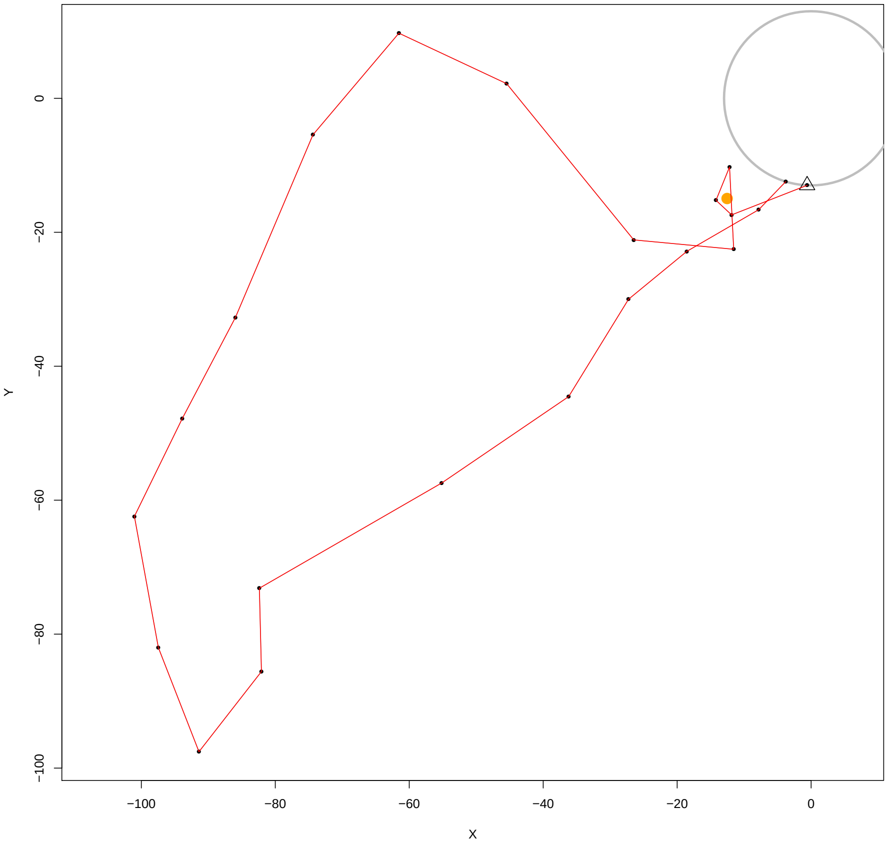

Fig SI-V-13.2 : Loop 75 — loop 2 of bee JT-1

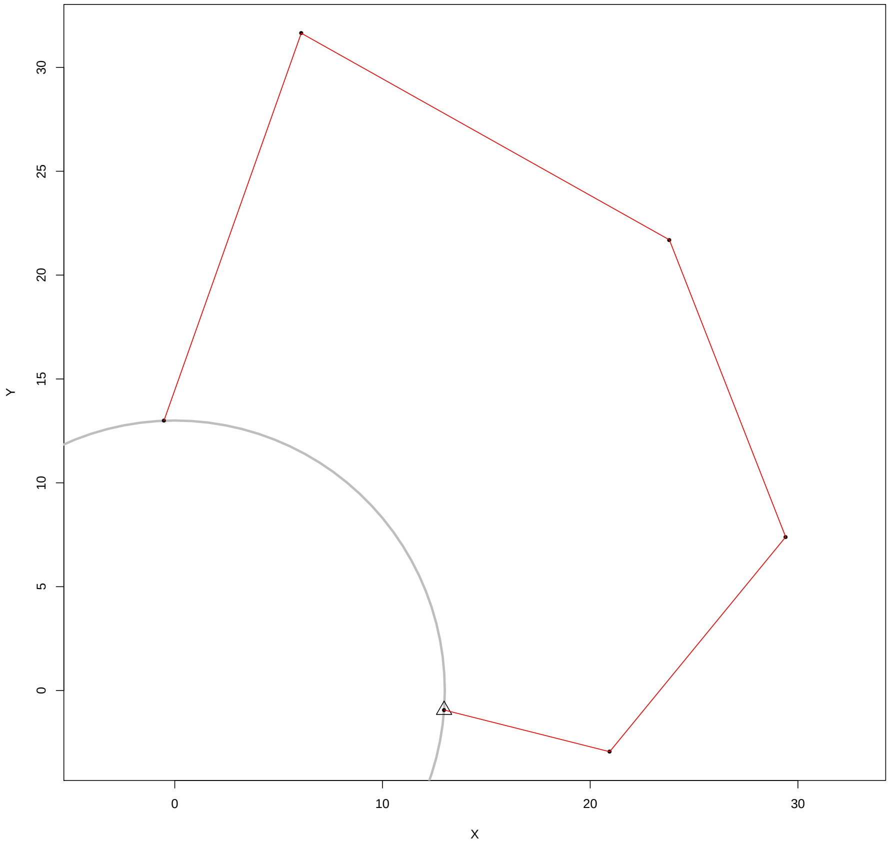

Fig SI-V-13.3 : Loop 76 — loop 3 of bee JT-1

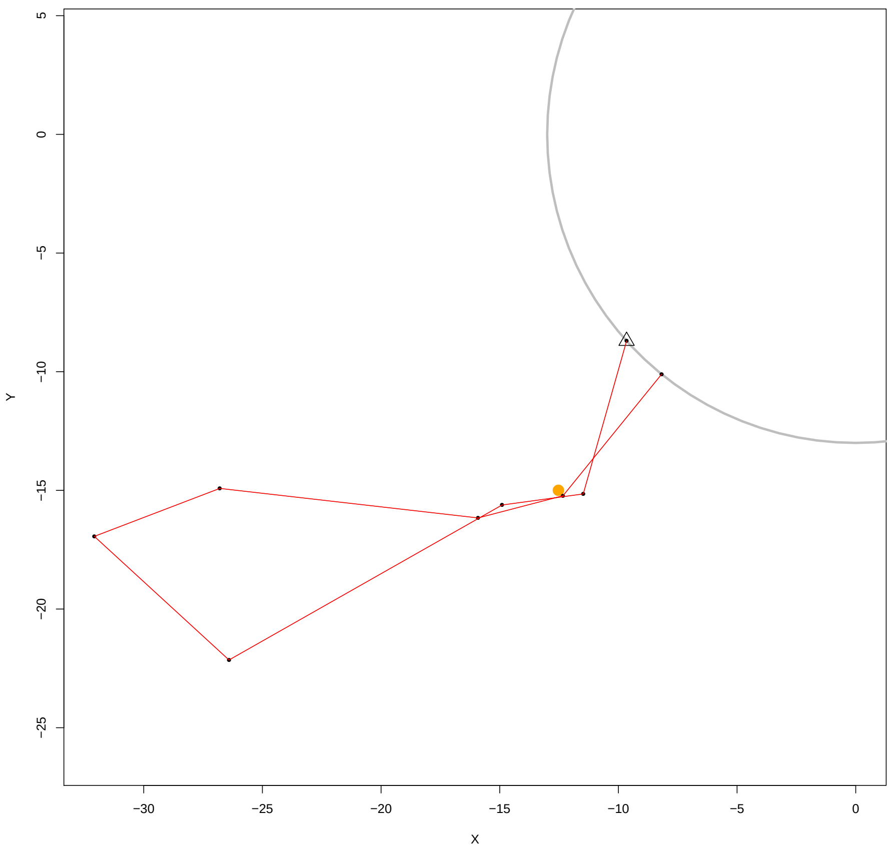

Fig SI-V-13.4 : Loop 77 — loop 4 of bee JT-1

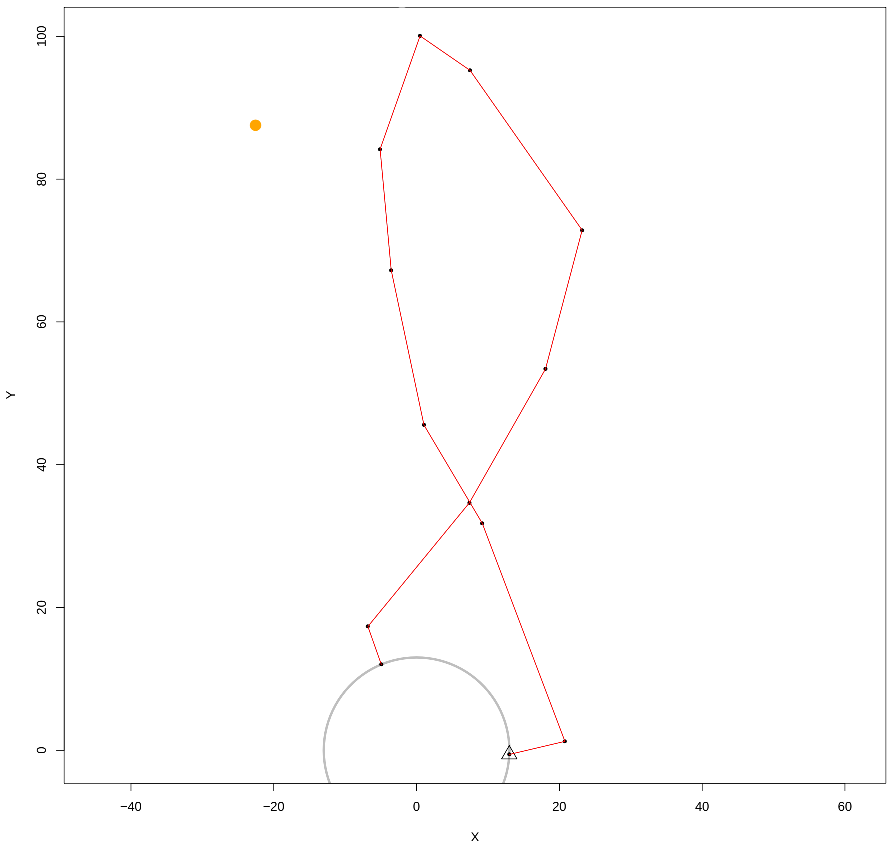

Fig SI-V-13.5 : Loop 78 — loop 5 of bee JT-1

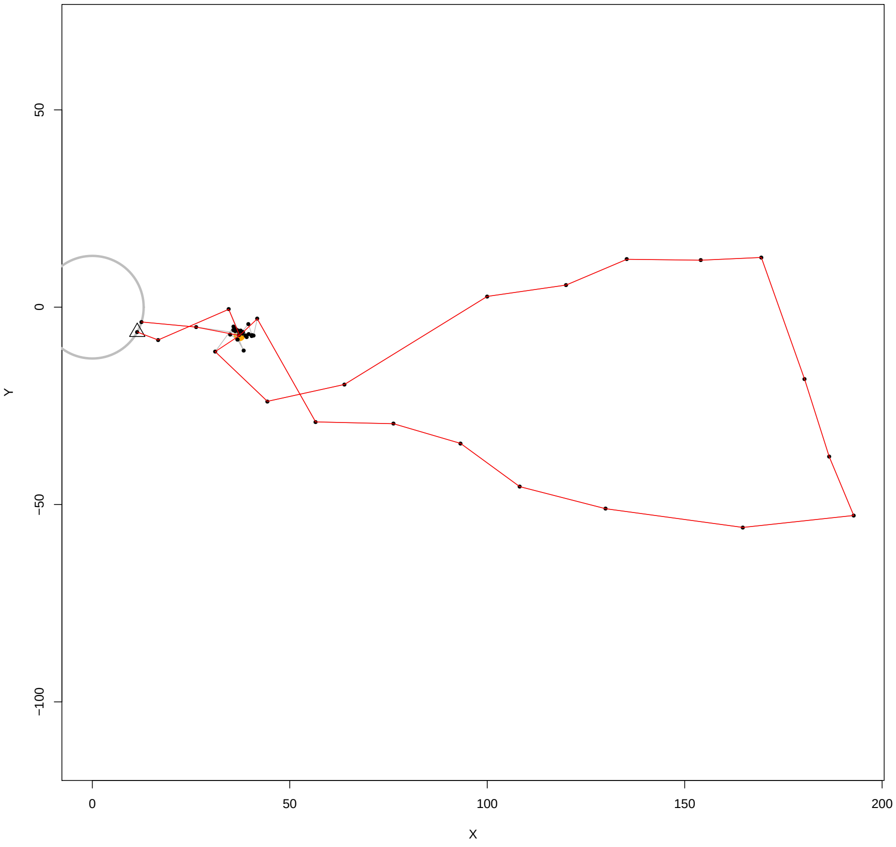

Fig SI-V-13.6 : Loop 79 — loop 6 of bee JT-1

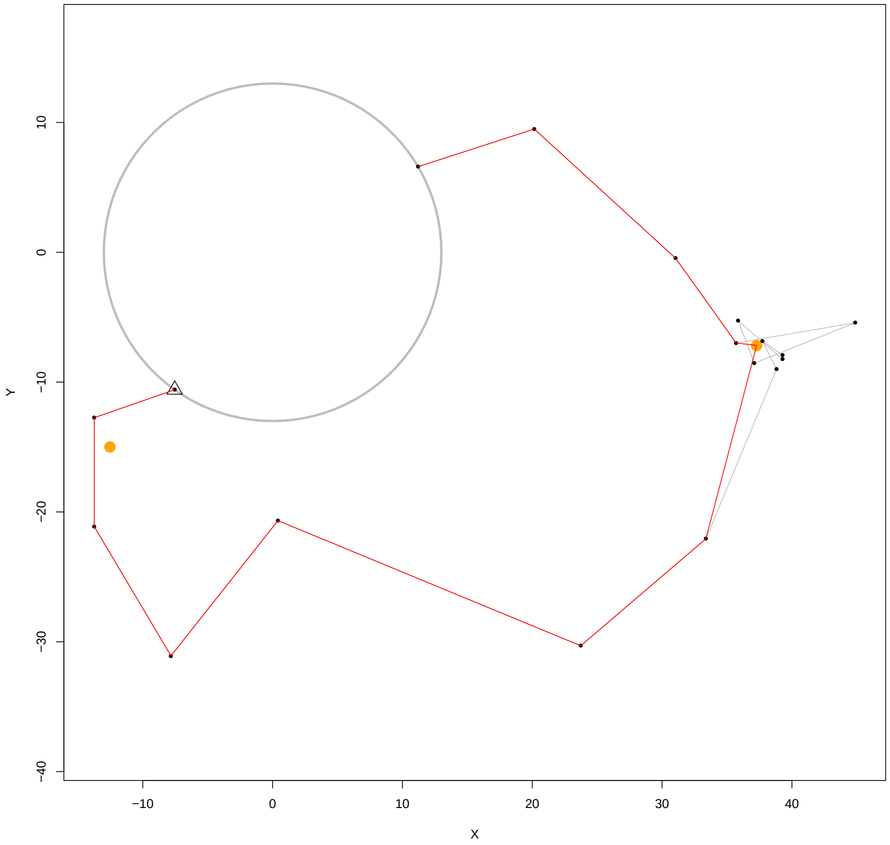

Fig SI-V-13.7 : Loop 80 — loop 7 of bee JT-1

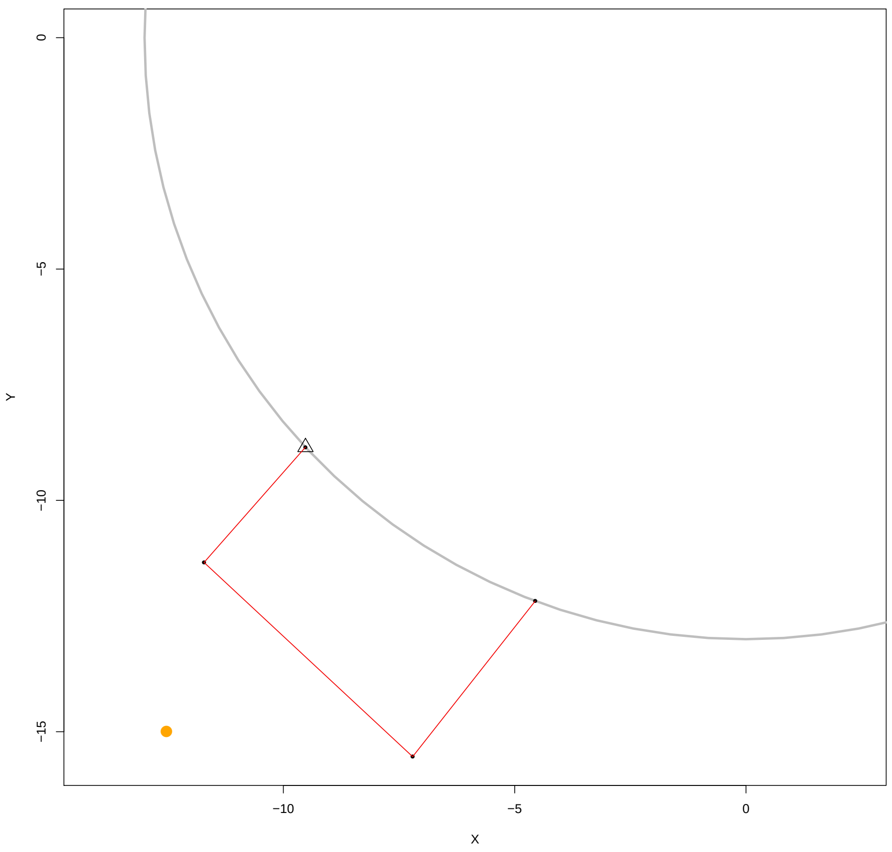

Fig SI-V-13.8 : Loop 81 — loop 8 of bee JT-1

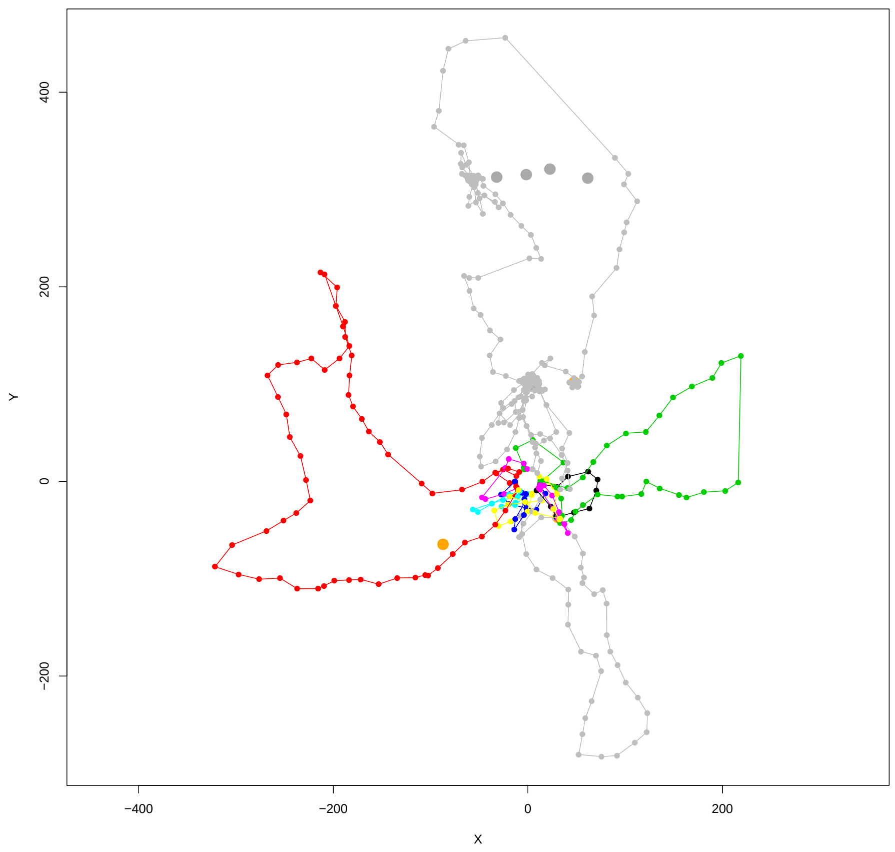

Fig SI-V-14 : Tracks of bee JT-2

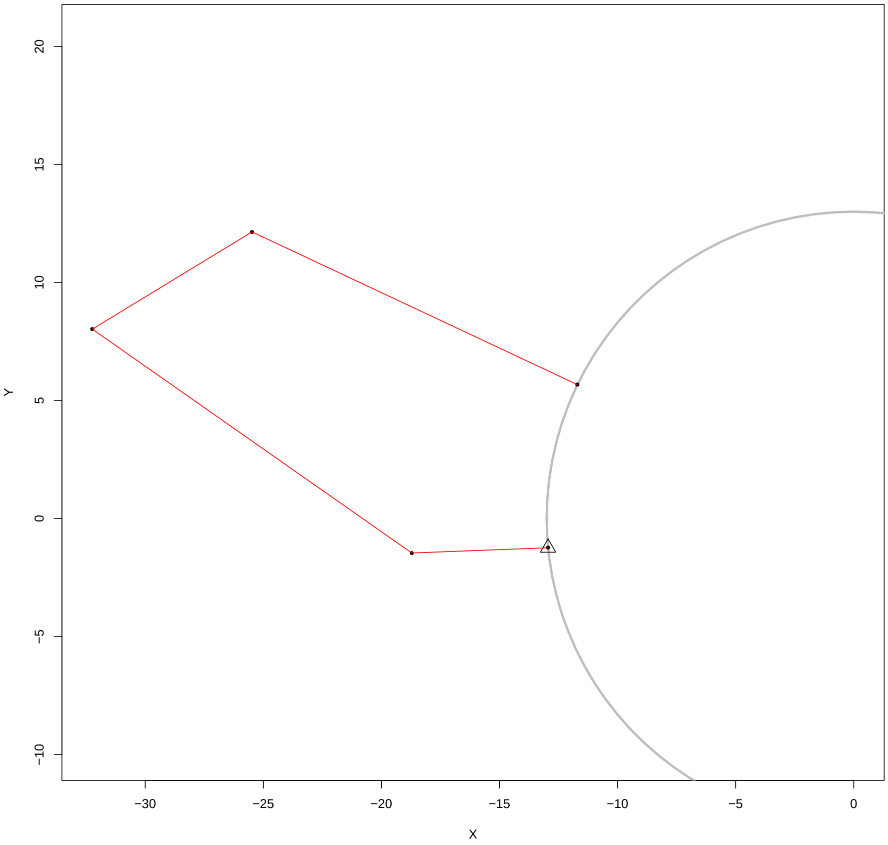

Fig SI-V-14.1 : Loop 82 — loop 1 of bee JT-2

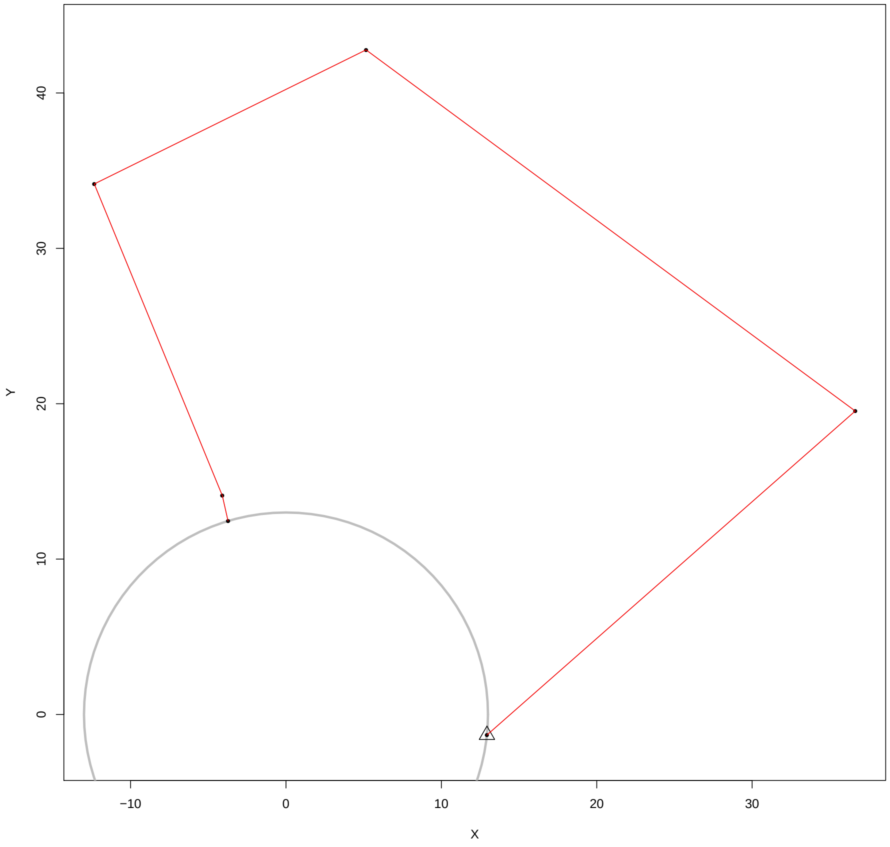

Fig SI-V-14.2 : Loop 83 — loop 2 of bee JT-2

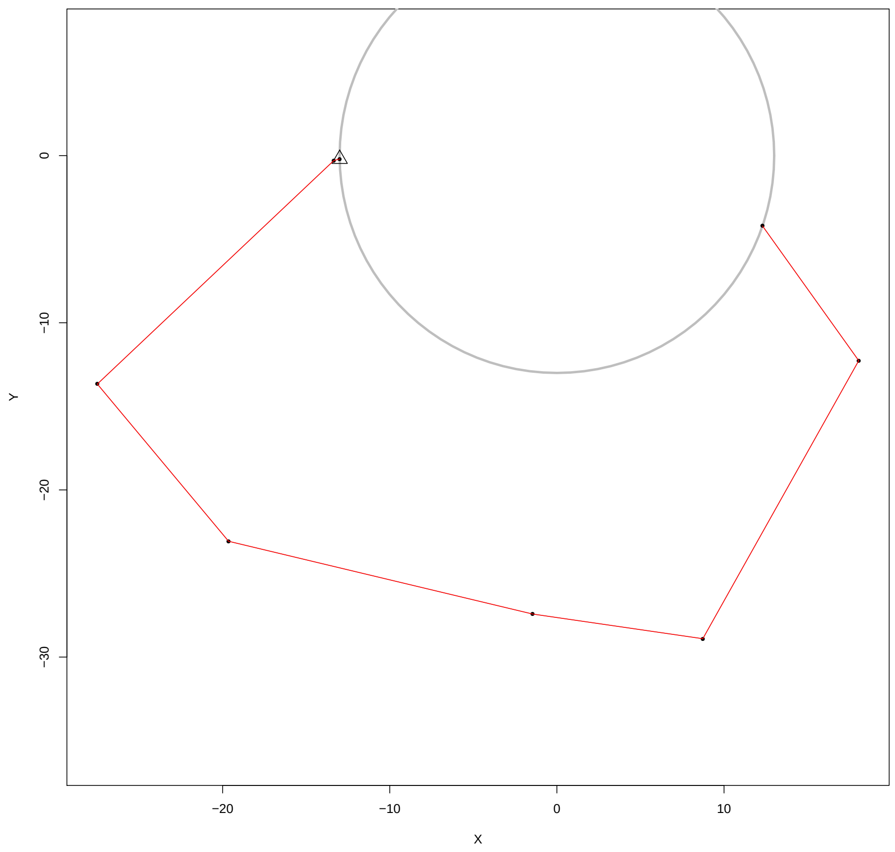

Fig SI-V-14.3 : Loop 84 — loop 3 of bee JT-2

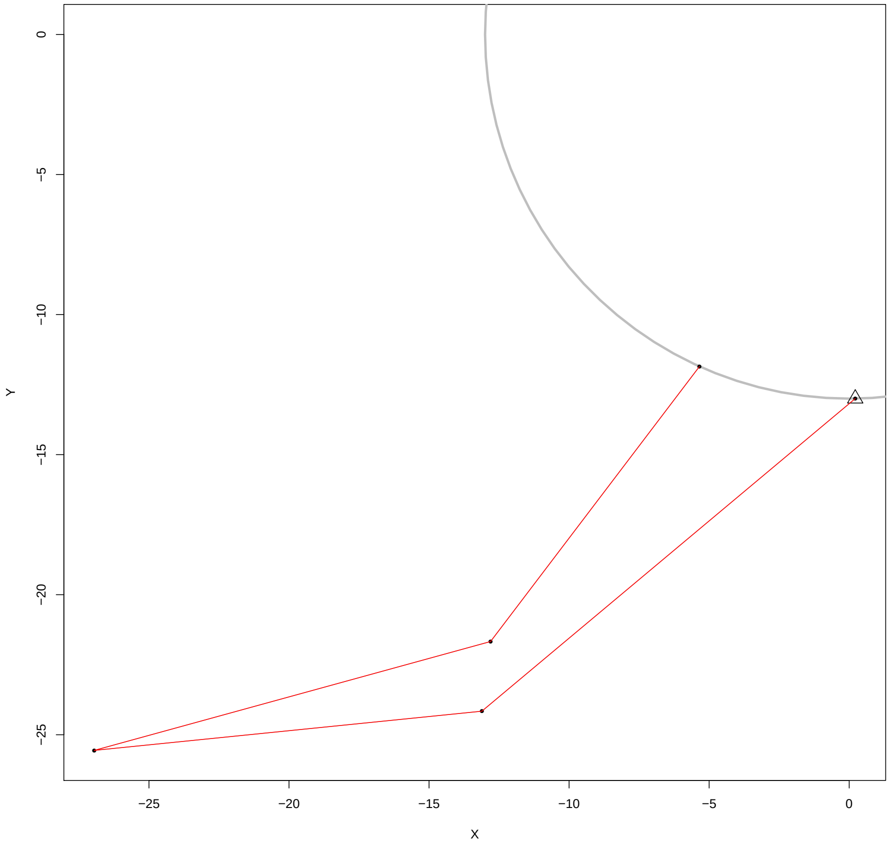

Fig SI-V-14.4 : Loop 85 — loop 4 of bee JT-2

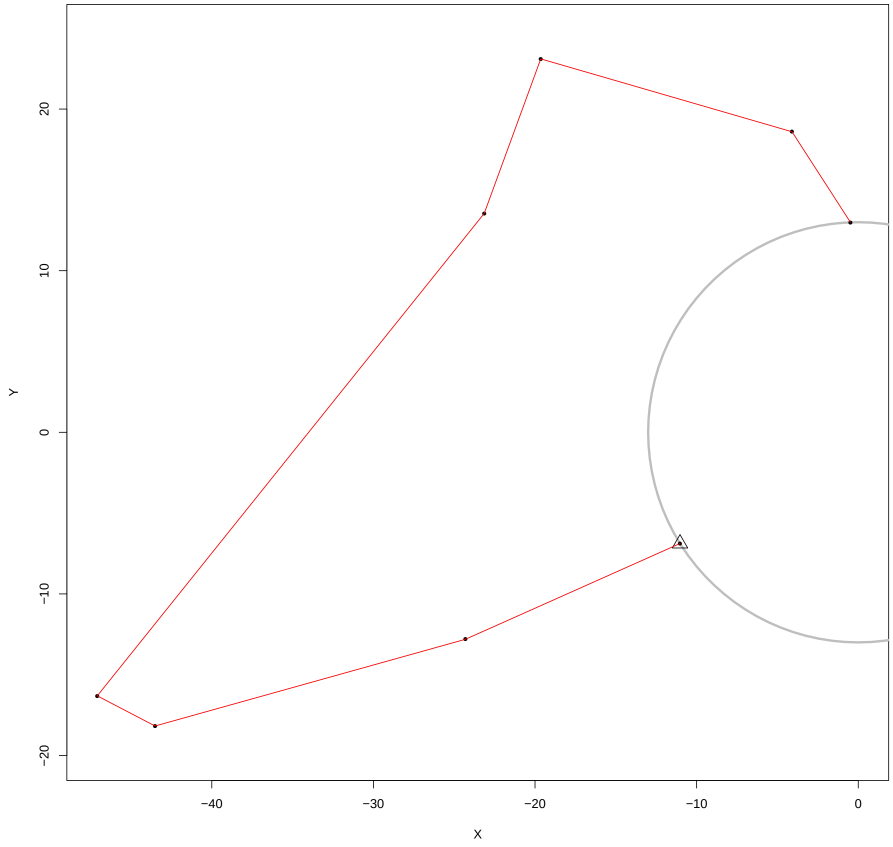

Fig SI-V-14.5 : Loop 86 — loop 5 of bee JT-2

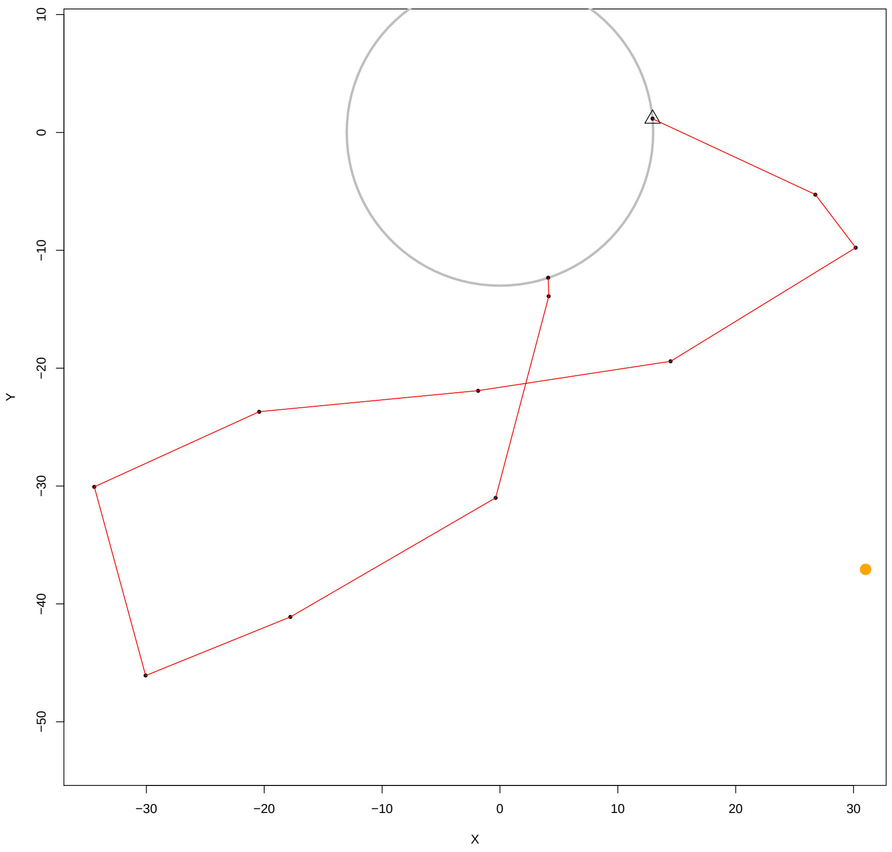

Fig SI-V-14.6 : Loop 87 — loop 6 of bee JT-2

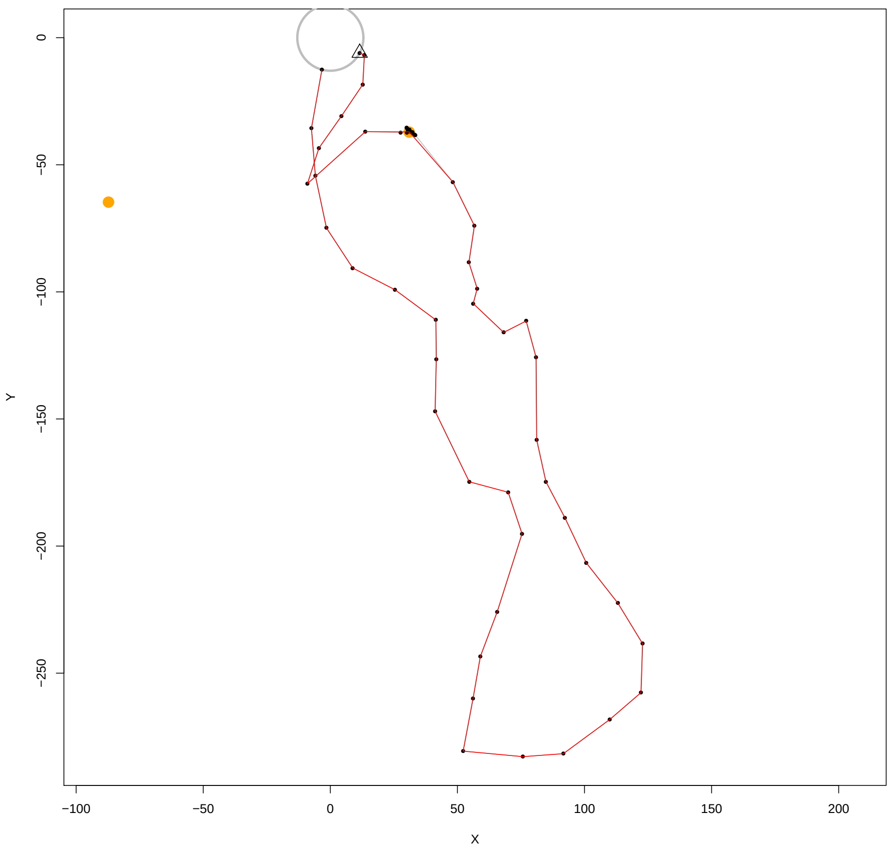

Fig SI-V-14.7 : Loop 88 — loop 7 of bee JT-2

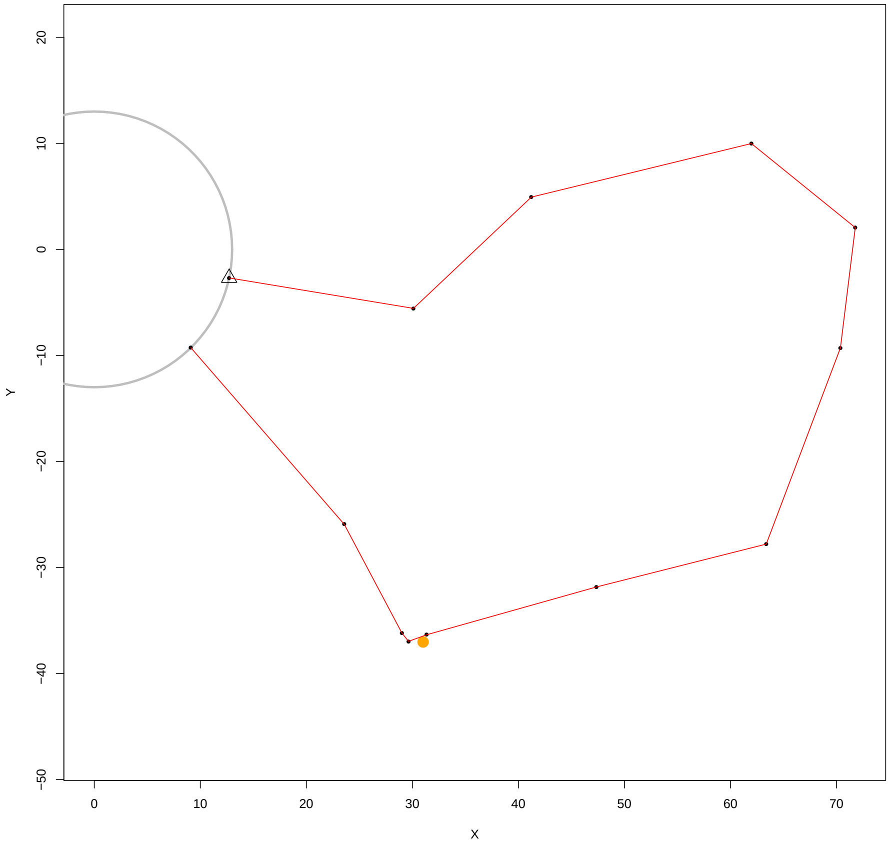

Fig SI-V-14.8 : Loop 89 — loop 8 of bee JT-2

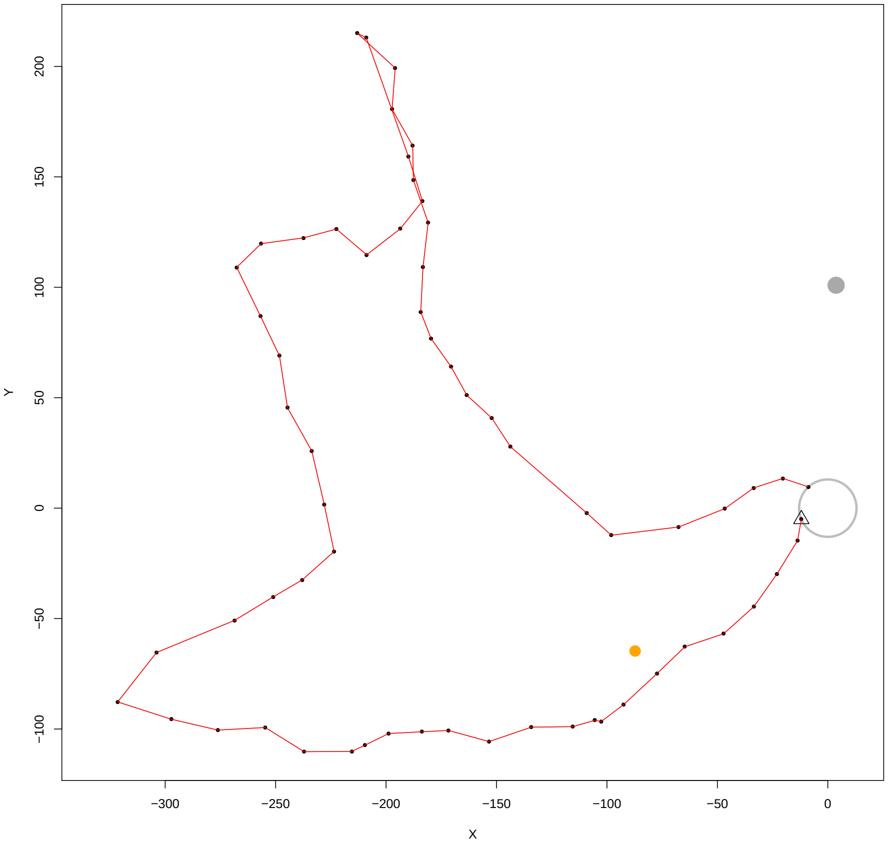

Fig SI-V-14.9 : Loop 90 — loop 9 of bee JT-2

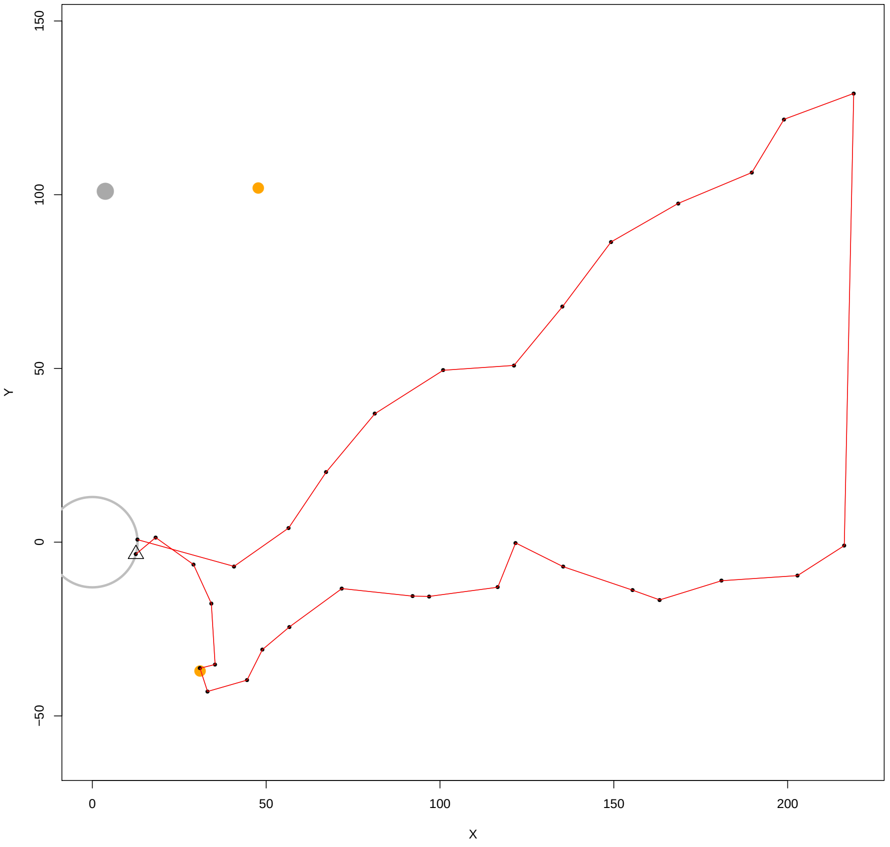

Fig SI-V-14.10 : Loop 91 — loop 10 of bee JT-2

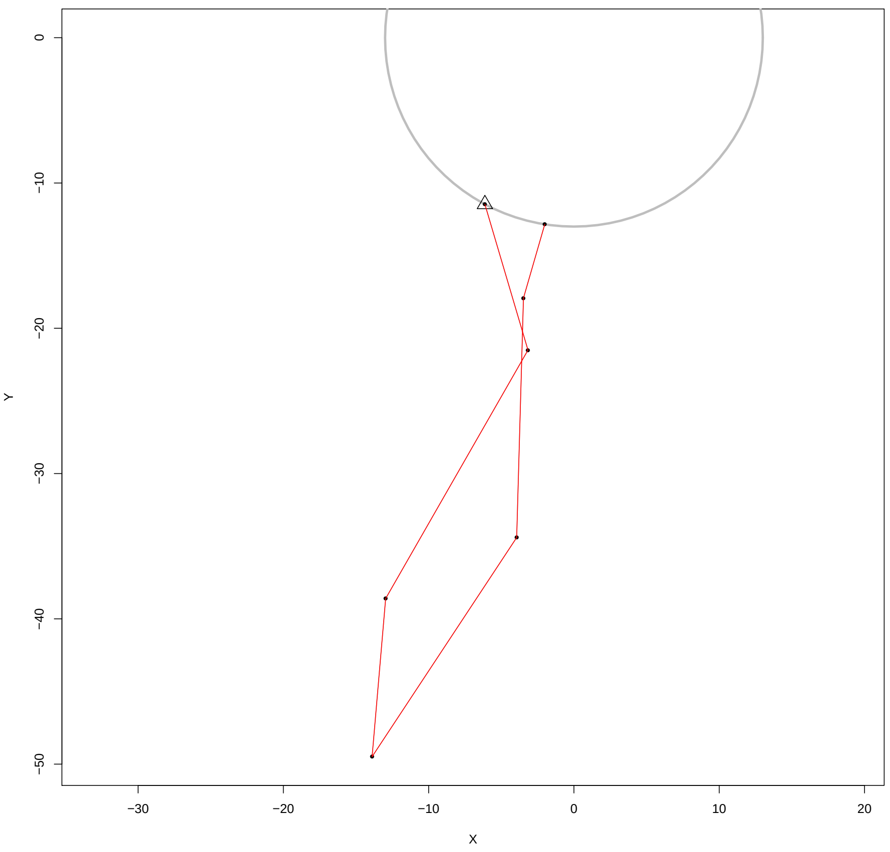

Fig SI-V-14.11 : Loop 92 — loop 11 of bee JT-2

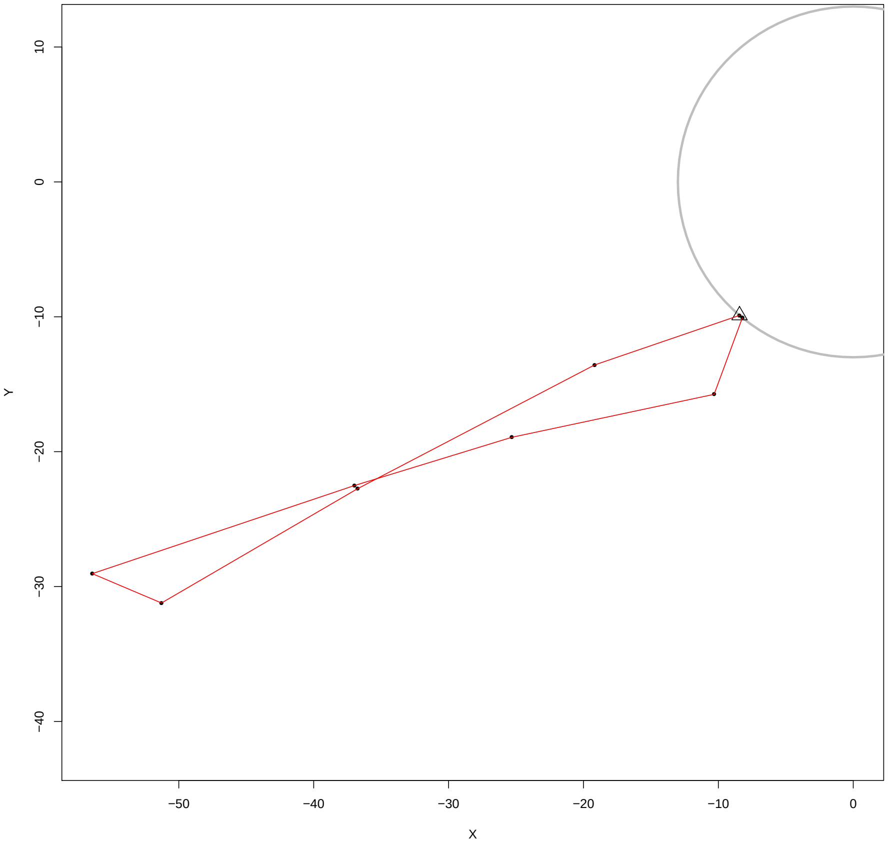

Fig SI-V-14.12 : Loop 93 — loop 12 of bee JT-2

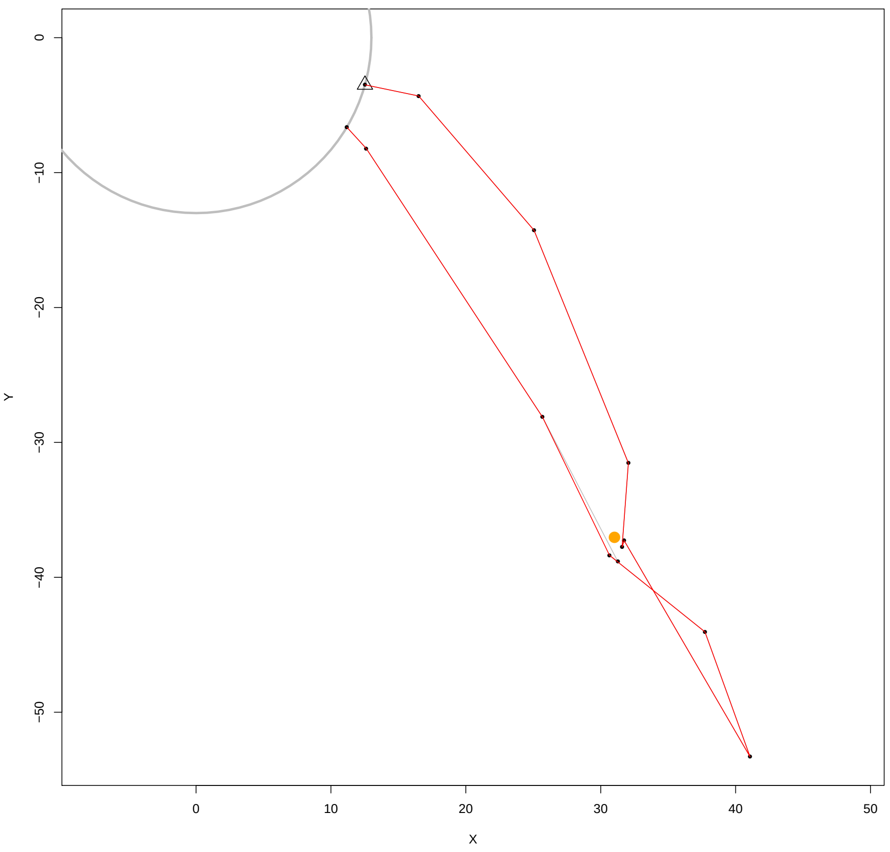

Fig SI-V-14.13 : Loop 94 — loop 13 of bee JT-2

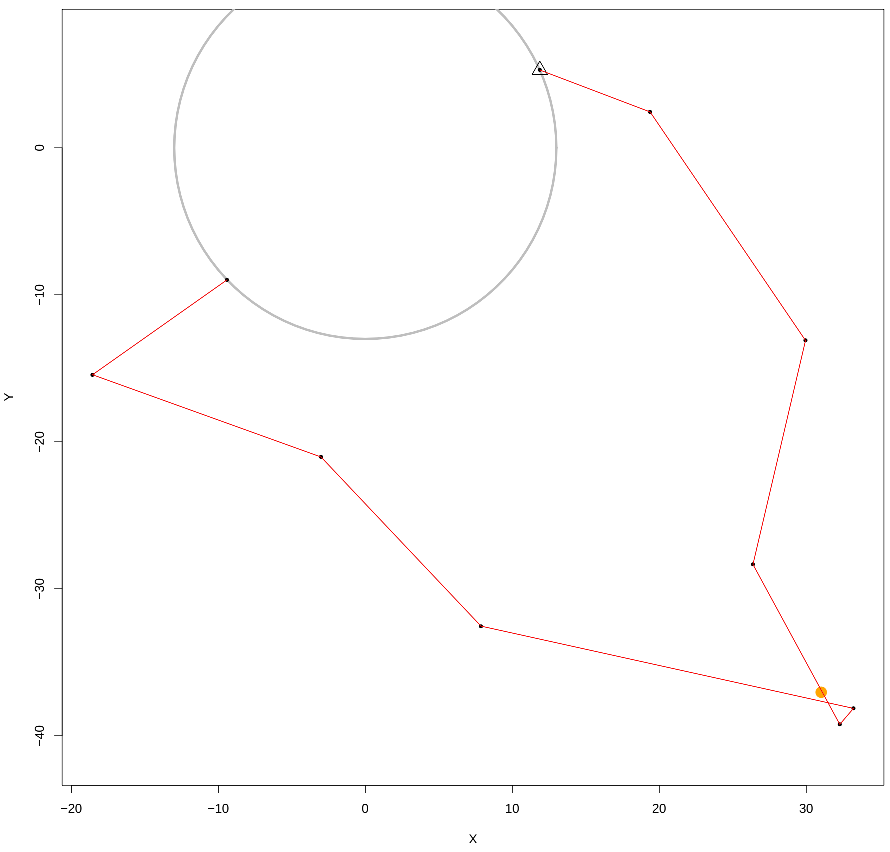

Fig SI-V-14.14 : Loop 95 — loop 14 of bee JT-2

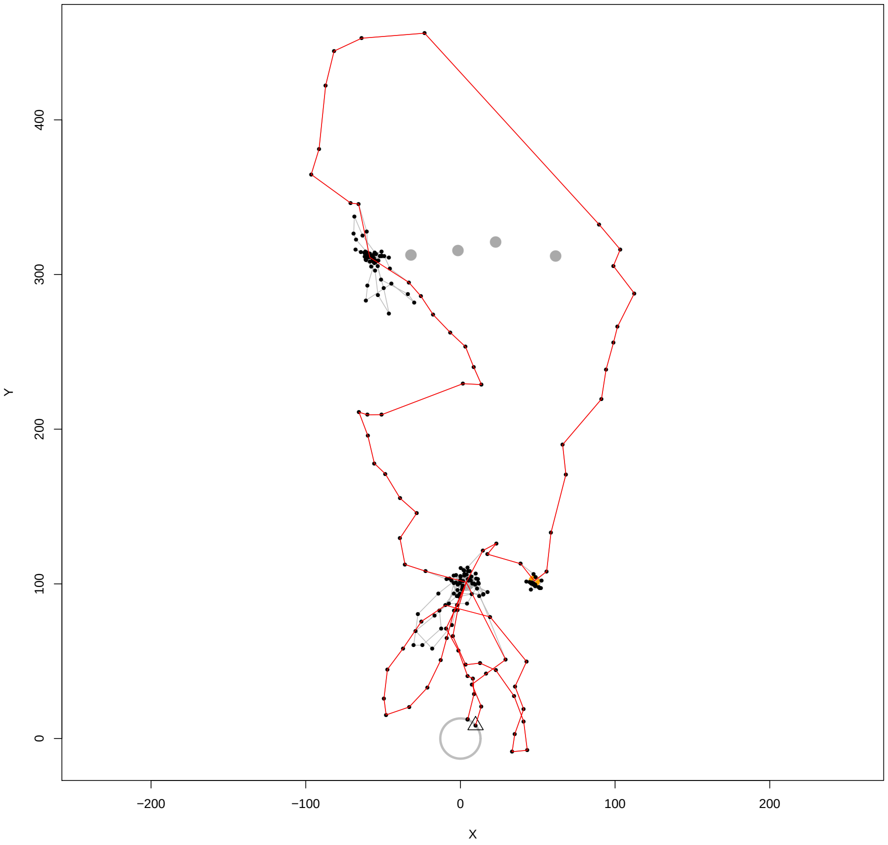

Fig SI-V-14.15 : Loop 96 — loop 15 of bee JT-2

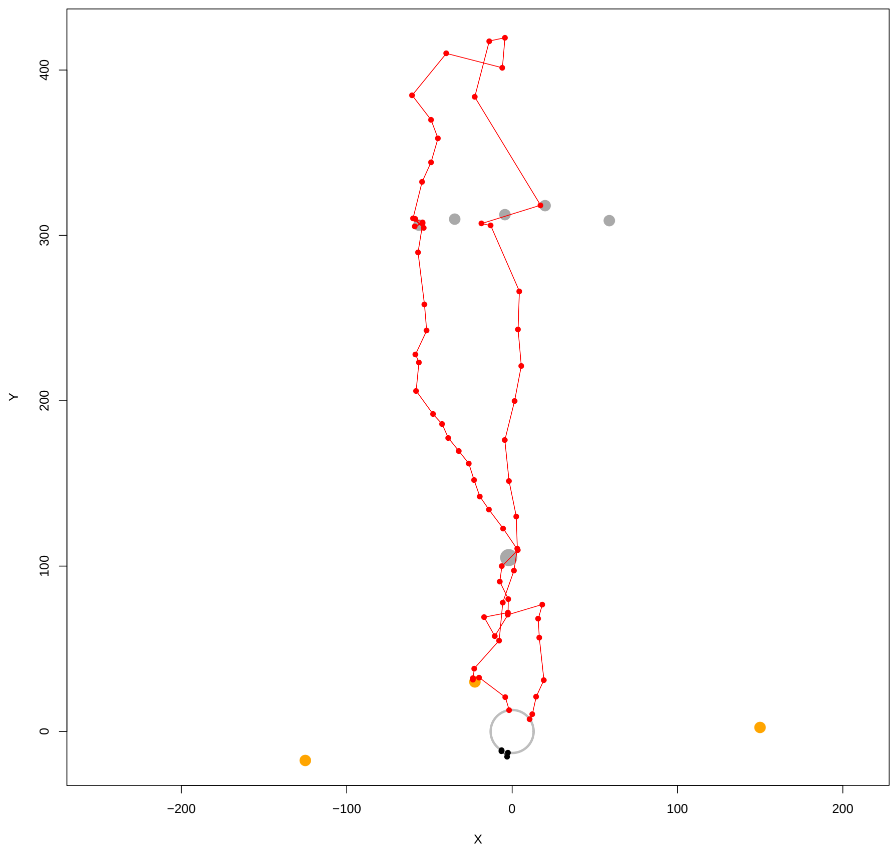

Fig SI-V-15 : Tracks of bee JT-3

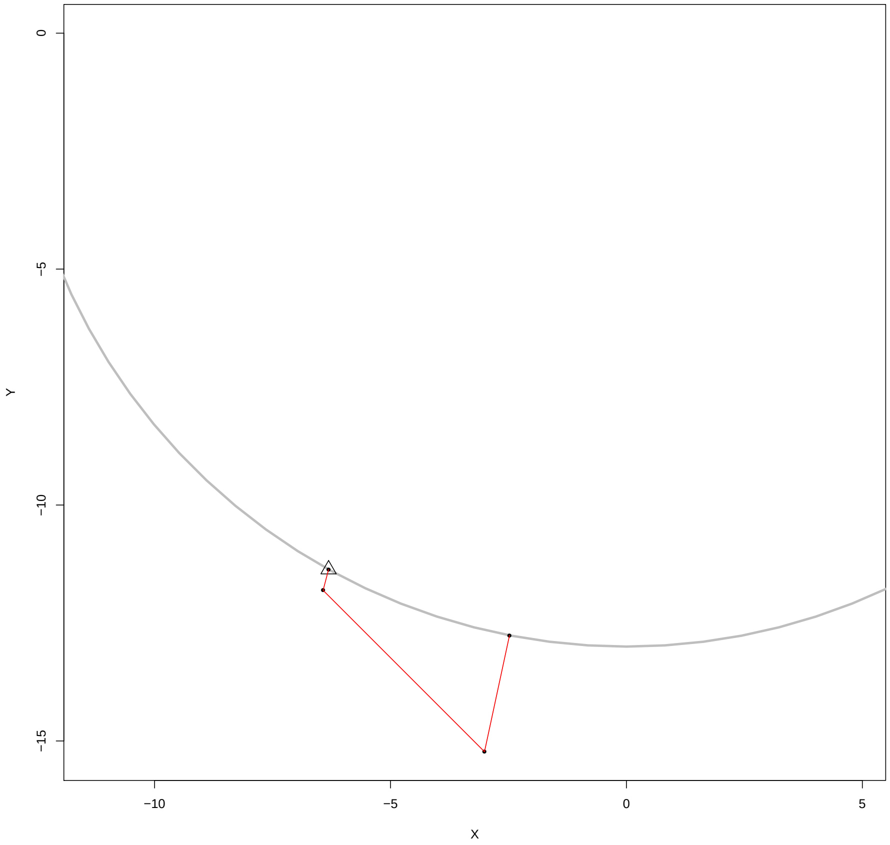

Fig SI-V-15.1 : Loop 97 — loop 1 of bee JT-3

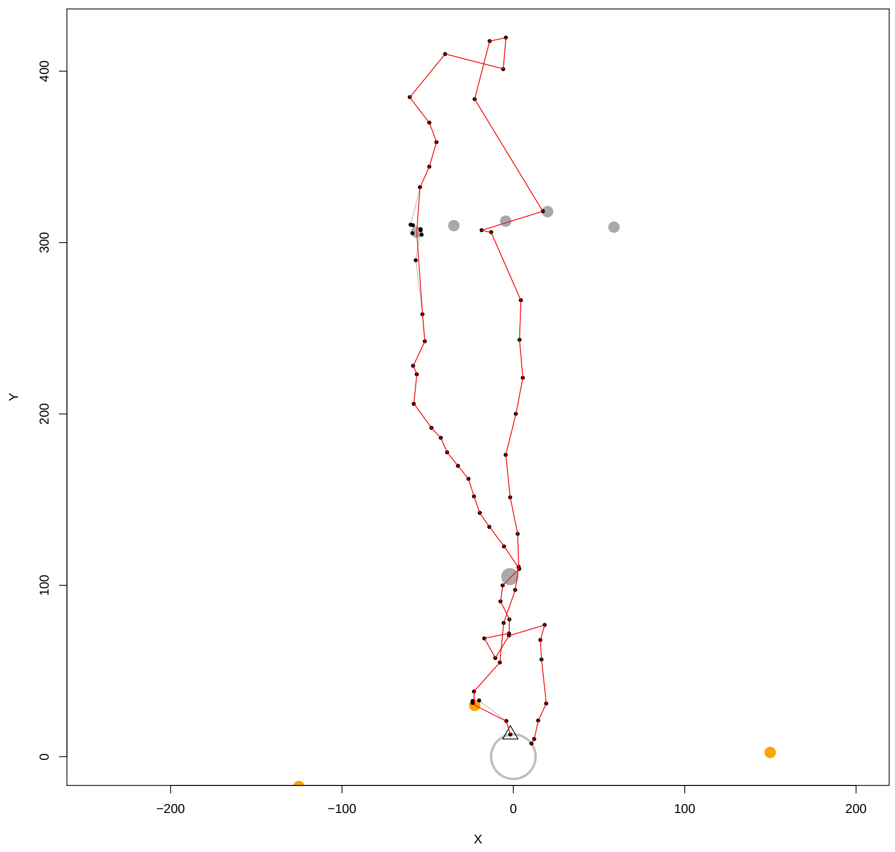

Fig SI-V-15.2 : Loop 98 — loop 2 of bee JT-3

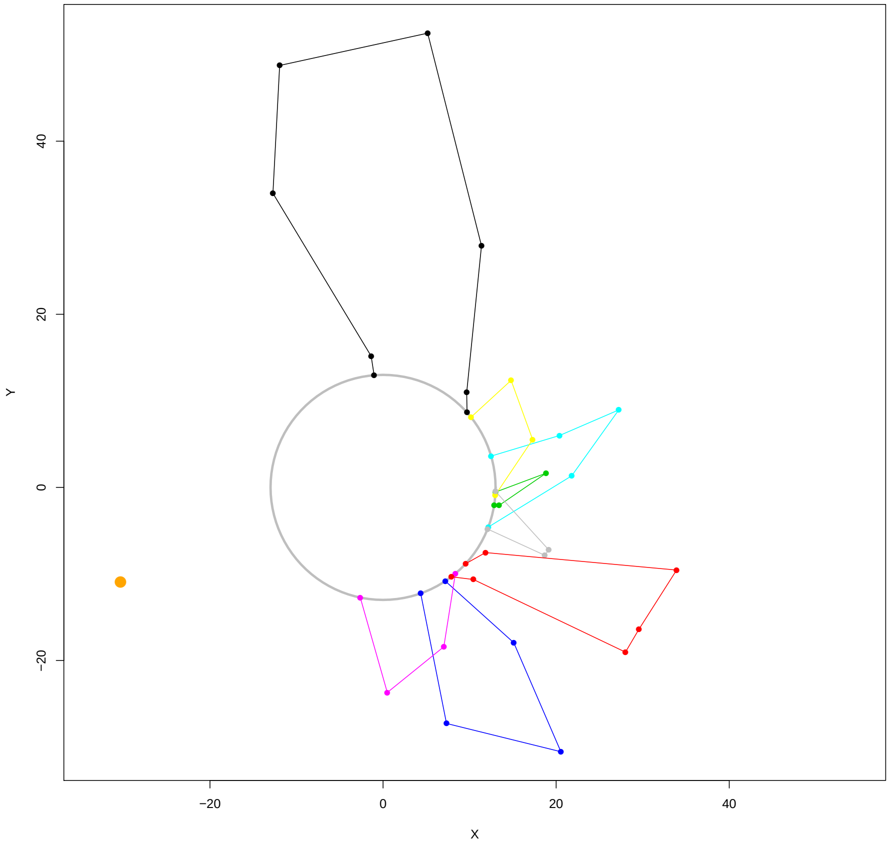

Fig SI-V-16 : Tracks of bee KC-2

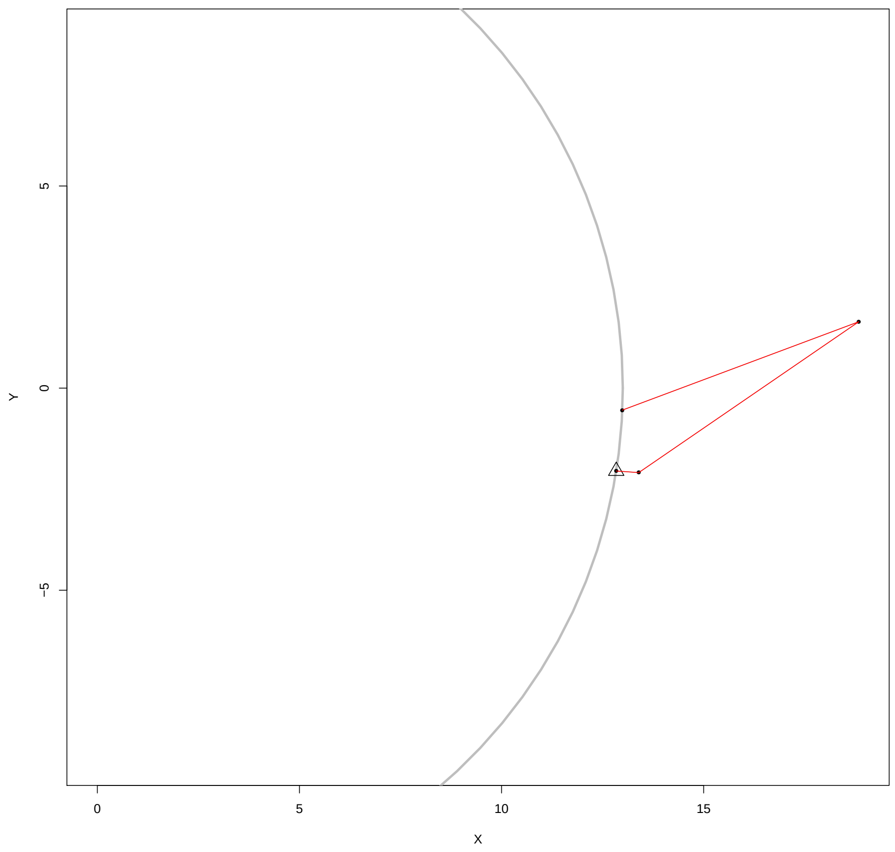

Fig SI-V-16.1 : Loop 99 — loop 1 of bee KC-2

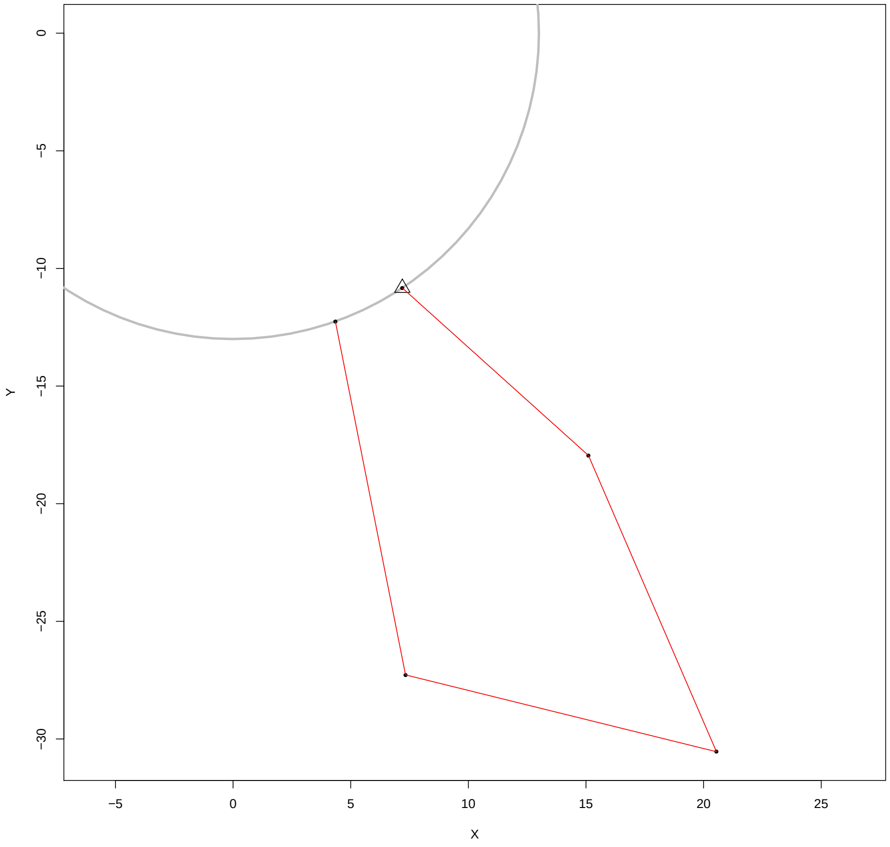

Fig SI-V-16.2 : Loop 100 — loop 2 of bee KC-2

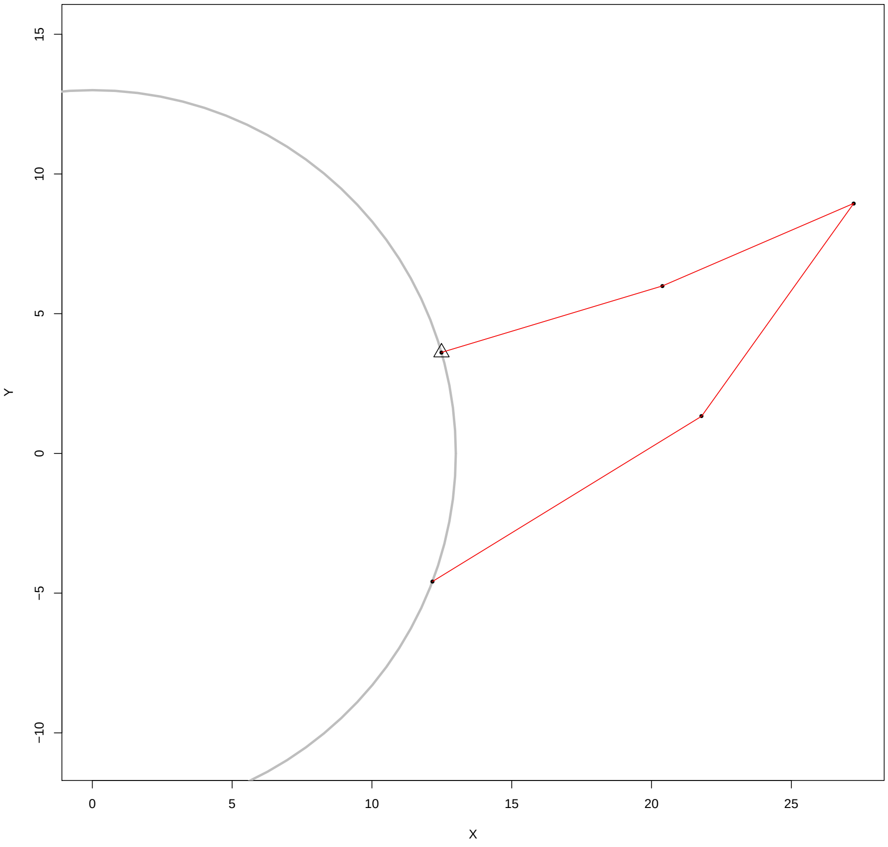

Fig SI-V-16.3 : Loop 101 — loop 3 of bee KC-2

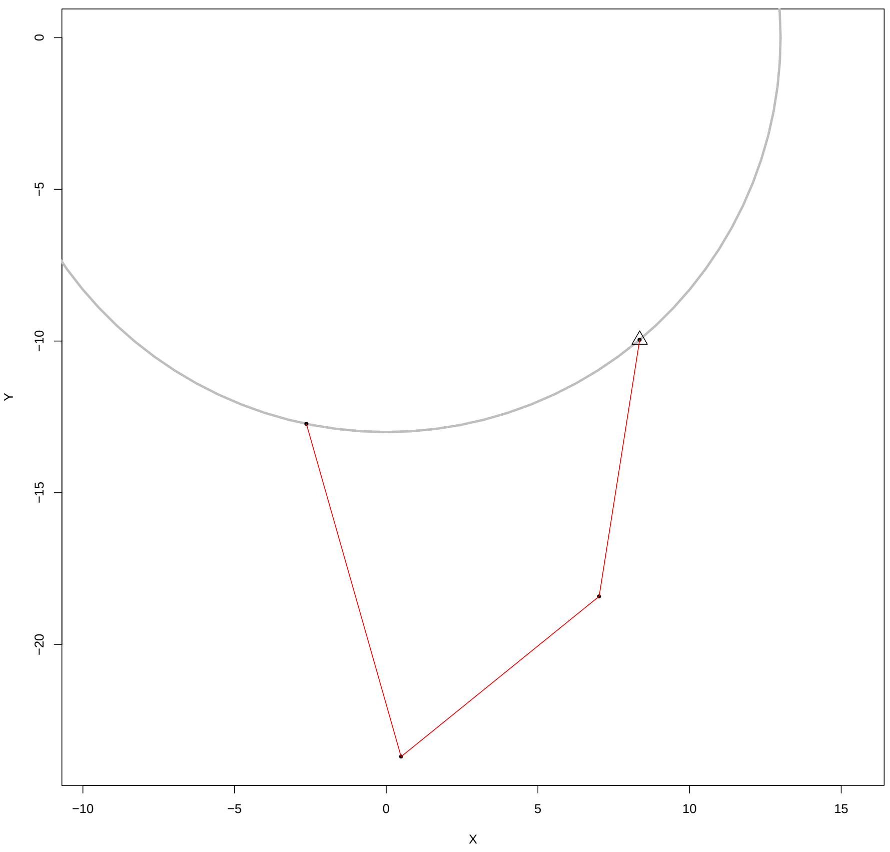

Fig SI-V-16.4 : Loop 102 — loop 4 of bee KC-2

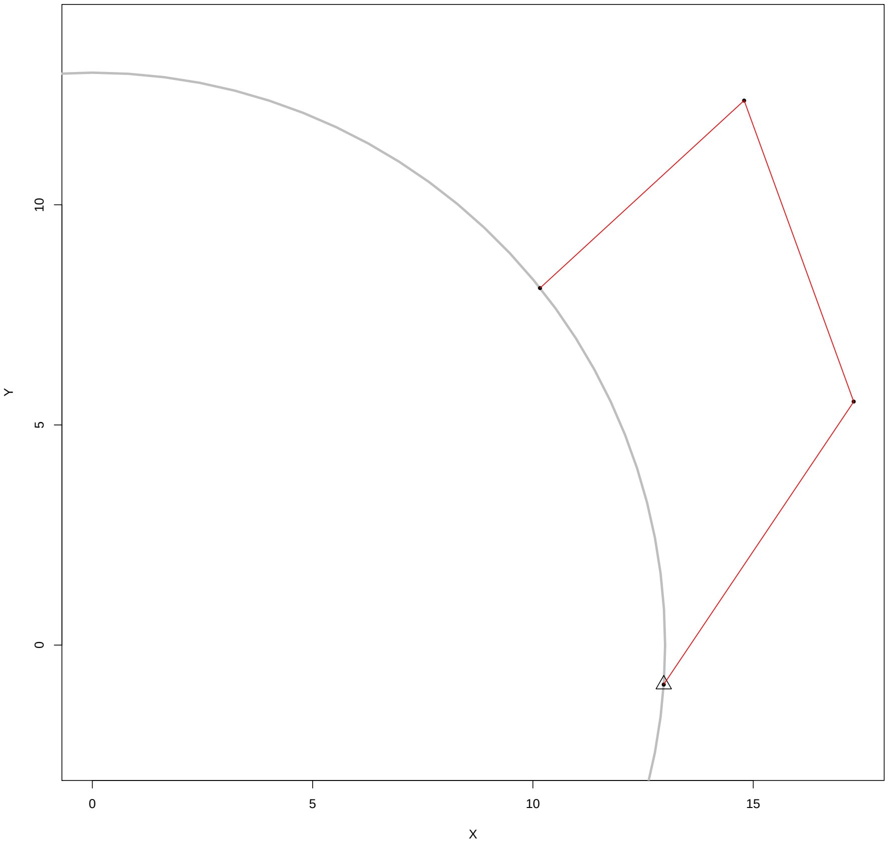

Fig SI-V-16.5 : Loop 103 — loop 5 of bee KC-2

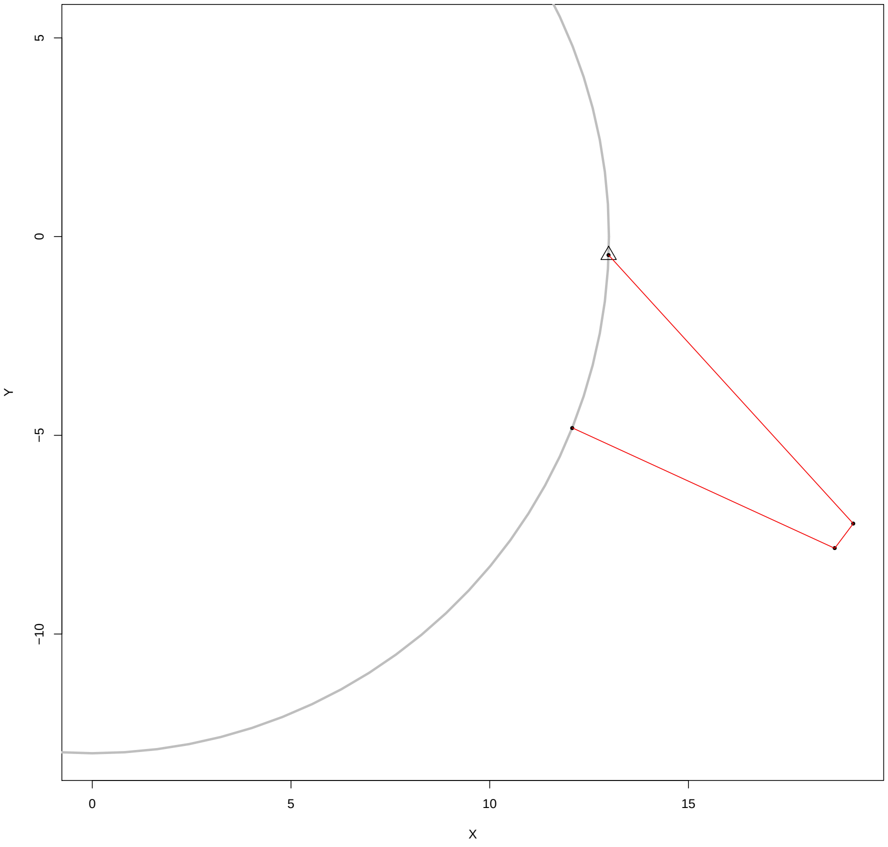

Fig SI-V-16.6 : Loop 104 — loop 6 of bee KC-2

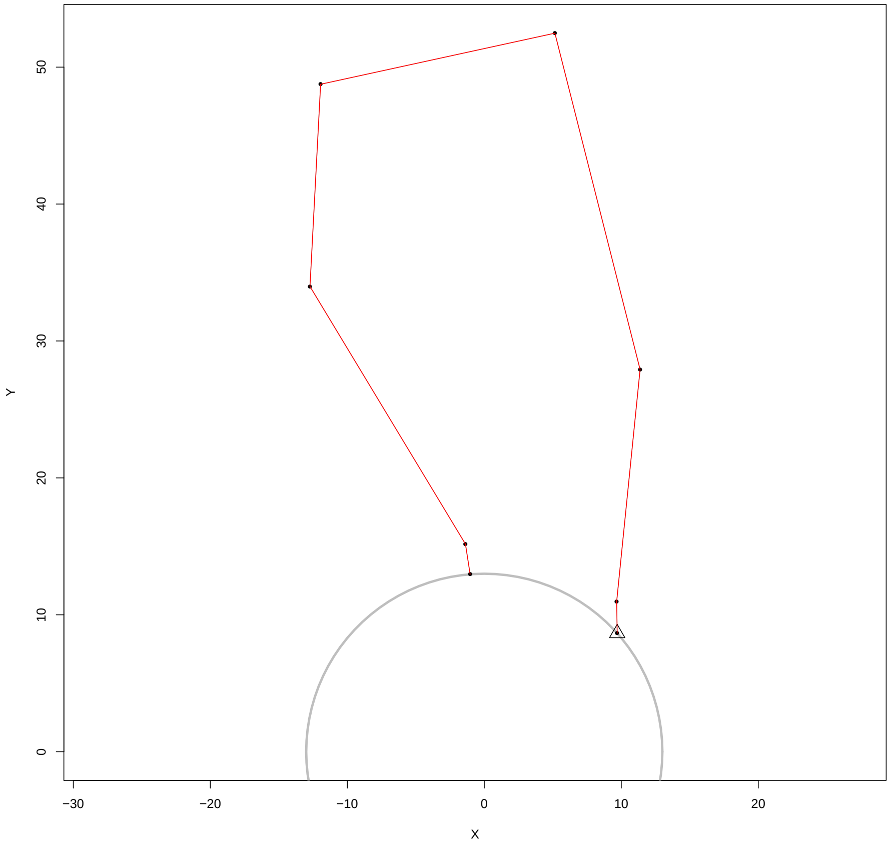

Fig SI-V-16.7 : Loop 105 — loop 7 of bee KC-2

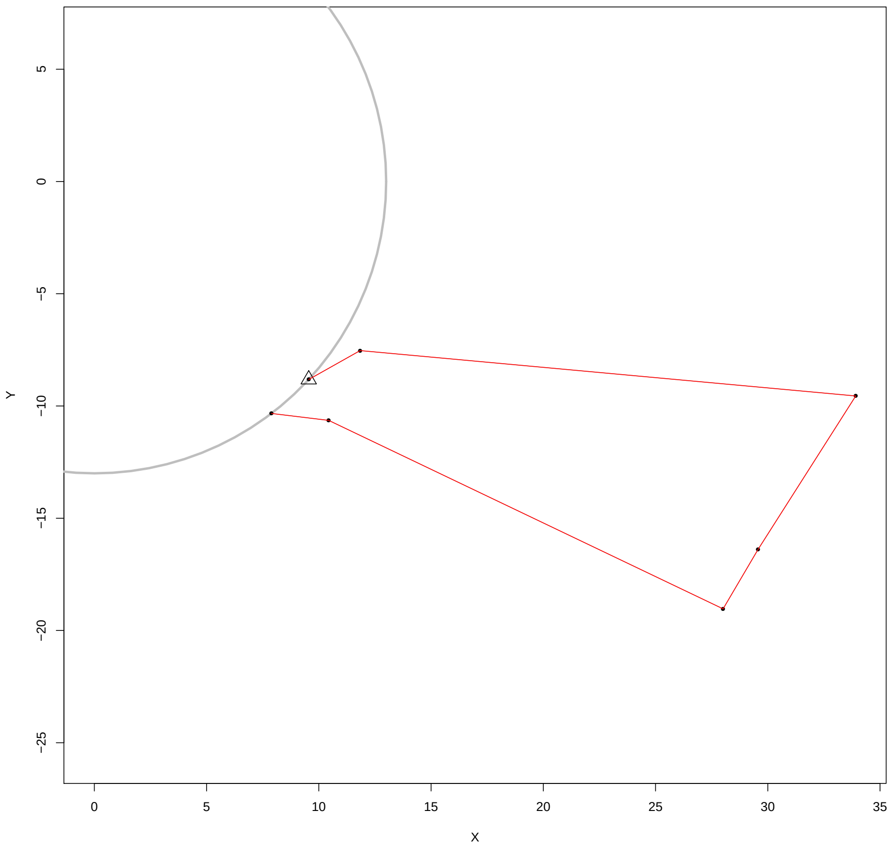

Fig SI-V-16.8 : Loop 106 — loop 8 of bee KC-2

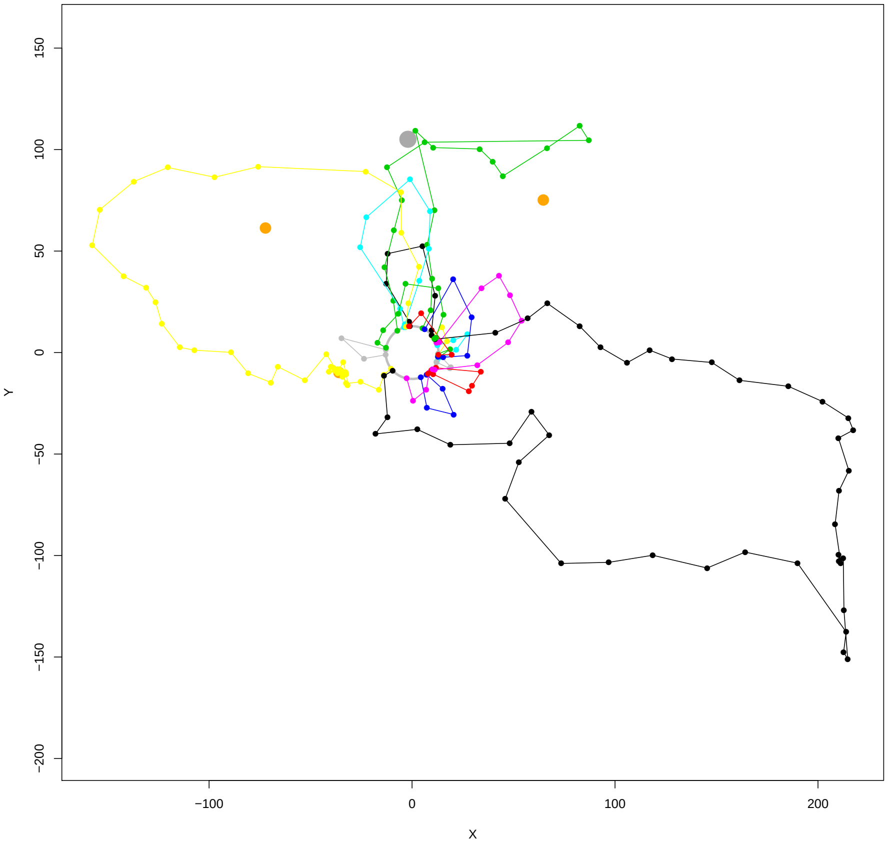

Fig SI-V-17 : Tracks of bee JP-3

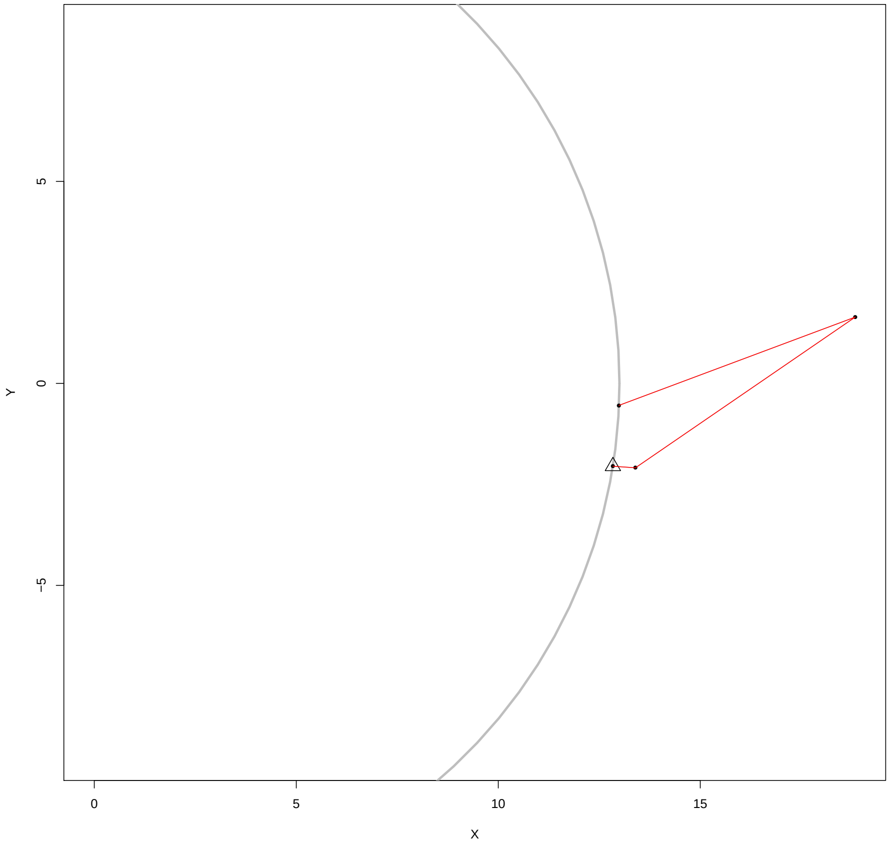

Fig SI-V-17.1 : Loop 107 — loop 1 of bee JP-3

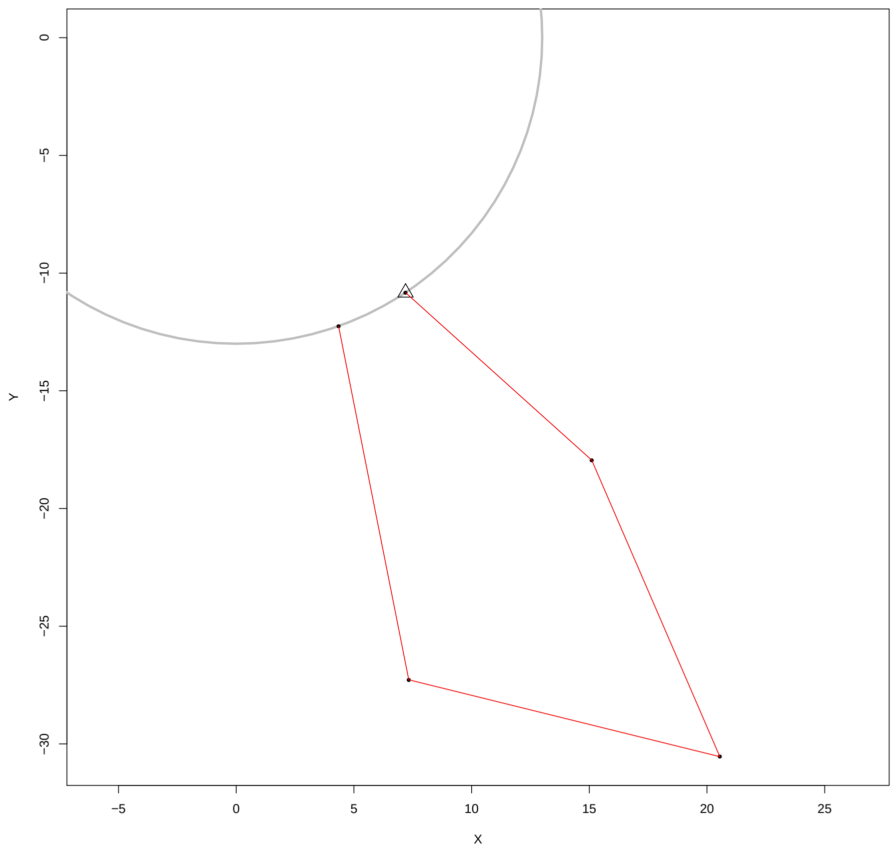

Fig SI-V-17.2 : Loop 108 — loop 2 of bee JP-3

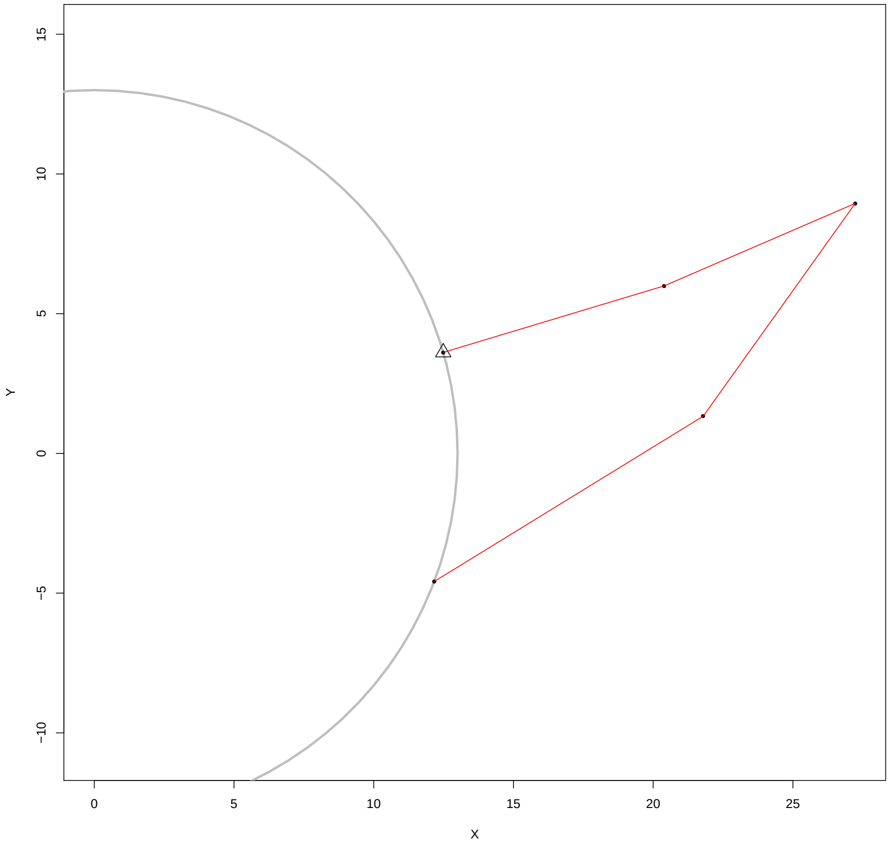

Fig SI-V-17.3 : Loop 109 — loop 3 of bee JP-3

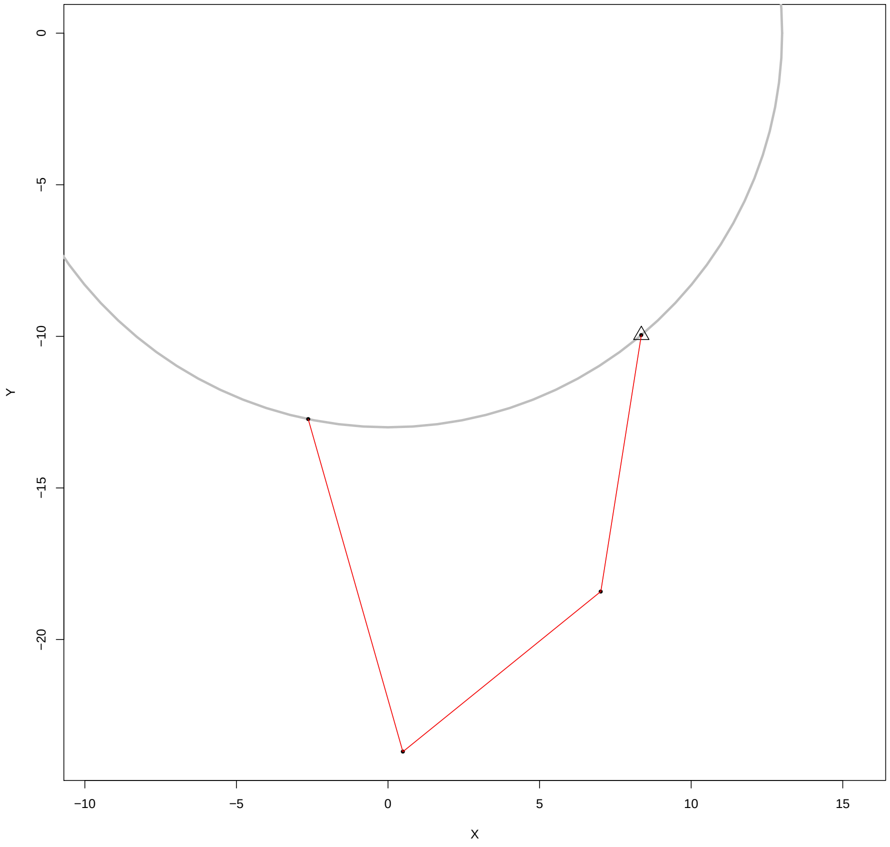

Fig SI-V-17.4 : Loop 110 — loop 4 of bee JP-3

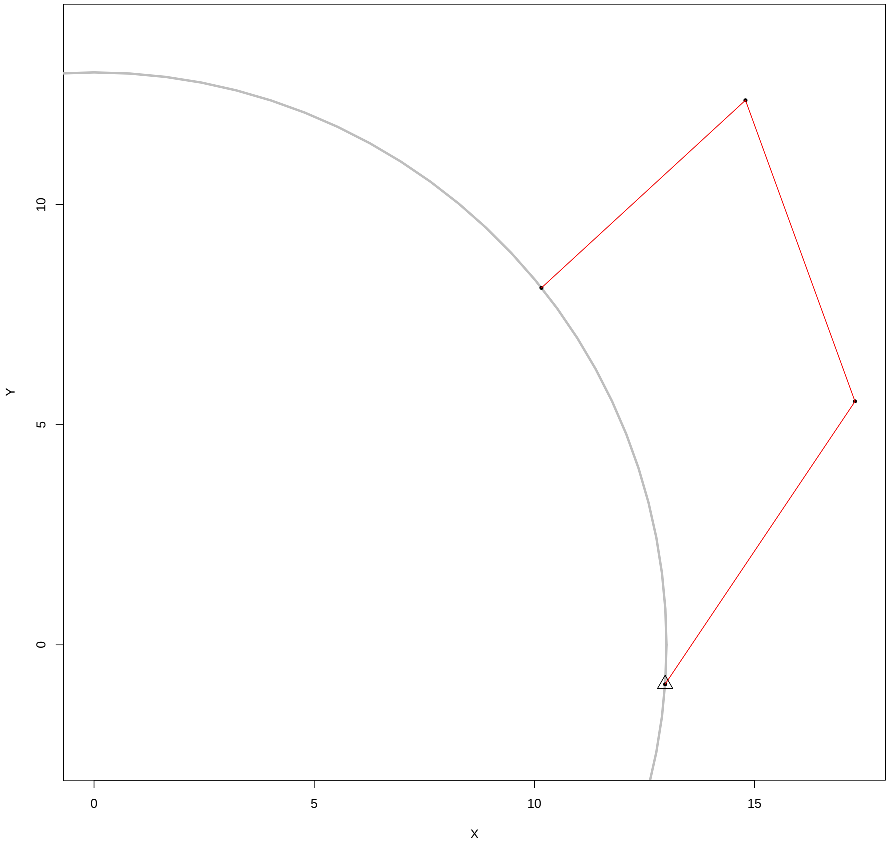

Fig SI-V-17.5 : Loop 111 — loop 5 of bee JP-3

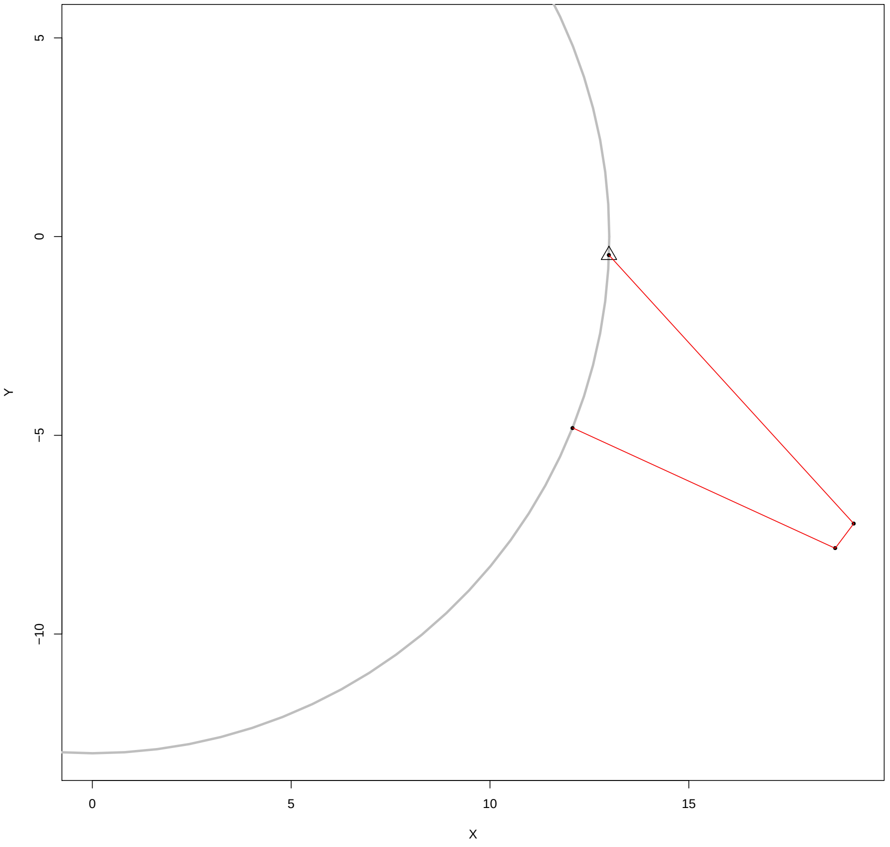

Fig SI-V-17.6 : Loop 112 — loop 6 of bee JP-3

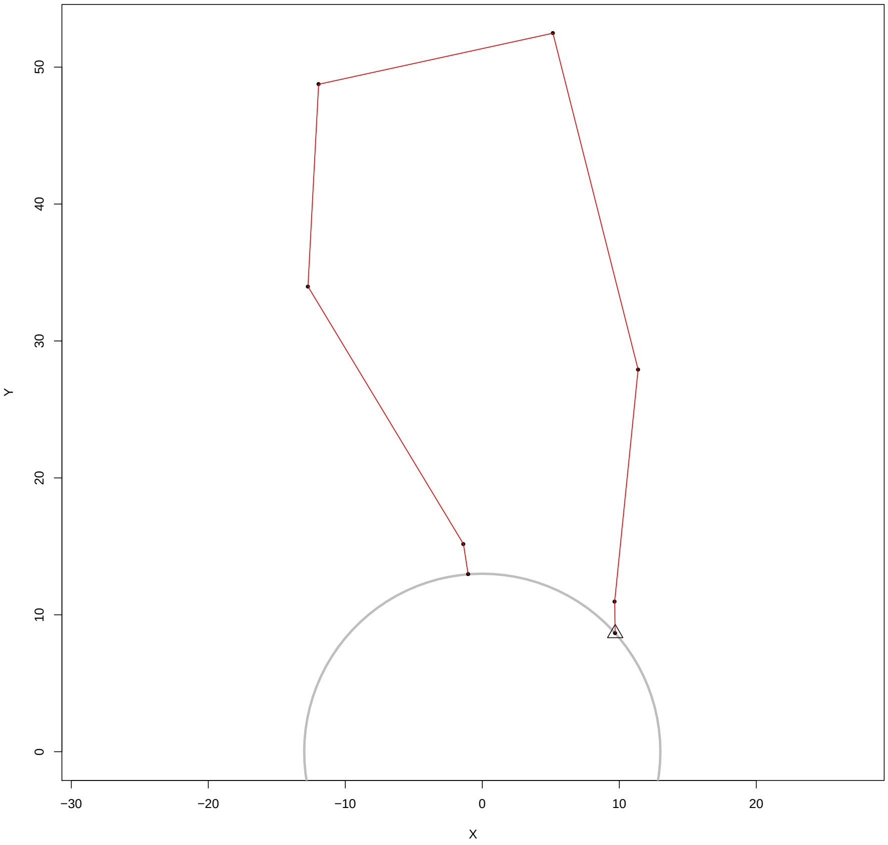

Fig SI-V-17.7 : Loop 113 — loop 7 of bee JP-3

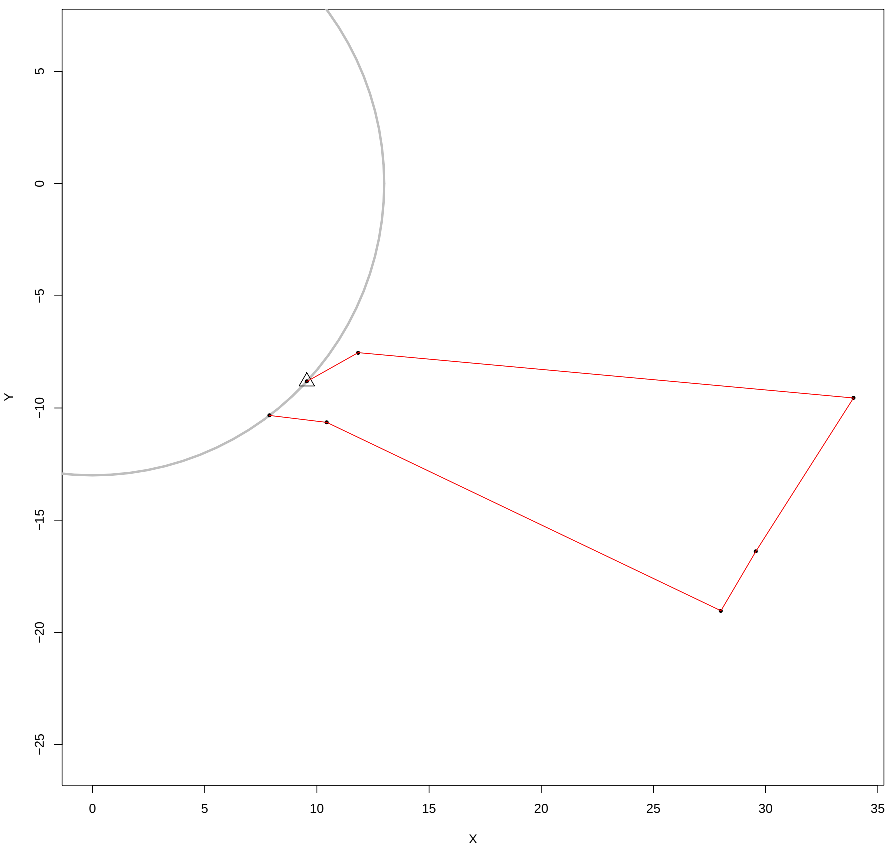

Fig SI-V-17.8 : Loop 114 — loop 8 of bee JP-3

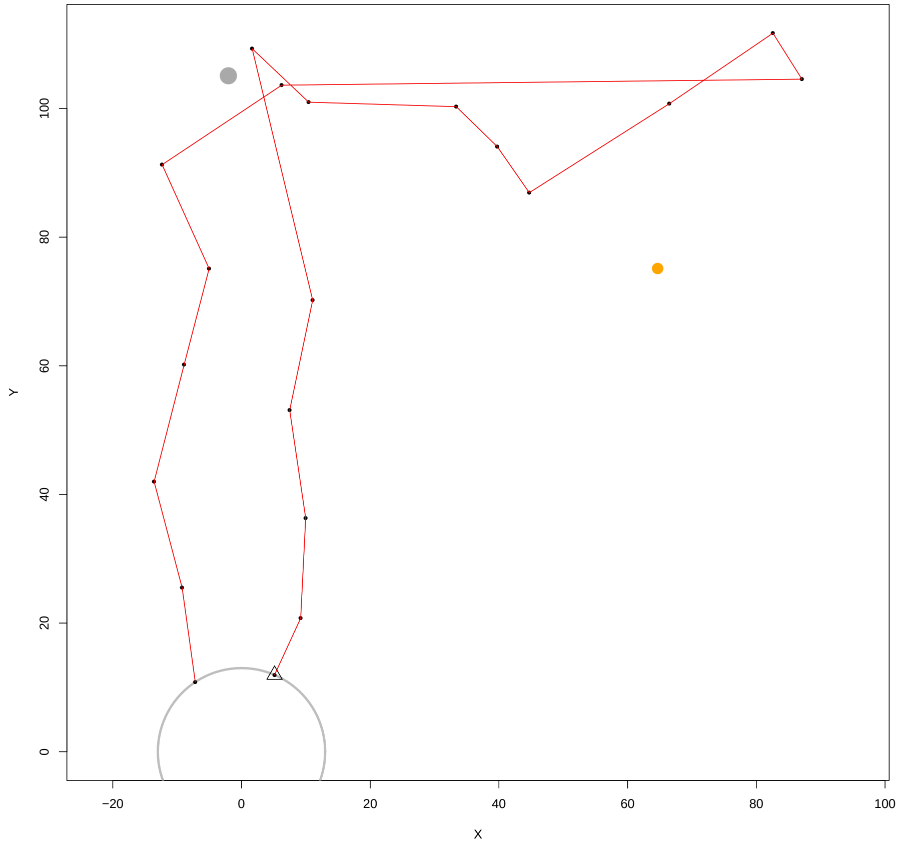

Fig SI-V-17.9 : Loop 115 — loop 9 of bee JP-3

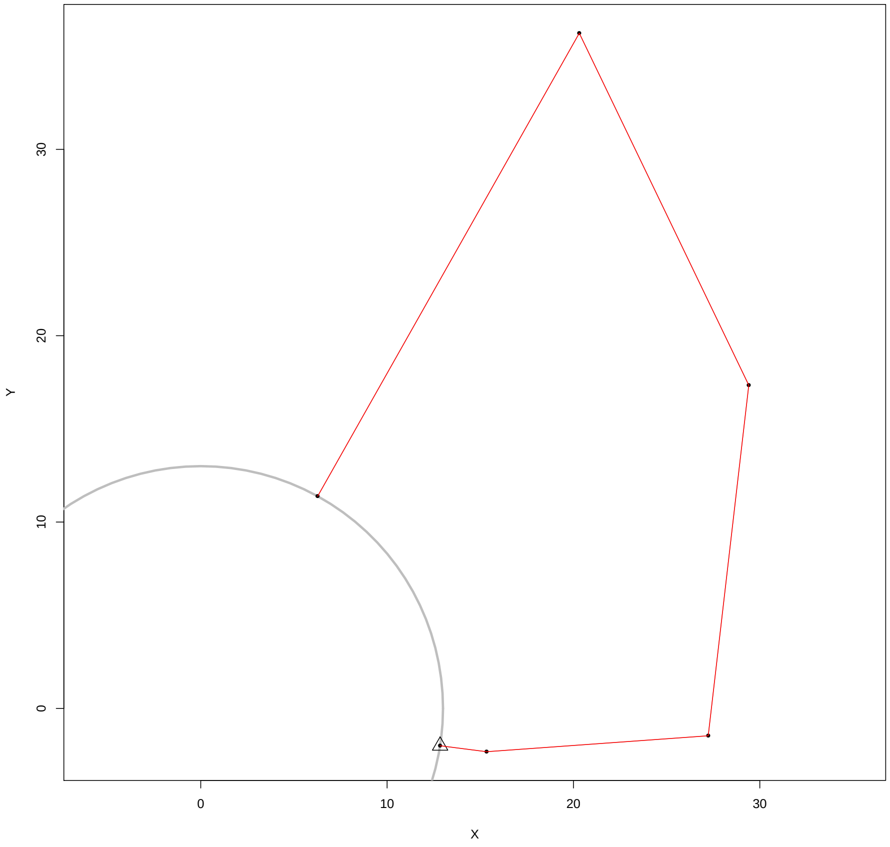

Fig SI-V-17.10 : Loop 116 — loop 10 of bee JP-3

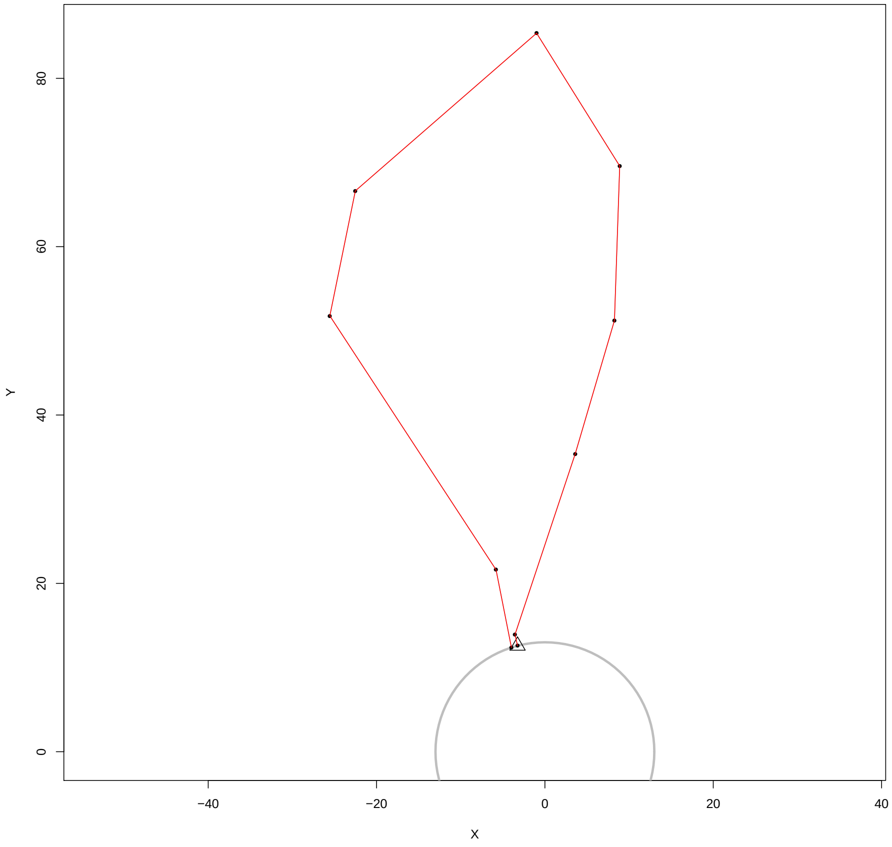

Fig SI-V-17.11 : Loop 117 — loop 11 of bee JP-3

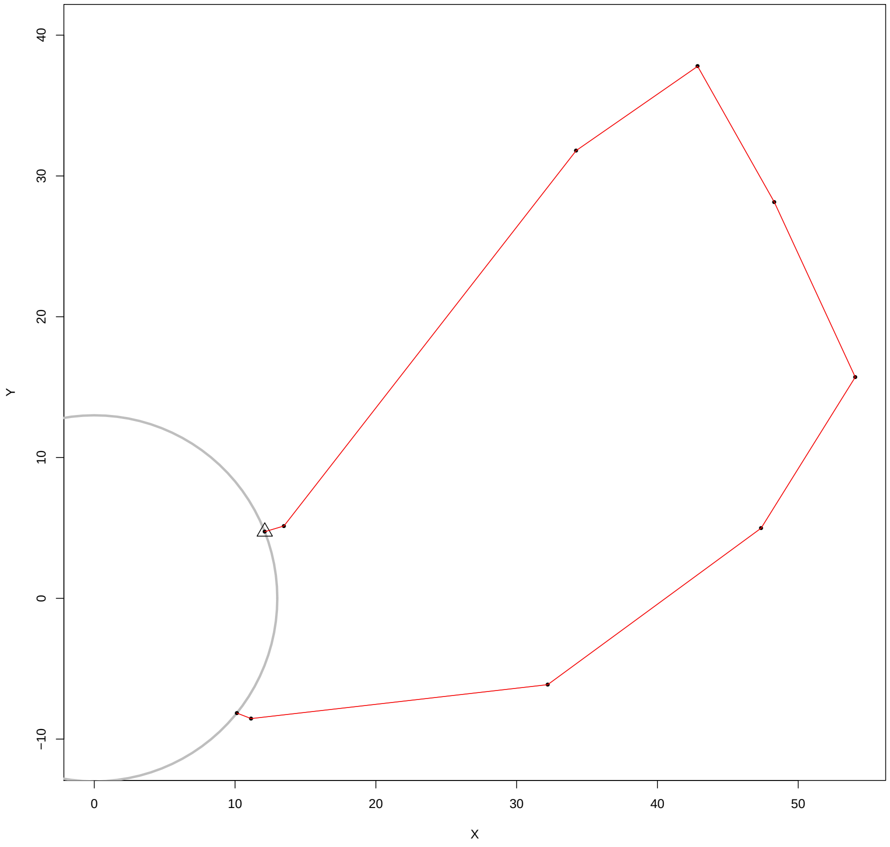

Fig SI-V-17.12 : Loop 118 — loop 12 of bee JP-3

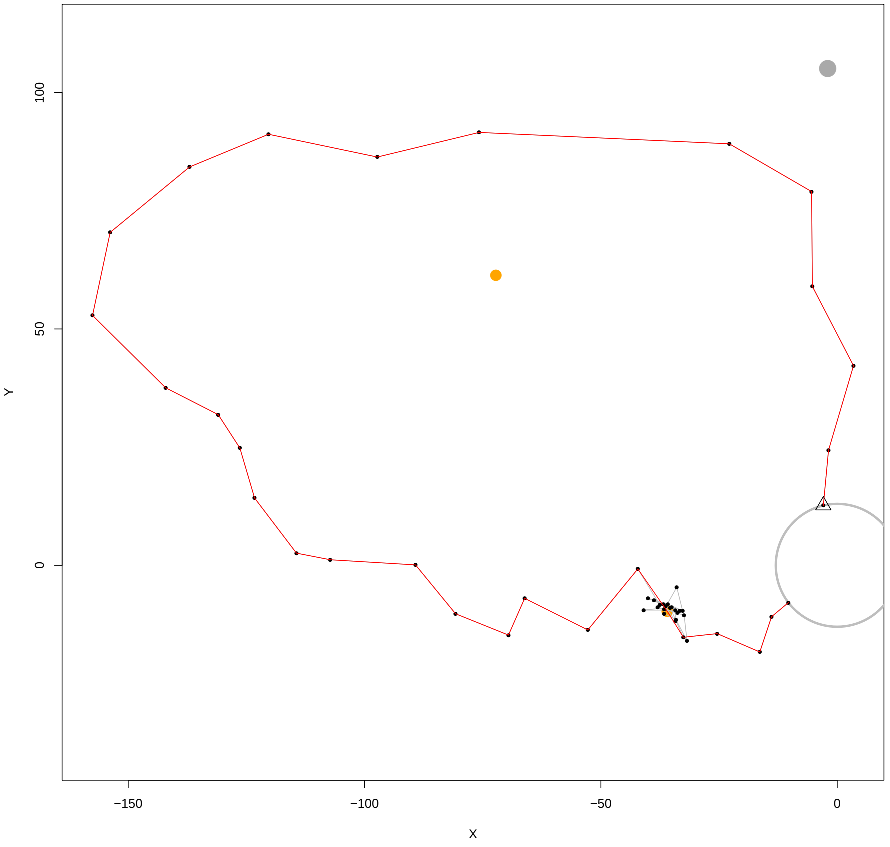

Fig SI-V-17.13 : Loop 119 — loop 13 of bee JP-3

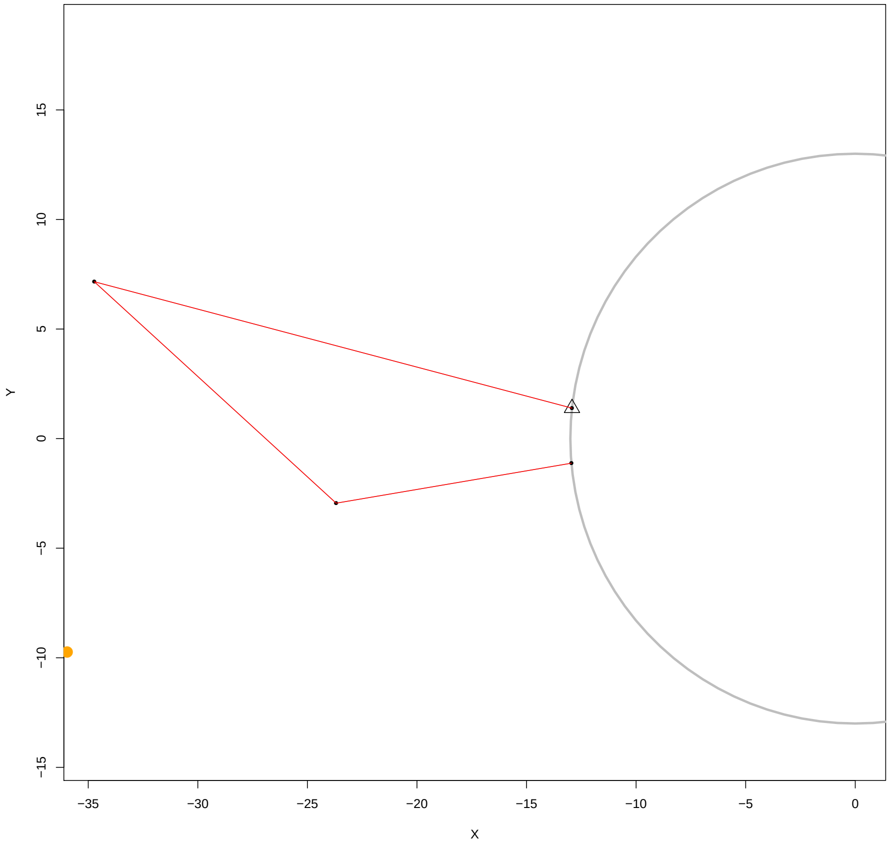

Fig SI-V-17.14 : Loop 120 — loop 14 of bee JP-3

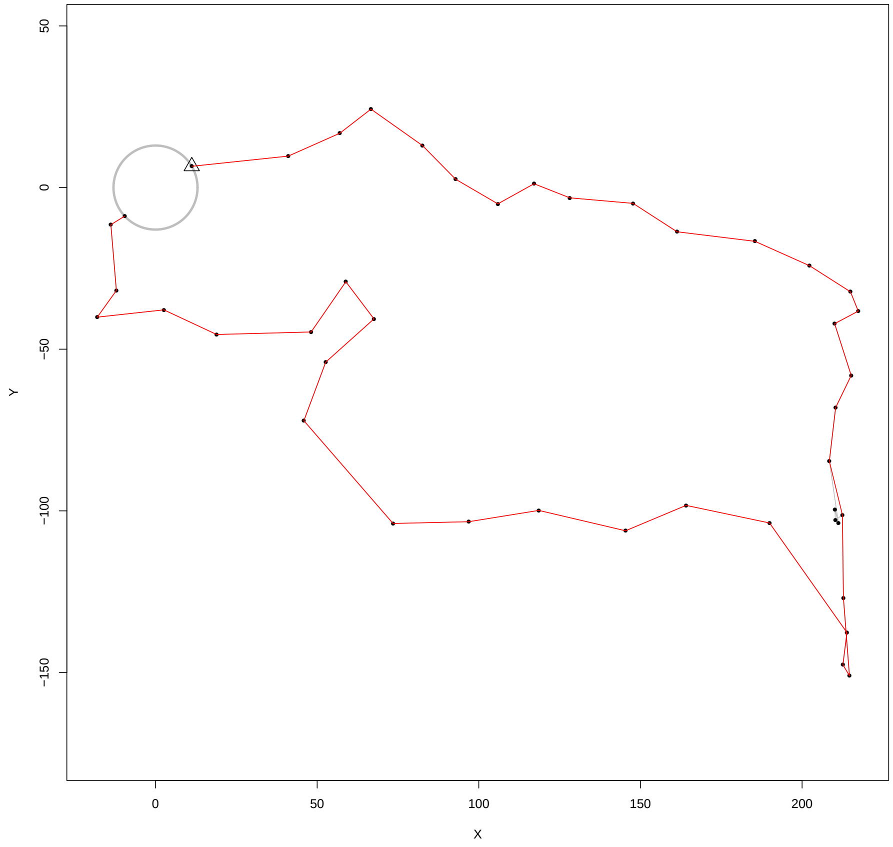

Fig SI-V-17.15 : Loop 121 — loop 15 of bee JP-3

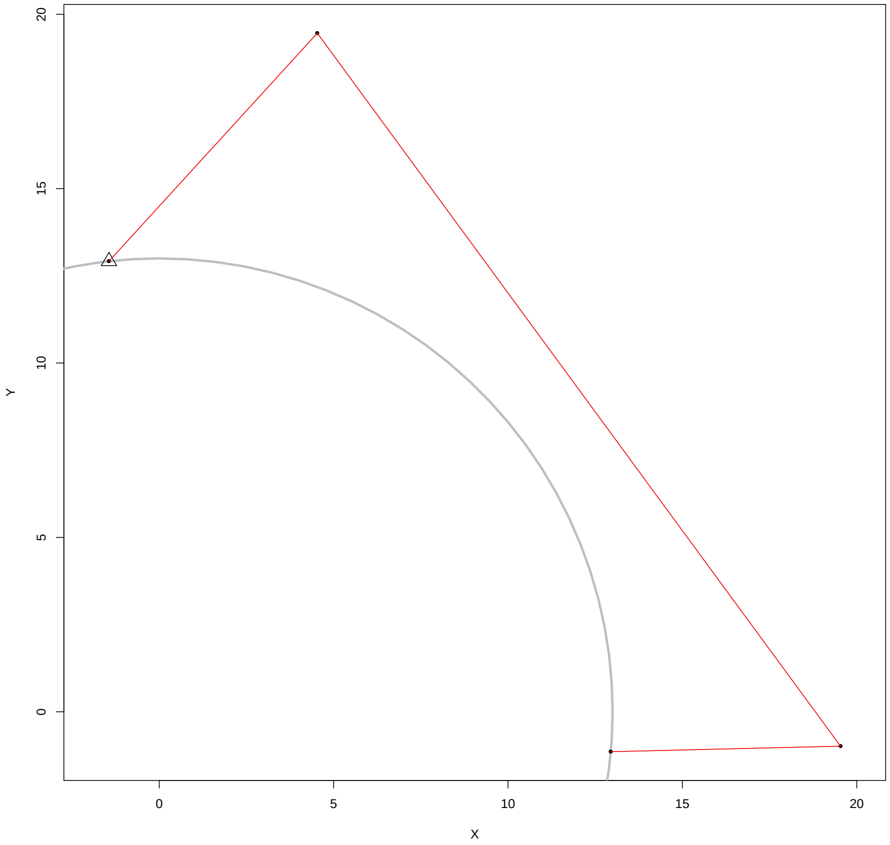

Fig SI-V-17.16 : Loop 122 — loop 16 of bee JP-3

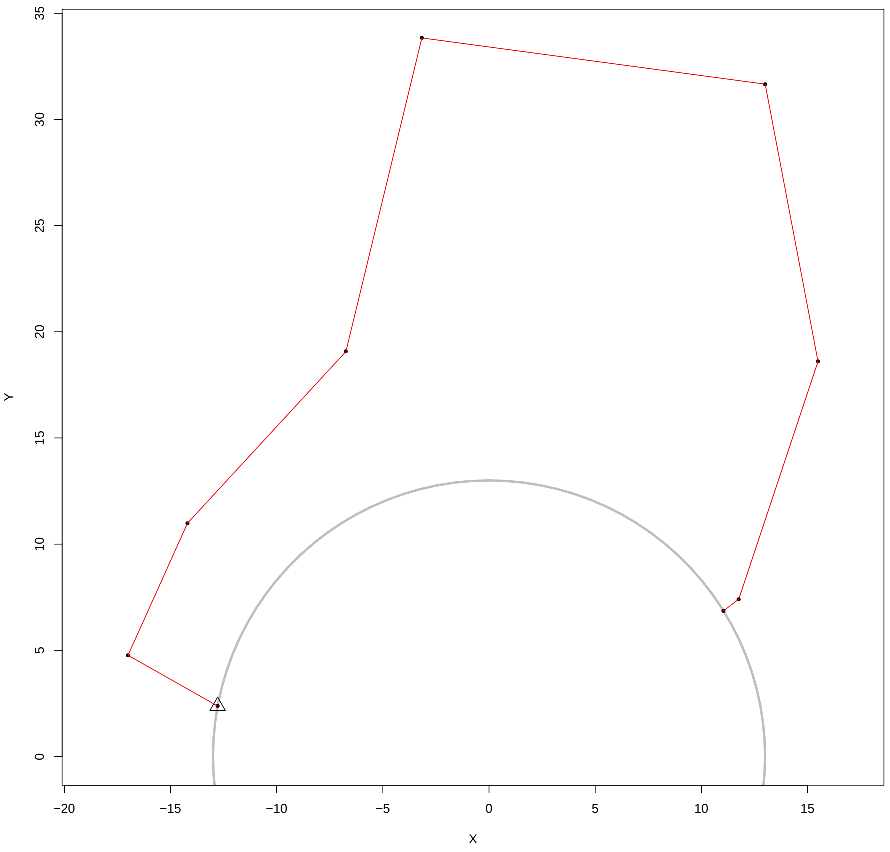

Fig SI-V-17.17 : Loop 123 — loop 17 of bee JP-3

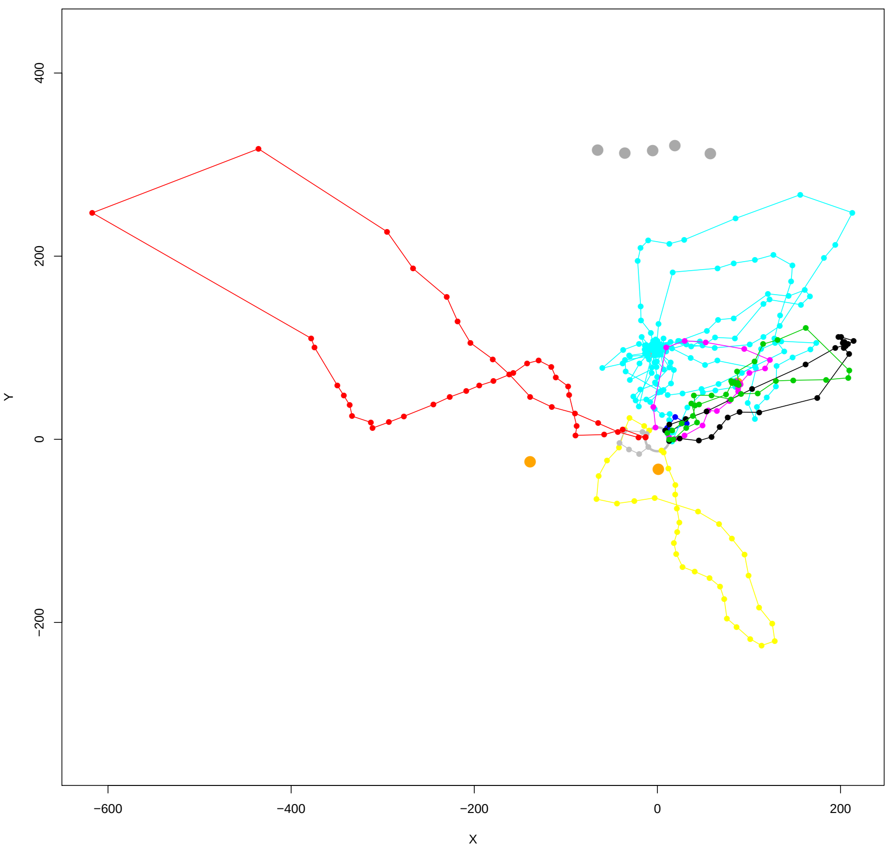

Fig SI-V-18 : Tracks of bee JP-4

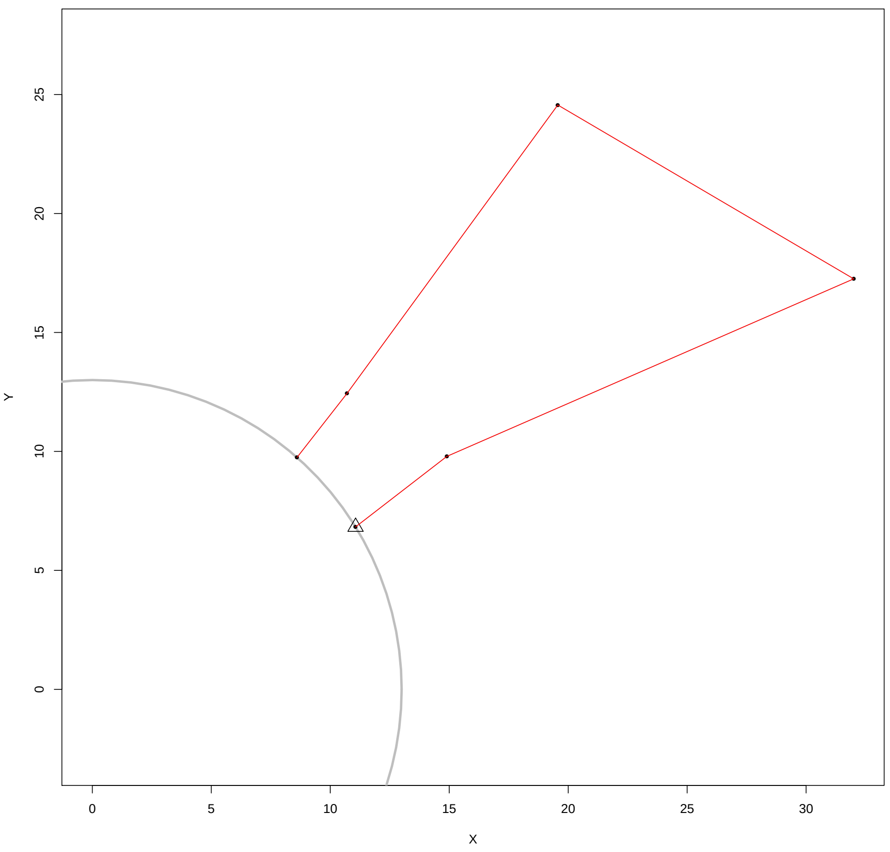

Fig SI-V-18.1 : Loop 124 — loop 1 of bee JP-4

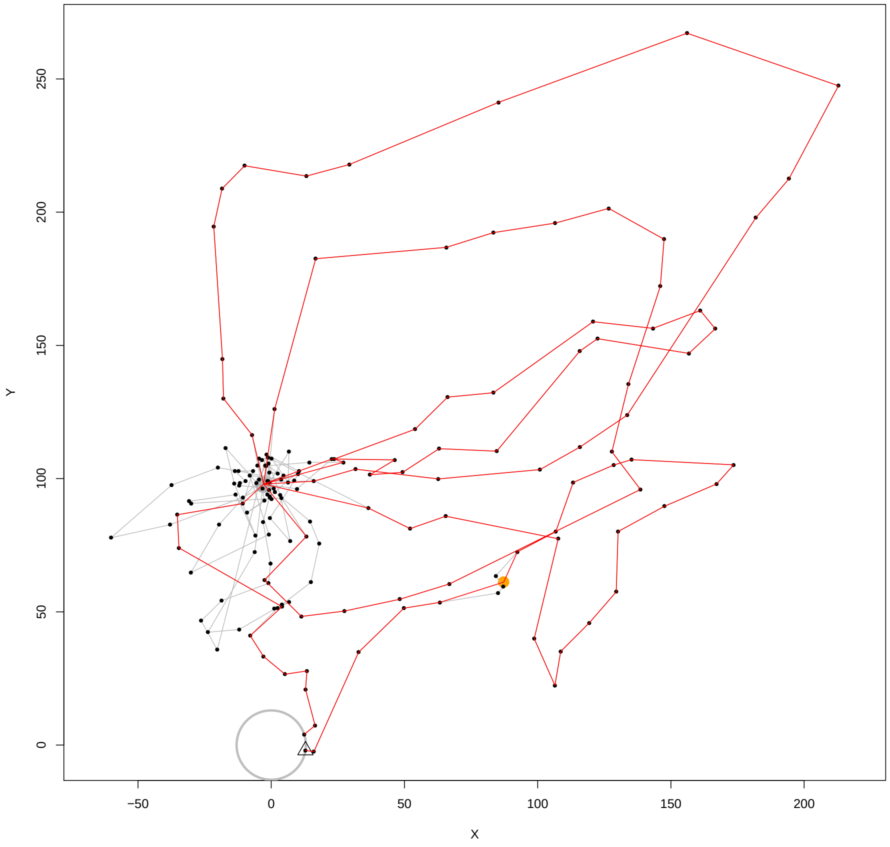

Fig SI-V-18.2 : Loop 125 — loop 2 of bee JP-4

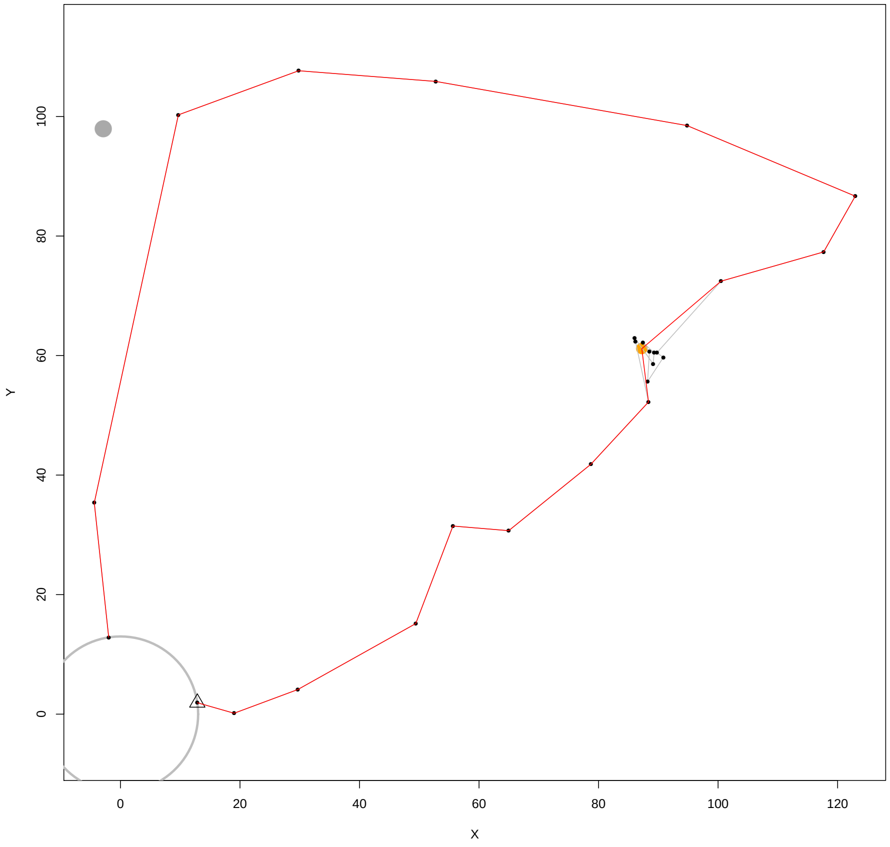

Fig SI-V-18.3 : Loop 126 — loop 3 of bee JP-4

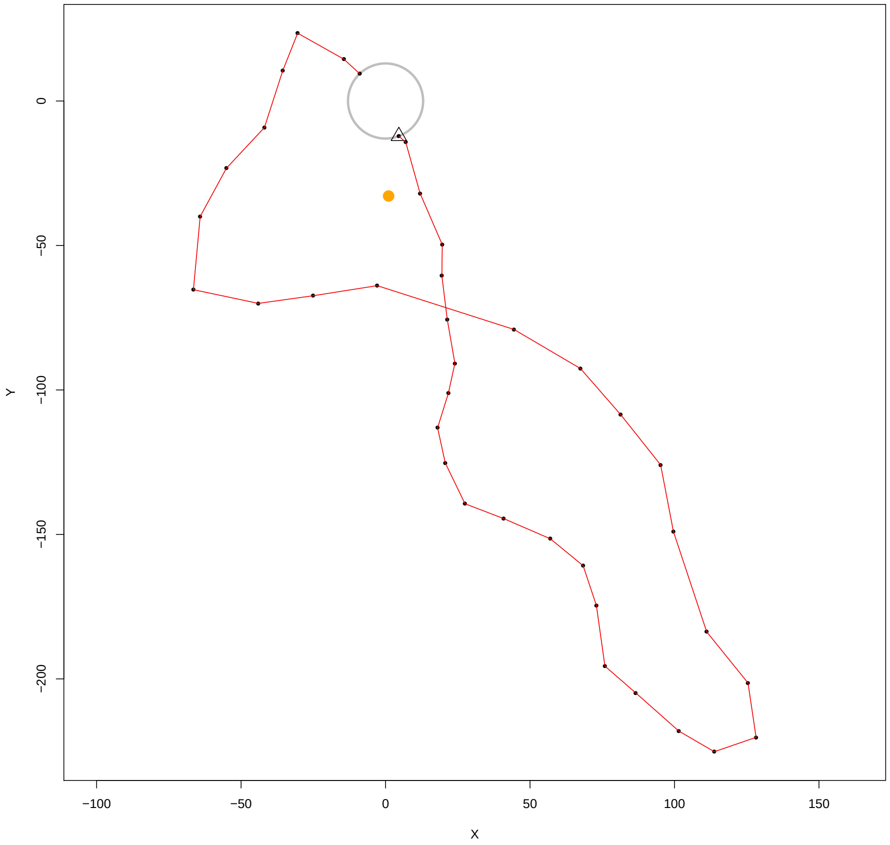

Fig SI-V-18.4 : Loop 127 — loop 4 of bee JP-4

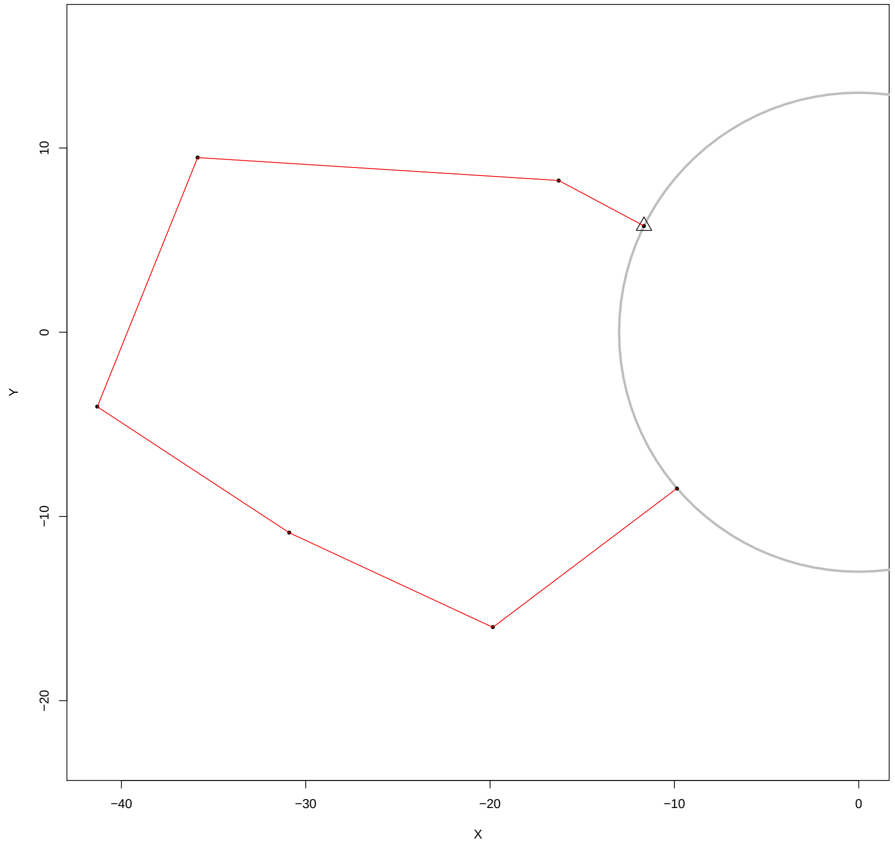

Fig SI-V-18.5 : Loop 128 — loop 5 of bee JP-4

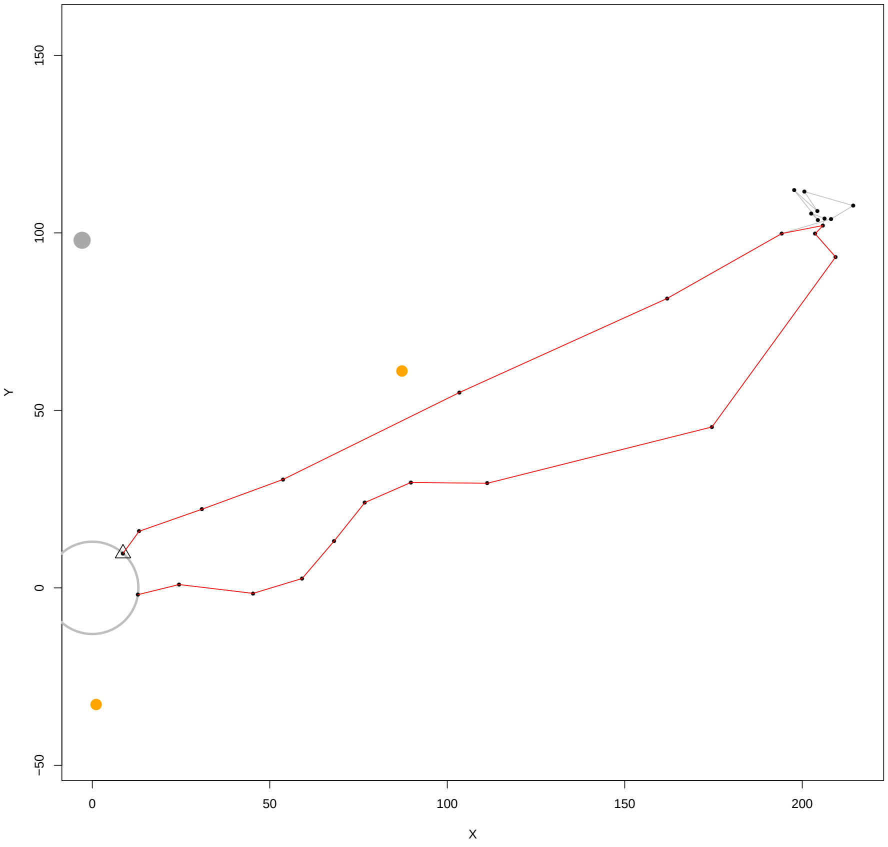

Fig SI-V-18.6 : Loop 129 — loop 6 of bee JP-4

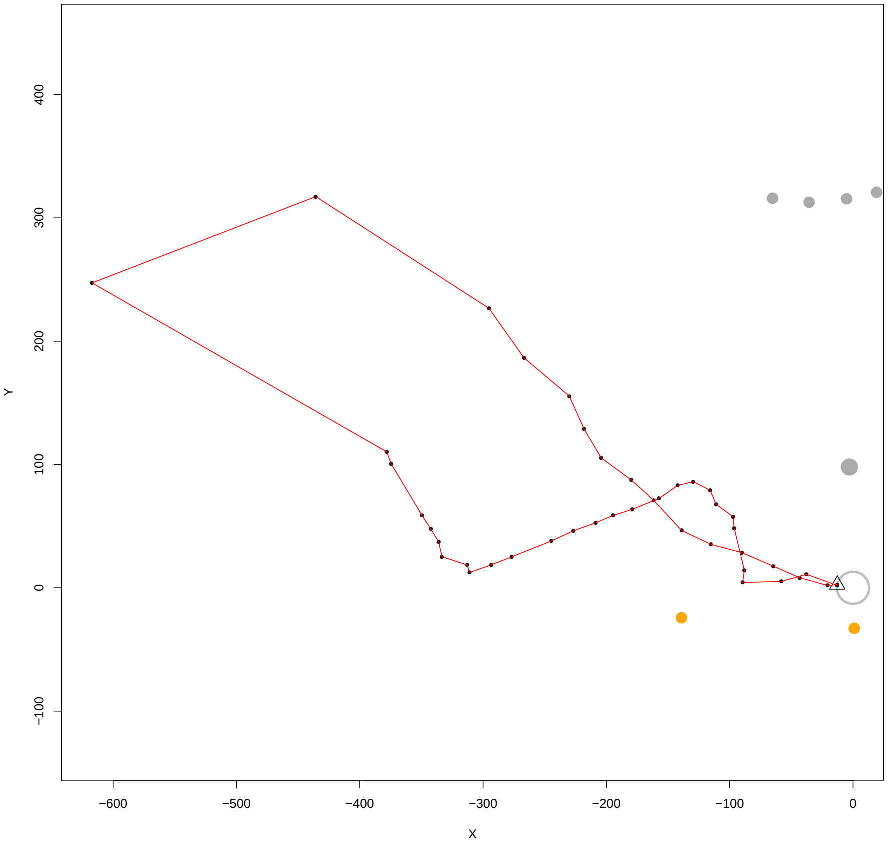

Fig SI-V-18.7 : Loop 130 — loop 7 of bee JP-4

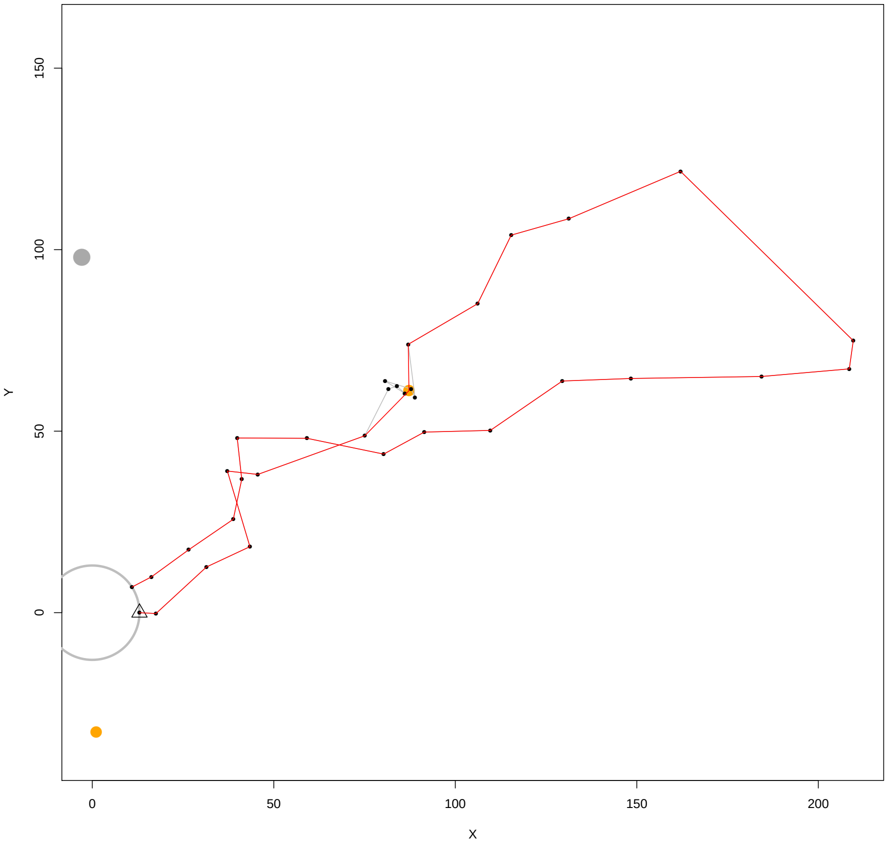

Fig SI-V-18.8 : Loop 131 — loop 8 of bee JP-4

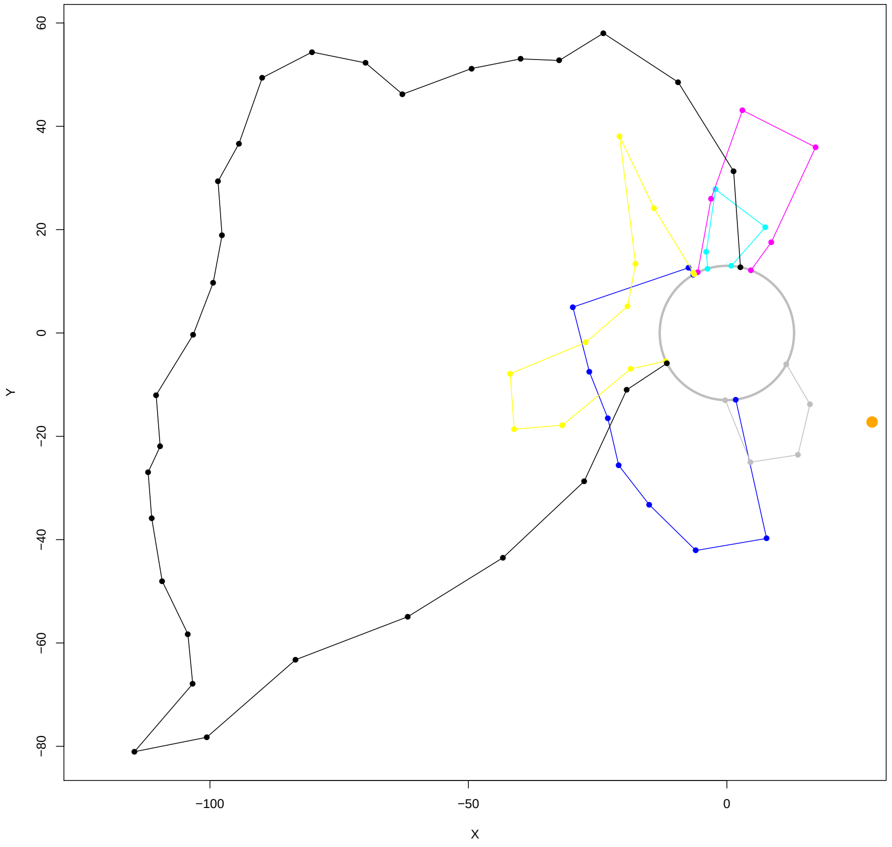

Fig SI-V-19 : Tracks of bee JP-5

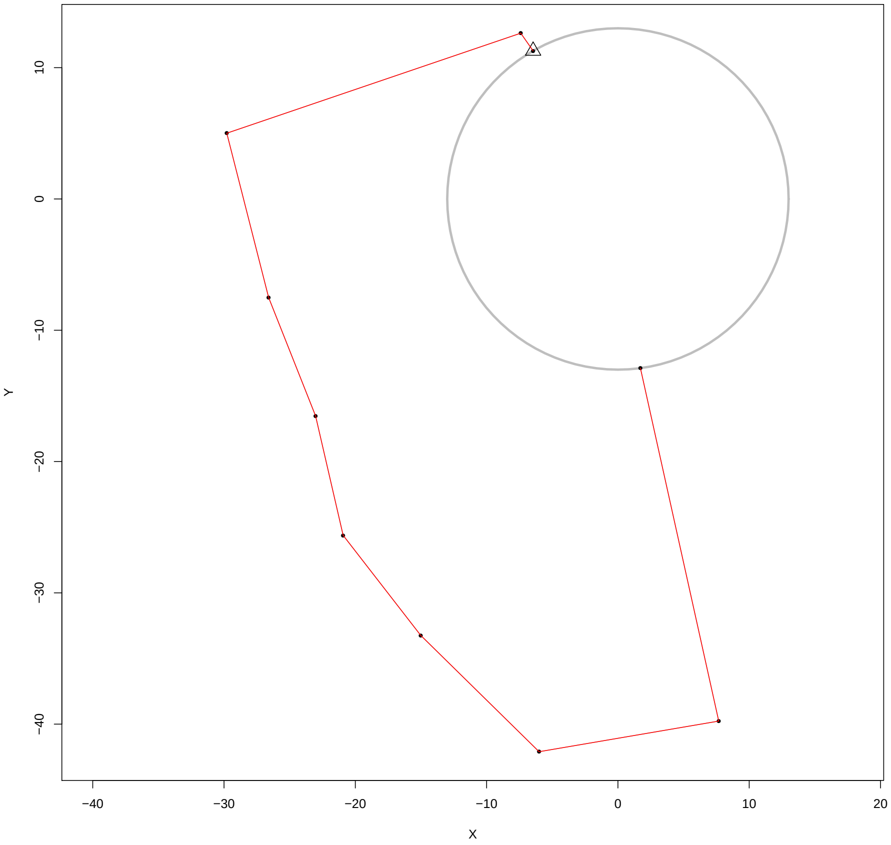

Fig SI-V-19.1 : Loop 132 — loop 1 of bee JP-5

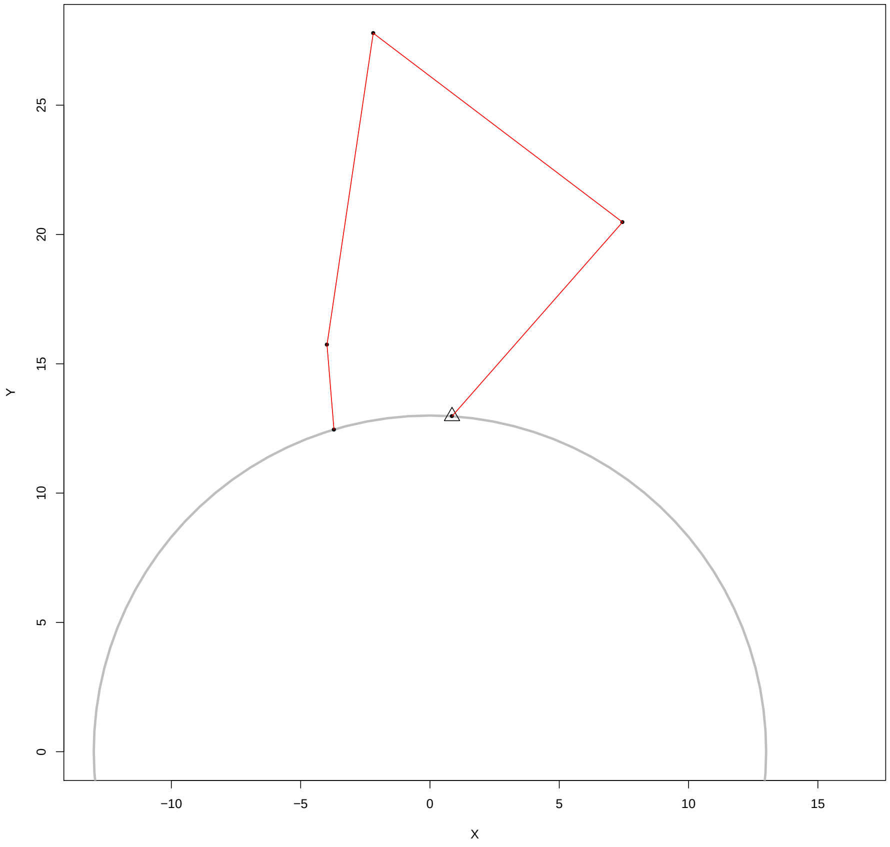

Fig SI-V-19.2 : Loop 133 — loop 2 of bee JP-5

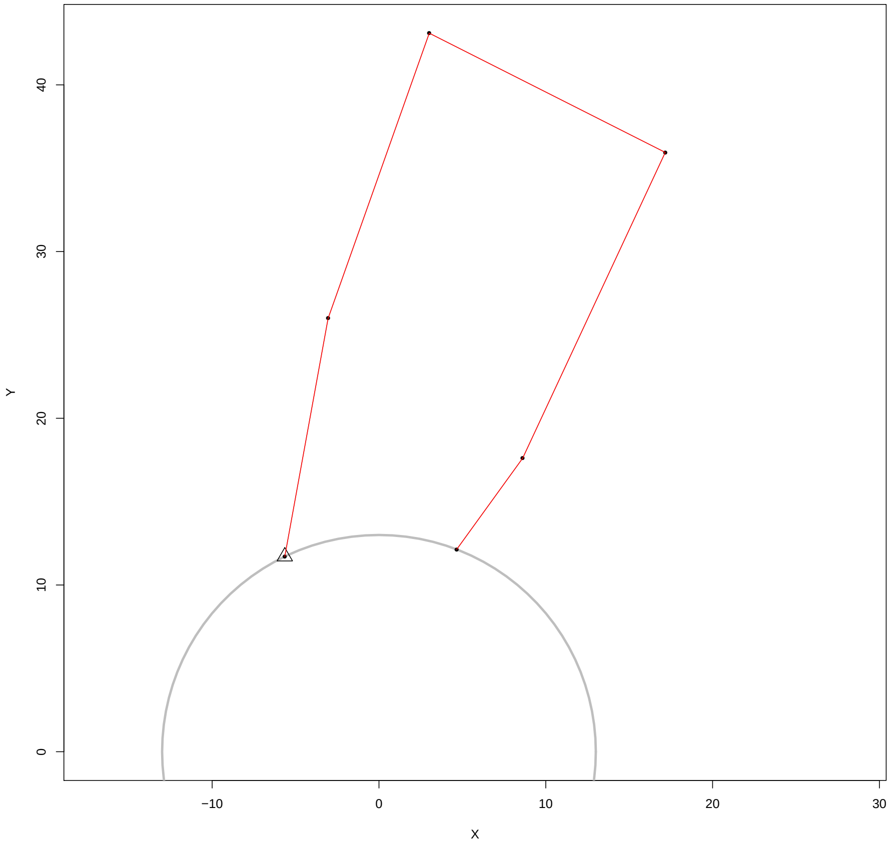

Fig SI-V-19.3 : Loop 134 — loop 3 of bee JP-5

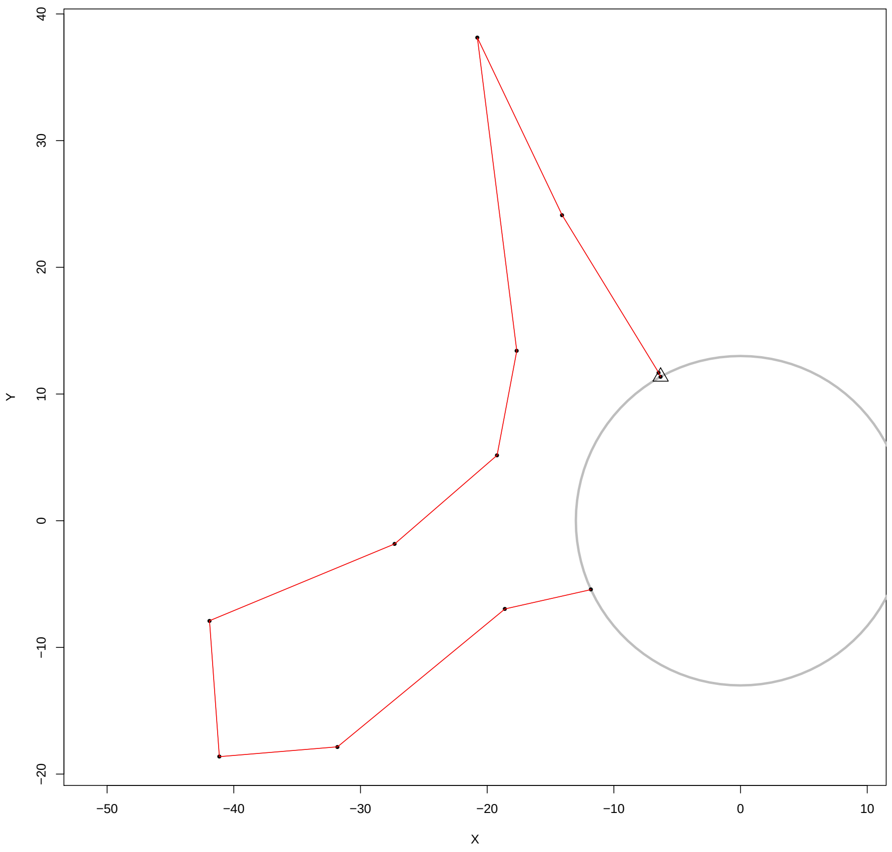

Fig SI-V-19.4 : Loop 135 — loop 4 of bee JP-5

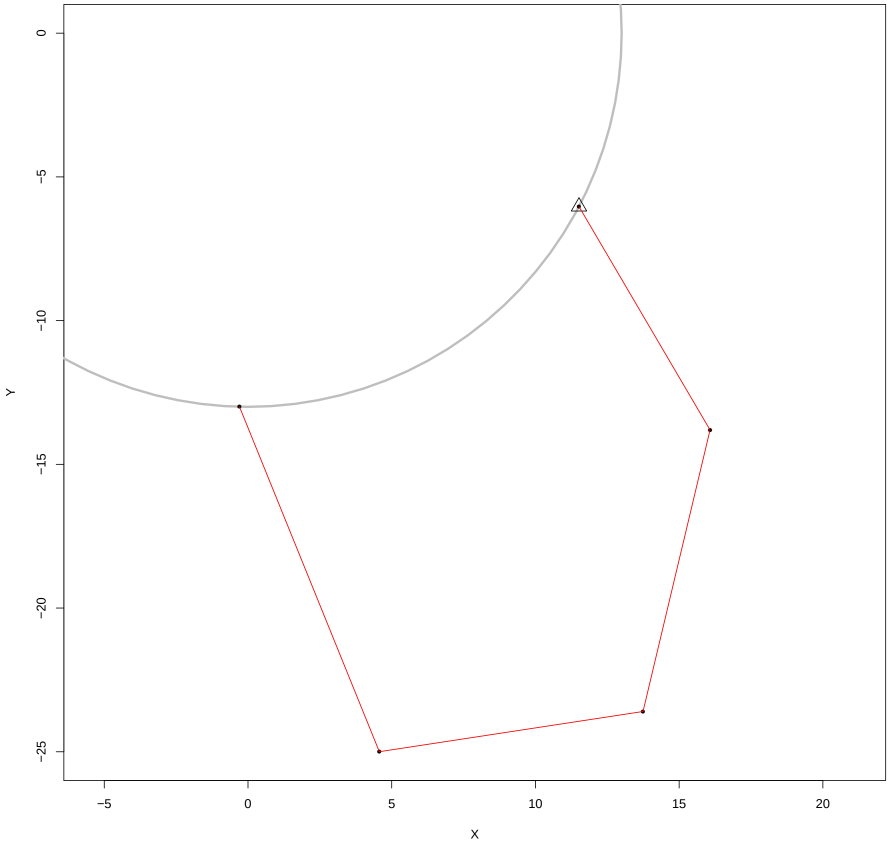

Fig SI-V-19.5 : Loop 136 — loop 5 of bee JP-5

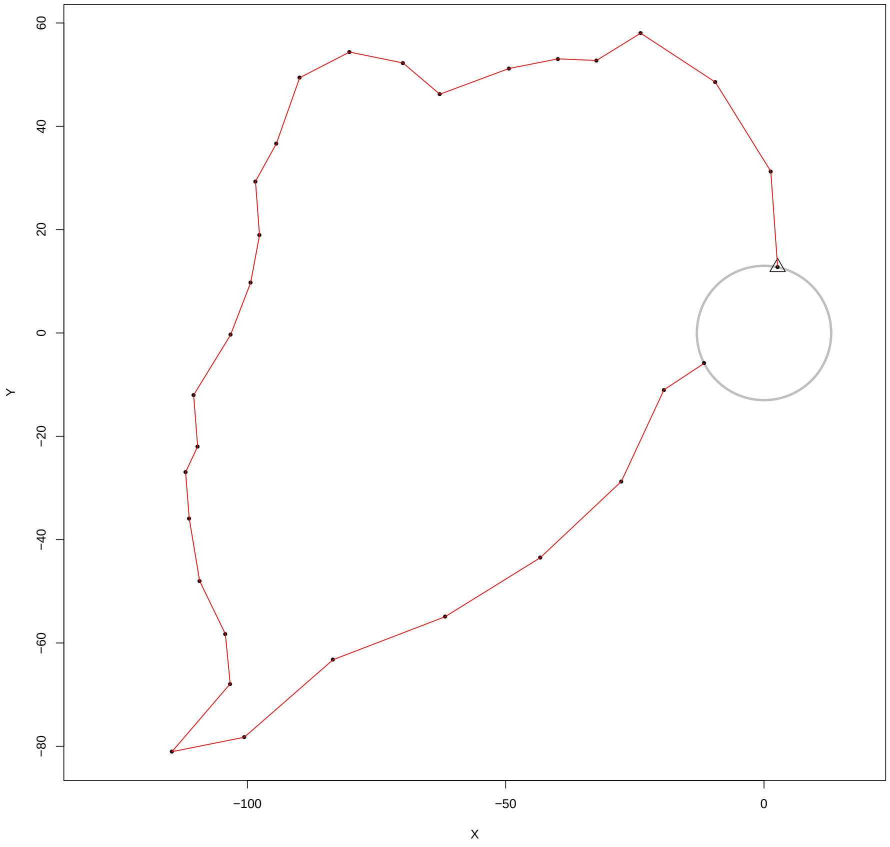

Fig SI-V-19.6 : Loop 137 — loop 6 of bee JP-5

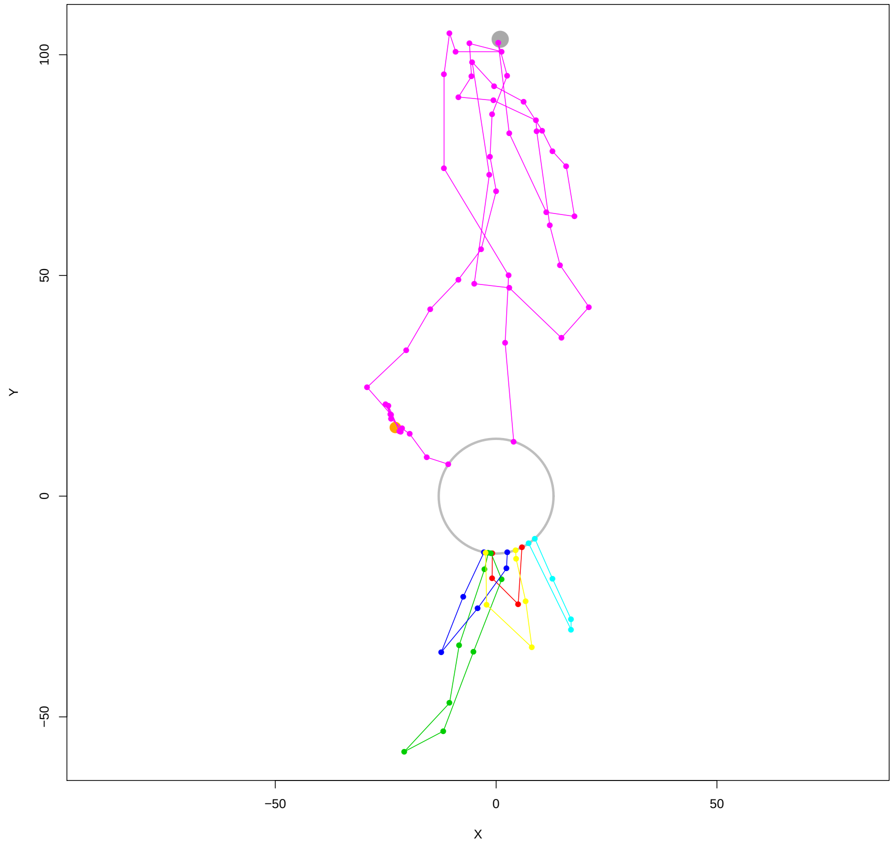

Fig SI-V-20 : Tracks of bee KH-1

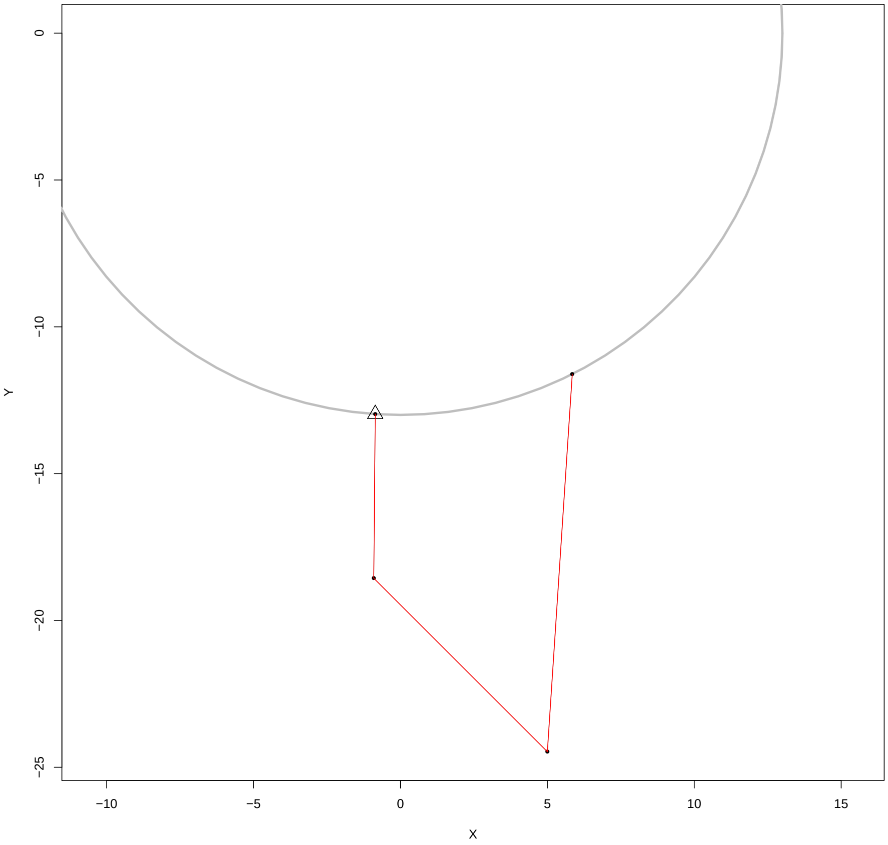

Fig SI-V-20.1 : Loop 138 — loop 1 of bee KH-1

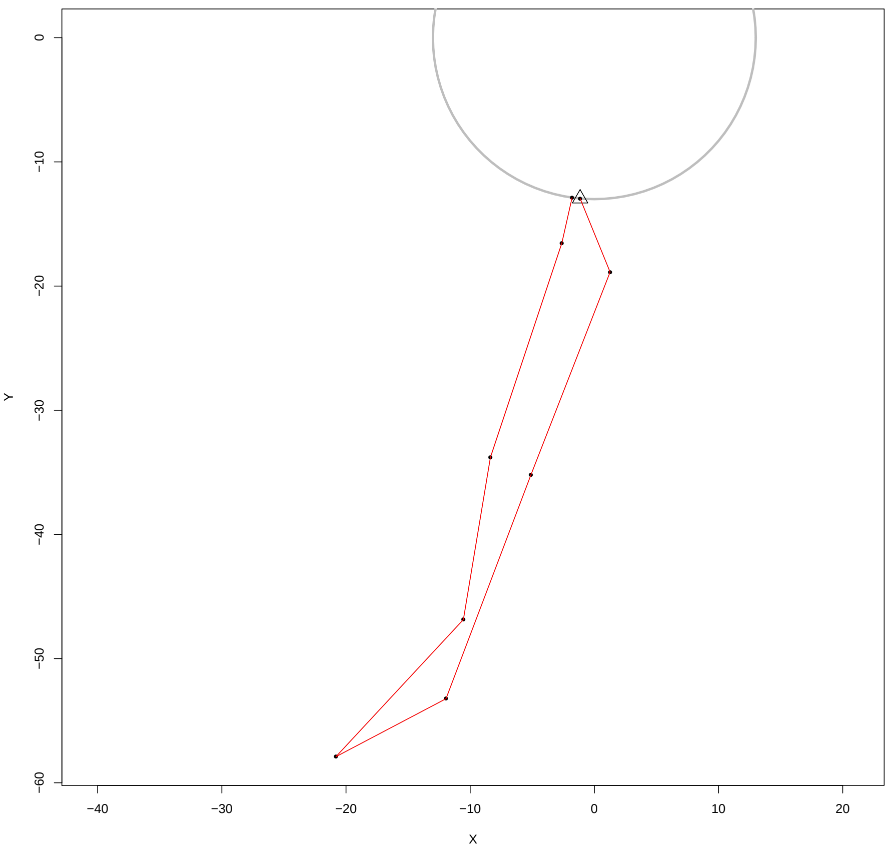

Fig SI-V-20.2 : Loop 139 — loop 2 of bee KH-1

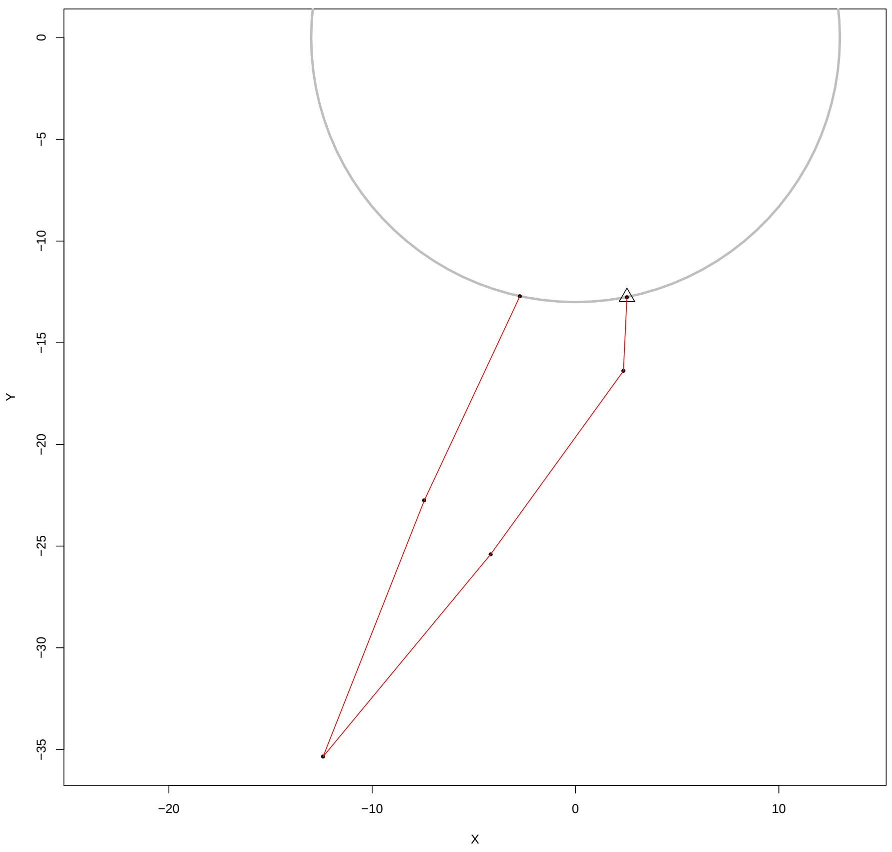

Fig SI-V-20.3 : Loop 140 — loop 3 of bee KH-1

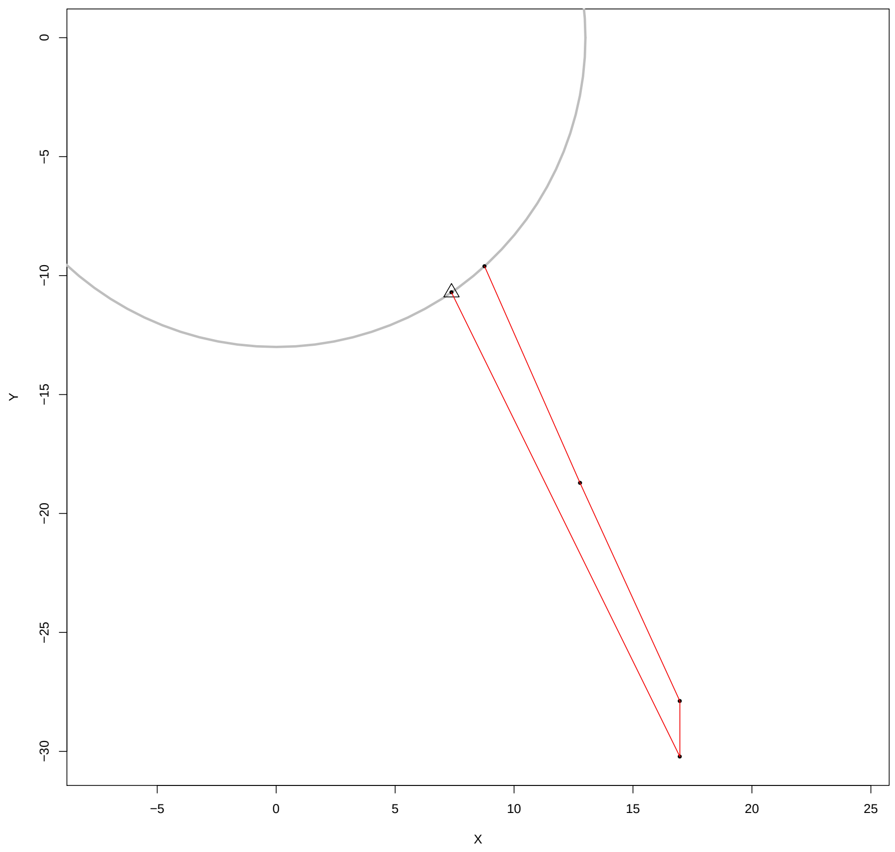

Fig SI-V-20.4 : Loop 141 — loop 4 of bee KH-1

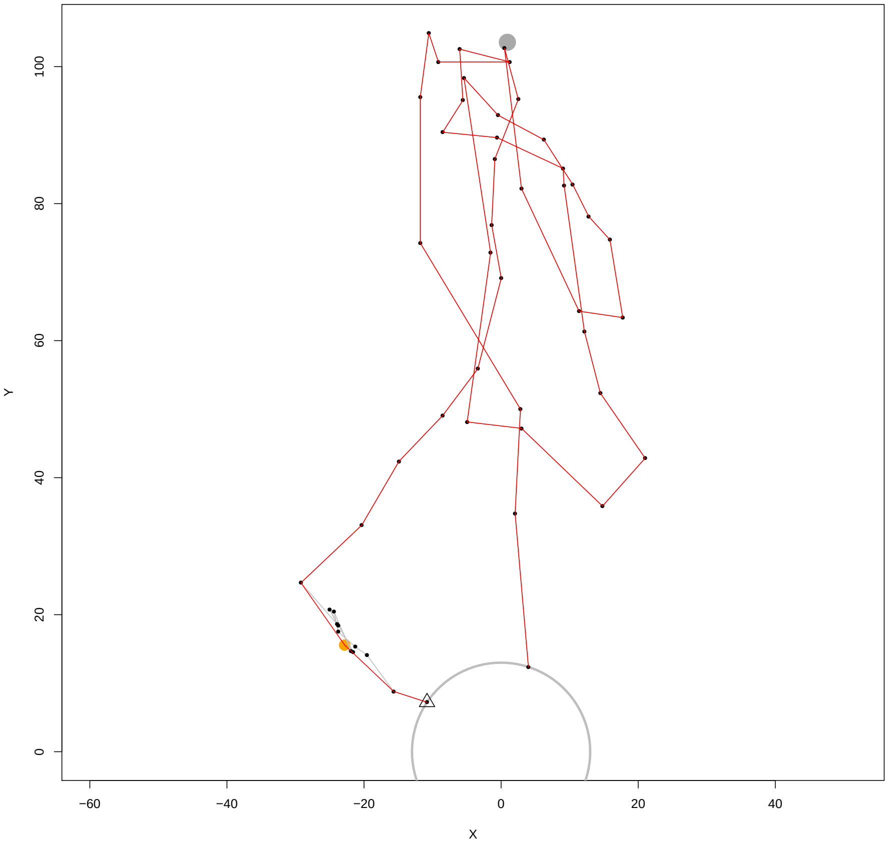

Fig SI-V-20.5 : Loop 142 — loop 5 of bee KH-1

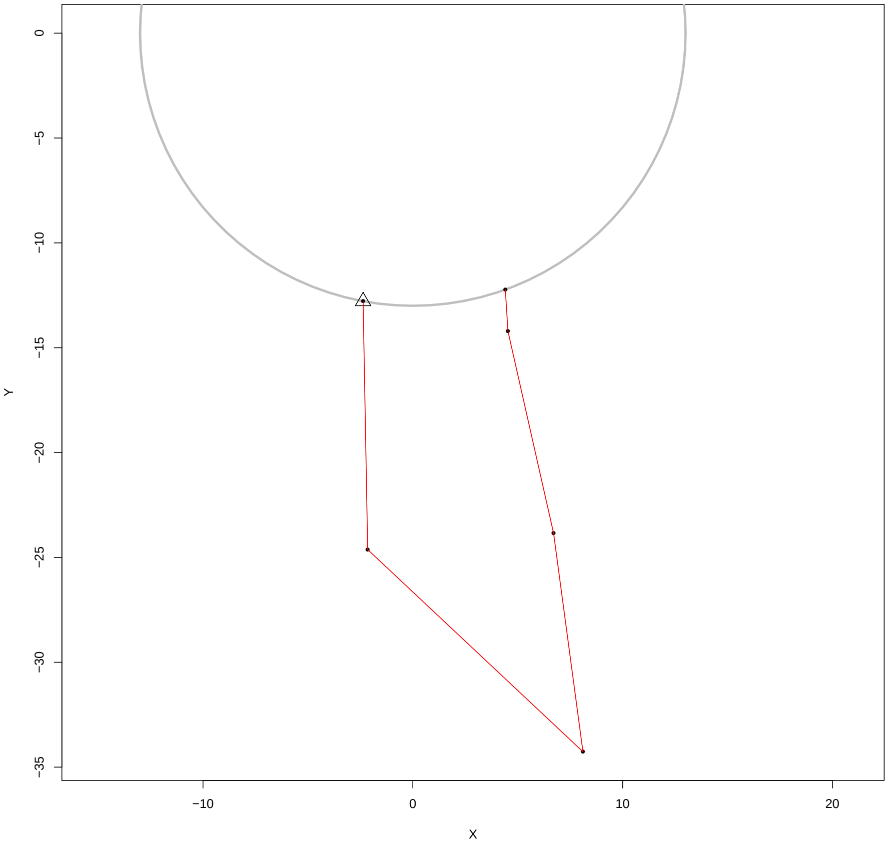

Fig SI-V-20.6 : Loop 143 — loop 6 of bee KH-1

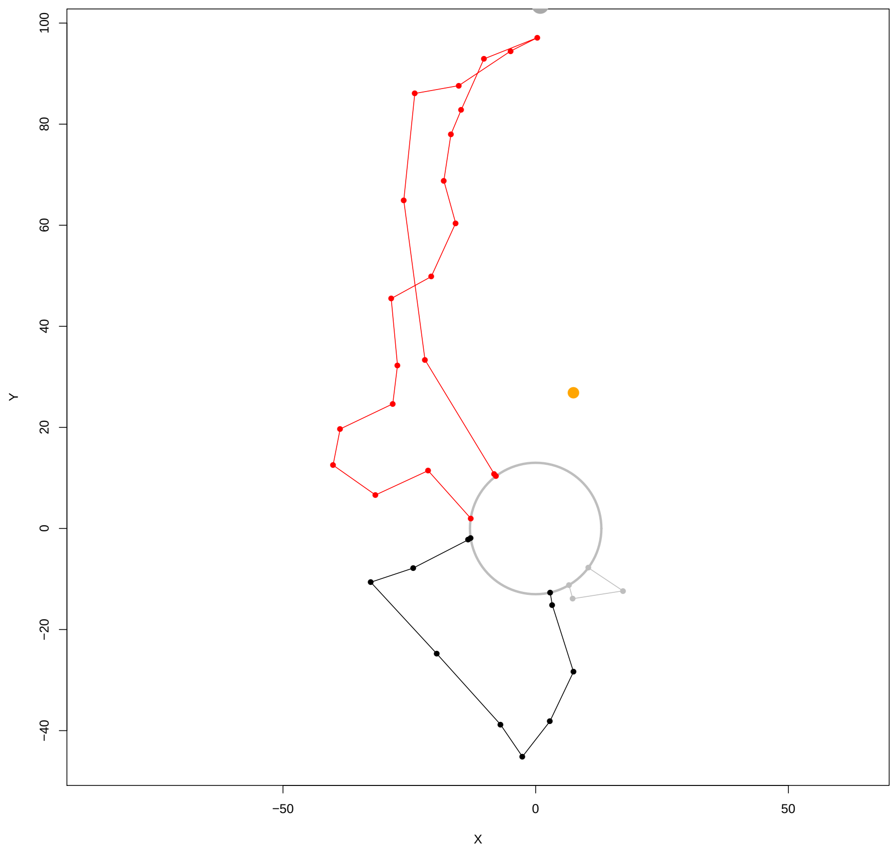

Fig SI-V-21 : Tracks of bee GF-1

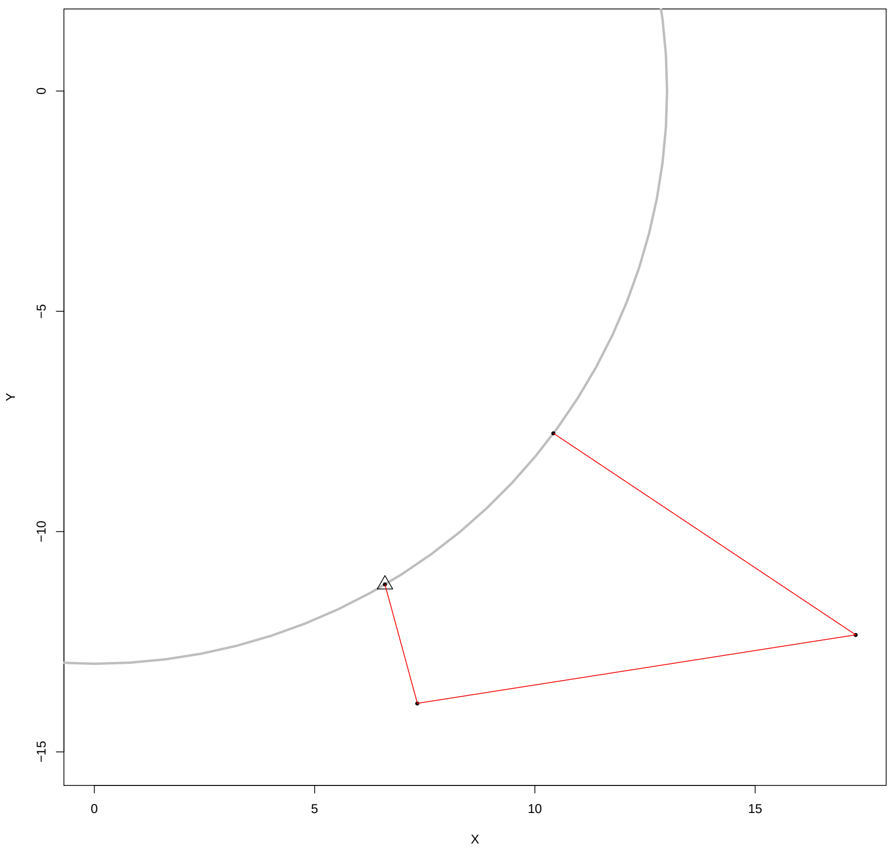

Fig SI-V-21.1 : Loop 144 — loop 1 of bee GF-1

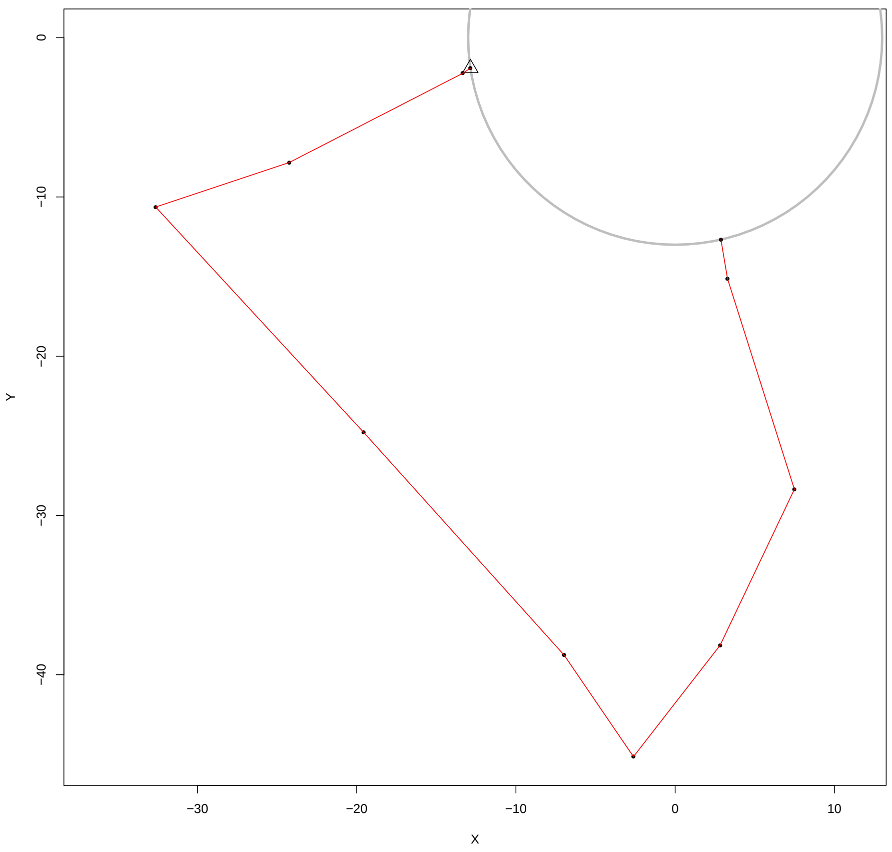

Fig SI-V-21.2 : Loop 145 — loop 2 of bee GF-1

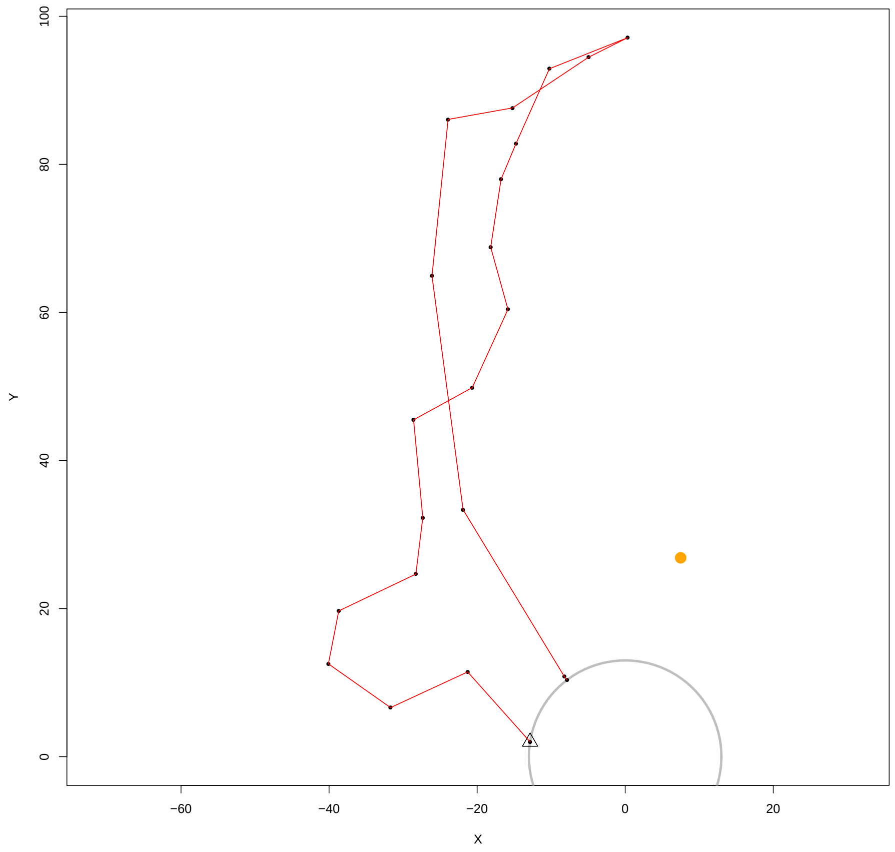

Fig SI-V-21.3 : Loop 146 — loop 3 of bee GF-1

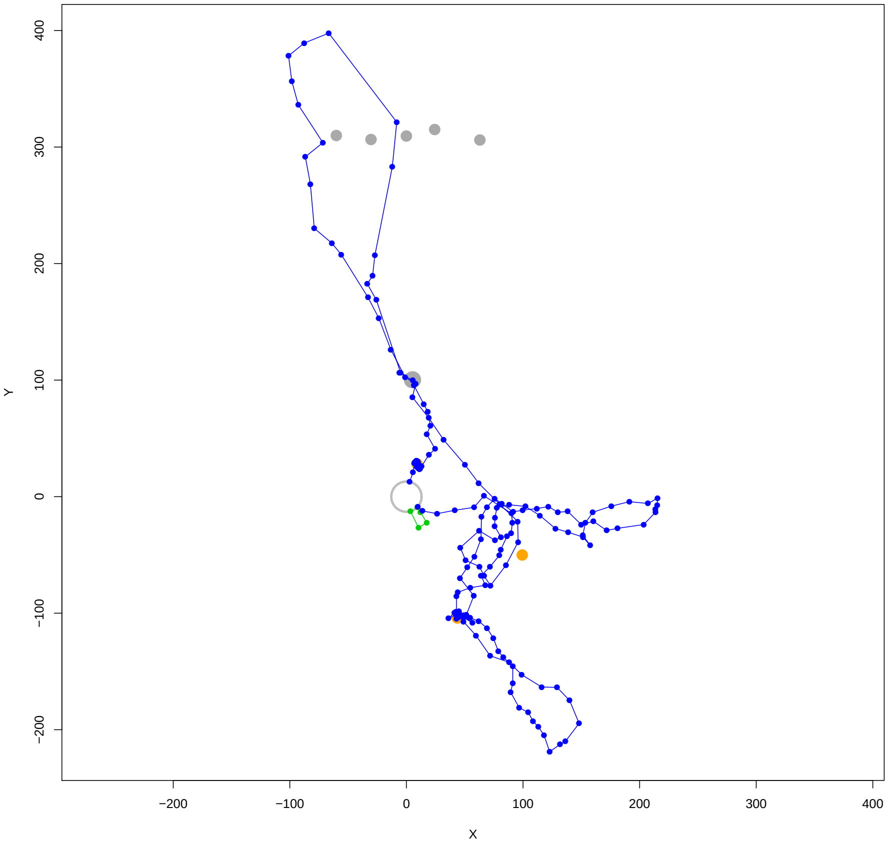

Fig SI-V-22 : Tracks of bee GE-3

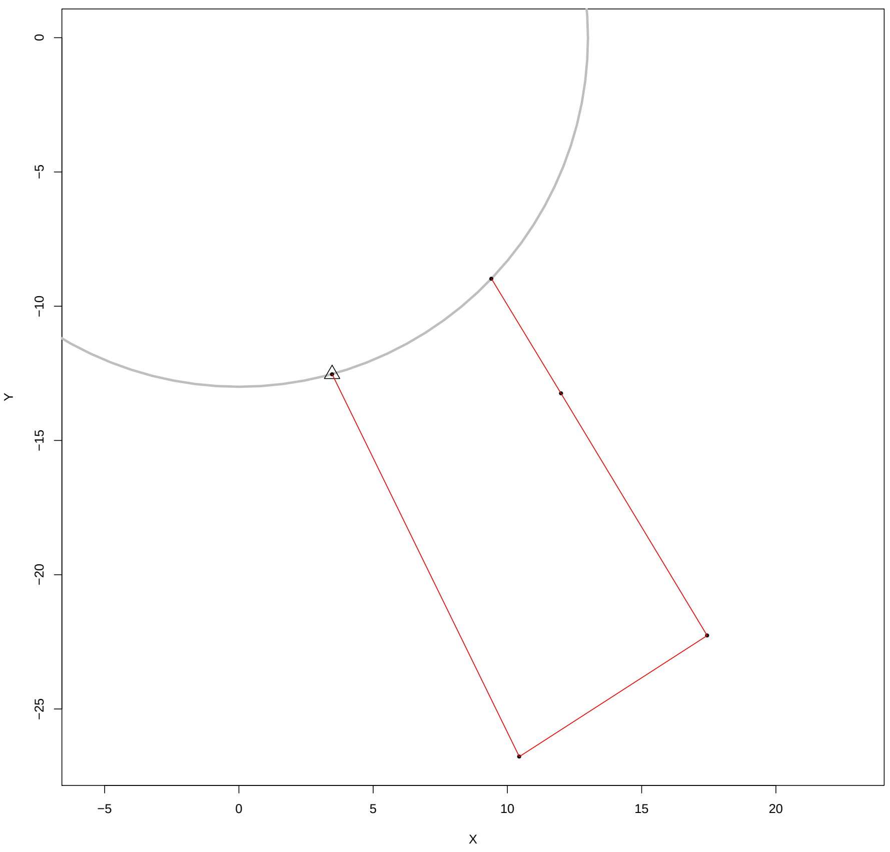

Fig SI-V-22.1 : Loop 147 — loop 1 of bee GE-3

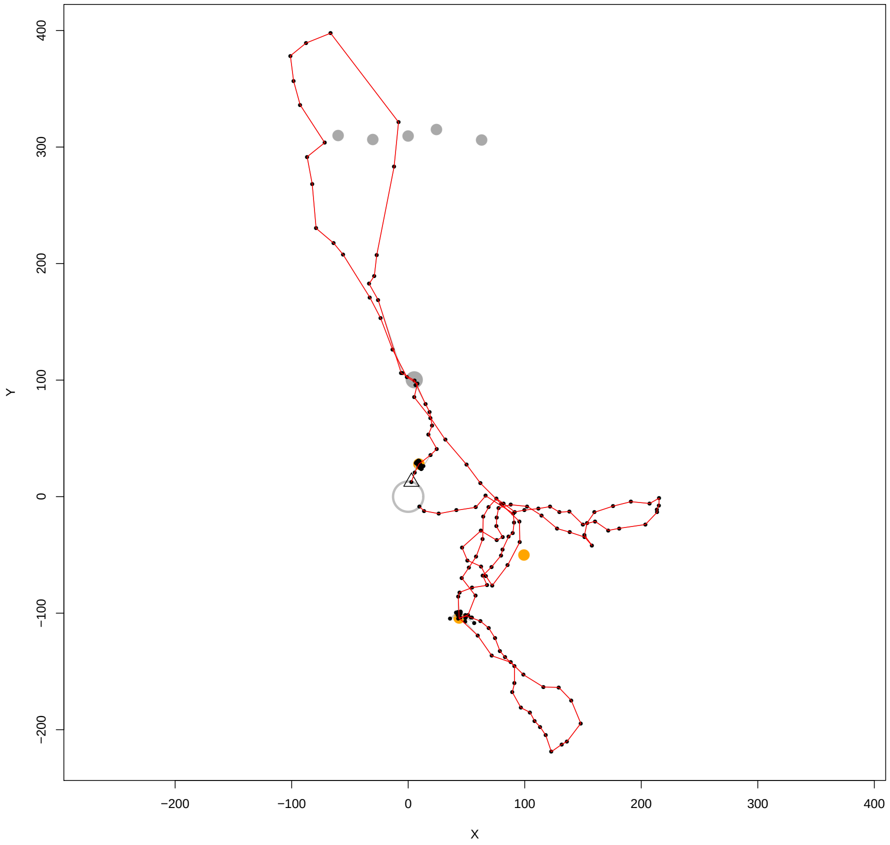

Fig SI-V-22.2 : Loop 148 — loop 2 of bee GE-3

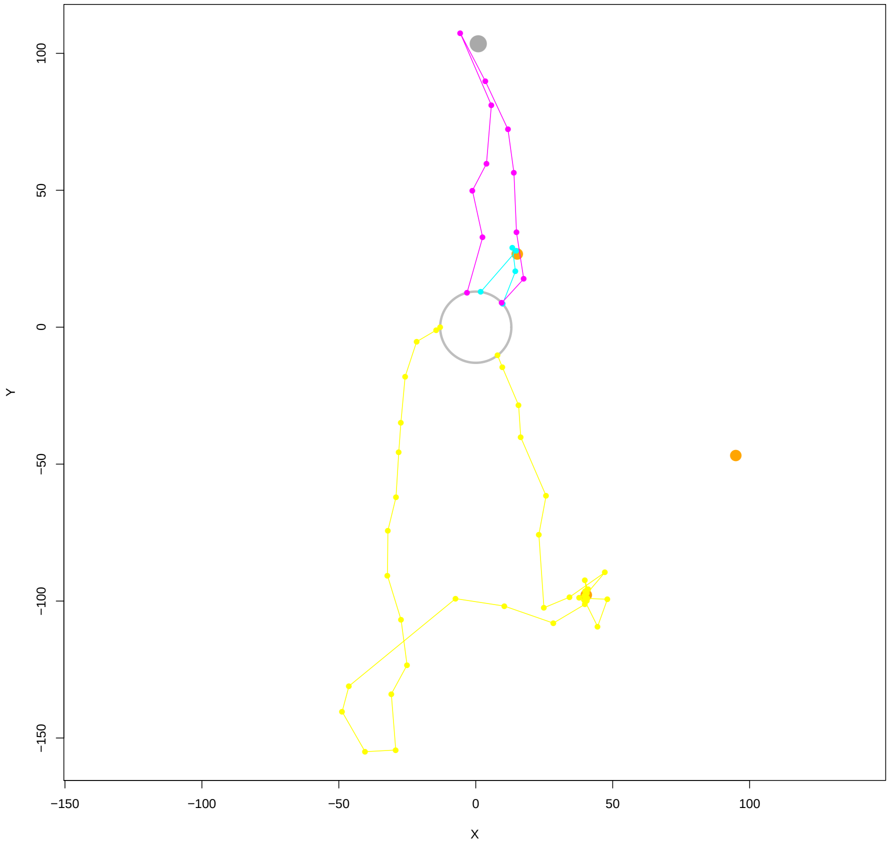

Fig SI-V-23 : Tracks of bee GS-2

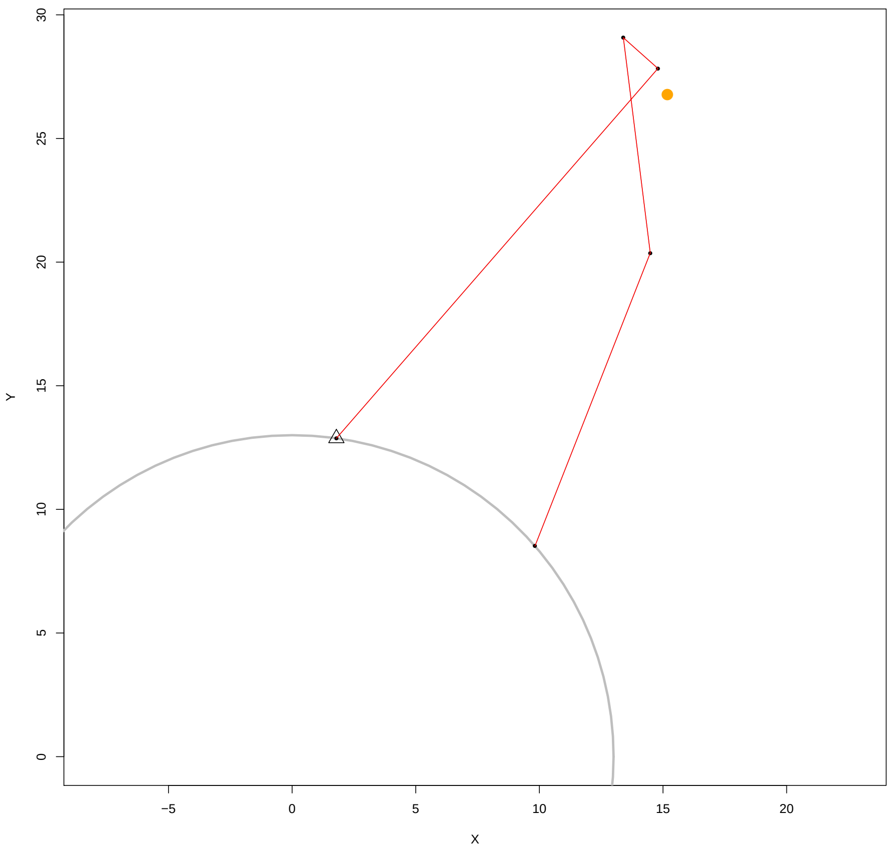

Fig SI-V-23.1 : Loop 149 — loop 1 of bee GS-2

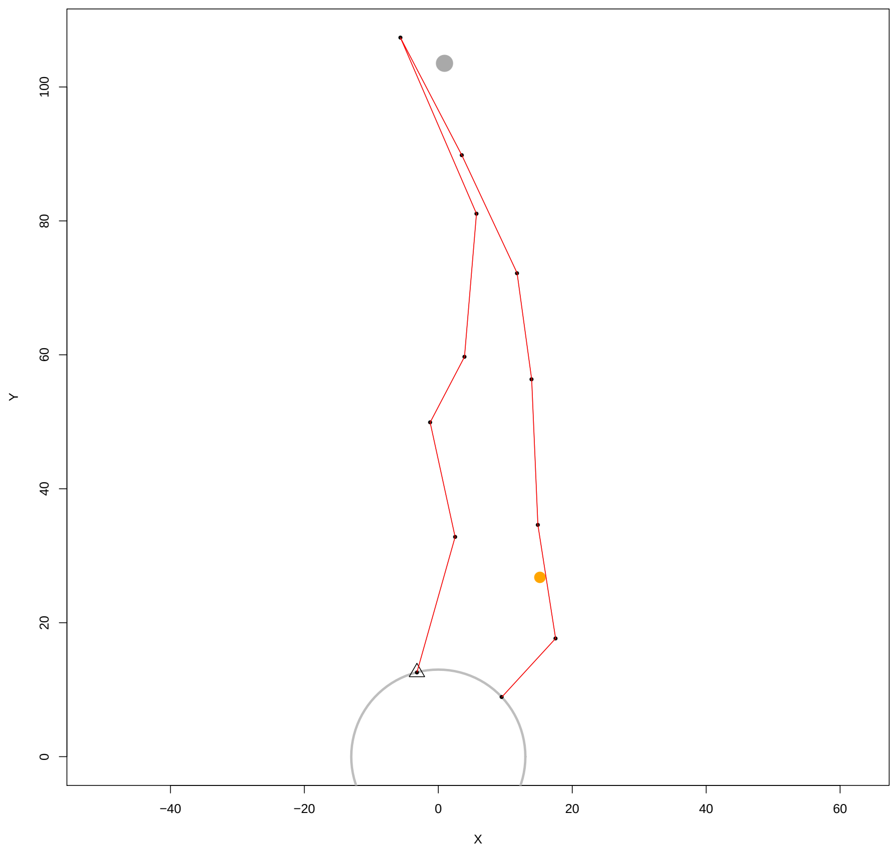

Fig SI-V-23.2 : Loop 150 — loop 2 of bee GS-2

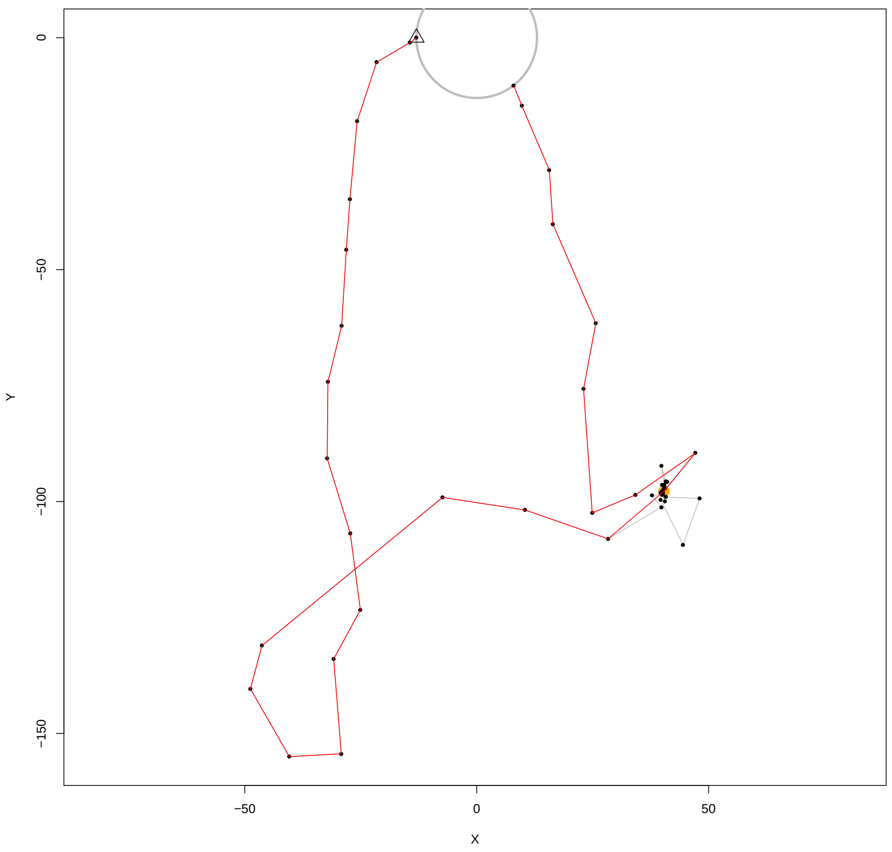

Fig SI-V-23.3 : Loop 151 — loop 3 of bee GS-2

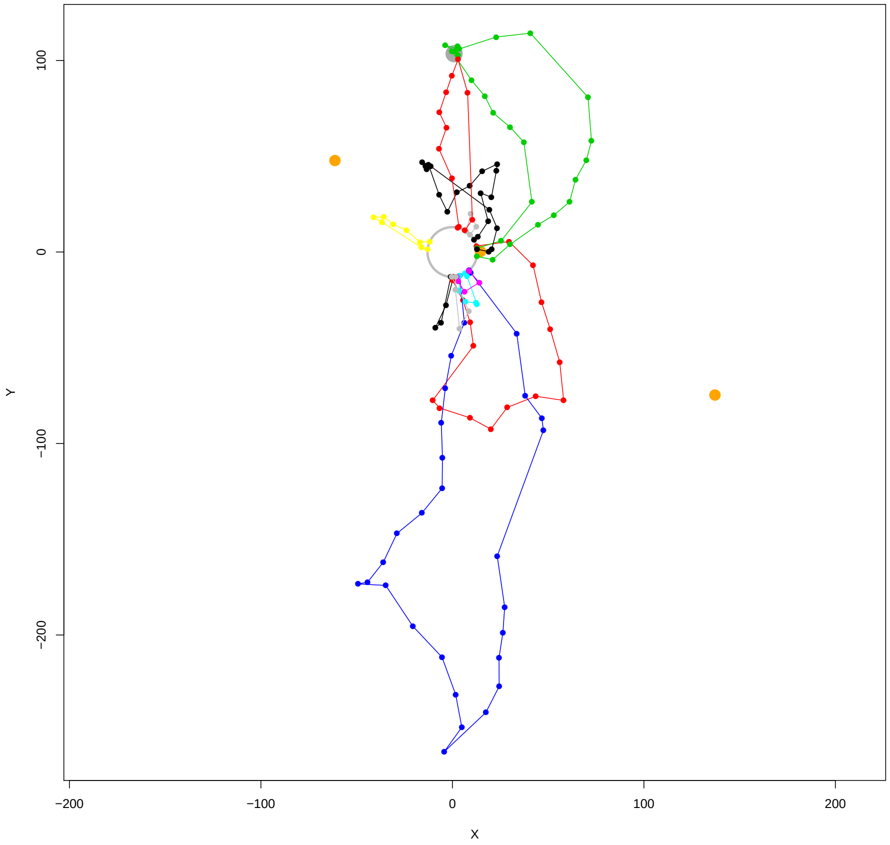

Fig SI-V-24 : Tracks of bee KG-1

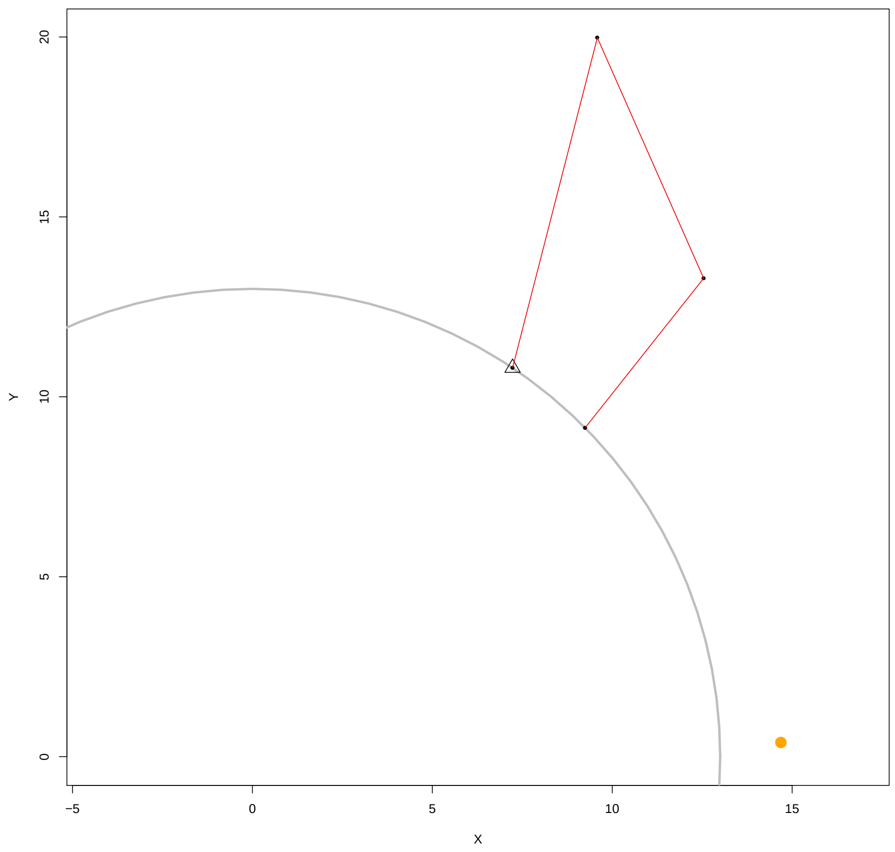

Fig SI-V-24.1 : Loop 152 — loop 1 of bee KG-1

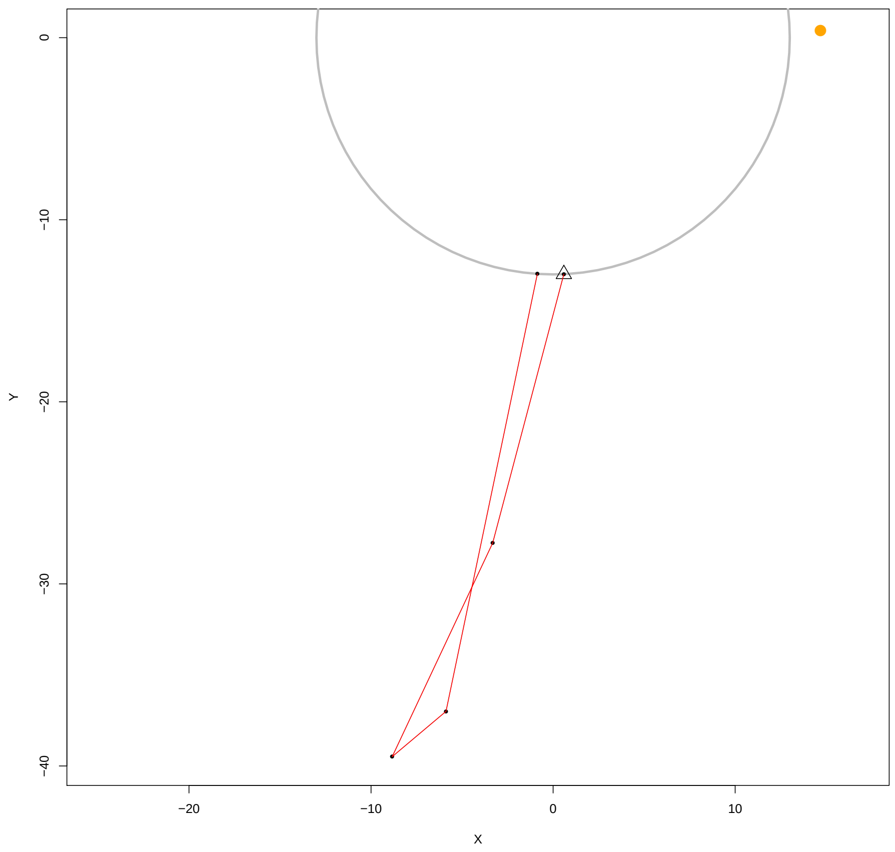

Fig SI-V-24.2 : Loop 153 — loop 2 of bee KG-1

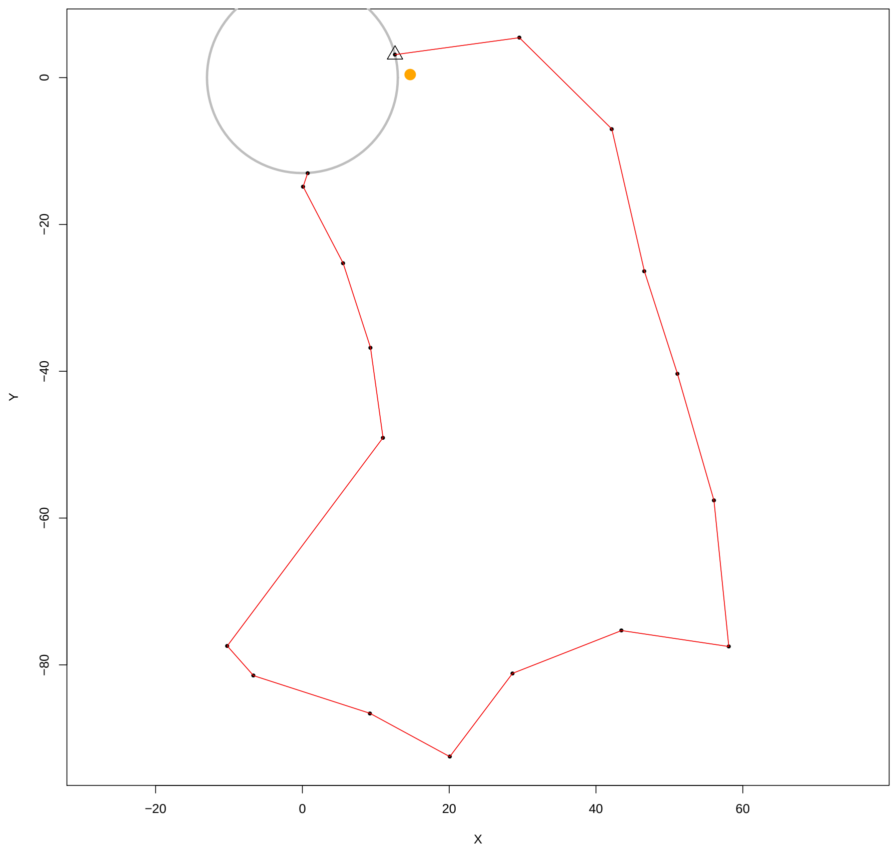

Fig SI-V-24.3 : Loop 154 — loop 3 of bee KG-1

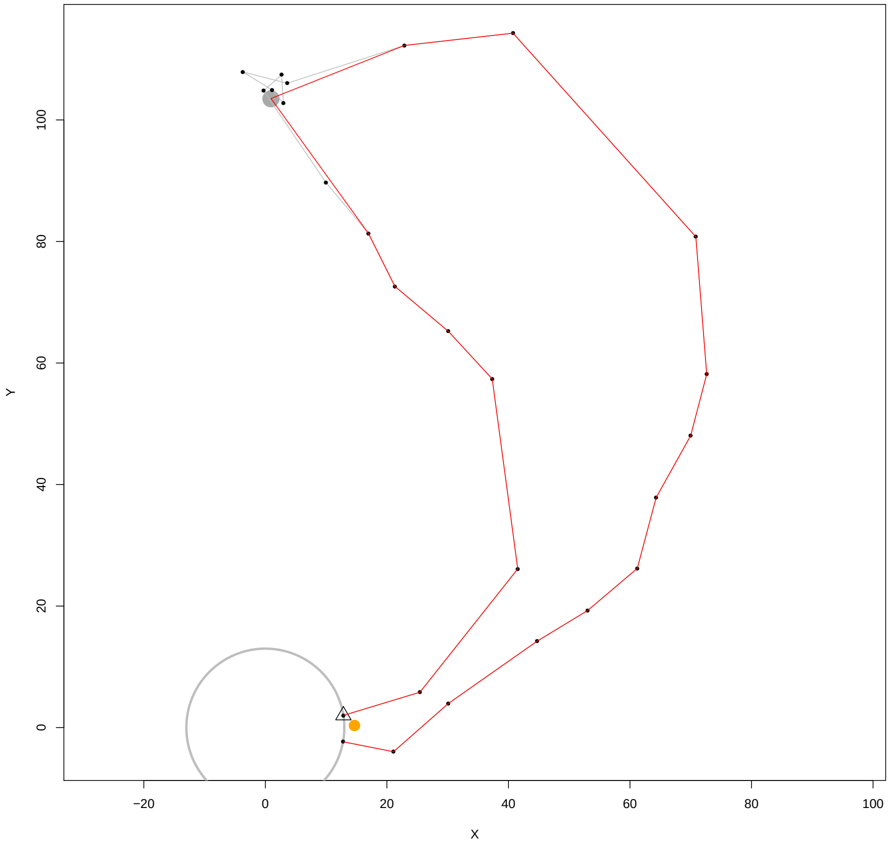

Fig SI-V-24.4 : Loop 155 — loop 4 of bee KG-1

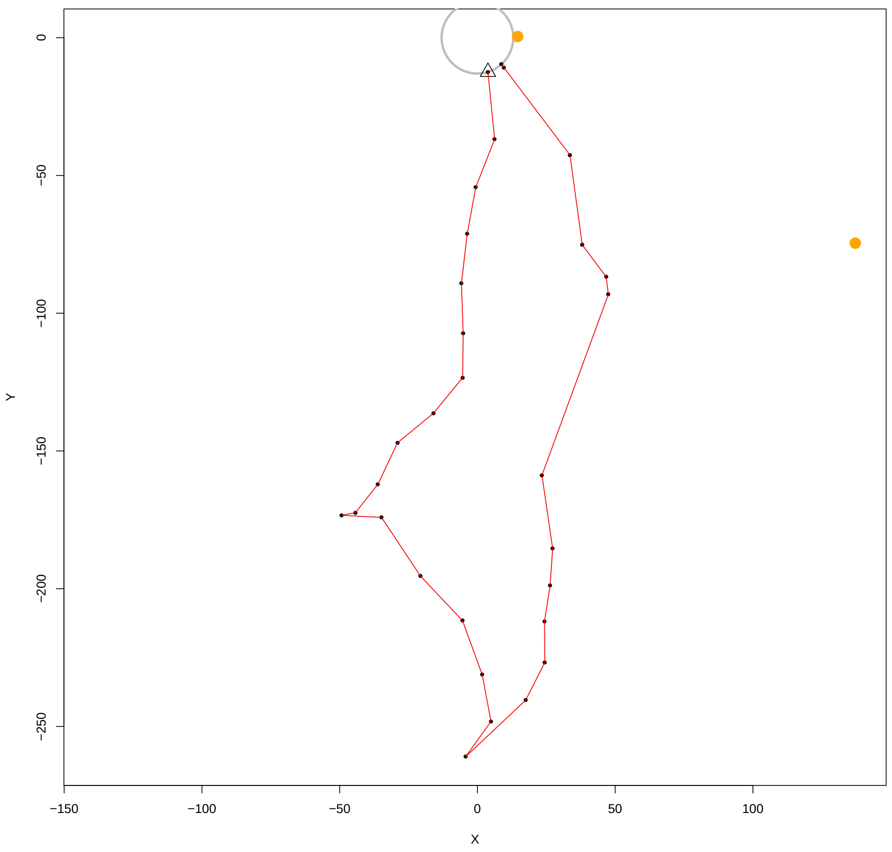

Fig SI-V-24.5 : Loop 156 — loop 5 of bee KG-1

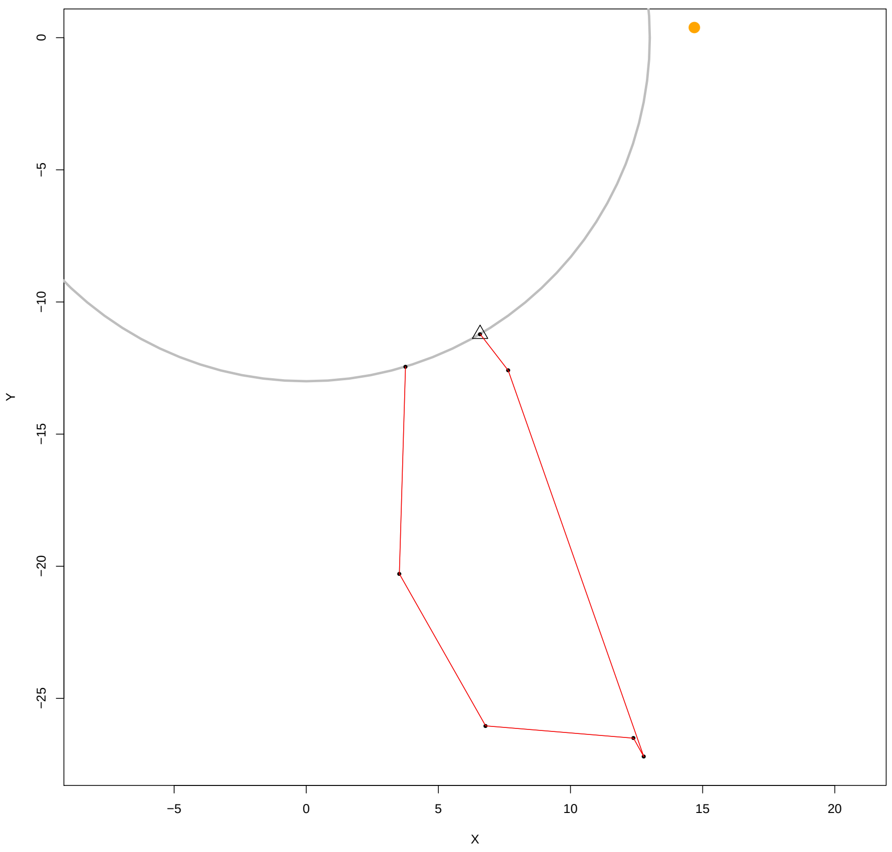

Fig SI-V-24.6 : Loop 157 — loop 6 of bee KG-1

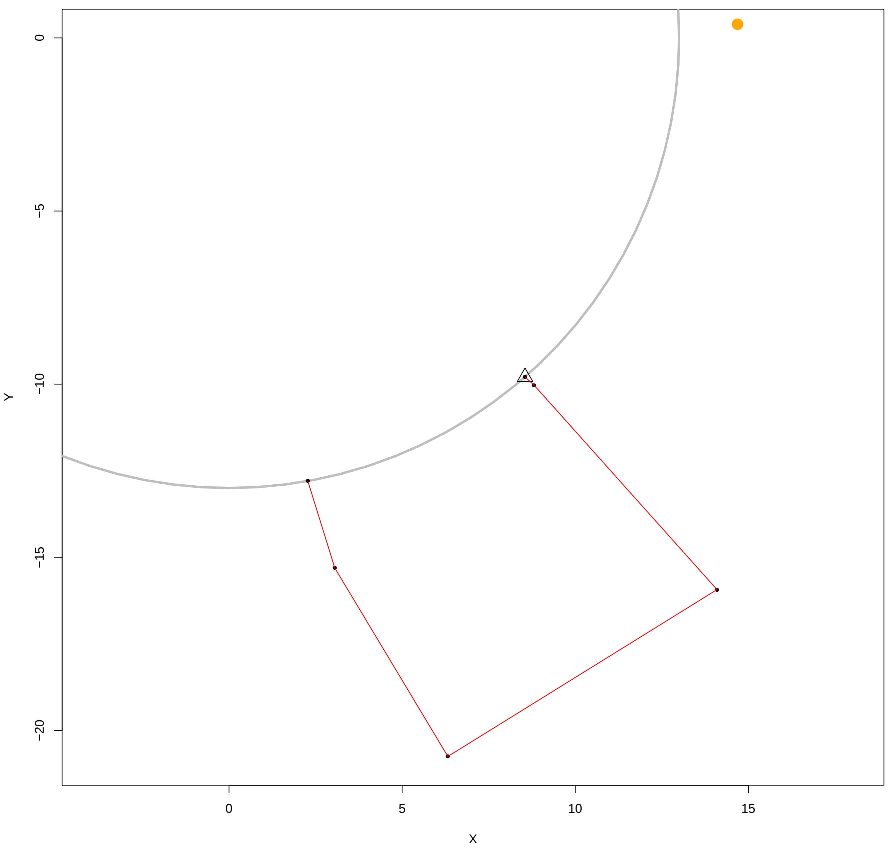

Fig SI-V-24.7 : Loop 158 — loop 7 of bee KG-1

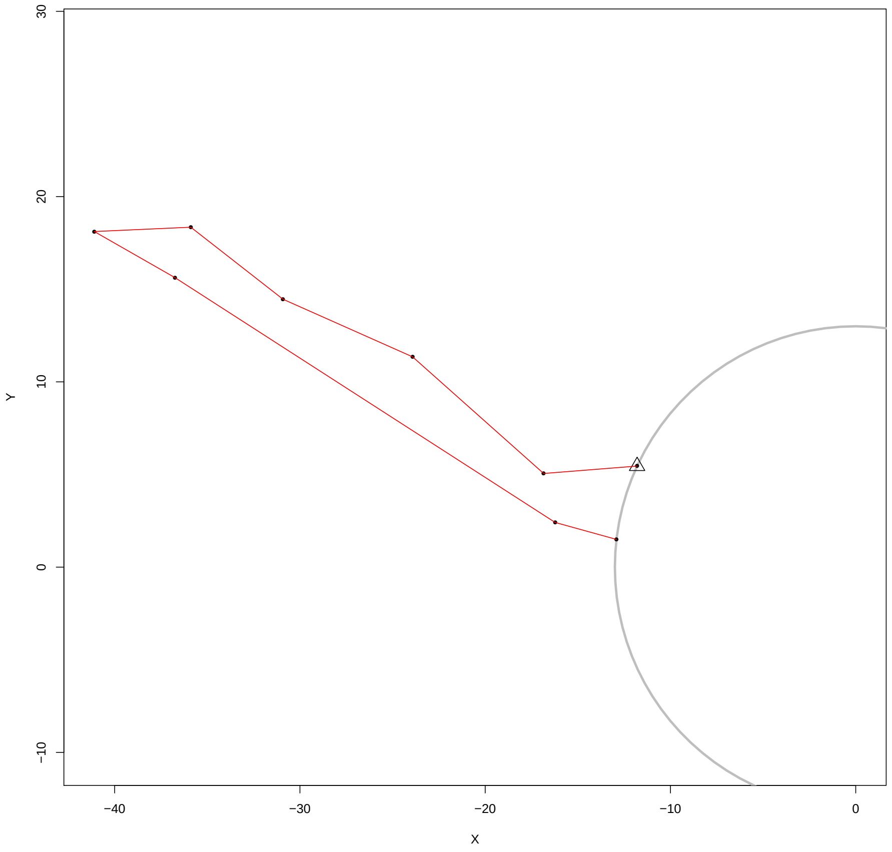

Fig SI-V-24.8 : Loop 159 — loop 8 of bee KG-1

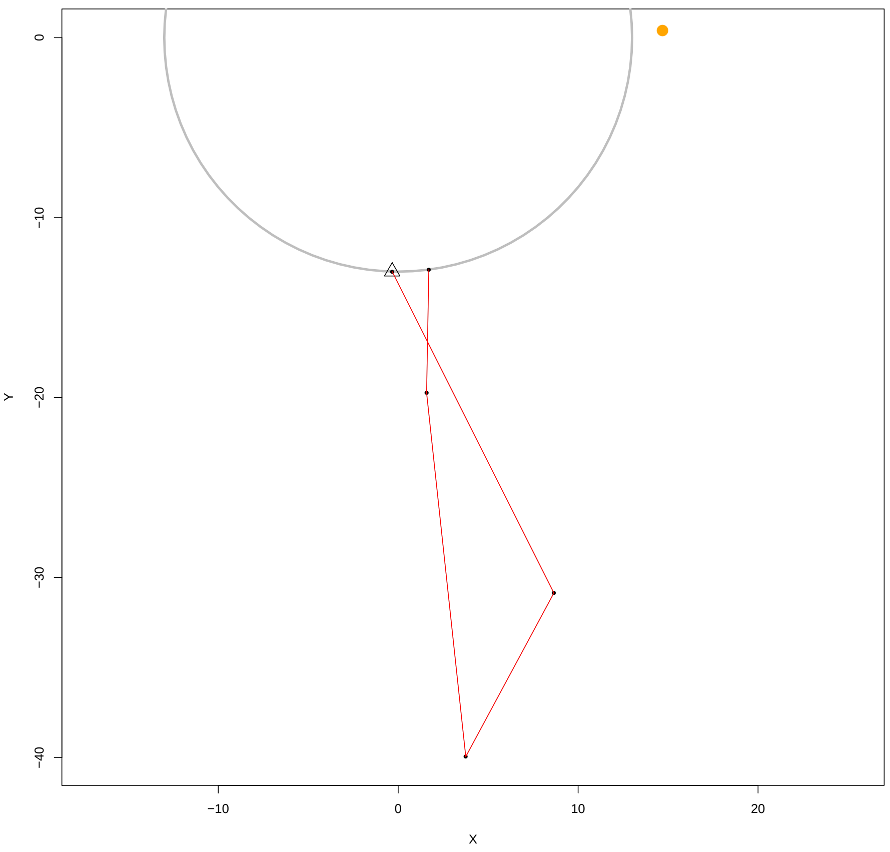

Fig SI-V-24.9 : Loop 160 — loop 9 of bee KG-1

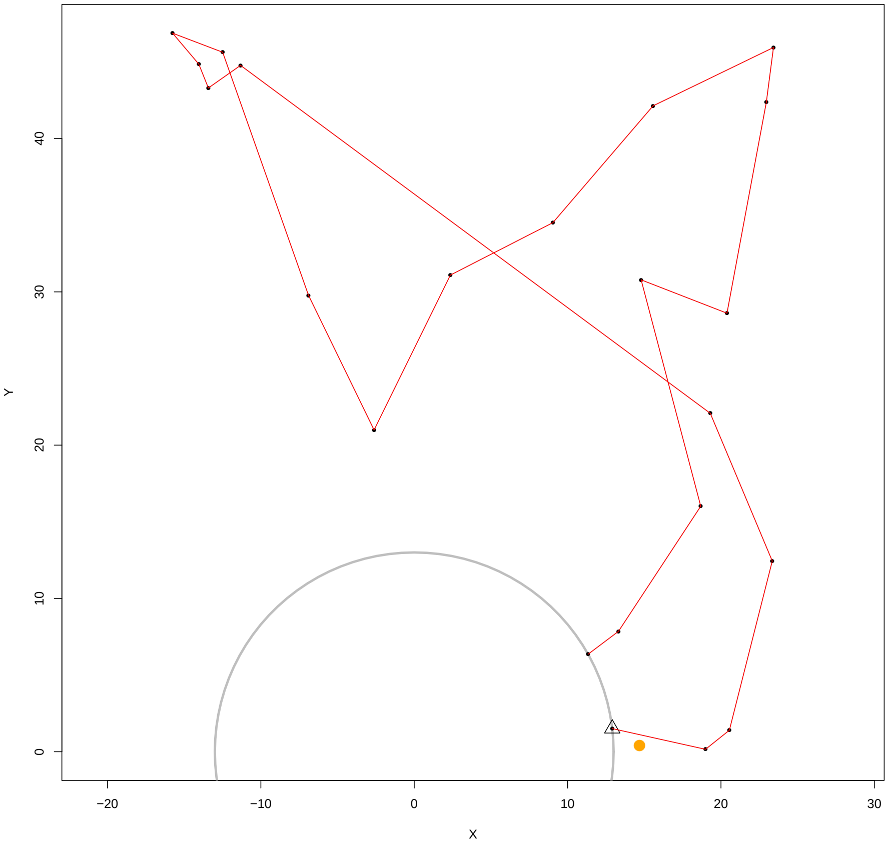

Fig SI-V-24.10 : Loop 161 — loop 10 of bee KG-1

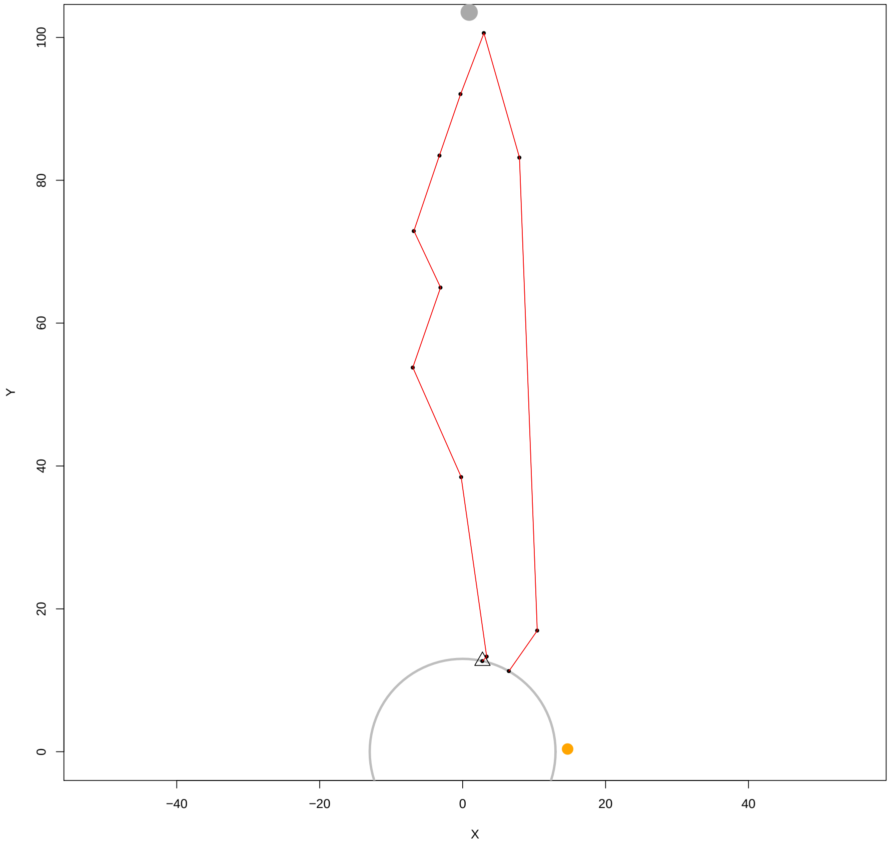

Fig SI-V-24.11 : Loop 162 — loop 11 of bee KG-1

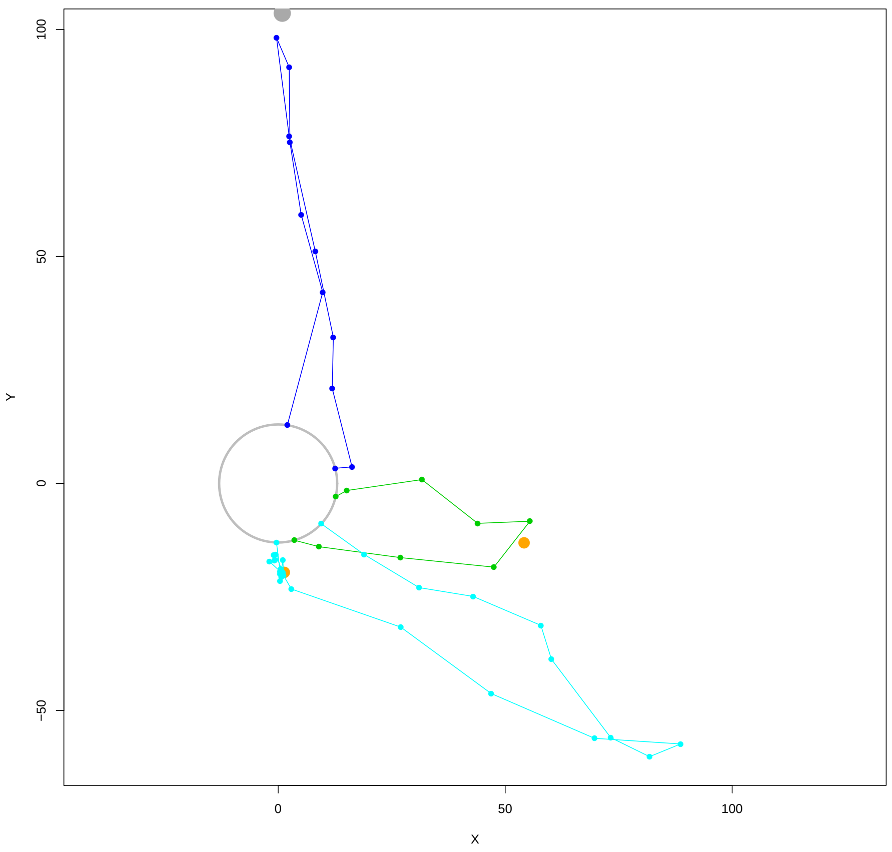

Fig SI-V-25 : Tracks of bee JN-4

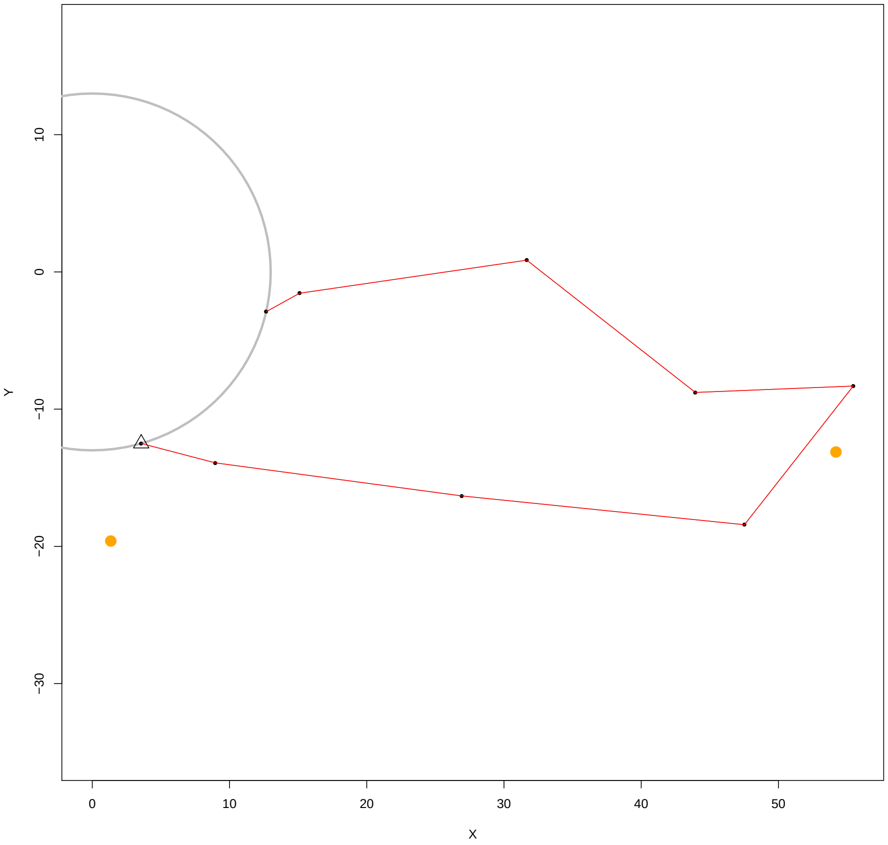

Fig SI-V-25.1 : Loop 163 — loop 1 of bee JN-4

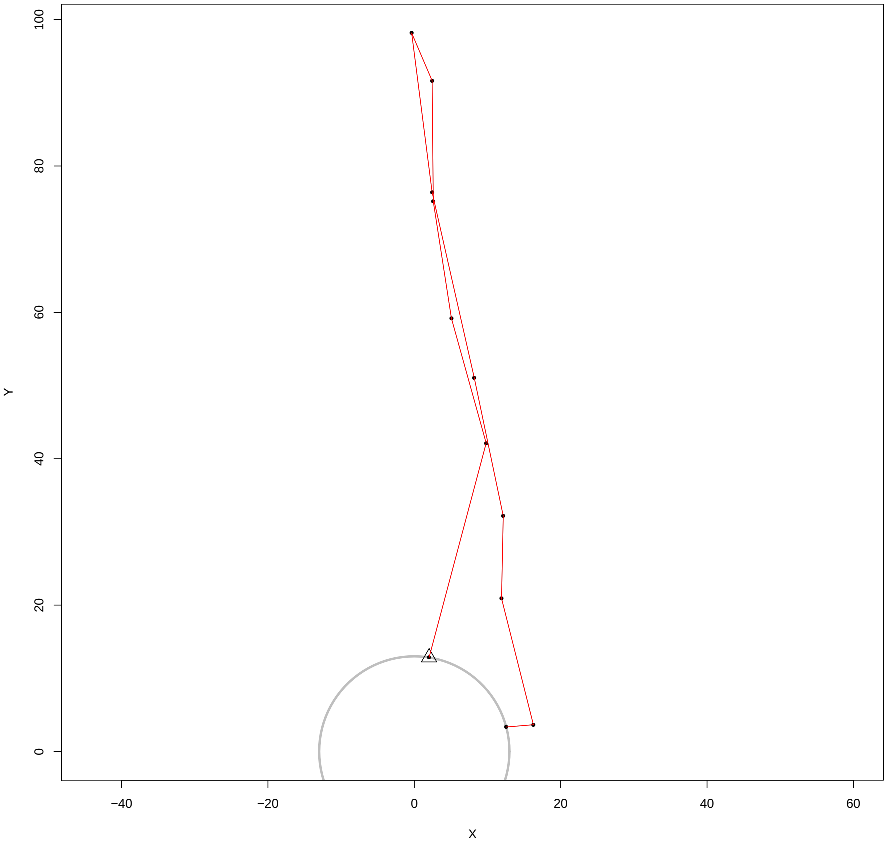

Fig SI-V-25.2 : Loop 164 — loop 2 of bee JN-4

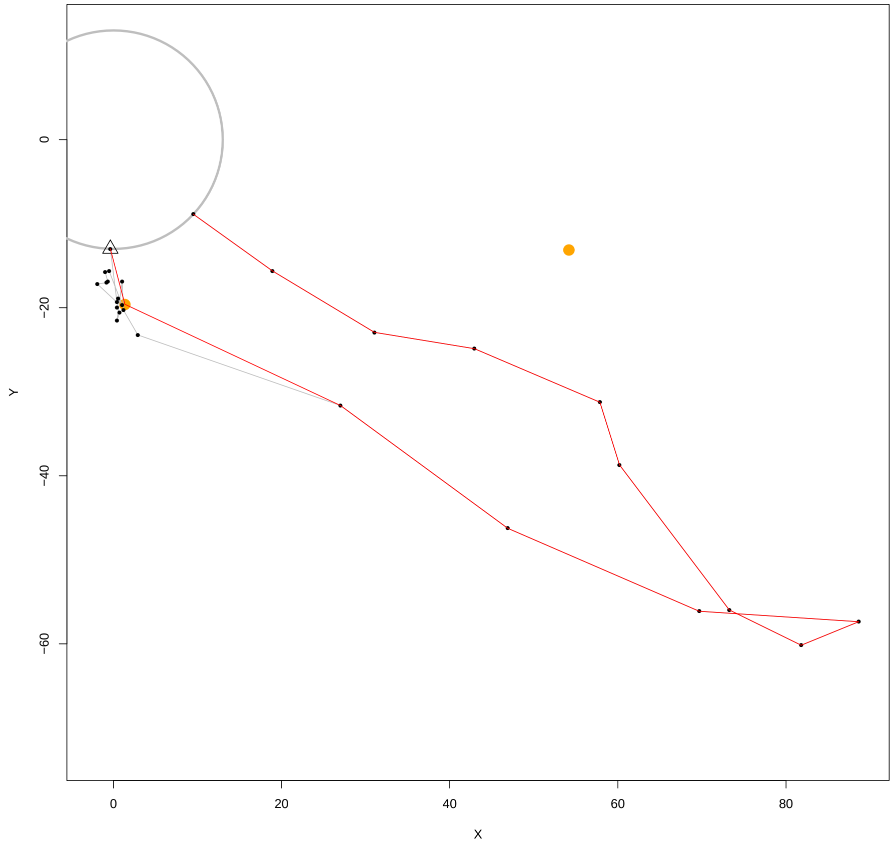

Fig SI-V-25.3 : Loop 165 — loop 3 of bee JN-4

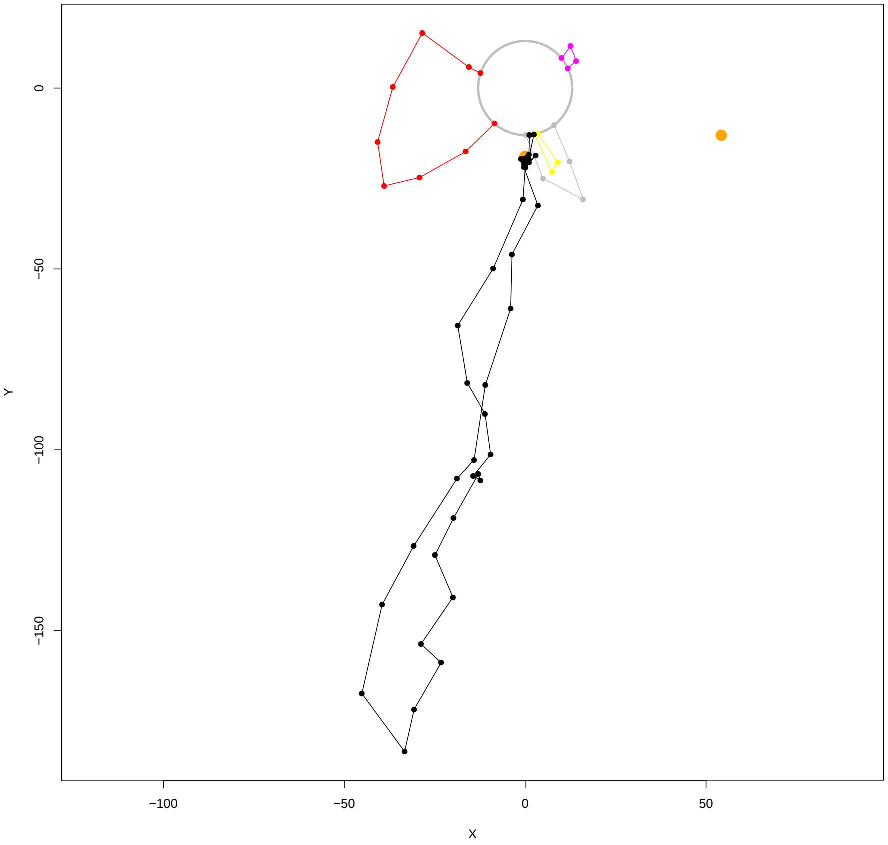

Fig SI-V-26 : Tracks of bee JU-1

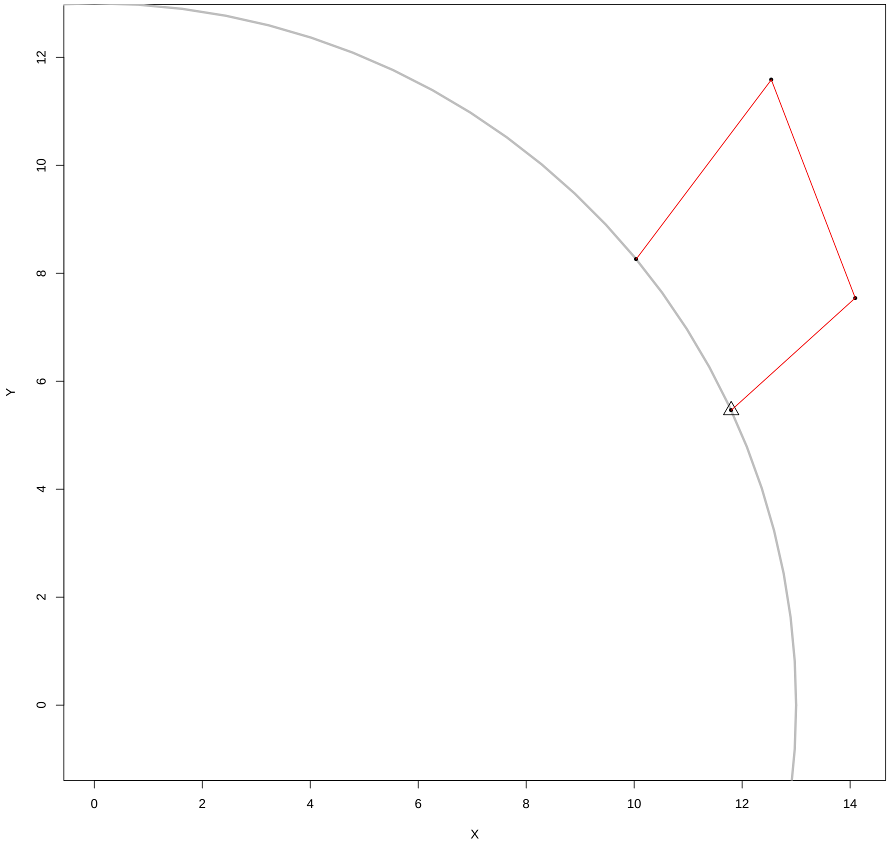

Fig SI-V-26.1 : Loop 166 — loop 1 of bee JU-1

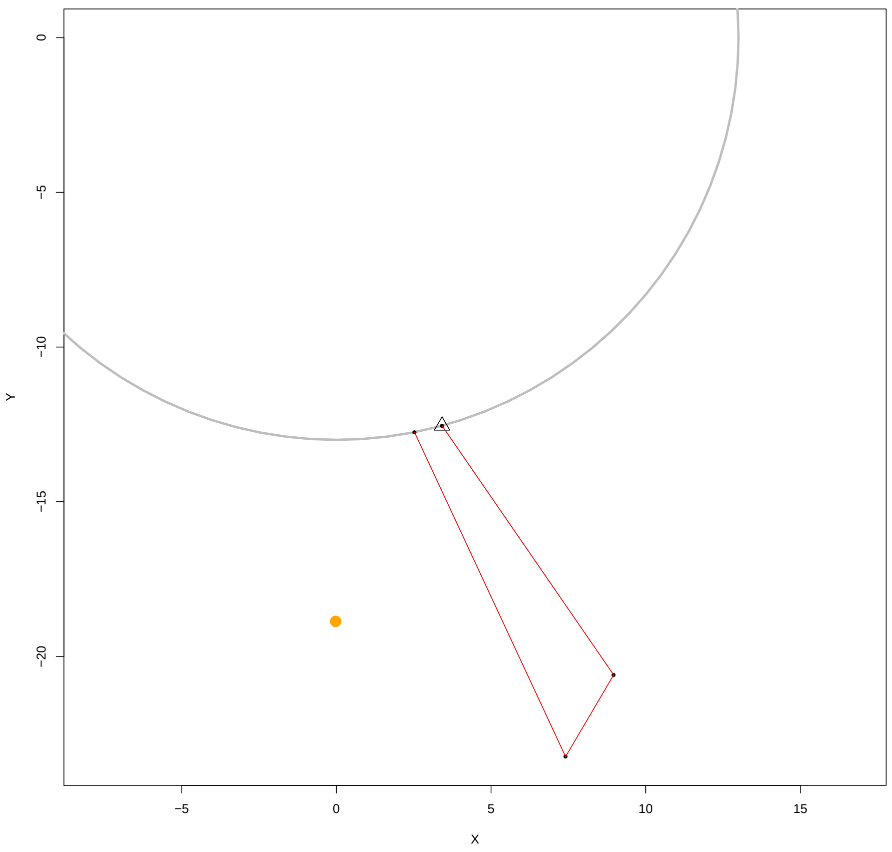

Fig SI-V-26.2 : Loop 167 — loop 2 of bee JU-1

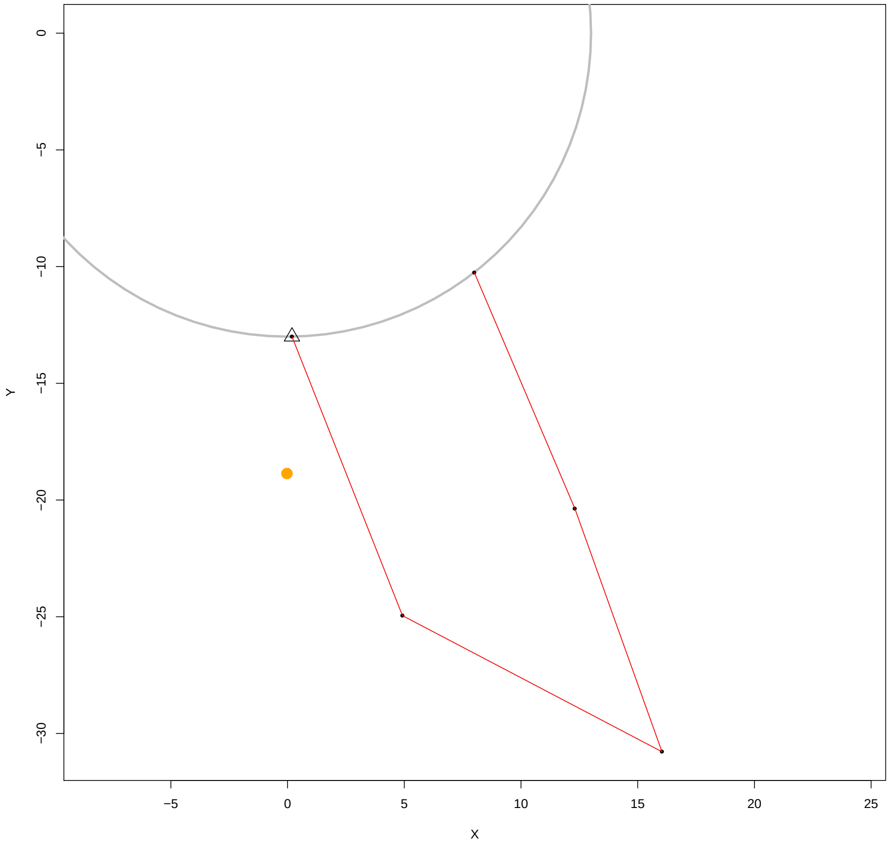

Fig SI-V-26.3 : Loop 168 — loop 3 of bee JU-1

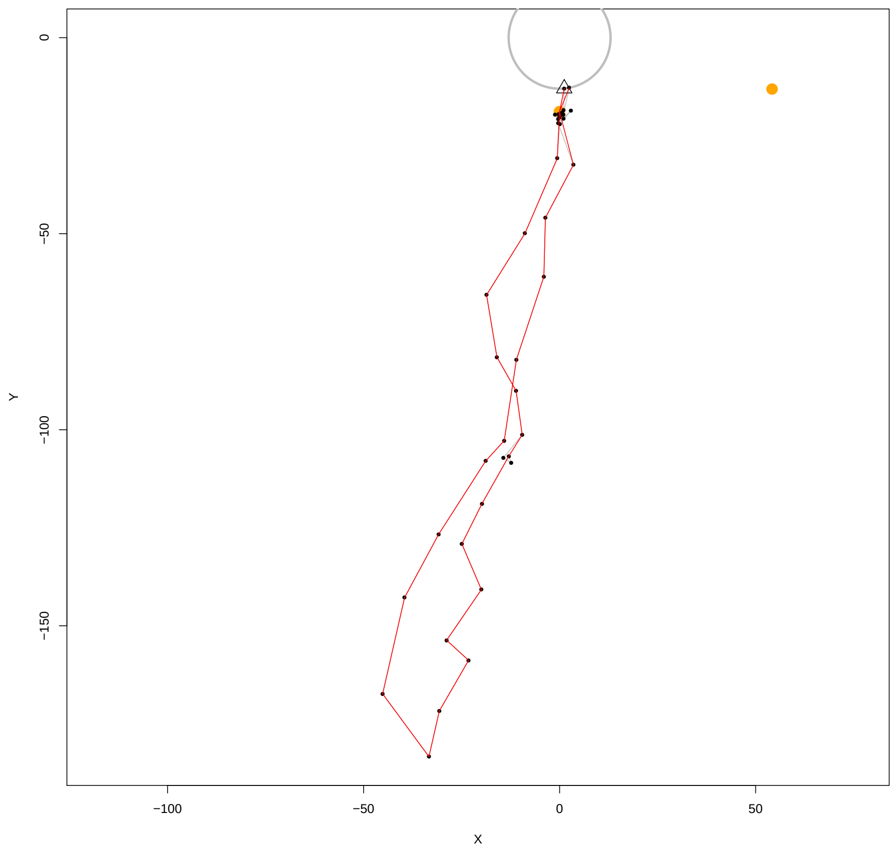

Fig SI-V-26.4 : Loop 169 — loop 4 of bee JU-1

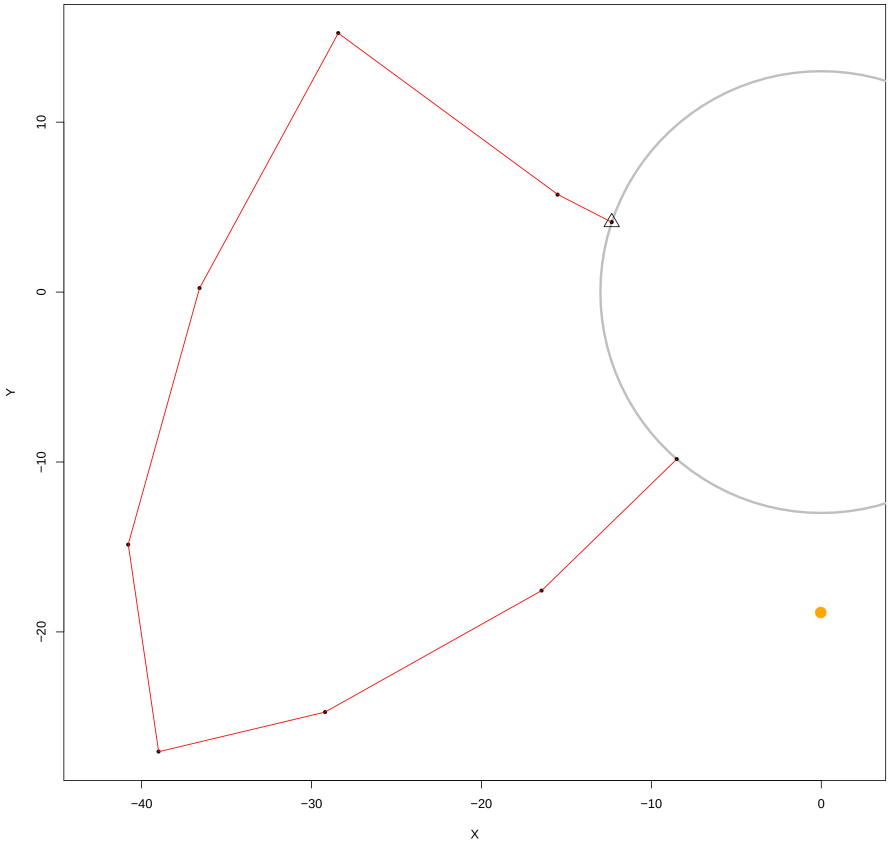

Fig SI-V-26.5 : Loop 170 — loop 5 of bee JU-1

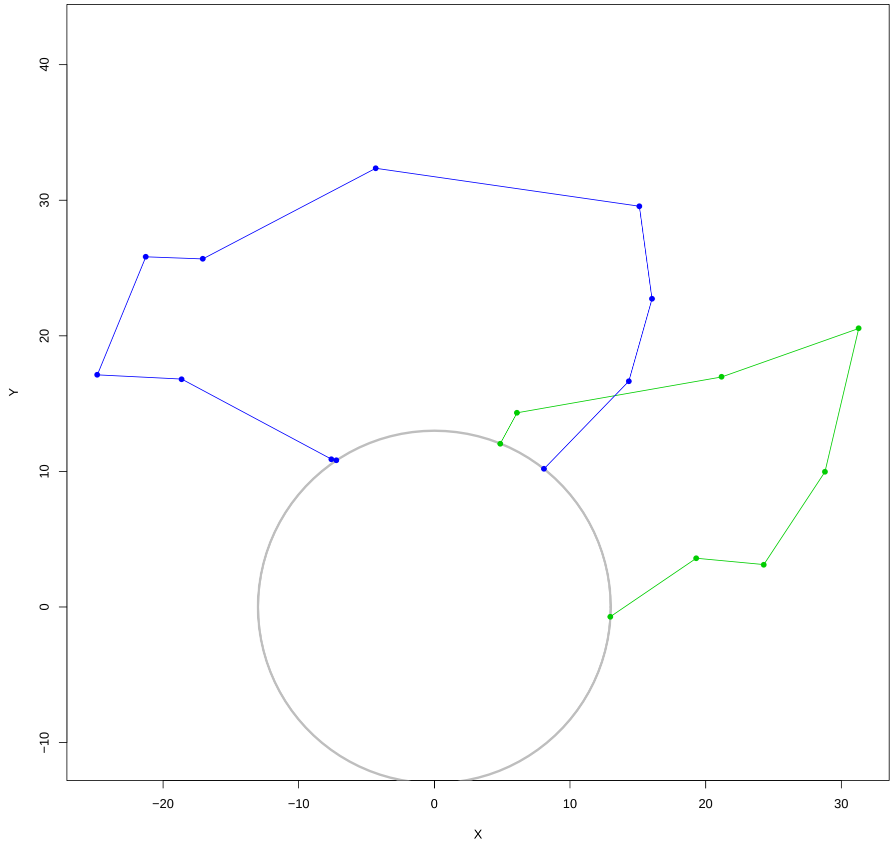

Fig SI-V-27 : Tracks of bee LL-1

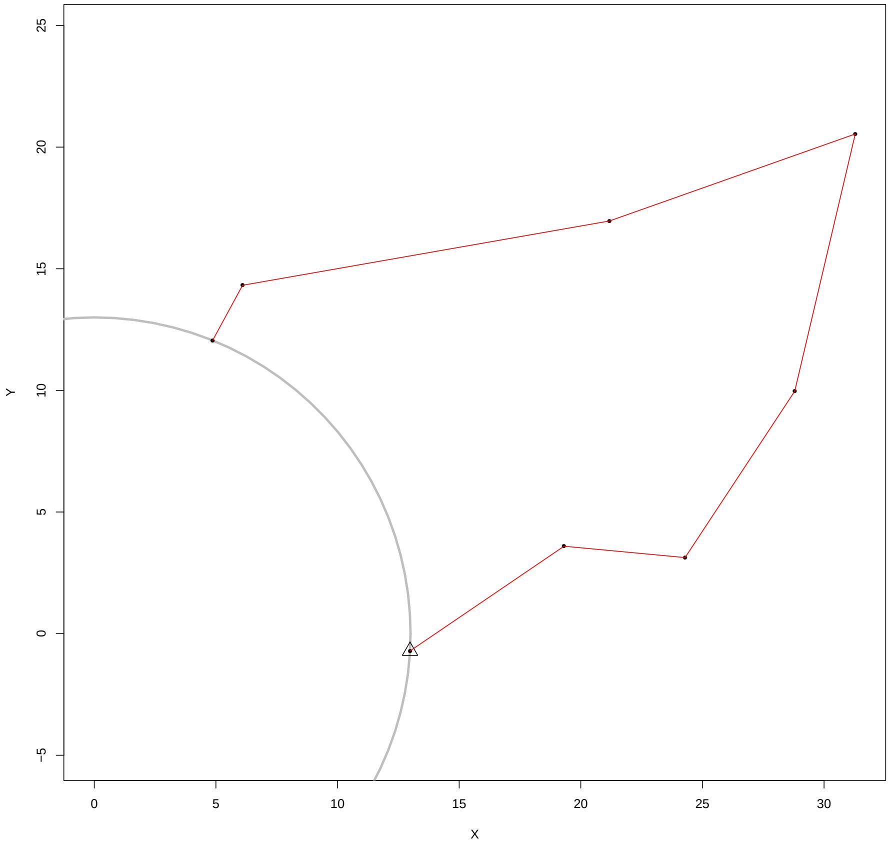

Fig SI-V-27.1 : Loop 171 — loop 1 of bee LL-1

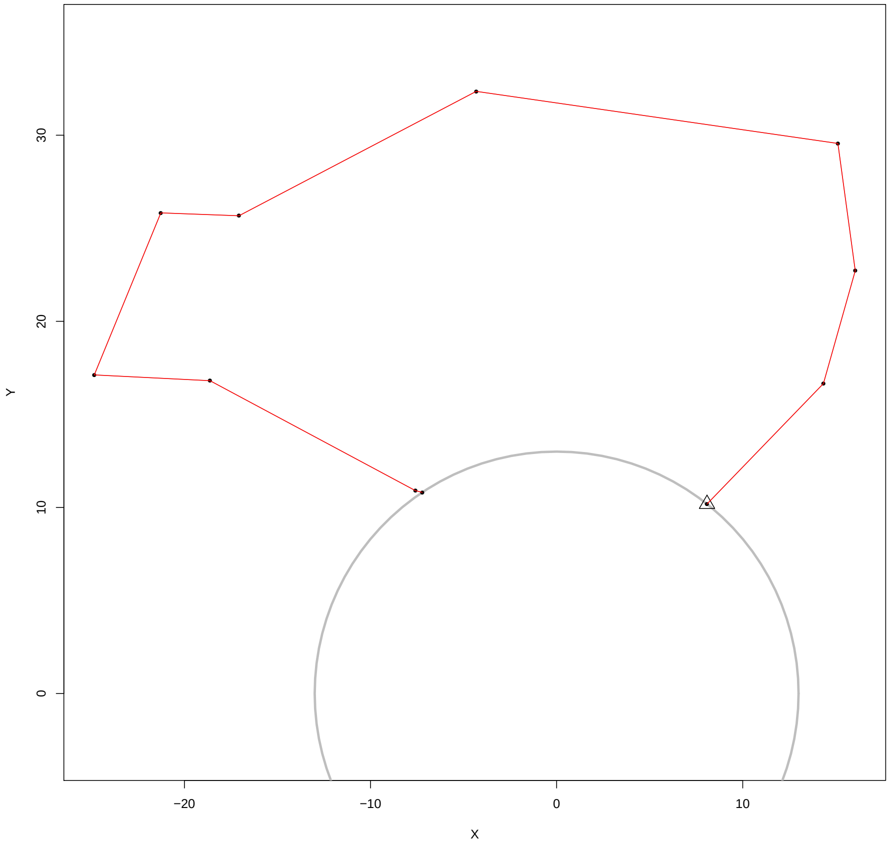

Fig SI-V-27.2 : Loop 172 — loop 2 of bee LL-1

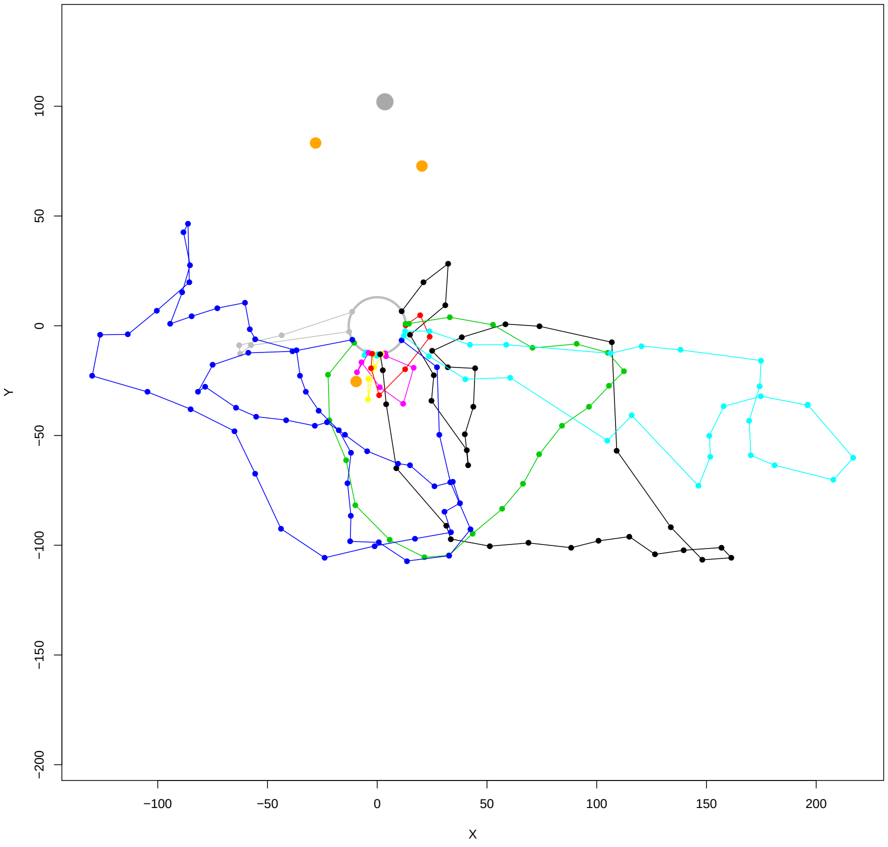

Fig SI-V-28 : Tracks of bee LE-1

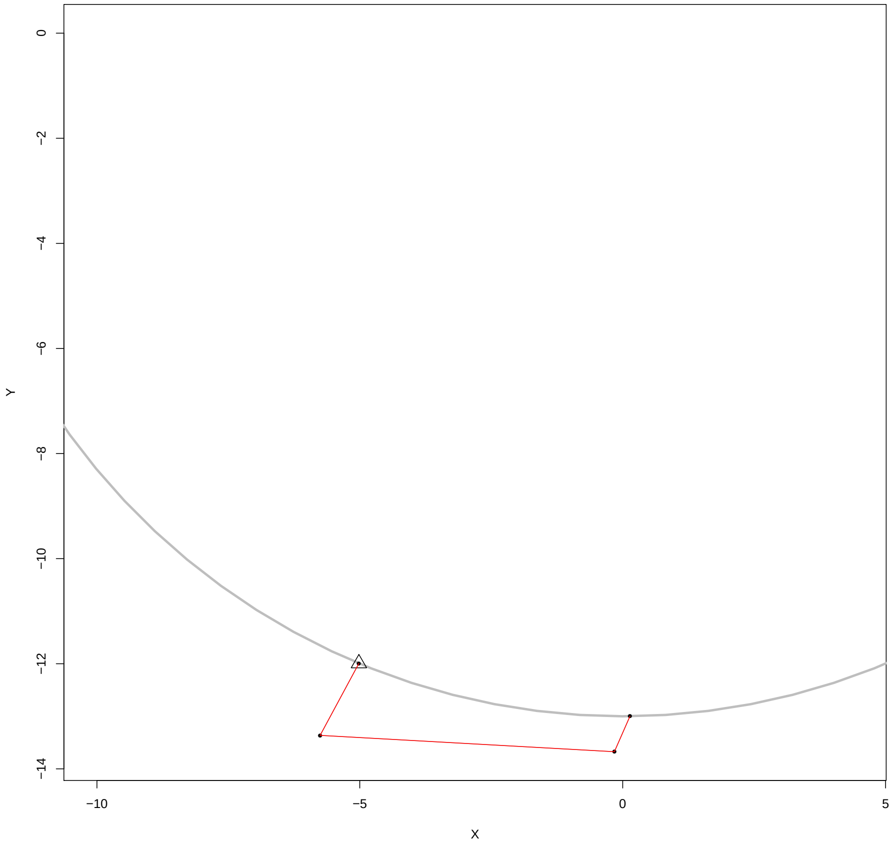

Fig SI-V-28.1 : Loop 173 — loop 1 of bee LE-1

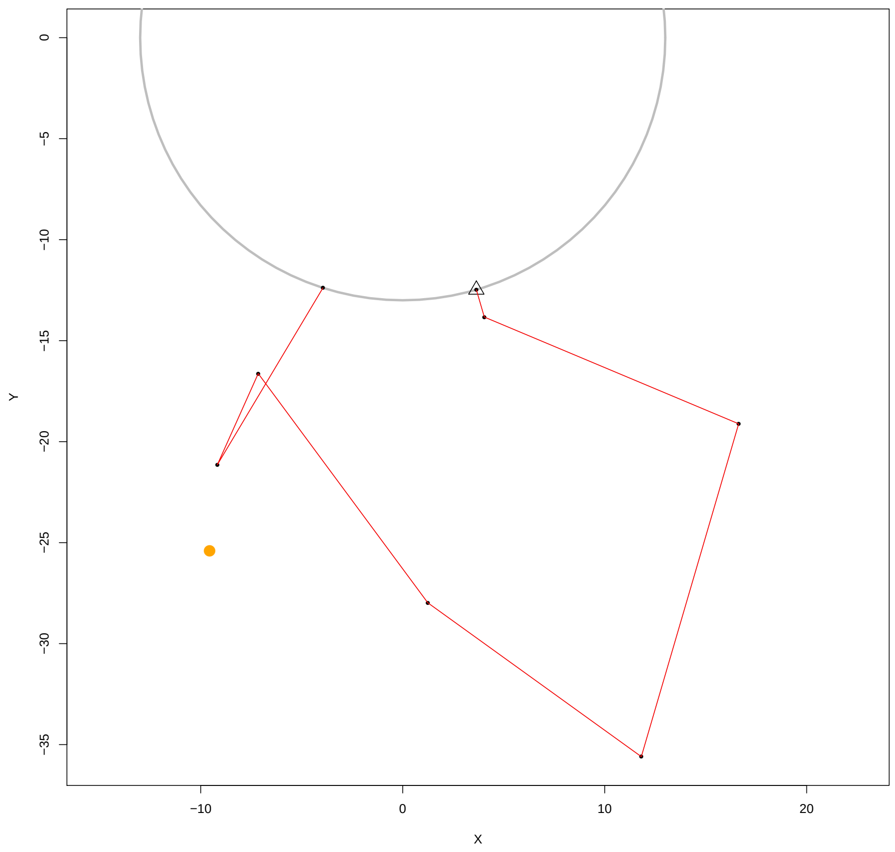

Fig SI-V-28.2 : Loop 174 — loop 2 of bee LE-1

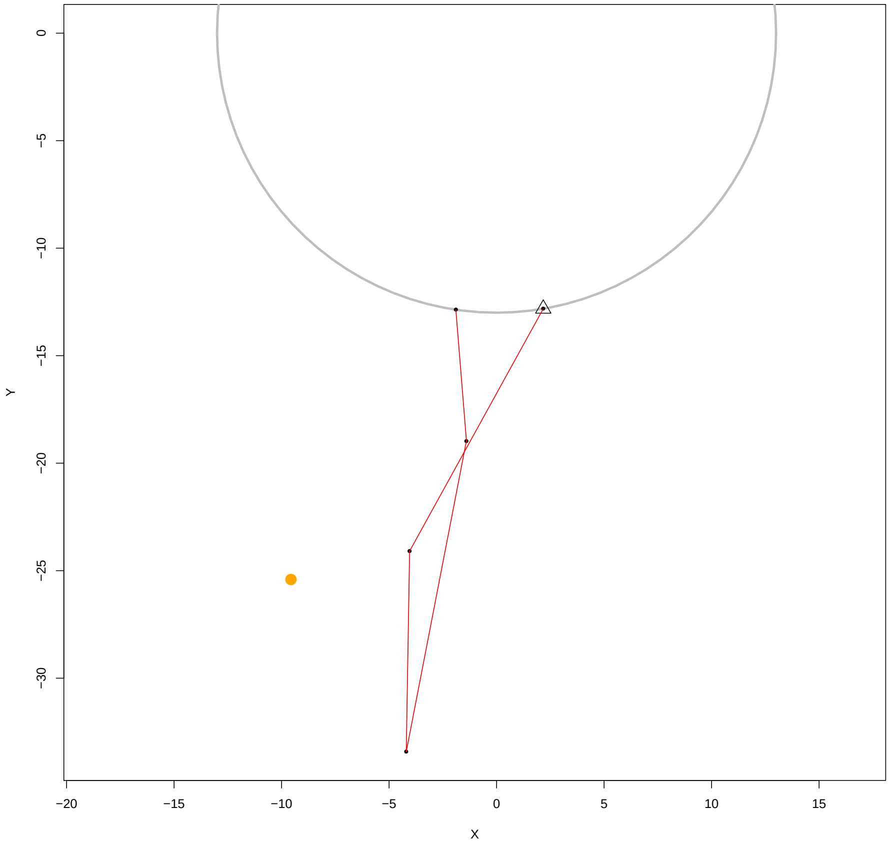

Fig SI-V-28.3 : Loop 175 — loop 3 of bee LE-1

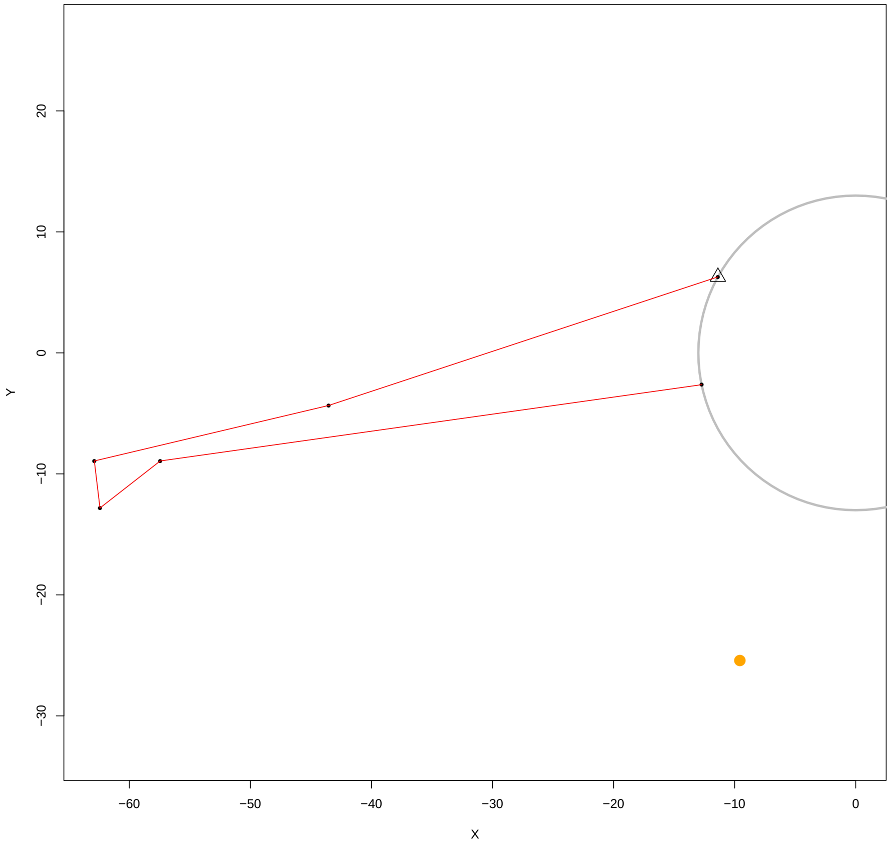

Fig SI-V-28.4 : Loop 176 — loop 4 of bee LE-1

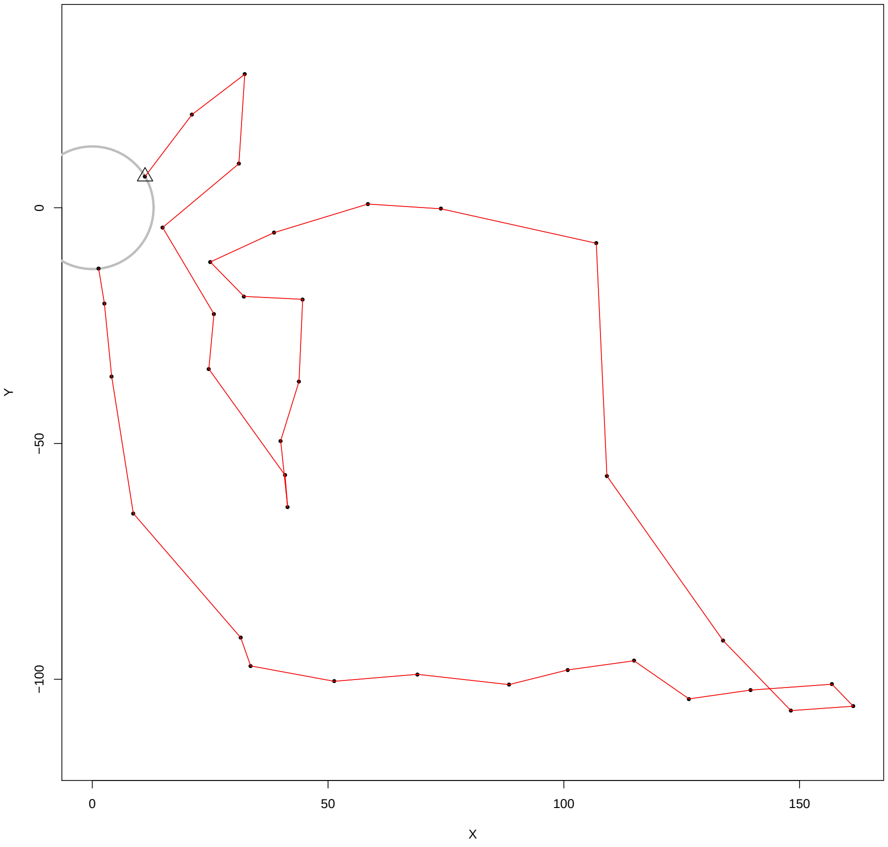

Fig SI-V-28.5 : Loop 177 — loop 5 of bee LE-1

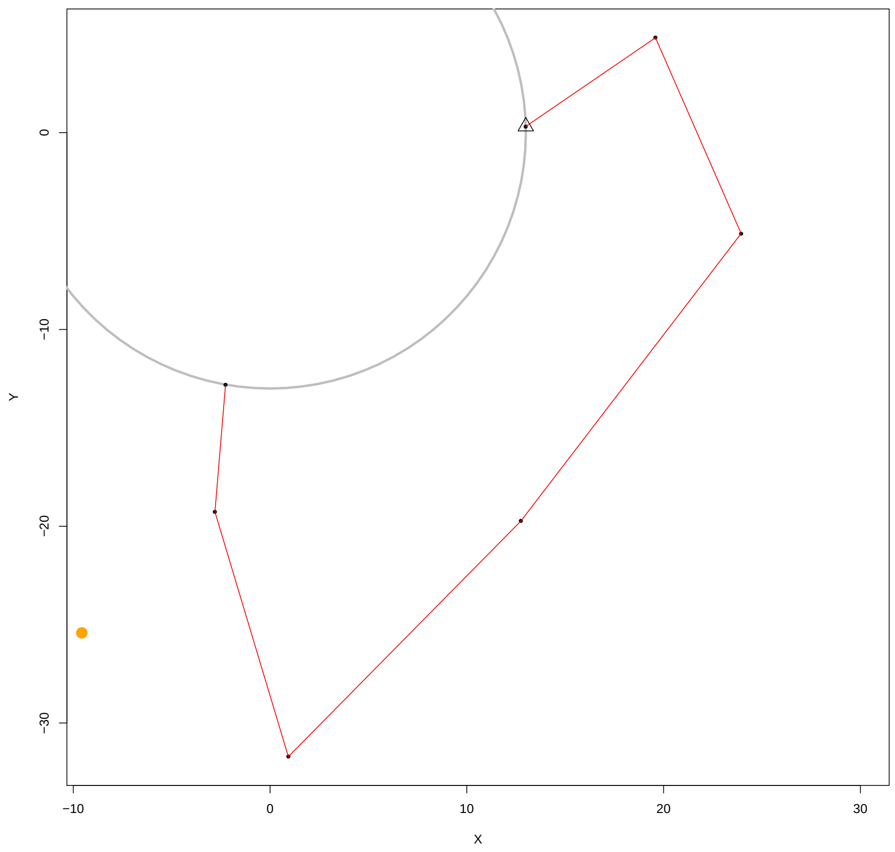

Fig SI-V-28.6 : Loop 178 — loop 6 of bee LE-1

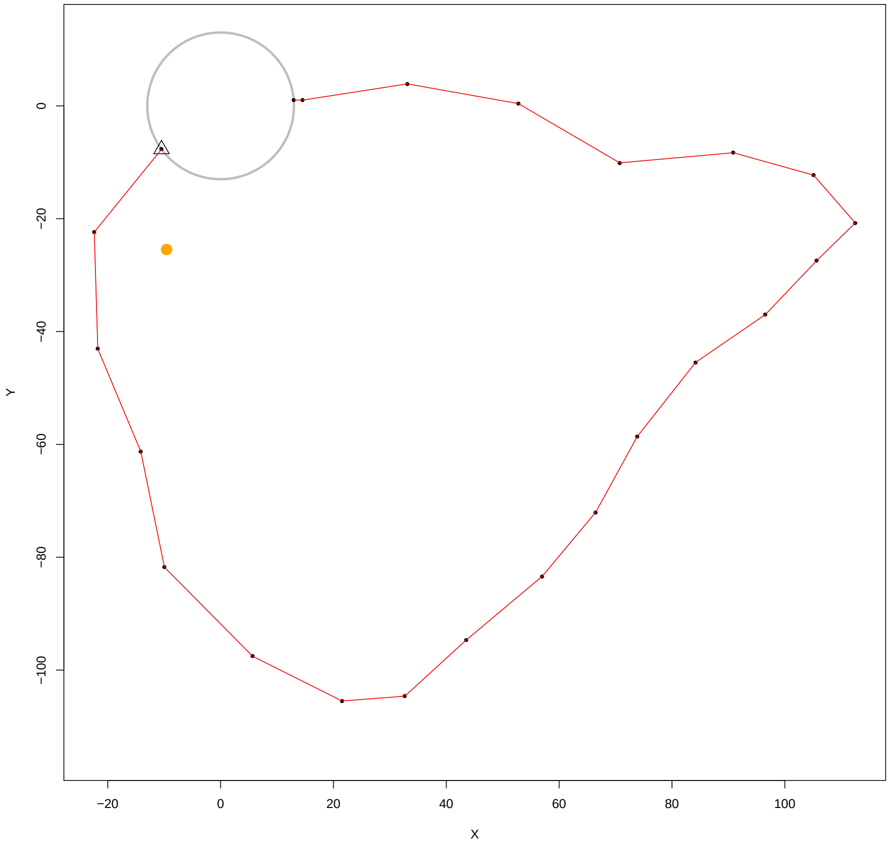

Fig SI-V-28.7 : Loop 179 — loop 7 of bee LE-1

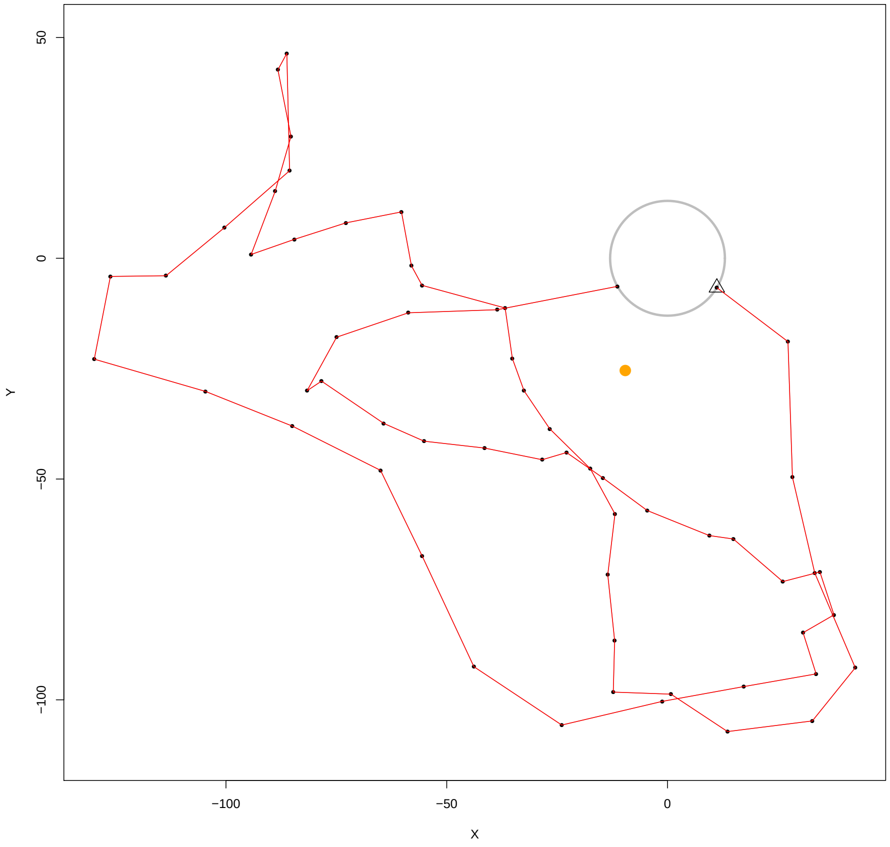

Fig SI-V-28.8 : Loop 180 — loop 8 of bee LE-1

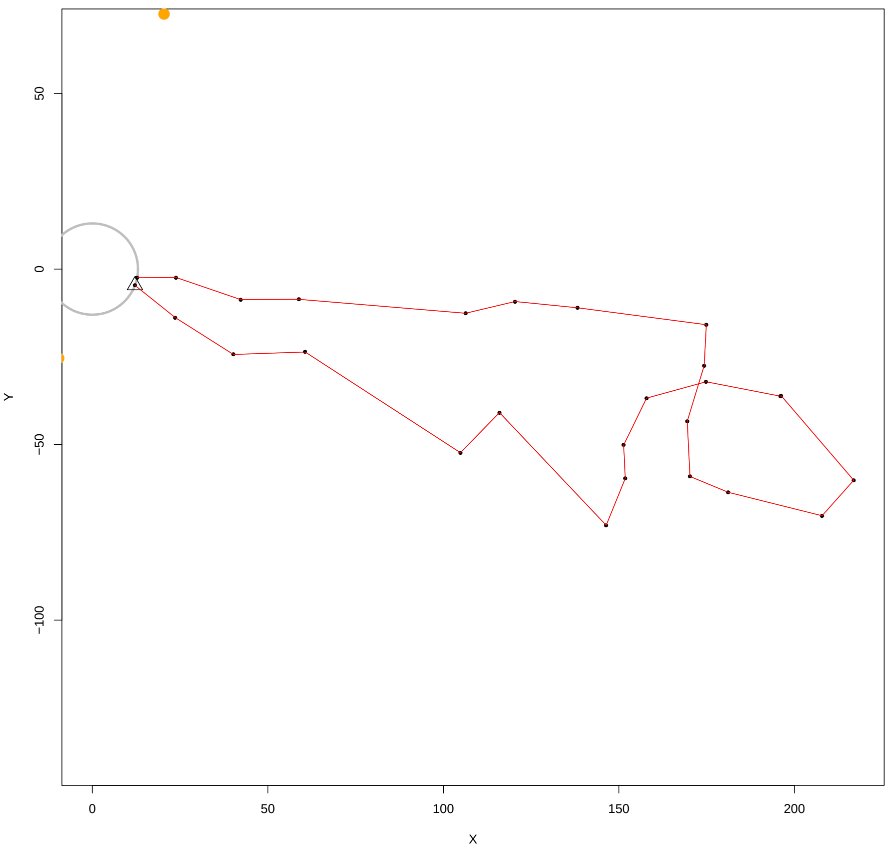

Fig SI-V-28.9 : Loop 181 — loop 9 of bee LE-1

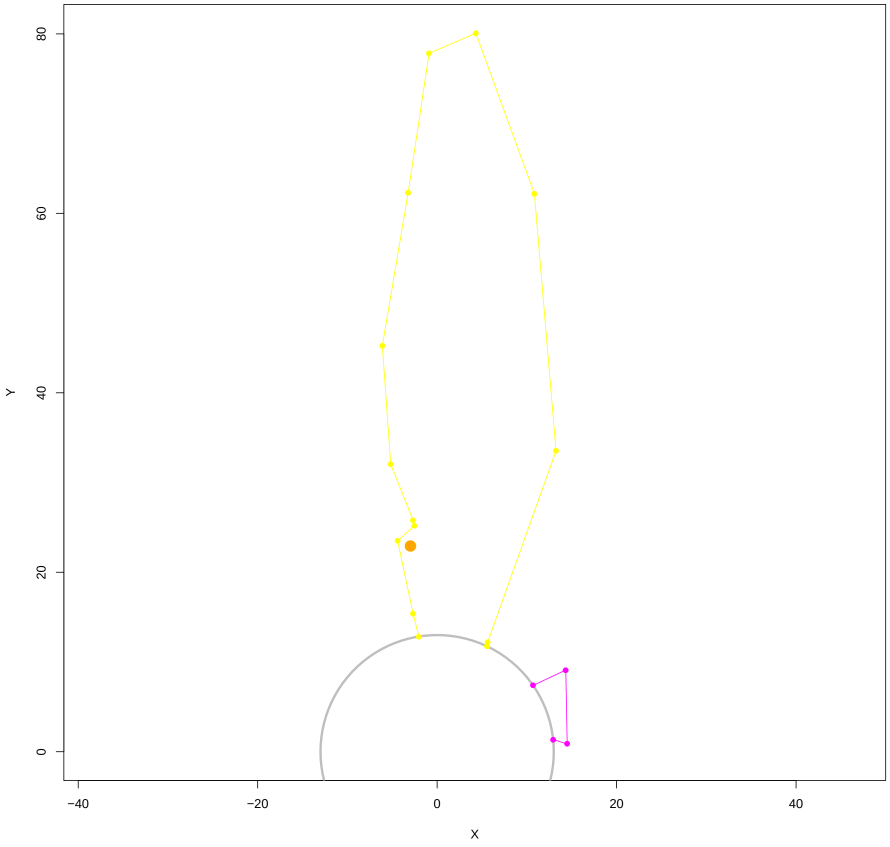

Fig SI-V-29 : Tracks of bee GX-1

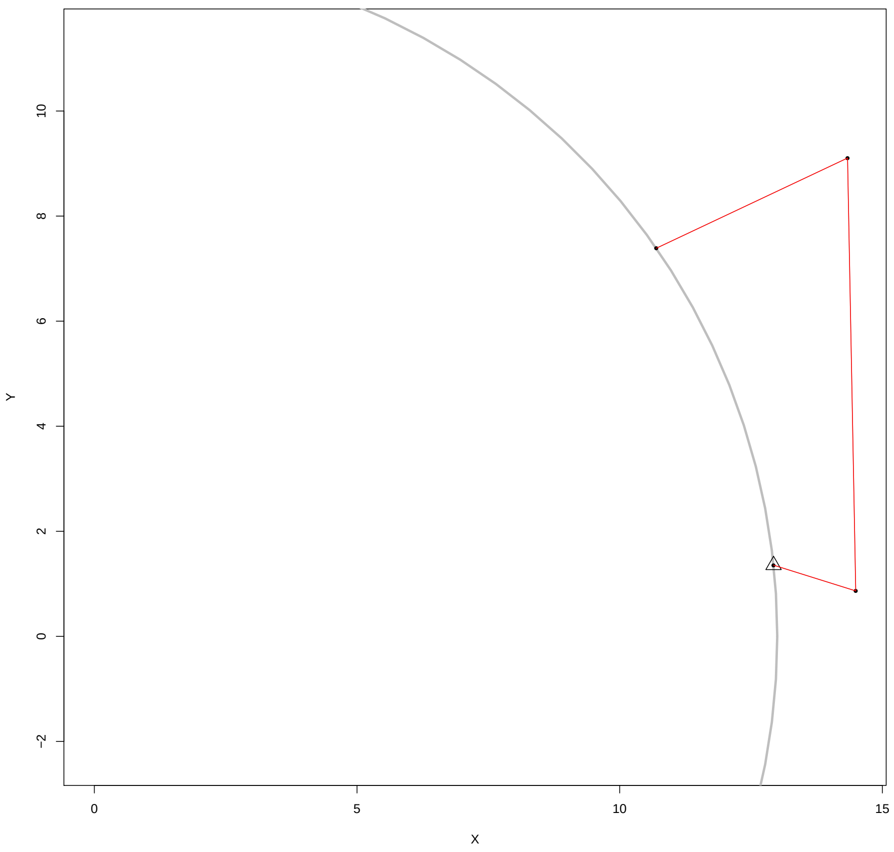

Fig SI-V-29.1 : Loop 182 — loop 1 of bee GX-1

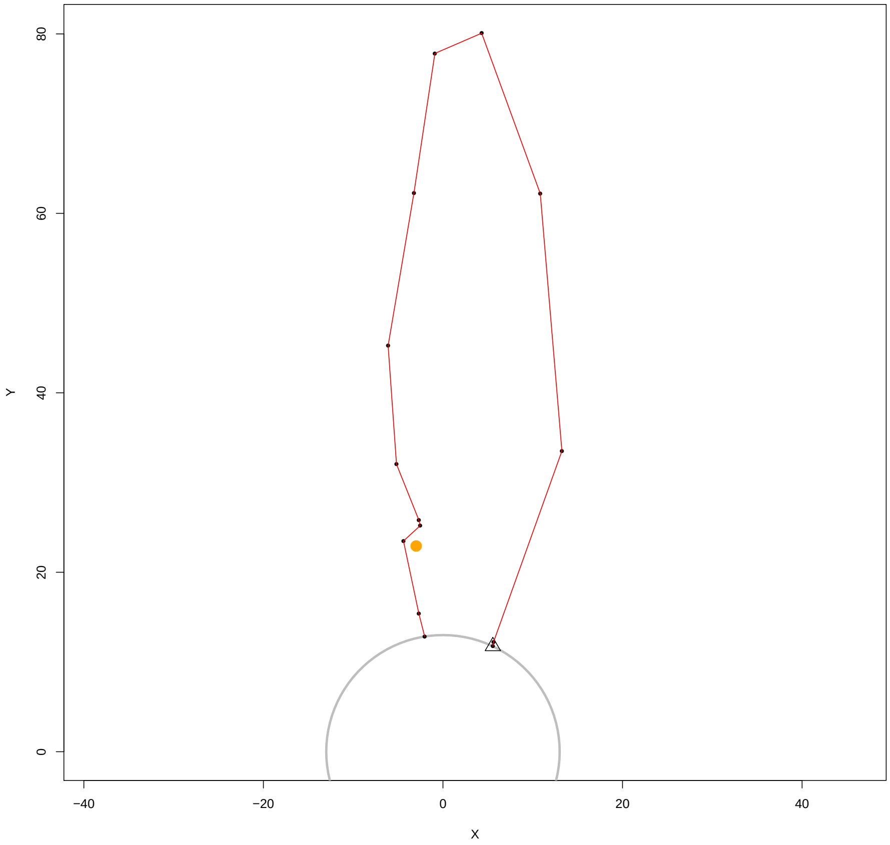

Fig SI-V-29.2 : Loop 183 — loop 2 of bee GX-1

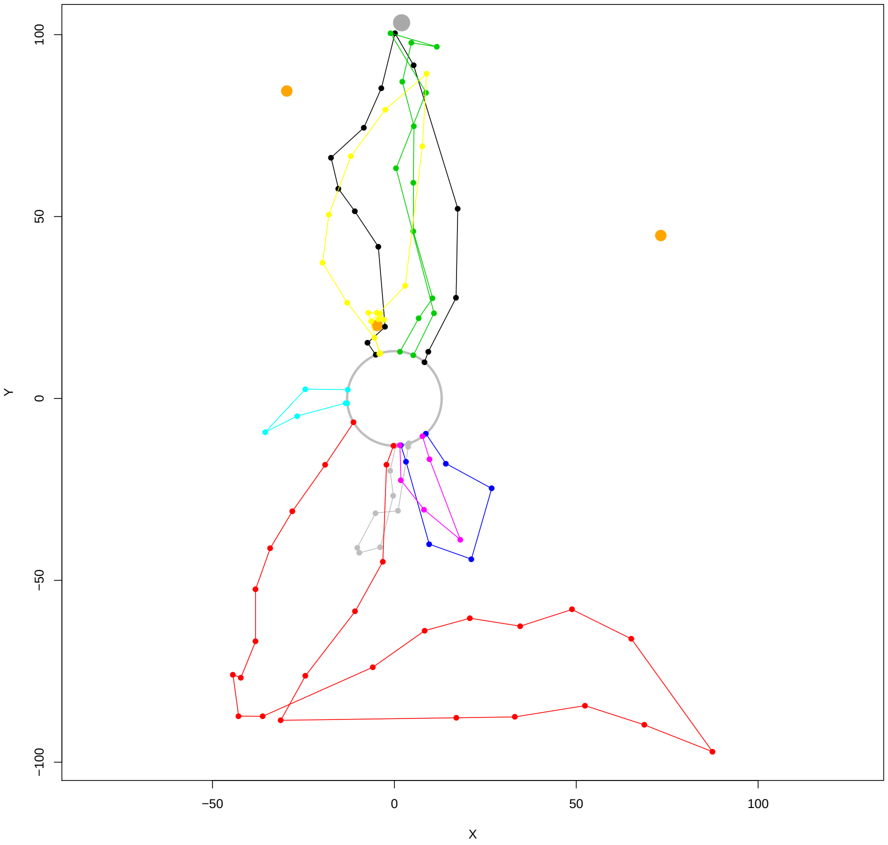

Fig SI-V-30 : Tracks of bee KO-1

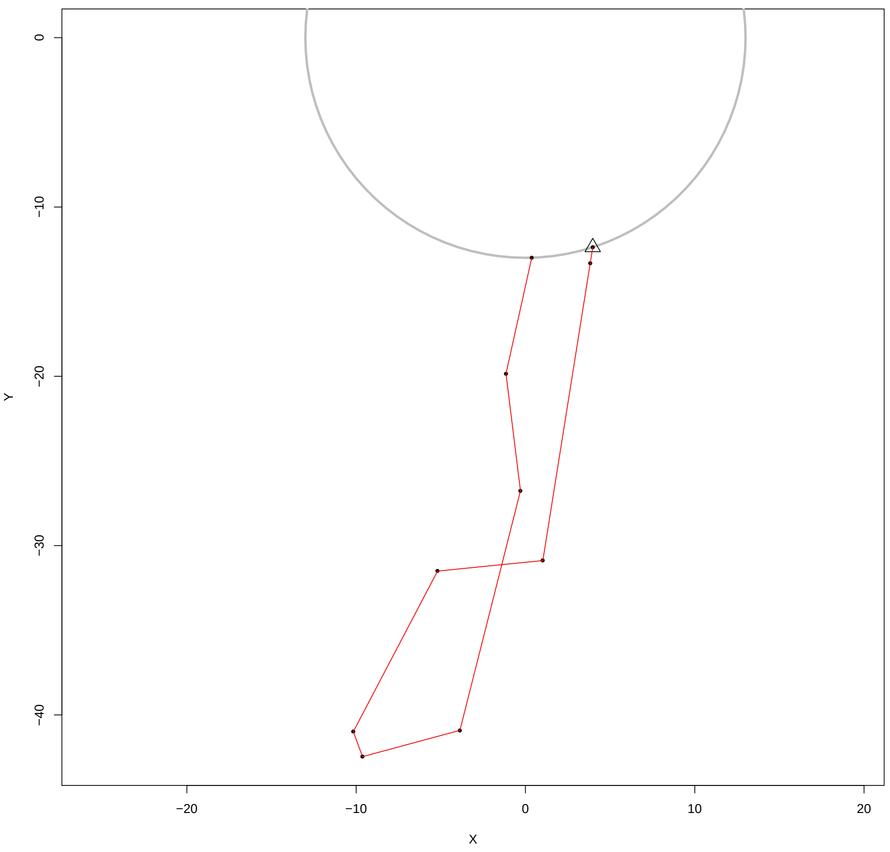

Fig SI-V-30.1 : Loop 184 — loop 1 of bee KO-1

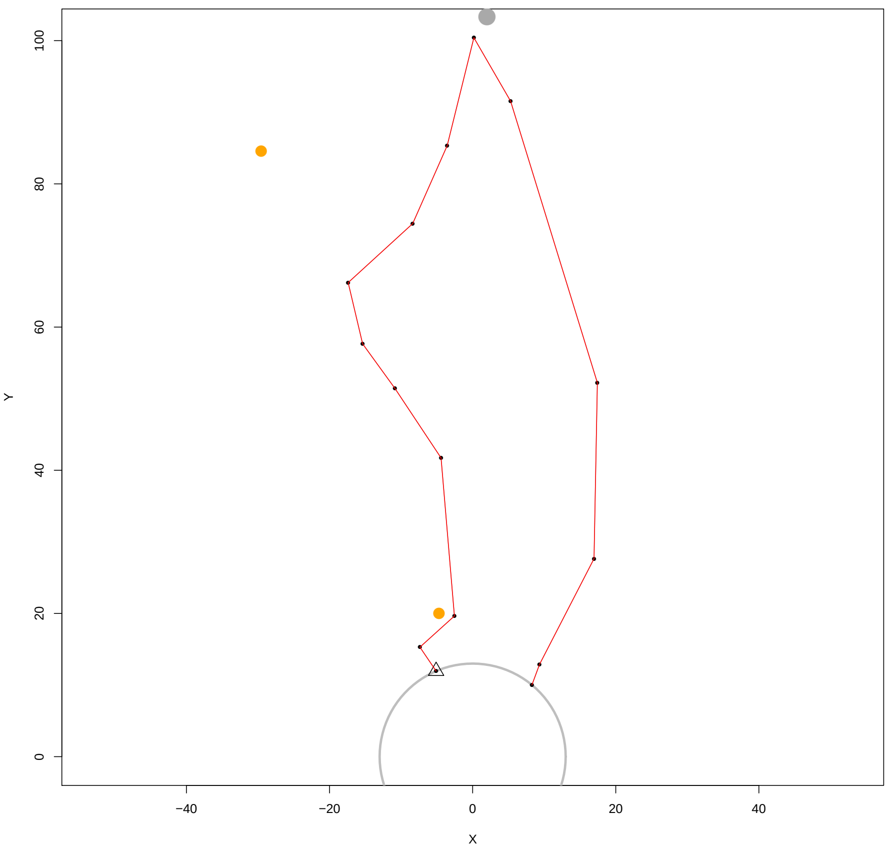

Fig SI-V-30.2 : Loop 185 — loop 2 of bee KO-1

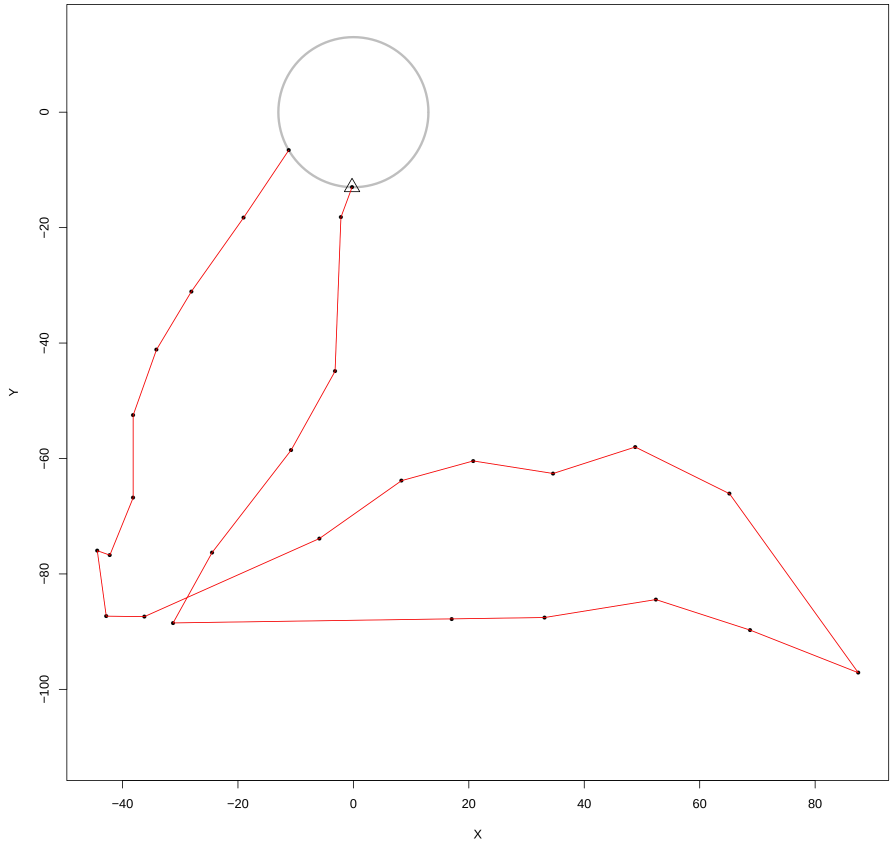

Fig SI-V-30.3 : Loop 186 — loop 3 of bee KO-1

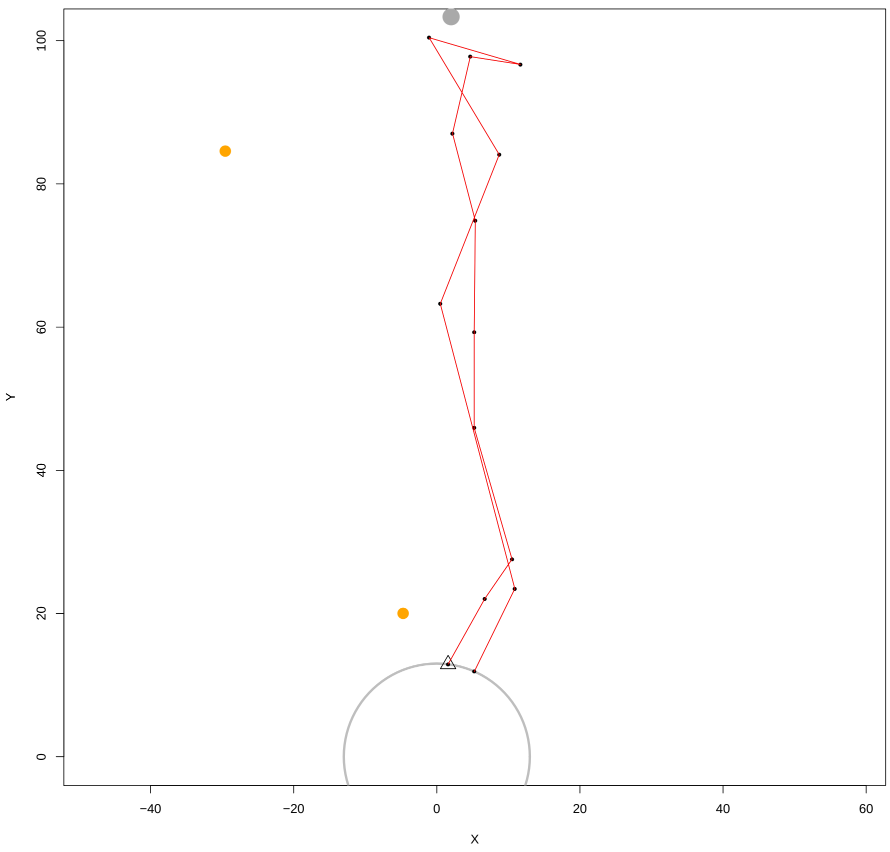

Fig SI-V-30.4 : Loop 187 — loop 4 of bee KO-1

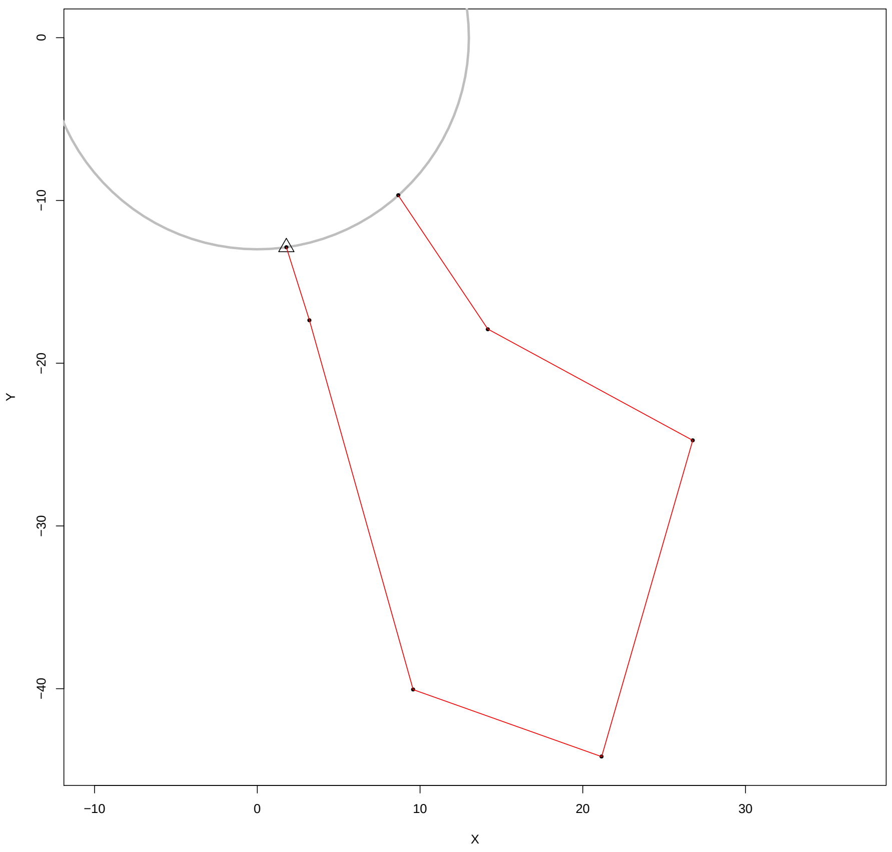

Fig SI-V-30.5 : Loop 188 — loop 5 of bee KO-1

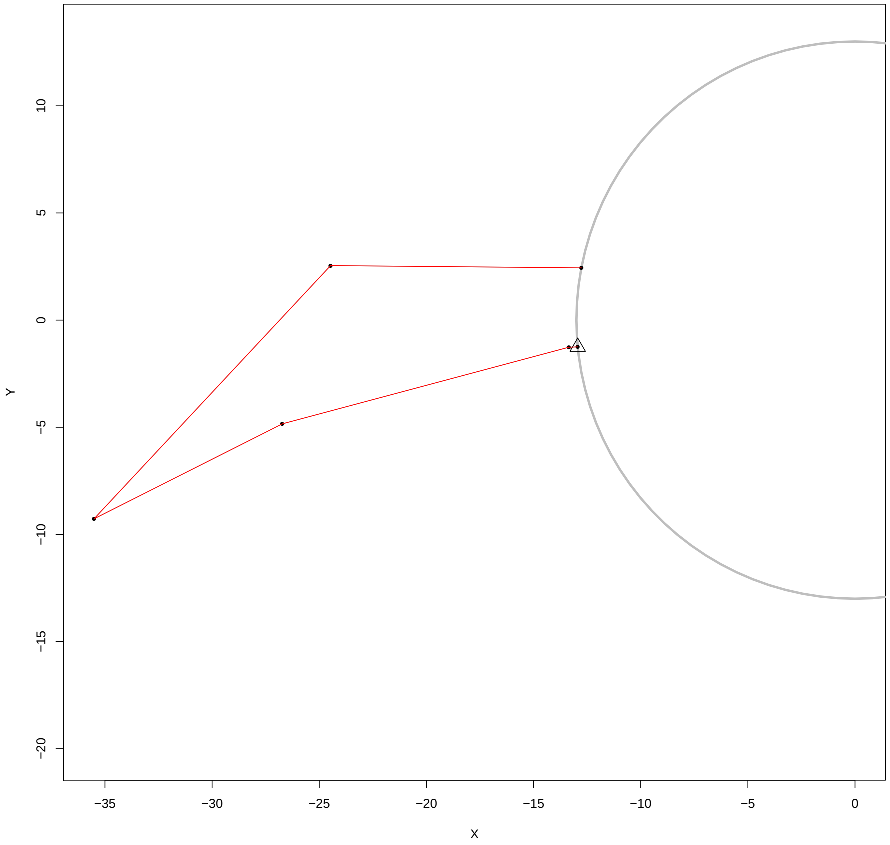

Fig SI-V-30.6 : Loop 189 — loop 6 of bee KO-1

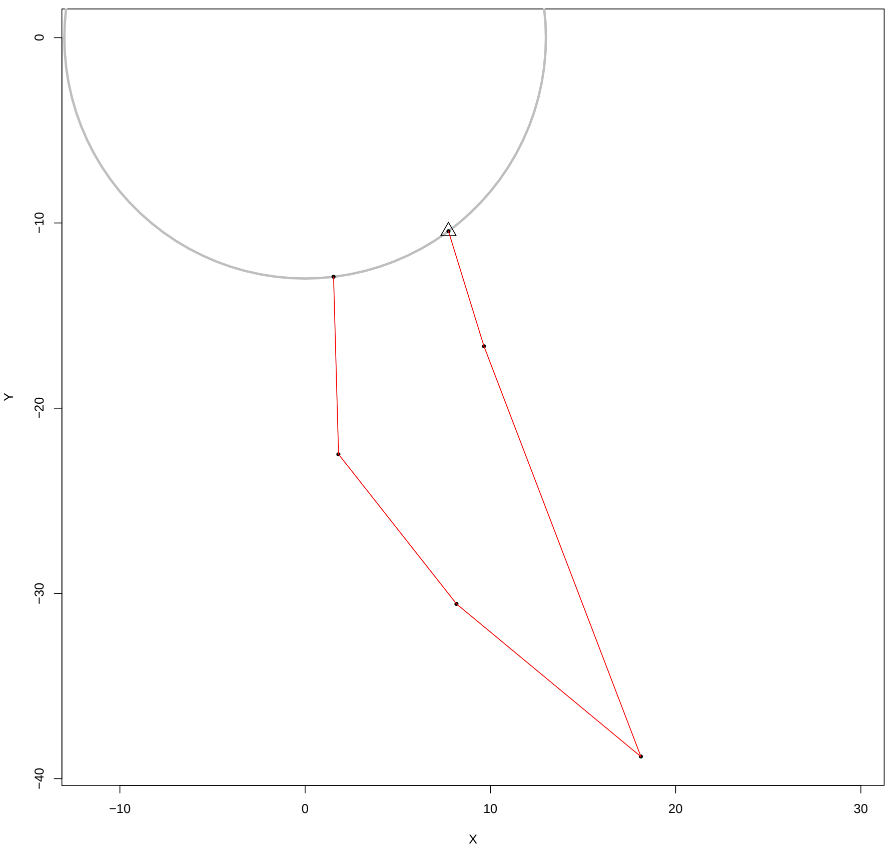

Fig SI-V-30.7 : Loop 190 — loop 7 of bee KO-1

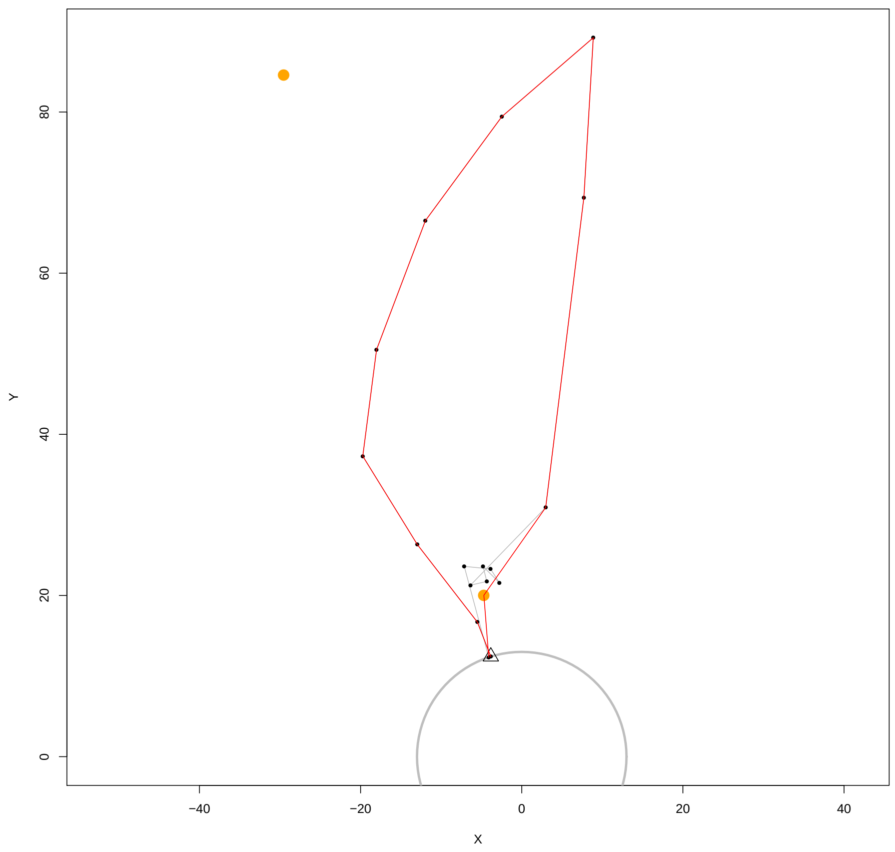

Fig SI-V-30.8 : Loop 191 — loop 8 of bee KO-1

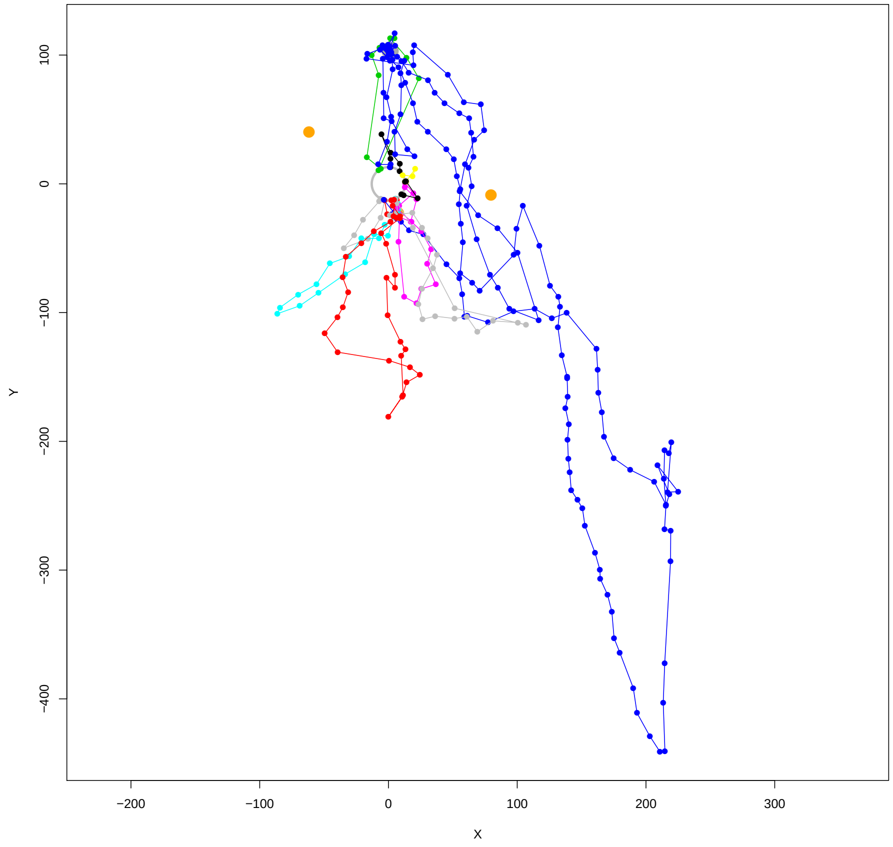

Fig SI-V-31 : Tracks of bee KH-2

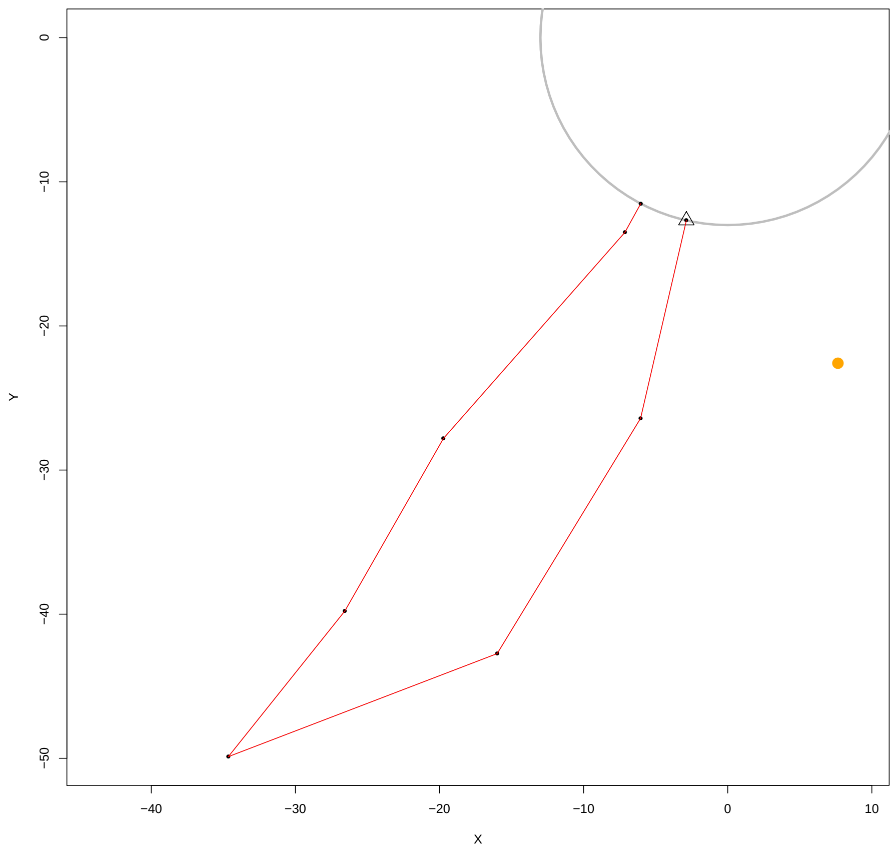

Fig SI-V-31.1 : Loop 192 — loop 1 of bee KH-2

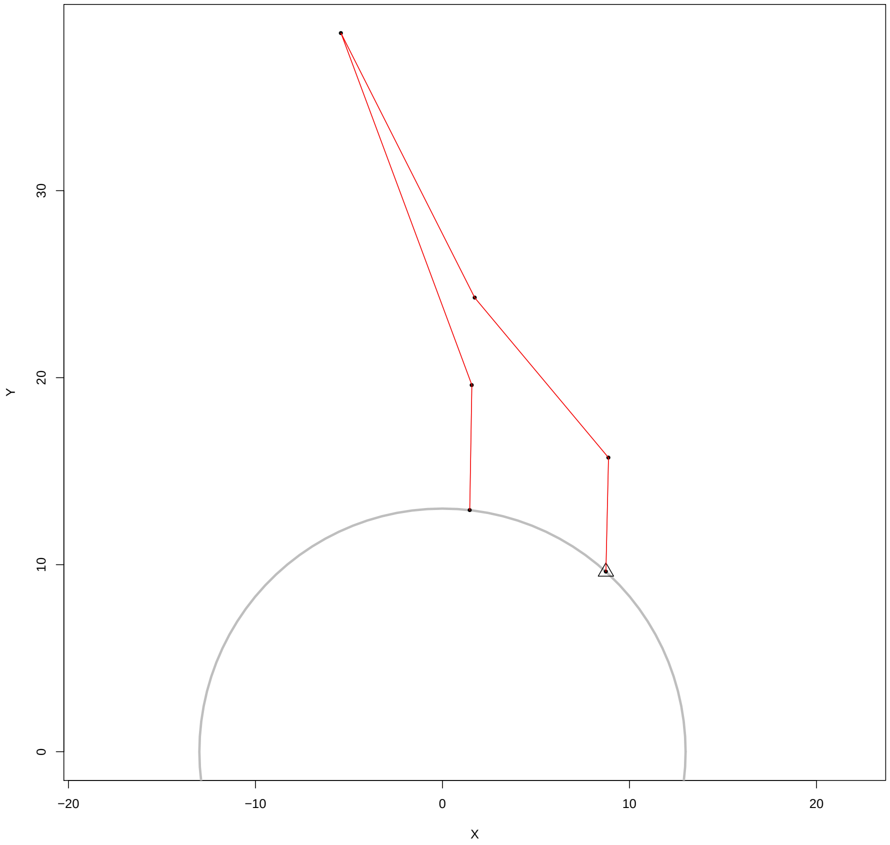

Fig SI-V-31.2 : Loop 193 — loop 2 of bee KH-2

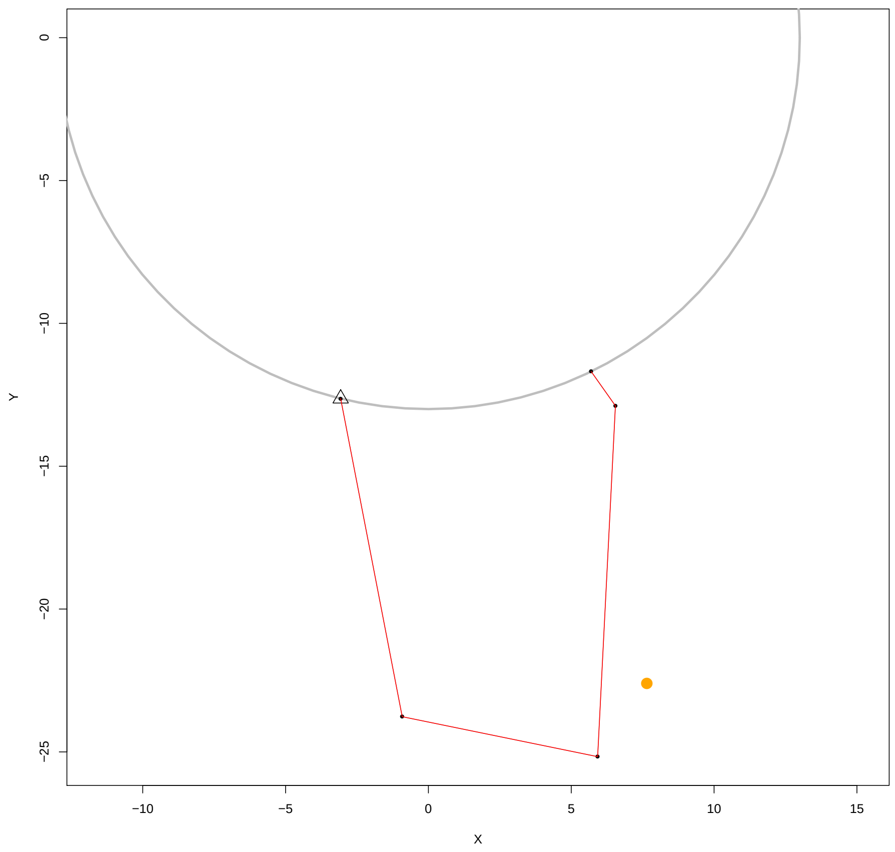

Fig SI-V-31.3 : Loop 194 — loop 3 of bee KH-2

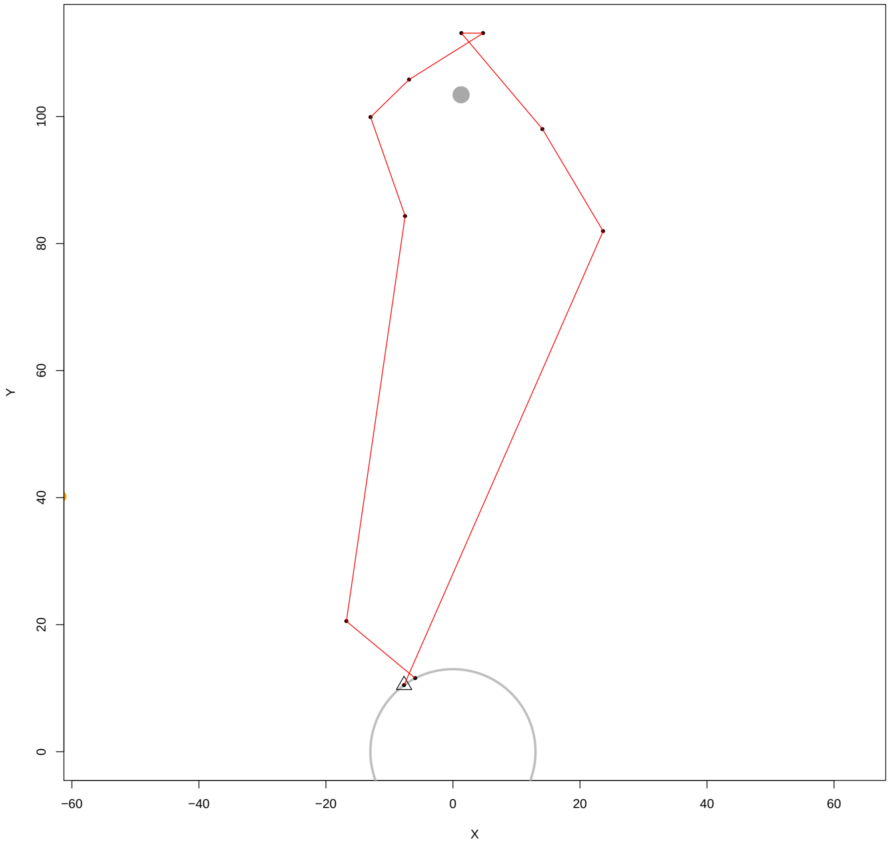

Fig SI-V-31.4 : Loop 195 — loop 4 of bee KH-2

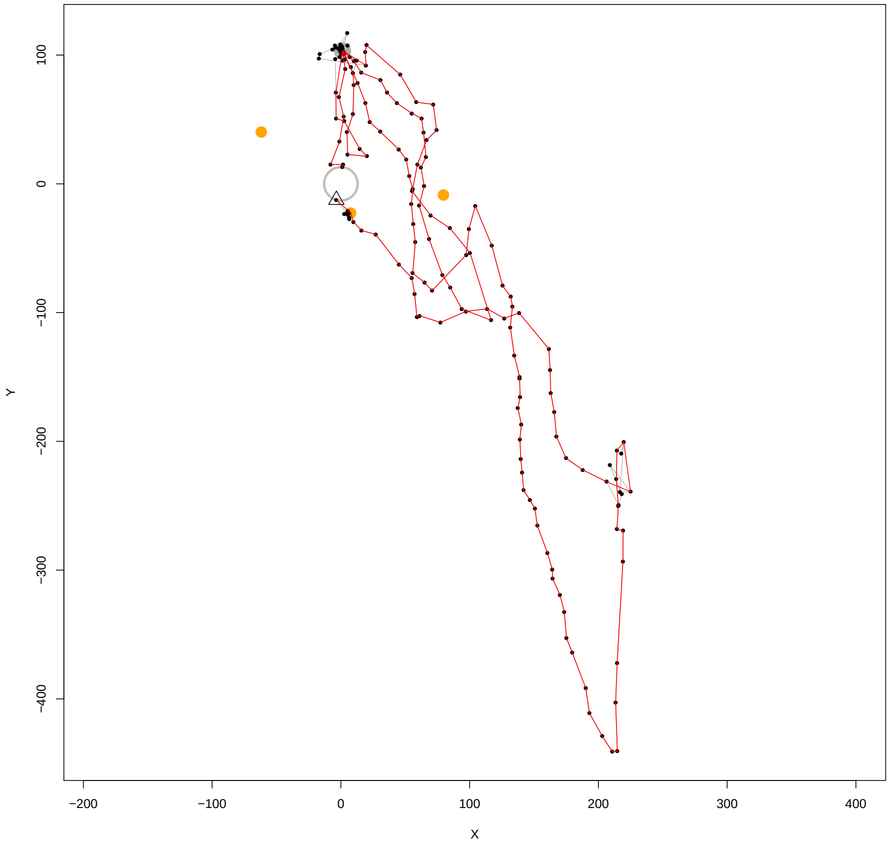

Fig SI-V-31.5 : Loop 196 — loop 5 of bee KH-2

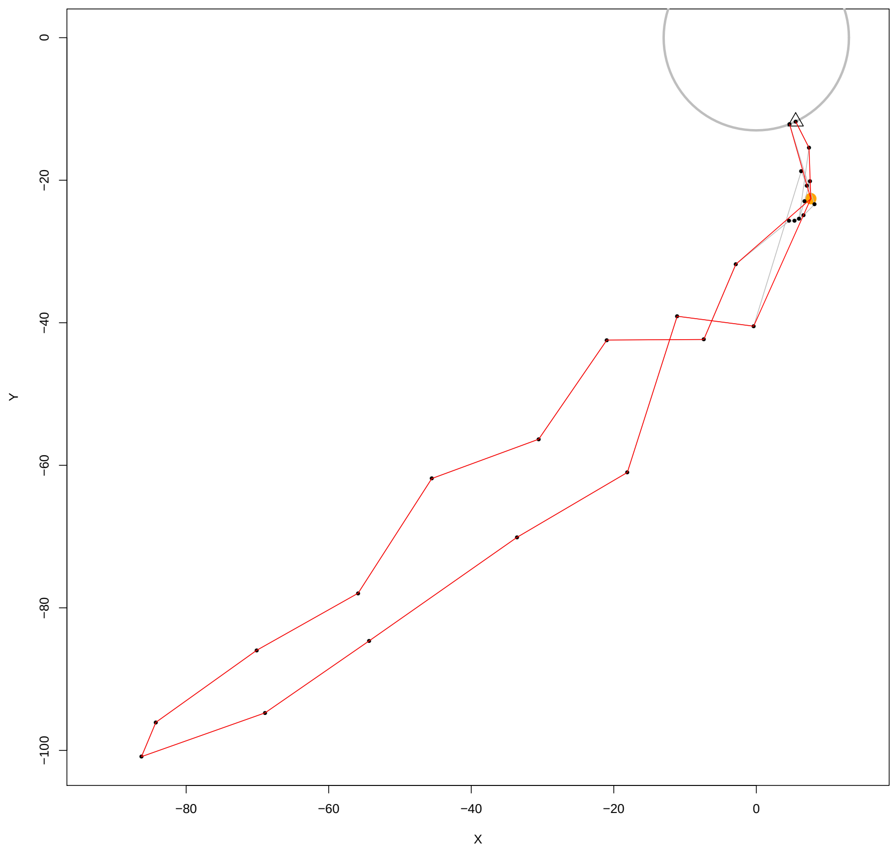

Fig SI-V-31.6 : Loop 197 — loop 6 of bee KH-2

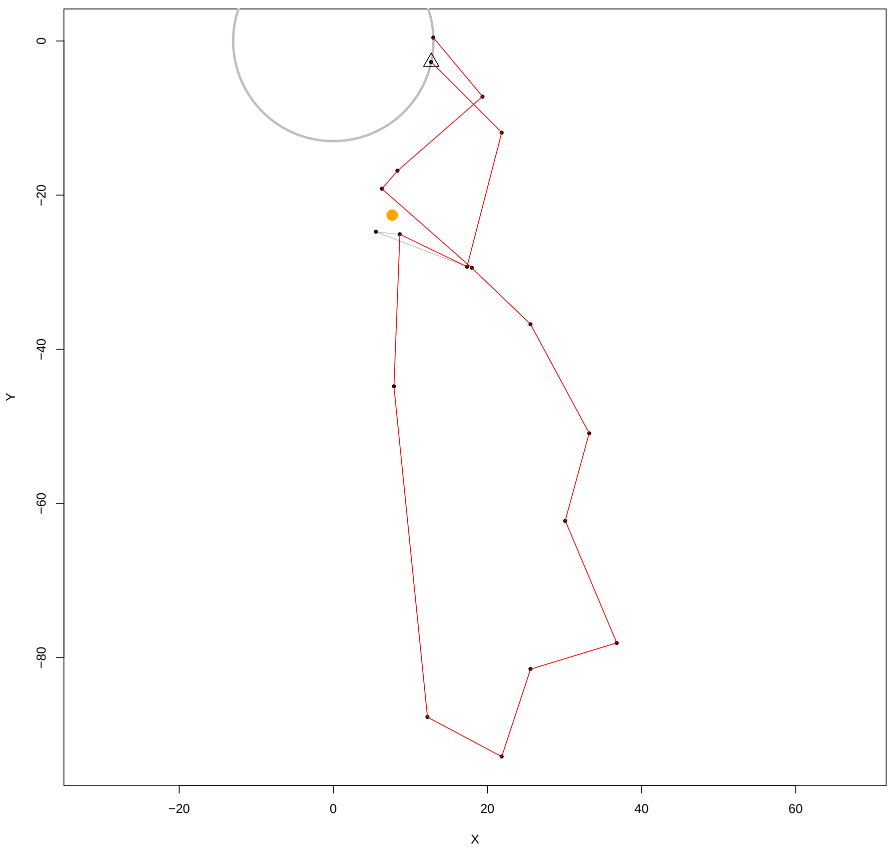

Fig SI-V-31.7 : Loop 198 — loop 7 of bee KH-2

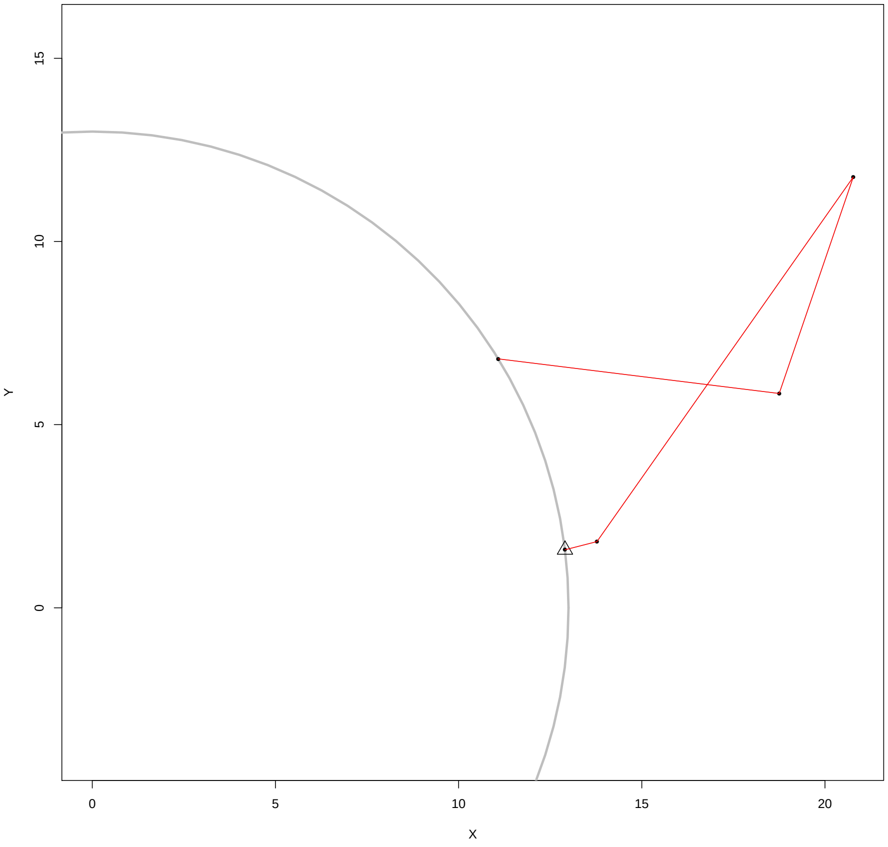

Fig SI-V-31.8 : Loop 199 — loop 8 of bee KH-2

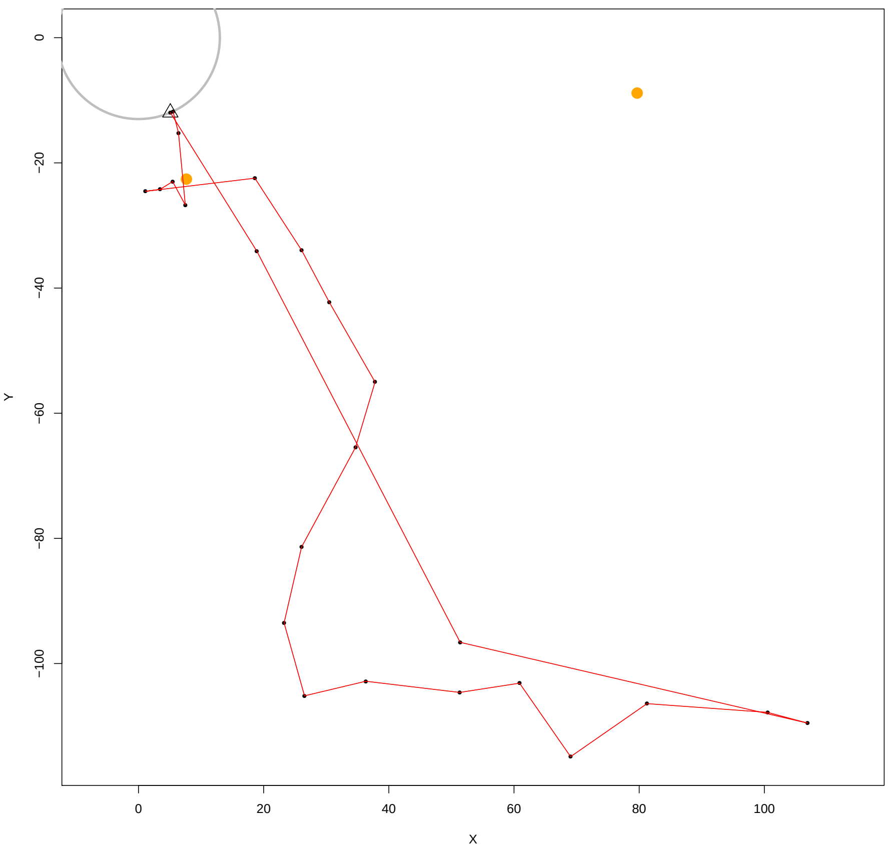

Fig SI-V-31.9 : Loop 200 — loop 9 of bee KH-2

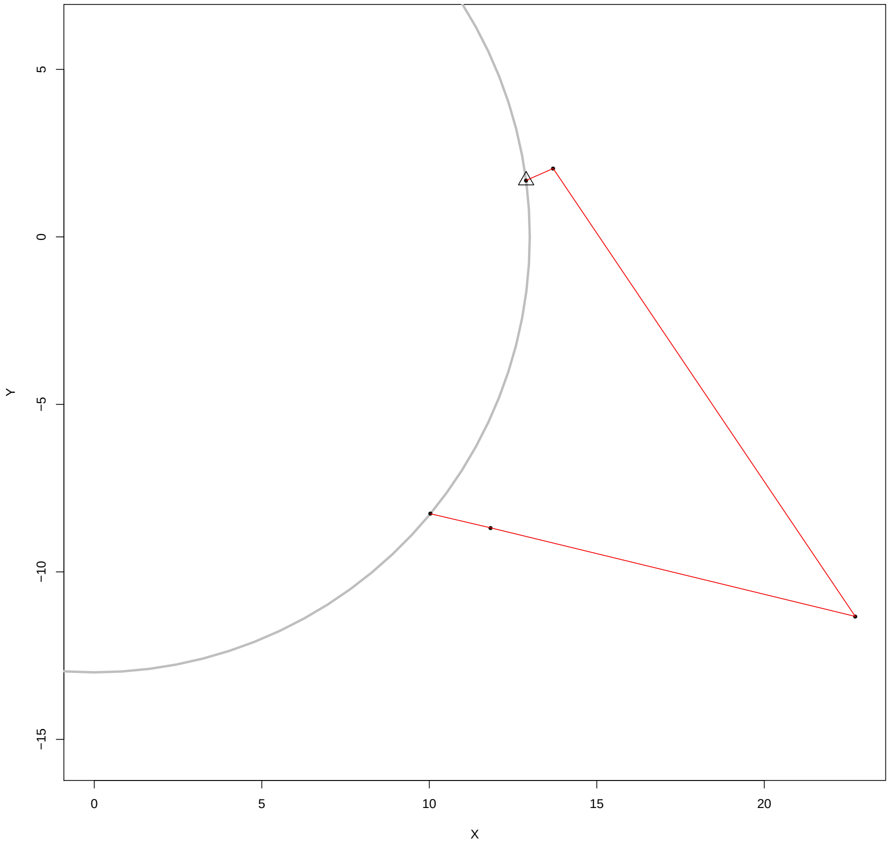

Fig SI-V-31.10 : Loop 201 — loop 10 of bee KH-2

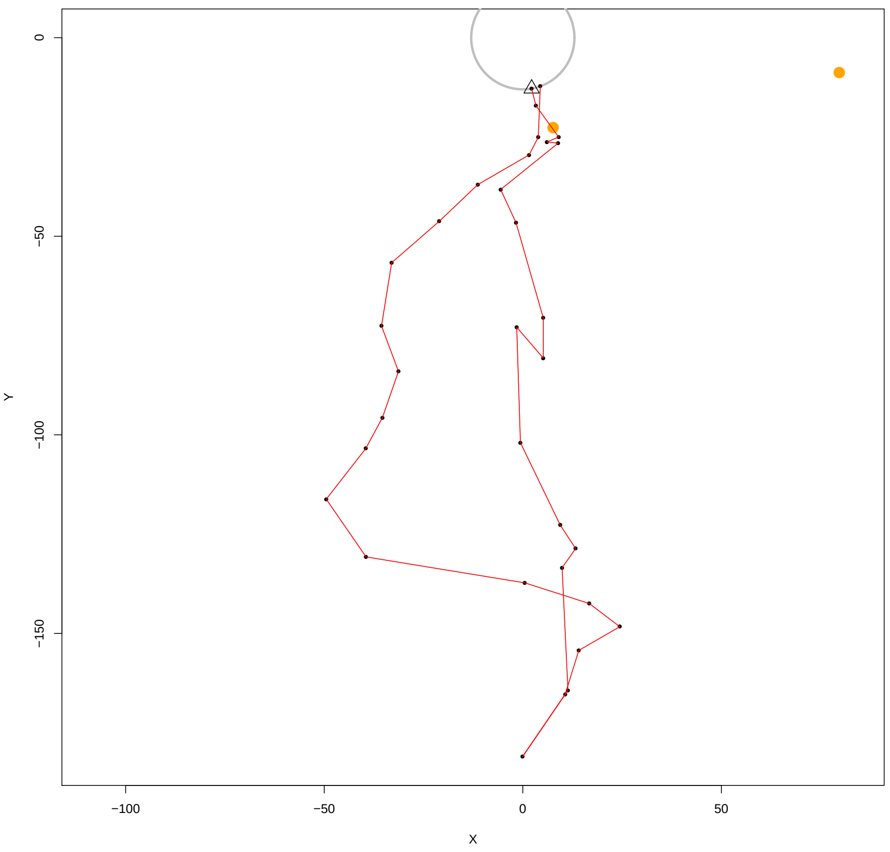

Fig SI-V-31.11 : Loop 202 — loop 11 of bee KH-2

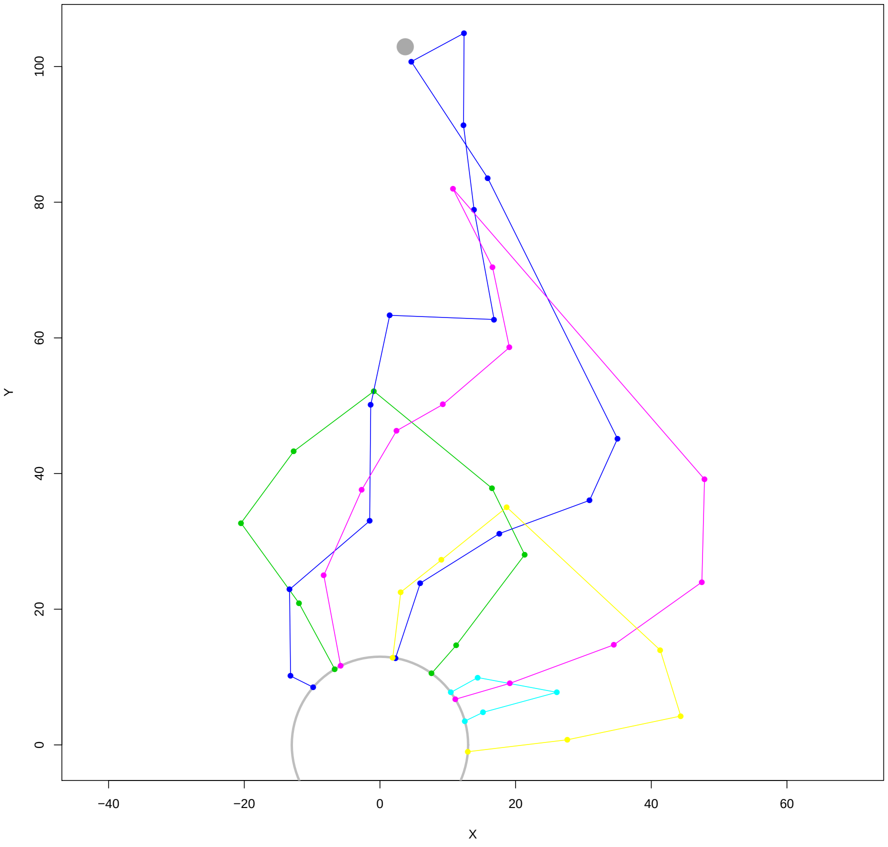

Fig SI-V-32 : Tracks of bee JY-1

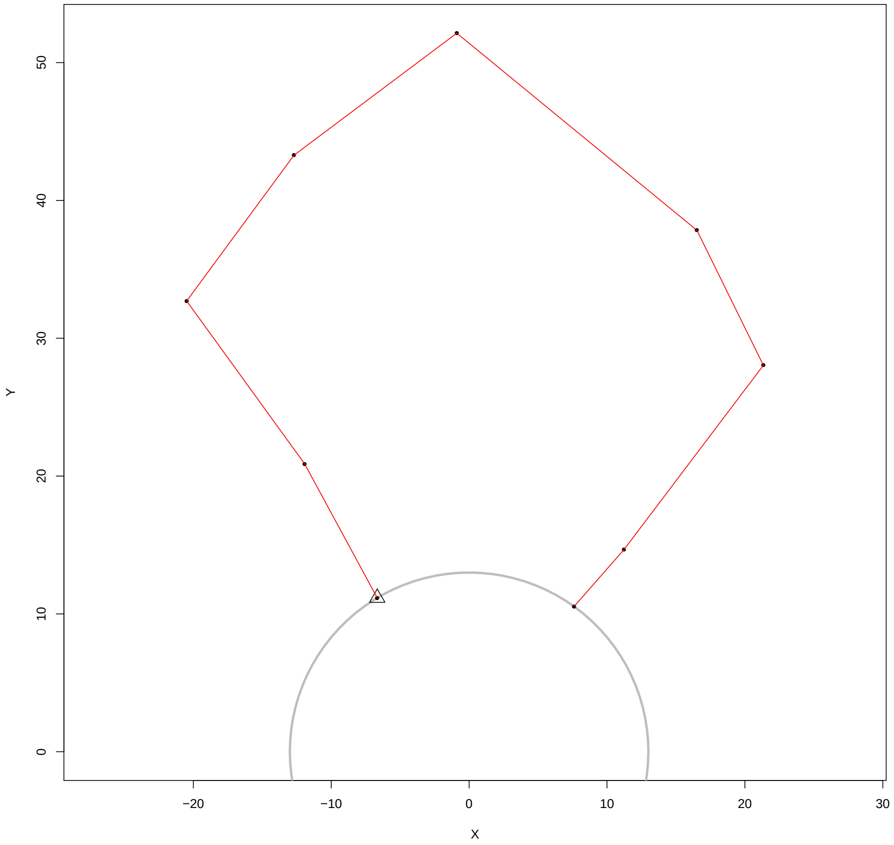

Fig SI-V-32.1 : Loop 203 — loop 1 of bee JY-1

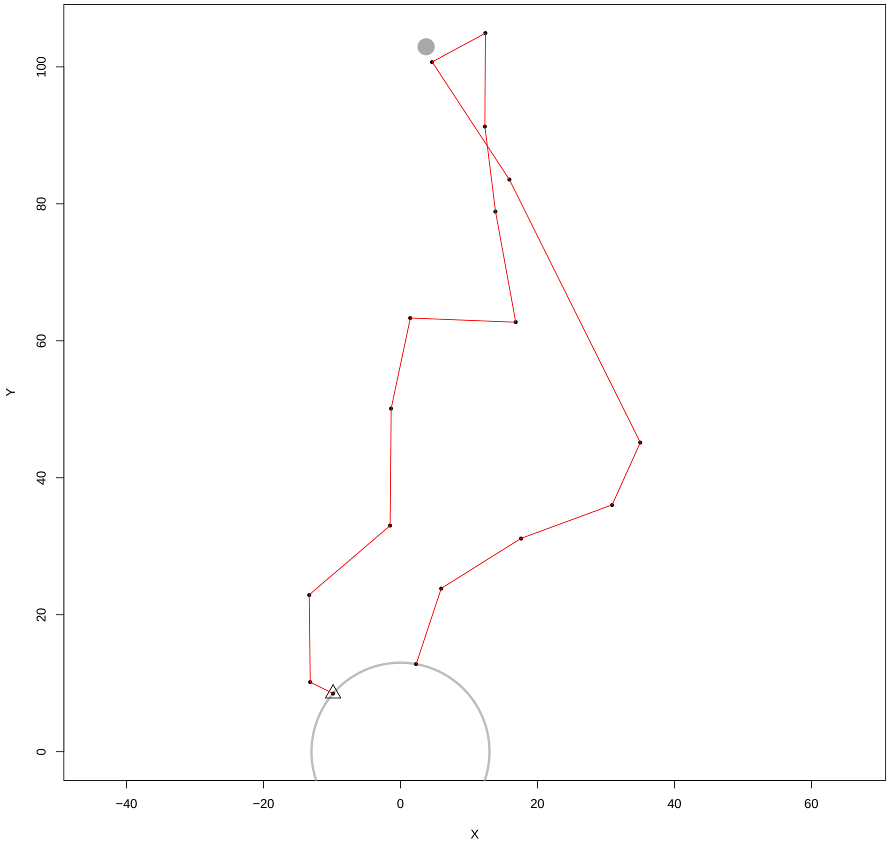

Fig SI-V-32.2 : Loop 204 — loop 2 of bee JY-1

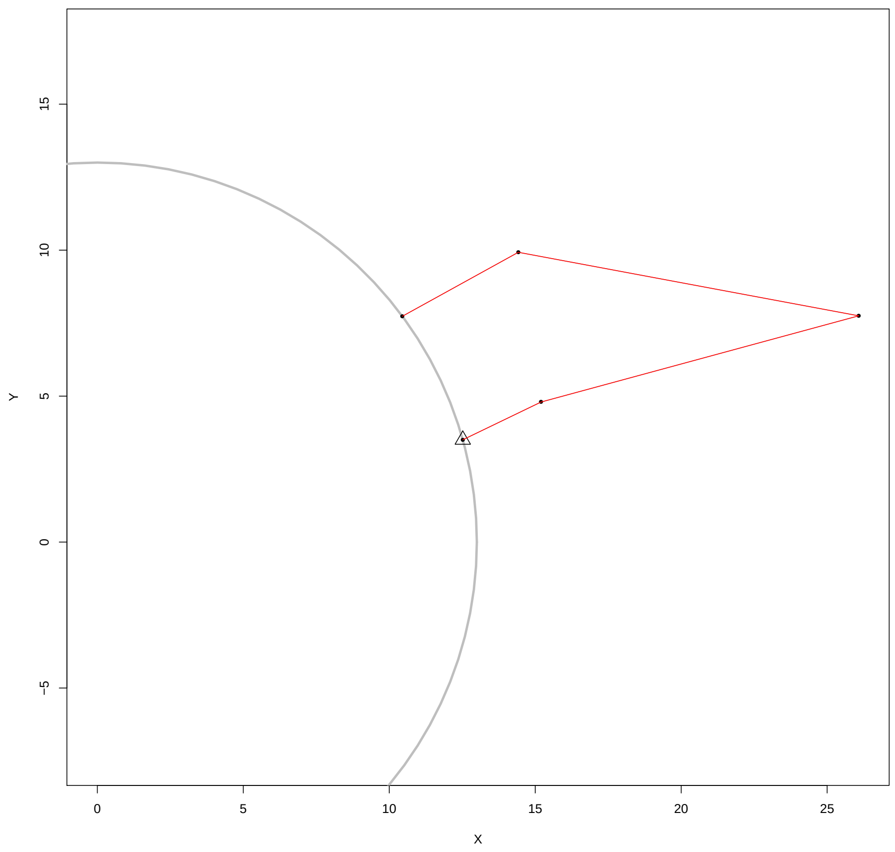

Fig SI-V-32.3 : Loop 205 — loop 3 of bee JY-1

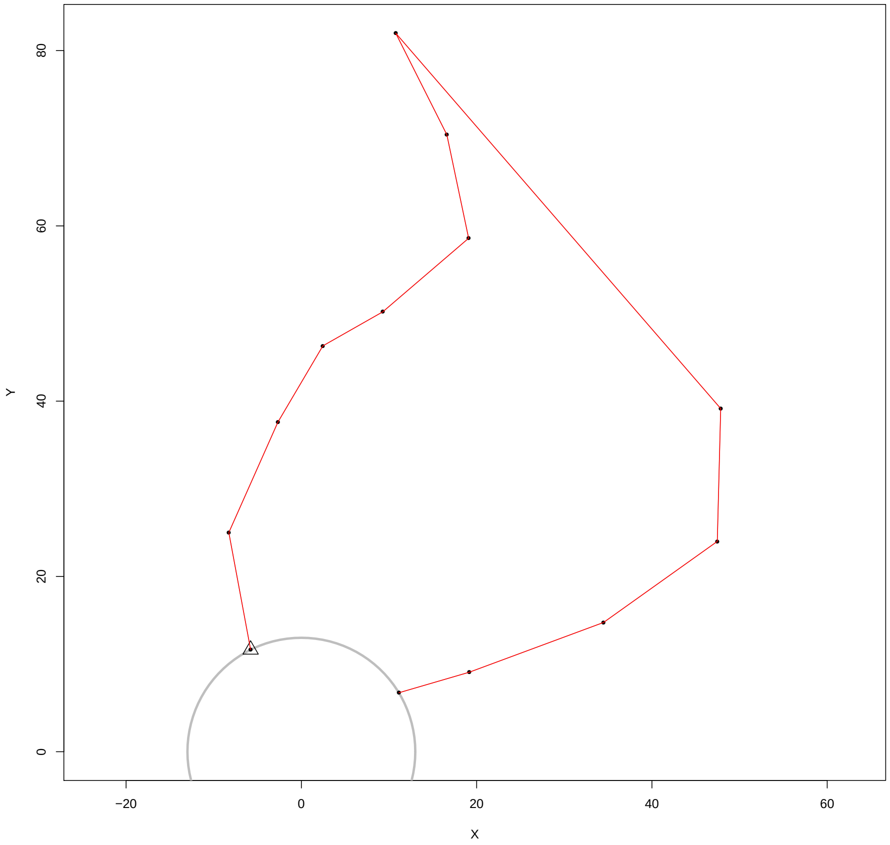

Fig SI-V-32.4 : Loop 206 — loop 4 of bee JY-1

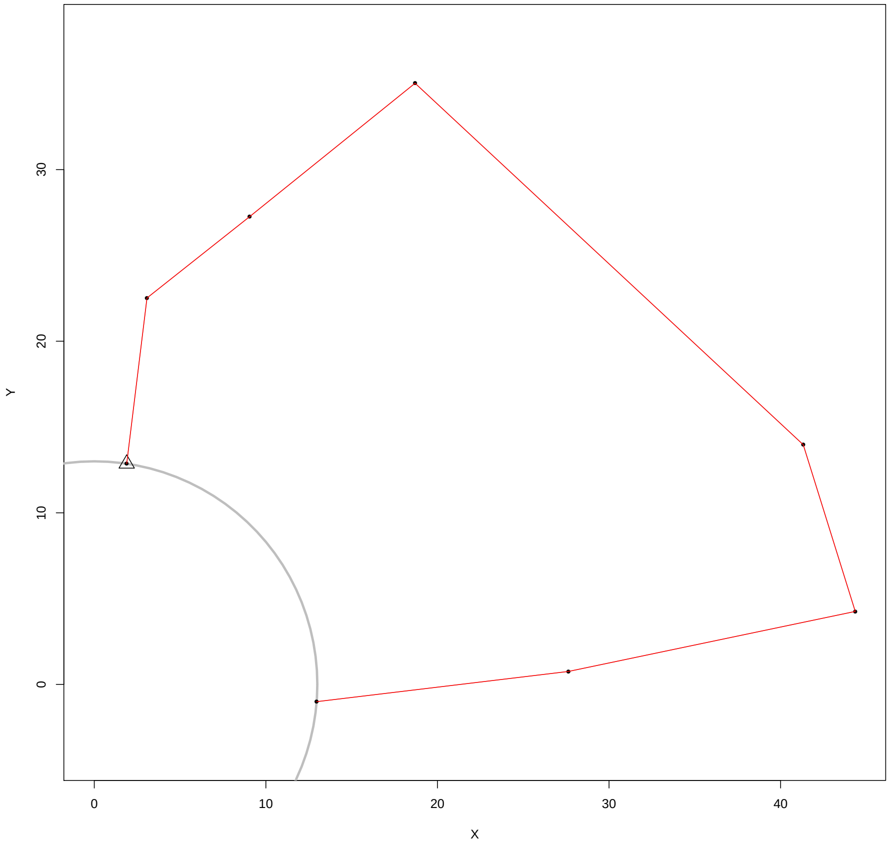

Fig SI-V-32.5 : Loop 207 — loop 5 of bee JY-1
