# Supplementary material for: Modeling bee movement shows how a perceptual masking effect can influence flower discovery
Source: PLoS Comput Biol. 2023 Mar 24;19(3):e1010558. doi: 10.1371/journal.pcbi.1010558 (PMC10075415; doi:10.1371/journal.pcbi.1010558)
Supplement: Attachment — Submitted filename: Author reponse.docx [file pcbi.1010558.s007.docx]

# Author’s responses to reviewers

## Reviewer #1:

**Dear Authors,**

**I find this to be a sound and well-designed model, and an important contribution to the body of bee models. It lays the groundwork for improvements in the modelling of bee exploratory behaviour and provides insights into some of the factors that could be affecting flower discovery beyond simple distance from the nest that is usually assumed. The manuscript provides a thorough assessment of the model, which is fitted using experimental data. I have just a few general comments that should clarify the biological insights resulting from the model and a few specific comments that should help readers to better understand the model.**

Thank you for your supportive comment.

**General Comments:**

1. **It is my understanding that your model mimics exploratory flight behaviour in which the bees are searching for flowers and learning the landscape, rather than directed foraging flights (such as are observed by Osborne et al (2013)/reference 40 in your manuscript or Woodgate et al (2016)/reference 14 in your manuscript). Naturally, a flower has to be discovered before it can be pollinated and, as you point out, this behaviour is under-studied and models generally assume that the bees are all-knowing or that discovery has a simple relationship with distance from the nest, so it's great to see a model that examines this behaviour in more detail. However, some of your results make it sound as if you are modelling the more focused foraging behaviour. For example, in line 188, the section title is “Distant flowers are more often visited in low-density environments”. I would suggest changing this to “Distant flowers are more frequently discovered in low-density environments” or something similar, as well as going through the results section to make sure that it is clear the topic is exploratory behaviour and flower discovery. I do find that your discussion section makes this distinction clear, however.**

Thank you for this important comment. We agree. The focus of our study was indeed exploration and flower discovery. Although this certainly has important consequences on foraging efficiency and pollination, our analyses did not specifically tackle these aspects. We, therefore, modified the title, the abstract (lines 14-21) and several parts of the results (lines 245-339) to clarify this point.

2. **I’m also curious as to why you chose to assume that the bee returns to the nest after discovering a single flower (or feeding location). How realistic is this for exploration behaviour? Do you think it leads to an overestimation of the masking effect?**

We made this assumption as we were interested in first flower discovery. This assumption holds true if we consider a large feeding location (such as a flower patch providing enough nectar for the bee to fill its crop to capacity). However, this is not true for many environmental conditions where flowers are isolated across the landscape. To account for this second scenario, we performed simulations where the bee returns to the nest after discovering more than a single flower (2, 4 or 8 flowers). The results remain the same (Figure 5B, lines 314-318 and supplementary figure S3).

3. **How does this behaviour fit in with exploration vs exploitation behaviour (to use the language of Woodgate et al (2016)) or the understanding that bumblebees use trapline foraging techniques?**

Our study only focuses on exploration behaviour. This was clarified throughout (see our response to your comment 1).

**Specific Comments:**

4. **Line 84: could you clarify what you mean by angular speed? If it includes both speed and direction, should it instead be angular velocity? Or is ω(t) only controlling the speed (frequency?) of direction changes?**

ω is indeed the angular speed (or turning speed) and refers to the rotational diffusion only, with the speed remaining constant. We have clarified this in line 128 by specifying the units.

5. **Line 90: should this be ω*(t)?**

Thank you for your comment. We corrected the typo.

6. **Line 300: I’m not clear on what you mean by “at different scales.”**

We modified the text and deleted that sentence. (L 377-384)

## Reviewer #2 :

**In the present manuscript the authors explore central-place foraging in bees with the help of a stochastic model. The model is essentially based on a random walk process based on a angular speed subject to a noise, together with an exploration/homing switching mechanism controlled by a constant switching rate. The parameters of the model are conveniently fitted to experimental data and the resulting numerical trajectories are used to explore the effects that target density/size have on the encounter probability between the bees and their targets (flowers).**

**While the starting point of the work is rather interesting (the idea that target density and/or clustering of targets may have an influence on the encounter probabilities) and the model/methodology used look reasonable, my impression after reading the manuscript is that there is nothing that this work adds on the knowledge we have about the encounter processes and/or central place foraging. To illustrate this, let me discuss on the "model predictions" that are provided in the main section of the manuscript, in their order of appearance:**

We thank you for your comments.

As we advocate below, we agree that, of course, general principles governing encounter processes have long been a general question, both in ecology and statistical physics.

Here, we add three main points to the topic:

- we propose a new model for rendering motion behavior, as close as possible to bumblebees’ ones, which does not obey nor brownian motion, nor run-and-tumble. This model combines previously published "Persistent Turning Walker" together with a steering mechanism acting on the turning speed,
- we calibrate this model using a dataset that is unprecedented,
- we show how this allows to numerically estimate realistic predictions with regards to interaction between bees and flowers.

We hope that our additions in response to your comments will clarify what is our purpose.

1. **Attraction to the nest limits the exploration range.**

**It is obvious that this must happen whenever a homing mechanism is introduced in the model. What the authors seem to obviate is that there is a large literature on stochastic processes where this effect has been accurately quantified in an analytical way. The probability distribution of diffusive models is well known (at least in the large-time regime), and the same is true for example for random walk models in which walkers return to the nest balistically with a given speed after a random time (see for example Masó et al. Physical Review E 100, 042104, and references there in). So, the main scaling properties of such central place mechanisms are well known (see also Pal et al. Physical Review Research 2, 043174). In case the present model can add something relevant to such known results then this should be discussed in detail, but instead the manuscript just discusses the effect of homing on the exploration range in a very qualitative and naive way.**

We agree that references to the literature in statistical physics were missing.

We have now added a full subsection ("Model Background") with a tentative overview of the most recent works in statistical physics we have found as being relevant to shed light on our choices for the model (lines 79 to 123 in the revised version).

While this literature is of great relevance as far as general principles are concerned, it is somewhat beyond reach for ecological community, and quite challenging for direct application in ecological quantitative modeling (for instance, results in Masó et al. 2019 are specific to Brownian Motion in 1D, and Pal et al. 2020 results are based on diffusion pde with ballistic returns).

We observe that our model (PTW x intermittent steering acting on the turning speed) does not appear (yet) in this literature, so there are no formal results for it published so far.

As you mention, theoretical results in ABP or Run-and-Tumble are derived at large-time regime.

More importantly for the case we deal with, they are derived in the diffusive limit (vanishing mean free path, or far-from-boundary regime), whereas this limit does not stand in our case: the mean free path of bumblebees, as they can be 'approximated by eye' in our trajectories or simulations are the same order as the typical distance from nest to flower (Knudsen number close to 1 or greater).

Moreover, we note that encounters with targets (MFPT or splitting probabilities) are formally predicted, in 2D, only in the limit of vanishing sizes (the so-called narrow capture problem, or diluted regime), precisely to avoid what we named as a "masking effect".

We would be glad, of course, to have theoretical predictions at hand in the future to derive quantitative predictions with no need to use Monte Carlo as the only option.

Still, your comment called for a further figure we have also added : how is the MSD of the NESS affected by the parameters of our behavioural model (now fig. S1) ?

An interesting point appeared there : since the steering acts upon the turning speed (and then, it is not a potential), and is constrained by a relaxation time, then it can not grow to infinite and show a threshold beyond which an increase has no more effect.

As a matter of fact, we also see that the MSD grows linearly with the characteristic time of resetting, a result we would be glad to see proven formally.

We comment on this on figure S1.

Also, we agree that our discussion of this topic is only qualitative. This section is intended as an illustration of this property of the model, and aimed to readers who are not experts in random walk models. Its main aim is to show how previous models used to describe bee movement have the unrealistic feature of being purely diffusive at long times, and how our model provides a more reasonable result. We have modified the text in lines 230-231 to make this clearer.

2. **Distant flowers are more often visited in low-density environments**

**Again this result is obvious and trivial, so the interest of the work could be on exploring how this effect actually occurs, but again the discussion provided is mostly qualitative. It is for example very easy to justify the exponential decay found in the encounter probabilities in Figures 4a and 4c. If one defines the habitat as divided into concentric regions around the nest, the probability to reach a target at a given distance is given by the probability to cross the inner regions previously. This "crossing probability" is obviously proportional to the area occupied by the targets, and so to the target density. This means that the characteristic decays in Figures 4a and 4c will be proportional to the density (except for very low densities, as in this case targets are so sparse that that inner regions barely contain any target. In that case the characteristic decay will tend to a constant value, as it is seen for lower densities in Figure 4c).**

We thank you for your suggestion.

We agree with your picture, that we understand as the classical Lambert's law.

If the bee were flying ballistically from the nest, and would be "absorbed" by a target with a constant probability per meter, we would indeed obtain an exponential attenuation along the distance, with a rate proportional to the absorption coefficient (here, the target density).

We are not sure that the picture would still stand with motion like diffusion-resetting, or scattering-reseting (e.g. Bressloff, 2020. “Target Competition for Resources under Multiple Search-and-Capture Events with Stochastic Resetting: Target Competition and Resetting.” Proceedings of the Royal Society A 476(2242)).

Here, the bee is not ballistic nor diffusive, so the exponential attenuation is convoluted by our NESS, for which we have no formal expression.

We think, however, that the exponential shape is indeed induced by the fact that bee's loops are closer to ballistic flight than to diffusive flights, with our parameters set.

In fig. 4D, the so-called discovery radius is a way to approximate the (inverse of) decay.

We estimated the decay using the exponential part between radii corresponding to 10^-2^ and 10^-3^ probability (Fig below, not included in the manuscript)

In low density, there is indeed an effect of sparsity within the area occupied by the NESS.

For larger densities, we see of course an increase of the decay, and it does not appear proportional to the density (albeit it may be an affine function of it).

3. **Bees find more flowers at intermediate densities**

**Finally, this result is based on a clear internal inconsistency in the work. As discussed in the manuscript, this optimal intermediate density appears because the homing mechanism is activated after a single flower is encountered, so producing the "masking effect". The authors justify this by saying that the bees will return to the nest to uncover their crop after each encounter. So, implicitly they are assuming that every single encounter is successful, it is, the crop is gathered after every single encounter. In consequence, it is inconsistent to say that "more successful foraging" is reached at intermediate flower densities just because more flowers are being encountered there. This is meaningless in a model where every encounter is assumed to be successful. To assess this question correctly it should be assumed that after a successful encounter the flower is exhausted, and so subsequent encounters with it (at least for a transient time) will be unsuccessful. Then an adequate measure of foraging efficiency could be conveniently proposed and evaluated.**

We agree that our results fell short of showing the relevance of this result. We have addressed this issue in the following ways:

- We have made it clearer throughout the text that we focus on flower discovery, and not foraging (see our response to comment 1 by reviewer 1). We changed the title, abstract, and significantly rephrased the results in order to remove the direct references to foraging success, now only mentioned in the discussion(from line 362-384). While this effect will influence foraging efficiency after the exploration phase, we find it sufficiently interesting and counter-intuitive even if it only pertains to flower discovery.
- To ensure the robustness of our result, we analyzed the case in which each individual visits more than one flower before returning to the nest. We find that all our results remain unchanged, regardless of this number. This is now explained at lines 314-318 and in figure 5B.
- You correctly pointed out that if flowers are completely depleted after one visit, and subsequent bees don’t react at all to depleted flowers, then our result won’t hold. We agree that flower depletion may affect exploration and subsequent flower discovery to some degree, but assuming that bees don’t react at all to previously visited flowers is too extreme and unlikely in most realistic scenarios. First, flowers are rarely fully depleted after a single exploration visit. Second, even if a bee discovers a fully-depleted flower, it must still land on it to assess its level of depletion, incurring costs in energy and time. To make sure that our conclusions are robust in more realistic scenarios, we performed an additional simulation: An individual will continue exploring after visiting an already-explored flower, and will return to the nest only when encountering a fresh flower or after a fixed number of visits to already-visited flowers. This maximum number of visits takes into account the visit cost and partial benefits discussed above. This alternative model finds the same qualitative results as our original manuscript, including the maximum number of discovered flowers at intermediate densities (lines 318-329 and figure S3).

## Reviewer #3:

**This article studies how bees search for flowers by developing an individual-based realistic model and comparing with data. The model of bee search movement takes into account the returning to the nest, i.e., the tendency of these insecdts to make loops around their nest. One of the important results obained is the masking effect, so that detection of flowers close to others can be reduced. These are very nice results, novel and the authors try to connect with realistic measurments. Moreover, the paper is very clearly written and thus I recommend this work for publication after the authors have addressed the following minor corrections/suggestions:**

1- **The average value of tau, the switching time, is 1/preturn. In their calculations this is 30 seconds. Is this time enough for the exploratory phase when the bees move some kilometers (as it happens some times)? Please clarify.**

A clarification was added line 211-215

2- **Fig 2 is labelled with Fig.3. Please correct.**

Thank you for pointing this out. We corrected the label.

3- **In the section of "Calibration with experimental data" the authors talk about "2D exploratory flight trajectories", but the data is for both exploratory and return phases. Am I correct? Please clarify.**

We clarified by replacing "exploratory flight trajectories" with "trajectories during exploration flights" (line 179)

4- **The fit to optimal parameters with experiment should be clarified in the appendix. However, there are nowadays many techiniques coming from maching learning (gradient descent and variants) to minimize cost functions, and obtain optimal fittings. Not for this article, but the authors should consider (and comment) this possibility for future works.**

We have clarified the fitting procedure in Methods (lines 475-513). We agree that using alternative fitting techniques might be interesting, but we found them unnecessary given that we could ensure finding the global minimum by exhaustive exploration of the parameter space. We preferred not to add a discussion of this point in the manuscript, considering that our four-dimensional cost function space combine two continuous functions (loop length and farther distance away) with two discrete functions (number of self-crossing and number of re-departure) so that gradient descent techniques are not straightforward to implement without proper scaling choices on each axis.
We are aware that this choice averaging across quantiles computed on each separate axis does not avoid exhaustive and then costly exploration of space parameters. It appeared as a robust one however. We would, of course, welcome a faster procedure if any one becomes available for such a context.

5- **Eq. M2 is not correct. It should be v(t+Delta t). Right?**

We rearranged the equations so it's easier to understand (lines 440-461)

6- **The very interesting effect of masking reminds me that of "excess or defect of information is bad for searching of resources" as obtained in some articles of foraging (see for example, Martinez-Garcia et al, Physical Review Letters (2013)). Maybe the authors consider this paper worth of dicussing in their Discussion section.**

Thank you for pointing out this paper. This is an interesting parallel to our study. However, we feel it is a bit far from our consideration of the effects of the structure of the foraging environment on flower discovery by bees and therefore decided not to discuss it in the revised manuscript.

7- **They may also consider to discuss some extensions of their model to include the interaction of the bees. I find this very interesting for the future.**

Thank you for your comment. One of the aims of our model is to include it into existing learning and exploitation models that already takes into account the interaction of the bees. This was discussed in lines 389-391.
