# Supplementary material for: Modeling bee movement shows how a perceptual masking effect can influence flower discovery
Source: PLoS Comput Biol. 2023 Mar 24;19(3):e1010558. doi: 10.1371/journal.pcbi.1010558 (PMC10075415; doi:10.1371/journal.pcbi.1010558)
Supplement: Attachment — Submitted filename: Authors response.docx [file pcbi.1010558.s008.docx]

**Authors' response**

Dear Editor,

Thank you very much for accepting to take into consideration our appeal request and allowing us to re-submit a revised version of the manuscript. We highly appreciate the time the Editors took to verify our claims and change their initial decision. Please find attached a revised manuscript that takes into account the minor suggestions of the Editors. In what follows we explain in details how we have modified the manuscript.

All the very best,

Ana Morán, Mathieu Lihoreau, Alfonso Pérez Escudero, Jacques Gautrais

**Revision suggestions**

- *It would be useful to edit the caption of Fig. 3D to mention that orange lines correspond to trajectories without homing and thus the model is not stationary in that limit.*

Thank you for your suggestion. The caption of Fig 3D now reads: “**(D)** Probability to find a bee below a given distance to the nest (i.e., inverse cumulative probability distribution for the distance to the nest) after different amounts of time. Blue: model with attraction (stationary distribution of bees). Orange: model without attraction (non-stationary distribution of bees)."

- *There appears to be a typo in Eq. (3) between tau and tau'. If that's not a typo, please explain what is tau'.*

It was indeed a typo. Eq. (3) now reads:

$$\left\{ \begin{aligned} \eta\left( t \right)=0 \mathrm{if}t<\tau\\ \eta\left( t \right)=\eta^{*}\mathrm{if}t\geq\tau\end{aligned} \right.$$

- *It would be useful to include a few words after the sentence in line 123 discussing why implementing resetting explicitly is important in the context of fully parameterizing the model using empirical data. Someone could think that the empirical trajectories can be segmented to remove the homing phase and use only the exploration phase of bee movement to parameterize the model.*

Thank you for this suggestion. Lines 123-125 now reads: “Thanks to this steering potential, our model describes the full exploration trips of bees (starting and ending at the nest location), which facilitates comparisons between model simulations and experimental data.”
